# A versatile route towards 6-arylpipecolic acids

**DOI:** 10.3762/bjoc.21.88

**Published:** 2025-06-04

**Authors:** Erich Gebel, Cornelia Göcke, Carolin Gruner, Norbert Sewald

**Affiliations:** 1 Department of Chemistry, Organic and Bioorganic Chemistry, Bielefeld University, Universitätsstraße 25, D-33615 Bielefeld, Germany

**Keywords:** conformational restraints, dihedral angle NMR, half-chair conformation, modified amino acids, pipecolic acid, stereoselective hydrogenation, Suzuki–Miyaura cross-coupling

## Abstract

Pipecolic acid is known as a non-proteinogenic amino acid with a secondary amine. It contains a six-membered ring and is, like its five-membered correlate, known for its secondary structure inducing properties, which are particularly useful in the design of peptide conformations. We present a new and improved way to generate enantiomerically pure pipecolic acid derivatives with aryl modifications in C^6^ position by utilising the chiral pool of a non-proteinogenic amino acid in combination with transition metal-catalysed cross-coupling reactions. Moreover, we present an in-depth NMR analysis of the key intermediate steps, which illustrates the conformational constraints in accordance with coupling constants and resulting dihedral angles.

## Introduction

Non-proteinogenic amino acids play an important role as building blocks for peptide synthesis [1–5], as organocatalysts [6–10] and as enzyme inhibitors [4,11–13]. The incorporation of such amino acids into peptides can, for example, influence peptide conformation, the binding affinity to receptors [14], as well as pharmacokinetics [15–16], stability against degradation [17] and general stability [10,18] of the peptide [19]. One of those amino acids is pipecolic acid [20–21], a homolog of proline with a six-membered piperidine ring. Pipecolic acid has similar features as proline in regard to its rigid nature and turn-inducing properties in peptides [22–24]. Furthermore, derivatives of pipecolic acid are known for their bioactivity as secondary metabolites [25–27] and for being building blocks for piperidine alkaloids [28] with a variety of uses.

## Results and Discussion

**Scheme 1:**
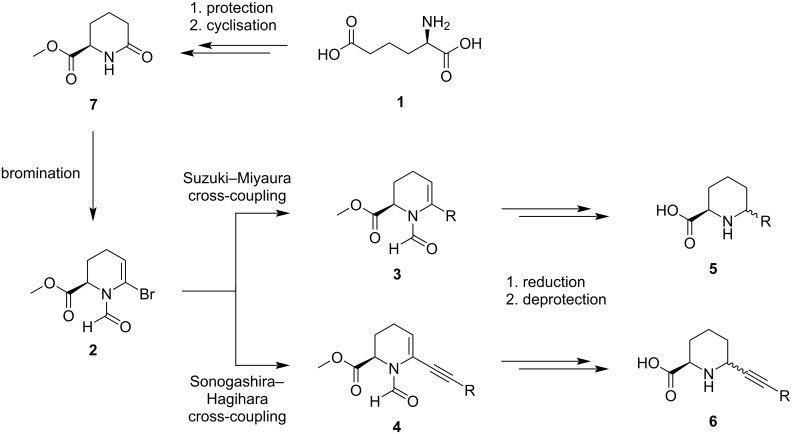
ᴅ-2-Aminoadipic acid (**1**) can be used to generate C^6^ aryl and alkynyl-modified pipecolic acid derivatives.

Addressing specific positions in the ring structure of pipecolic acid is rather challenging and often necessitates early-stage derivatization followed by the formation of the six-membered ring [29–32]. An alternative is to utilise derivatization reactions such as Suzuki–Miyaura [33] or Sonogashira–Hagihara [34] cross-coupling reactions on a key intermediate product. This late-stage approach was previously described by us while utilising Suzuki–Miyaura or Sonogashira–Hagihara cross-coupling reactions to generate pipecolic acid derivatives with alkynyl substituents in the C^6^ position [35]. Here, we present a robust synthetic route to C^6^-aryl-modified pipecolic acid derivatives, employing non-proteinogenic amino acids and cross-coupling reactions (Scheme 1) with an emphasis on scaling up the reaction. The cross-coupling products **3** and **4** require a saturation of the double bond, which previously was successful only in the case of the Sonogashira–Hagihara cross-coupling products [35]. Furthermore, an in-depth NMR analysis was conducted on the resulting constraints leading to conformational structure predictions.

**Scheme 2:**
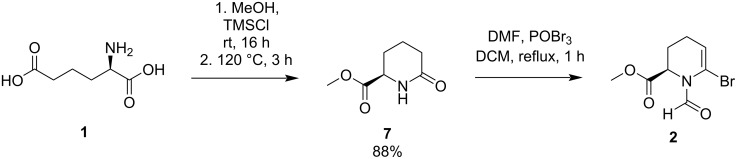
Methyl ester formation, followed by cyclization, *N*-formylation, as well as bromination under Vilsmeier–Haack conditions.

ᴅ-2-Aminoadipic acid (**1**) [36], a side product formed in the pharmaceutical semisynthesis of 7-aminocephalosporanic acid (7-ACA) from cephalosporin C by cephalosporin C acylase [37], was used as a chiral starting material. After esterification of both carboxylic acid functions followed by cyclization to the lactam [38–39], the six-membered ring structure was established as (*R*)-methyl 6-oxopipecolate (**7**) with a high yield of 88%. (*R*)-Methyl 6-oxopipecolate (**7**) was converted under Vilsmeier–Haack conditions [35,40–41] to undergo *N*-formylation and concomitant enol bromination to give product **2** (Scheme 2). Due to slow degradation of the bromide **2**, the subsequent cross-coupling reaction was conducted immediately after workup. Purifying the bromide **2** via column chromatography was deemed unnecessary after comparing the NMR spectra of the worked-up and the purified product. While the worked-up product **2** exhibited only slight differences in impurities, the yield was significantly reduced due to purification by column chromatography. The vinyl bromide **2** had been shown to undergo palladium-catalysed cross-coupling reactions, e.g., Suzuki–Miyaura or Sonogashira–Hagihara cross-coupling reactions [35]. Emphasis was placed on the Suzuki–Miyaura reaction to give a variety of arylated compounds, as previous attempts to reduce the arylated *N*-formyl enamine moiety in **3** remained unsuccessful. We also aimed at improving conversion and yield by first testing different bases and catalysts [42–43]. DMF, a well-established solvent for cross-coupling reactions, led to lower degradation of the bromide **2** compared to other solvents.

Table 1 provides an overview (a more detailed table can be found in Supporting Information File 1), which catalysts and bases are showing the best results. Most of the bases did not change the conversion drastically, apart from Et_3_N, which shows the least conversion regardless of the catalyst. This tendency of amine bases in cross-coupling reactions aligns with the literature [44–46], reporting that they are either superior to or significantly outperformed by oxygen-based bases, depending on the conditions and substrates. K_2_CO_3_ was found to be the most suitable base, having similar performance to Cs_2_CO_3_ including the lack of methyl ester hydrolysis, but a lower price.

**Table 1:** Screened conditions for the Suzuki–Miyaura cross-coupling between bromide **2** and phenylboronic acid (**8a**).

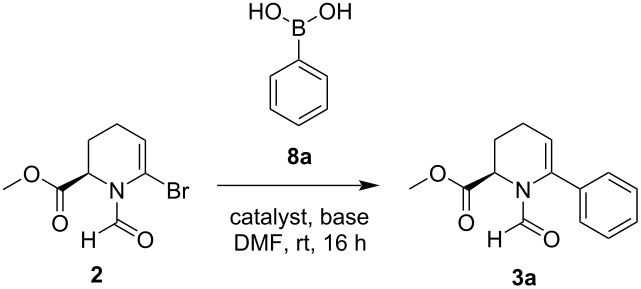		

Catalysts^a^	Base^b^	Conversion

PdCl_2_(PPh_3_)_2_	Et_3_N	72%
PdCl_2_(PPh_3_)_2_	Cs_2_CO_3_	88%
PdCl_2_(PPh_3_)_2_	K_2_CO_3_	81%
Pd(PPh_3_)_4_	Et_3_N	61%
Pd(PPh_3_)_4_	Cs_2_CO_3_	75%
Pd(PPh_3_)_4_	K_2_CO_3_	76%
Pd(OAc)_2_	Et_3_N	–
Pd(OAc)_2_	Cs_2_CO_3_	15%
Pd(OAc)_2_	K_2_CO_3_	13%
Pd(dppf)Cl_2_	Et_3_N	67%
Pd(dppf)Cl_2_	Cs_2_CO_3_	99%
Pd(dppf)Cl_2_	K_2_CO_3_	99%
XPhos Pd G2	K_2_CO_3_	80%

^a^10 mol % of catalyst was used; ^b^2.0 equiv of base were used except for Et_3_N with 4.0 equiv.

The catalyst's performance had a more significant influence on the reaction results than the base. Both phosphine catalysts as well as the second generation of the Buchwald–Hartwig catalyst [47–48] gave similar results under the same conditions. The advantage of XPhos Pd G2 is the use of an aqueous solution without inert conditions, but even under these conditions, the conversion was lower than in DMF. Pd(OAc)_2_ showed the lowest conversion by far, with no conversion observed when Et_3_N was used as the base. Overall, the best results were achieved with Pd(dppf)Cl_2_. The conversion was among the highest overall regardless of base and the removal of the catalyst afterwards was most straightforward. While the phosphine-based catalysts tend to be oxidised during the workup, resulting in the contamination of the arylated products **3** with triphenylphosphine oxide, this issue does not occur using Pd(dppf)Cl_2_.

**Scheme 3:**
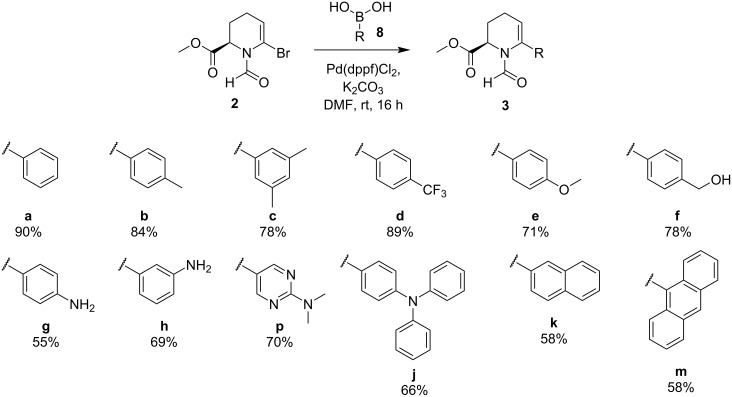
Suzuki–Miyaura cross-coupling reaction between bromide **2** and a variety of boronic acids **8**.

The best conditions for the cross coupling were determined to be 5 mol % Pd(dppf)Cl_2_ as catalyst, 2.0 equiv K_2_CO_3_ as base, 1.5 equiv of the required boronic acid and DMF as the solvent. In addition, a minute amount of water was added to activate the boronic species and to dissolve the inorganic base under otherwise inert conditions. The reaction mixture was then stirred at room temperature overnight. Under these conditions, a variety of boronic acids with different steric and electronic properties was coupled with yields ranging from 50 to 90% (Scheme 3).

**Scheme 4:**
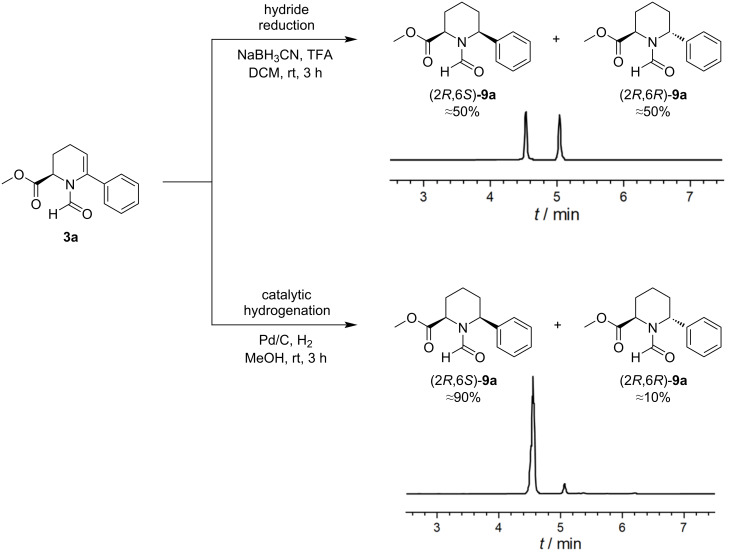
Reaction of **3a** to (2*R*,6*S*)-**9a** and (2*R*,6*R*)-**9a**. The chromatograms prove the simple diastereoselection.

Once the cross-coupling has been performed, the next step included establishing the piperidine motif through hydrogenation or reduction of the *N*-formyl enamine thereby introducing a second stereocenter in C^6^ position. We decided to use two approaches to investigate how the configuration of the stereocenter in C^2^ position influences diastereoselectivity. In the first approach, NaBH_3_CN was used under acidic conditions to reduce the acyliminium intermediate formed from the *N*-acyl enamine upon protonation at C^5^, while in the second approach, heterogeneous catalytic hydrogenation of the enamine with palladium on carbon was chosen. While the hydride reduction of the acyliminium intermediate gave a nearly 1:1 diastereomer ratio, a 9:1 ratio was obtained for the catalytic hydrogenation (Scheme 4). While the hydride reduction of the *N*-acyliminium species did not show any significant diastereofacial discrimination, the catalytic hydrogenation occurs stereospecifically, particularly in the case of hydrogenation with palladium on carbon [49–52]. In this case, we propose that the restraints exerted by the first stereocenter lead to a kinetically controlled diastereofacial selectivity.

**Figure 1:**
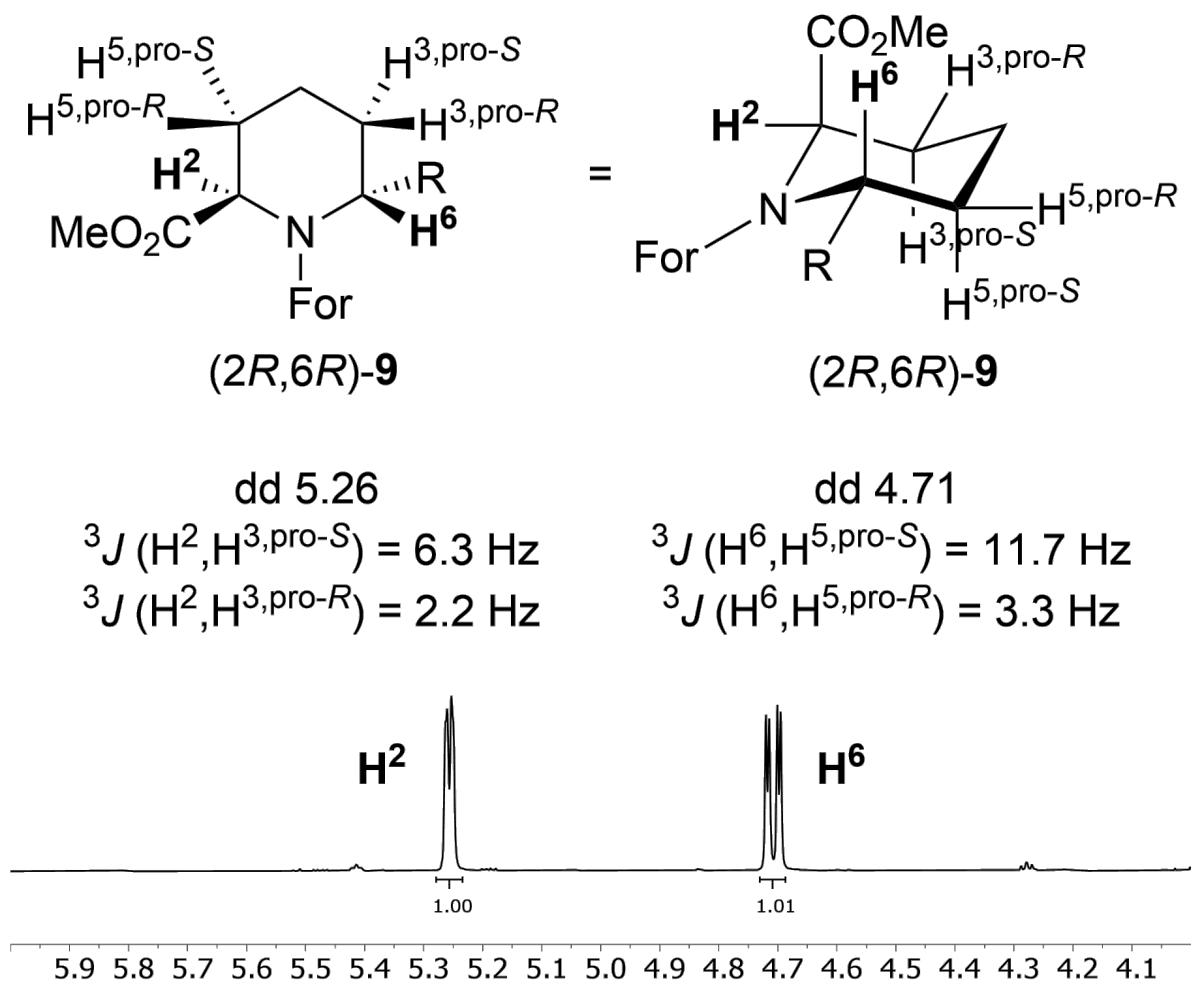
The minor diastereomer of the catalytic hydrogenation was assigned as (2*R*,6*R*)-**9**, based on the analysis of the coupling constants for H^2^ and H^6^.

The resulting diastereomers are separable by chromatography. ^1^H NMR was used to assign the configuration of the 2 diastereomers [53–55]. Carbon C^2^ is *R*-configured, as ᴅ-2-aminoadipic acid (**1**) was employed as the starting material. The minor diastereomer obtained by catalytic hydrogenation, assigned as (2*R*,6*R*)-**9**, gave a single set of signals (Figure 1). Based on the observed coupling constants for (2*R*,6*R*)-**9**, H^2^ adopts an equatorial position as indicated by the coupling constants ^3^*J*(H^2^,H^3,pro-^*^R^*) = 2.2 Hz and ^3^*J*(H^2^,H^3,pro-^*^S^*) = 6.3 Hz, corresponding to slightly distorted gauche couplings with torsion angles of approximately 60° and −60°, respectively. By analogy to a cyclohexane ring, the carboxylate of (2*R*,6*R*)-**9** would be expected to adopt an equatorial position rather than an axial one. However, it is known from *N*-acyl pipecolic acid derivatives, that the carboxylate (or carboxamide) preferably occupies the axial position, as the equatorial position is disfavoured because of pseudoallylic strain exerted by the partial double bond character of the *N*-acyl bond [56]. Conversely, the aryl substituent at C^6^ is assumed to adopt an equatorial position as evident by an antiperiplanar coupling of the axially positioned H^6^ with ^3^*J*(H^6^,H^5,pro-^*^S^*) = 11.7 Hz and a gauche coupling with ^3^*J*(H^6^,H^5,pro-^*^R^*) = 3.3 Hz.

**Figure 2:**
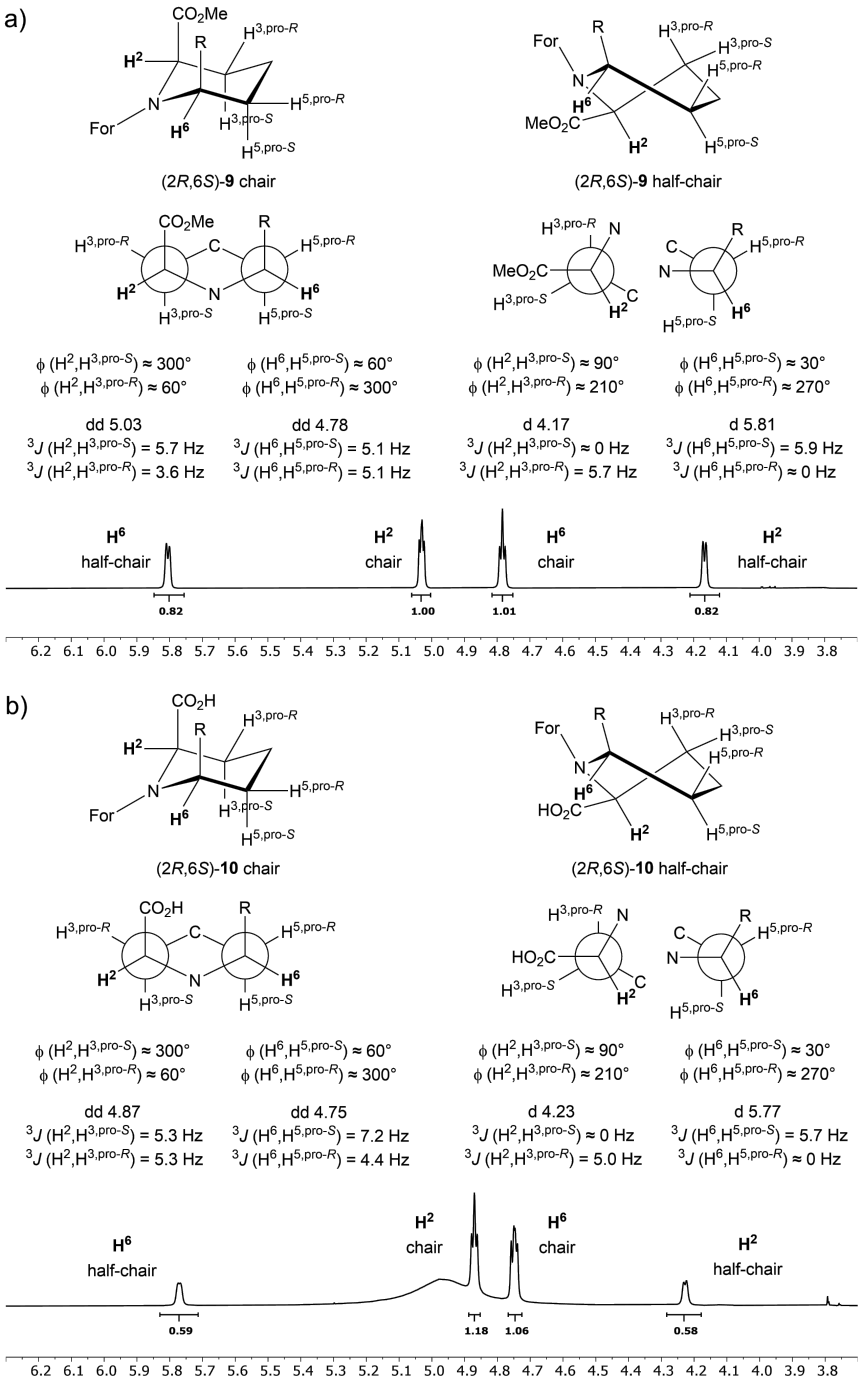
^1^H NMR spectra with both signal sets for the chair and half-chair configuration as well as Newman projection for both protons H^2^ and H^6^ with corresponding dihedral angles ϕ for a) (2*R*,6*S*)-**9**, b) saponification product (2*R*,6*S*)-**10**.

The major diastereomer, assigned as (2*R*,6*S*)-**9** was found as a mixture of conformers in solution (Figure 2a). Like for (2*R*,6*R*)-**9**, one conformer of (2*R*,6*S*)-**9** adopts a chair with only gauche couplings for H^2^ [^3^*J*(H^2^,H^3,pro-^*^S^*) = 5.7 Hz and ^3^*J*(H^2^,H^3,pro-^*^R^*) = 3.6 Hz]. Similarly, the configuration at C^6^ becomes evident by gauche couplings only between H^6^ and H^5^ [^3^*J*(H^6^,H^5,pro-^*^S^*) = ^3^*J*(H^6^,H^5,pro-^*^R^*) = 5.1 Hz] with an axial position for the aryl substituent at C^6^ as well as an axial position for the methyl ester at C^2^.

All spectra of the stereoisomer (2*R*,6*S*)-**9** contain signals for a second data set. This phenomenon might be associated with *cis-/trans*-isomerism around the formamide bond. Maison et al. noted that a phenyl substituent at C^6^ of *N*-acyl pipecolic acid derivatives exclusively leads to a *cis*-amide bond [57]. Most noteworthy are the signals of both protons H^2^ and H^6^, which display a different coupling pattern and a notable shift around 1 ppm upfield for H^2^ and around 1 ppm downfield for H^6^. Examination of the coupling constants of both these protons shows broad doublets instead of double-doublets (Figure 2a,b). The lack of a second coupling constant indicates a conformation in between a chair and a boat with a dihedral angle ϕ near 90°, which is necessary for a coupling constant to be around 0 Hz. Half-chair or twist-boat conformations are well known for being intermediate conformations in six-membered rings with dihedral angles ϕ near 90° (a front and side view of cyclohexane is shown in Figure S1, Supporting Information File 1). In case of a twist-boat conformation all dihedral angles for H^2^ would ultimately result in double-doublets for ^3^*J*(H^2^,H^3,pro-^*^S^*) and ^3^*J*(H^2^,H^3,pro-^*^R^*). Therefore, a half-chair conformation with ϕ(H^2^,H^3,pro-^*^S^*) = 90° and ϕ(H^6^,H^5,pro-^*^R^*) = 270° resulting in ^3^*J*(H^2^,H^3,pro-^*^S^*) = ^3^*J*(H^6^,H^5,pro-^*^R^*) ≈ 0 Hz and ^3^*J*(H^2^,H^3,pro-^*^R^*) = 5.0 Hz as well as ^3^*J*(H^6^,H^5,pro-^*^S^*) = 5.7 Hz (as shown in Figure 2a,b) would be the most suitable explanation. The co-existence of *cis*-/*trans*-isomers around the *N*-formyl bond instead of the proposed conformational isomers would not change the coupling pattern and can, therefore, be ruled out.

**Figure 3:**
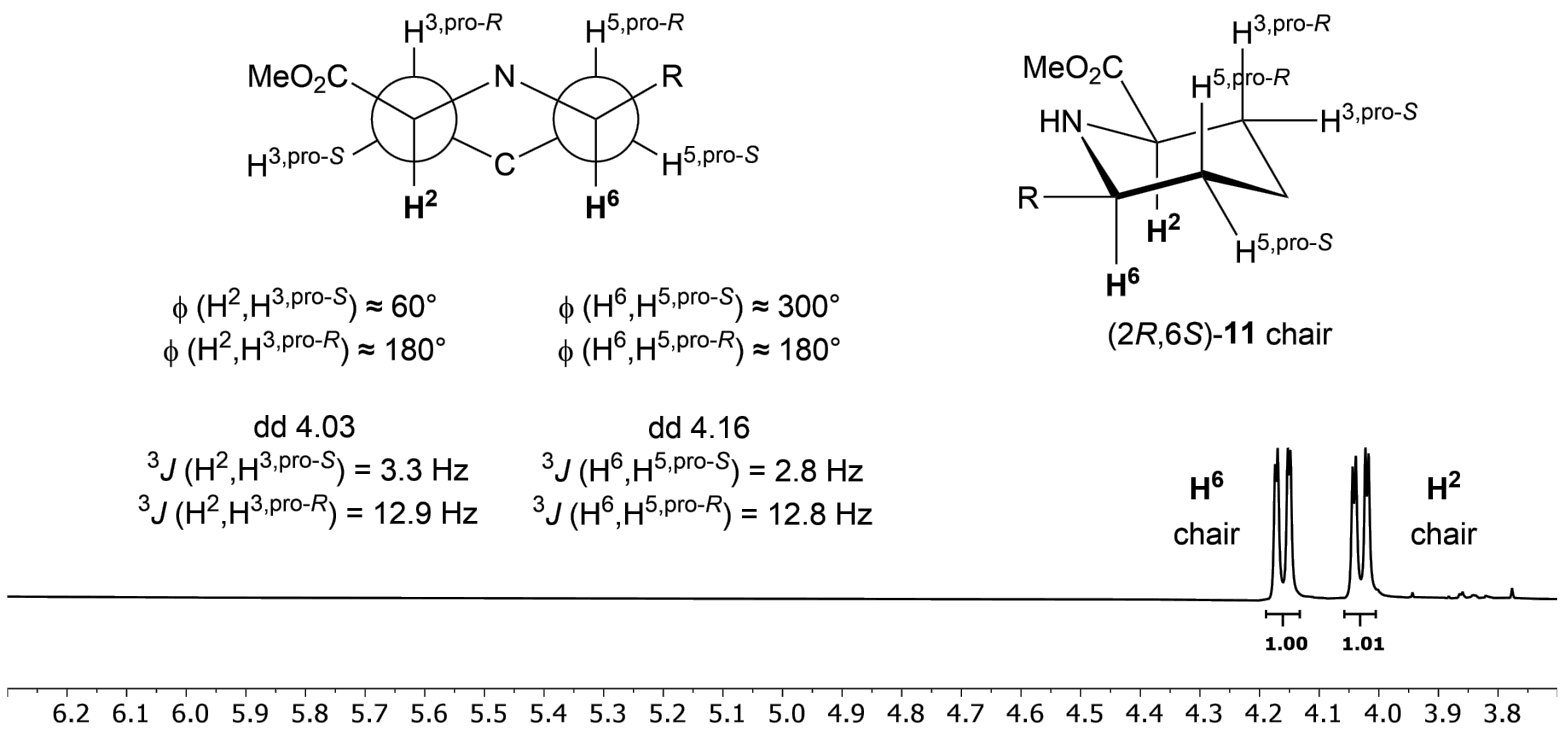
^1^H NMR spectra with signal set for the chair configuration as well as Newman projection for both protons H^2^ and H^6^ with corresponding dihedral angles ϕ for deformylated product (2*R*,6*S*)-**11** with one signal set.

A complete flip of the conformation is not likely due to the partial double bond between the formyl group and nitrogen in the amide bond and their resulting restraints. The coupling patterns of H^2^ and H^6^ do not change upon hydrolysis of the methyl ester, resulting in product (2*R,*6*S*)-**10**, where the second set of signals still remains (Figure 2b). However, cleaving the formyl group, on the other hand, leads to product (2*R,*6*S*)-**11**, with only one set of ^1^H NMR signals, and, hence, one conformer. In addition, for the unprotected (2*R,*6*S*)-**11** the coupling constants ^3^*J*(H^6^,H^5,pro-^*^R^*) = 12.8 Hz for proton H^6^ and ^3^*J*(H^2^,H^3,pro-^*^R^*) = 12.9 Hz for proton H^2^ indicate diaxial couplings, which in return confirms a flip to the more favourable chair conformation with the aryl substituent and the methyl ester in equatorial positions (Figure 3), providing further evidence for conformational restraints (pseudoallyl strain) enforced by the formyl group.

This behaviour is observable in all NMR spectra of the compound array of the reduction products **9**, with slight variance in chemical shift and coupling constants, but they always display two data sets for the (2*R*,6*S*)-**9** isomers and one set for (2*R*,6*R*)-**9** isomers (Table 2, a comprehensive table can be found in Supporting Information File 1).

**Table 2:** Overview of the relevant compounds with their chemical shifts δ, multiplicity, coupling constants *J* and dihedral angles ϕ for both protons H^2^ and H^6^ as well as the resulting conformation for the compound.

Product	Class δ (H^2^) [ppm]	^3^*J*(H^2^,H^3,pro-^*^S^*) ^3^*J*(H^2^,H^3,pro-^*^R^*) [Hz]	ϕ(H^2^,H^3,pro-S^) ϕ(H^2^,H^3,pro-R^) [°]	Class δ (H^6^) [ppm]	^3^*J*(H^6^,H^5,pro-S^) ^3^*J*(H^6^,H^5,pro-R^) [Hz]	ϕ(H^6^,H^5,pro-S^) ϕ(H^6^,H^5,pro-R^) [°]	Conf.

(2*R*,6*S*)-**9**	dd 5.03	5.7 3.6	300 60	dd 4.78	5.1 5.1	60 300	chair
(2*R*,6*S*)-**9**	d 4.17	0 5.8	90 210	d 5.81	5.9 0	30 270	half-chair
(2*R*,6*R*)-**9**	dd 5.26	6.3 2.2	300 60	dd 4.71	11.7 3.3	180 60	chair
(2*R*,6*S*)-**10**	dd 4.87	5.3 5.3	300 60	dd 4.75	7.2 4.4	60 300	chair
(2*R*,6*S*)-**10**	d 4.23	0 5.0	90 210	d 5.77	5.7 0	30 270	half-chair
(2*R*,6*S*)-**11**	dd 4.03	3.3 12.9	60 180	dd 4.16	2.8 12.8	300 180	chair
(2*R*,6*S*)-**13_I_**	d 5.11	4.7 0	330 90	d 5.06	0 6.0	270 210	half-chair
(2*R*,6*S*)-**13_II_**	d 4.68	0 5.8	90 210	d 5.39	4.8 0	30 270	half-chair
(2*R*,6*R*)-**13**	dd 4.97	4.2 4.2	300 60	dd 4.64	10.3 3.3	180 60	chair

**Scheme 5:**
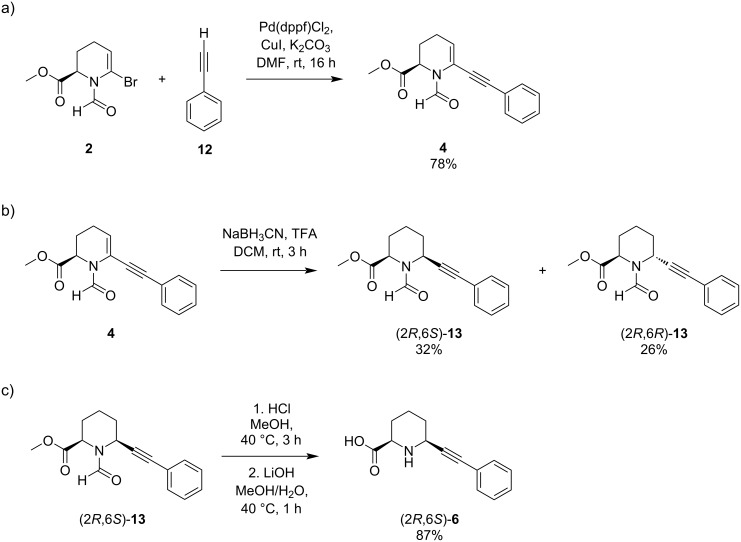
a) Sonogashira–Hagihara cross-coupling reaction followed by b) NaBH_3_CN reduction of the *N*-acyliminium species and c) deprotection.

A similar behaviour can also be observed for the reduction product **13** of a Sonogashira–Hagihara cross-coupling reaction. This was synthesised under similar conditions as the Suzuki–Miyaura counterpart **3** utilising the same Pd catalyst, base and solvent. In addition, 10 mol % CuI as a co-catalyst and 2.0 equiv phenylacetylene (**12**) were used, resulting in product **4** (Scheme 5a). The transformation of the enamine had to be carried out by reduction of the *N*-acyliminium ion by NaBH_3_CN in the presence of TFA, as Pd/C with H_2_ would lead also to the reduction of the triple bond. The products (2*R*,6*S*)-**13** and (2*R*,6*R*)-**13** were obtained in a 1:1 ratio (Scheme 5b).

**Figure 4:**
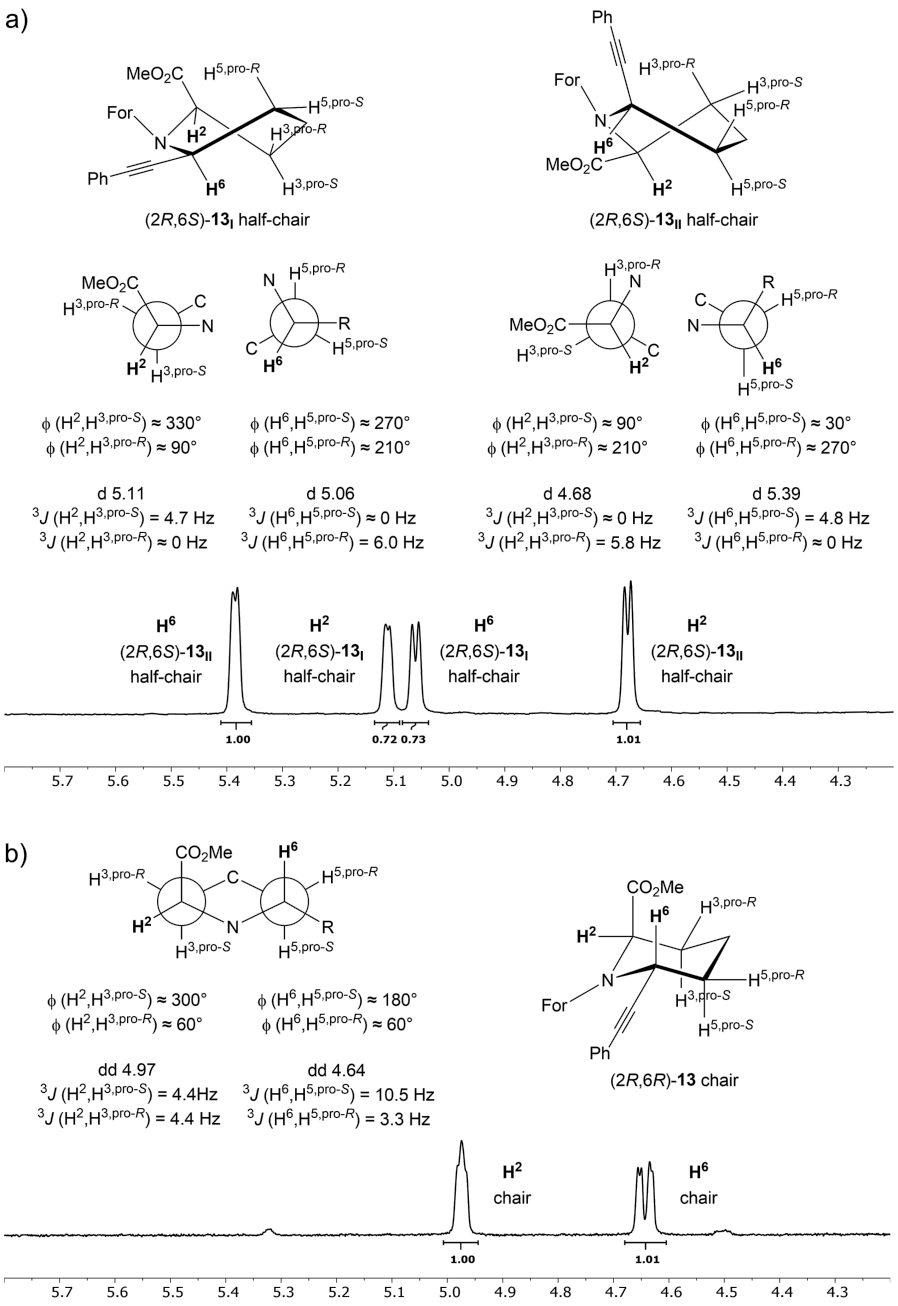
^1^H NMR with Newman projection for both protons H^2^ and H^6^ with corresponding dihedral angles ϕ for a) both signal sets of two half-chair conformations for (2*R*,6*S*)-**13** and b) for the chair conformations of (2*R*,6*R*)-**13**.

Analysing the ^1^H NMR spectra of reduction product (2*R*,6*S*)-**13** reveals a very similar pattern to the previously discussed Suzuki–Miyaura product (2*R*,6*S*)-**9** with one significant distinction. The signals of both protons H^2^ and H^6^ of the first conformer (2*R*,6*S*)-**13_I_** in comparison to the second one (2*R*,6*S*)-**13_II_** are split into doublets instead of double-doublets (Table 2). Therefore, the chair conformation is not a viable option as evident by the lack of a second coupling constant. Additionally, the boat configuration is not possible with torsion angles of approximately ϕ(H^2^,H^3,pro-^*^S^*) = ϕ(H^6^,H^5,pro-^*^S^*) = 0° as well as ϕ(H^2^,H^3,pro-^*^R^*) = 120° and ϕ(H^6^,H^5,pro-^*^R^*) = 240°. These angles would lead to double-doublets with coupling constants ^3^*J*(H^2^,H^3,pro-^*^S^*) = ^3^*J*(H^6^,H^5,pro-^*^S^*) in the range of 8–11 Hz and ^3^*J*(H^2^,H^3,pro-^*^R^*) = ^3^*J*(H^6^,H^5,pro-^*^R^*) in the range of 3–5 Hz. Ultimately, this indicates that for both isomers a half-chair configuration with one dihedral angle ϕ near 90° and one coupling constant at 0 Hz is preferred. The previously described more equatorial arrangement for the methyl ester in the half-chair conformer (2*R*,6*S*)-**9** can also be observed for half-chair conformer (2*R*,6*S*)-**13_II_**. Flipping further into the other half-chair conformer (2*R*,6*S*)-**13_II_** reveals a dihedral angle for ϕ(H^6^,H^5,pro-^*^S^*) = 270° and ϕ(H^2^,H^3,pro-^*^R^*) = 90° with a rather equatorial arrangement for the phenylacetylene residue (Figure 4a). For the second diastereomer (2*R*,6*R*)-**13** all signals are comparable to (2*R*,6*R*)-**9** resulting also in a chair conformation and in an equatorial position for the phenylacetylene residue and an axial position for the methyl ester (Figure 4b). This arrangement is most likely the reason for neither (2*R*,6*R*)-**13** nor (2*R*,6*R*)-**9** displaying a second signal set. The steric hindrance by forcing both bulky residues into an axial position is no longer given by switching one in an equatorial position.

**Scheme 6:**
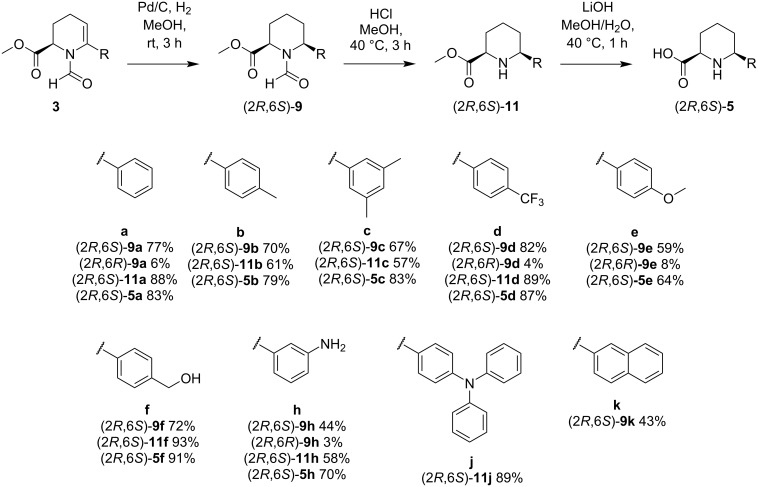
Overview of reduction and deprotection to the final pipecolic acid derivatives (2*R*,6*S*)**-5**.

Using Pd/C and H_2_ for hydrogenation reactions usually leads to reduction of double or triple bonds, while aromatic systems tend not to be affected [58]. However, **3m** and **3p** undergo hydrogenation in the aromatic moiety, while **3p** displays a complete reduction of the pyrimidine ring, only a partial reduction occurs for the anthracene ring. For all other hydrogenation products **9** the coupled aromatic moiety is not affected (Scheme 6). Removal of both protecting groups is the last step in the generation of pipecolic acid derivatives (2*R*,6*S*)**-5** and (2*R*,6*S*)**-6**. The formyl group can be cleaved under acidic conditions to yield the esters (2*R*,6*S*)**-11**. Treatment of the solution with LiOH at pH 9–10 and at 40 °C leads to saponification of the methyl ester (Scheme 5c and Scheme 6).

## Conclusion

In conclusion, we present a straightforward and efficient method for the generation of novel pipecolic acid derivatives with aryl and alkynyl modifications in the C^6^ position, employing Suzuki–Miyaura and Sonogashira–Hagihara cross-coupling reactions. Through choosing a *N*-acyliminium reduction with NaBH_3_CN approach in a homogeneous solution both diastereomers (2*R*,6*S*)-**9** and (2*R*,6*R*)**-9** are generated in a 1:1 ratio, while a hydrogenation of the *N*-formyl enamine with Pd/C and H_2_ favours the (2*R,6S*)**-9** diastereomer. Moreover, an in-depth NMR analysis, focusing on coupling constants and subsequent dihedral angles of diastereomers (2*R*,6*S*)**-9** and (2*R*,6*R*)**-9**, as well as selected deprotection products, provides an interpretation of the NMR-spectra of (2*R*,6*S*)**-9** in regard to conformation. This also offers insight into how specific constraints lead to certain conformations in six-membered rings, such as half-chair conformations. Noteworthy, the application of ᴅ-aminoadipic acid as an abundant chiral pool building block provides an entry into ᴅ-pipecolic acid derivatives. However, the synthetic strategy is of course applicable to ʟ-aminoadipic acid as well.

## Supporting Information

File 1 Experimental procedures, characterization data and NMR spectra.

## Data Availability

All data that supports the findings of this study is available in the published article and/or the supporting information of this article.
