# Supplementary material for: A versatile route towards 6-arylpipecolic acids
Source: Beilstein J Org Chem. 2025 Jun 4;21:1104–15. doi: 10.3762/bjoc.21.88 (PMC12152316; doi:10.3762/bjoc.21.88)
Supplement: File 1 — Experimental procedures, characterization data and NMR spectra. [file Beilstein_J_Org_Chem-21-1104-s001.pdf]

BEILSTEIN JOURNAL OF ORGANIC CHEMISTRY

Supporting Information

for

A versatile route towards 6-arylpipecolic acids

Erich Gebel, Cornelia Göcke, Carolin Gruner and Norbert Sewald

Beilstein J. Org. Chem. **2025,** *21,* 1104–1115. doi:10.3762/bjoc.21.88

Experimental procedures, characterization data and NMR spectra

License and Terms: This is a supporting information file under the terms of the Creative Commons Attribution License (https://creativecommons.org/licenses/by/4.0). Please note that the reuse, redistribution and reproduction in particular requires that the author(s) and source are credited and that individual graphics may be subject to special legal provisions.
The license is subject to the *Beilstein Journal of Organic Chemistry* terms and conditions: (https://www.beilstein-journals.org/bjoc/terms)

Table of contents

Chemicals and instruments S2
Condition screening S4
Conformational overview S5
Half-chair and twist-boat conformation of cyclohexane S6
General synthetic procedures S7
Synthetic procedures and characterization S9
Assigned NMR spectra S36
References S105

Chemicals and instruments

Commercially obtained reagents were purchased from Alfa Aesar, BLDpharm, Fisher Scientific, Fluorochem, Sigma-Aldrich, Thermo Fisher Scientific or VWR and were used as received without further purification unless otherwise stated. DCM was dried over CaH_2, EtOAc and cHex were distilled before use. Column chromatography was carried out using silica gel 60, particle size 0.040–0.063 mm.

Nuclear magnetic resonance spectroscopy

NMR spectroscopy was done on a Bruker Avance III 500 HD (500 MHz for ^{1}H, 126 MHz for ^{13}C{^{1}H}, 471 MHz for ^{19}F{^{13}C}) or Bruker Avance NEO 600 (600 MHz for ^{1}H, 150 MHz for ^{13}C{^{1}H}, 565 MHz for ^{19}F{^{13}C}). The chemical shift δ is reported in ppm relative to the proton signal of the solvent: $CDCl_3$: $\delta = 7.26$ ppm (^{1}H), $\delta = 77.16$ ppm (^{13}C{^{1}H}); DMSO-d_6: $\delta = 2.50$ ppm (^{1}H), $\delta = 39.52$ ppm (^{13}C{^{1}H}); D_2O: $\delta = 4.79$ ppm (^{1}H); MeOD: $\delta = 3.31$ ppm (^{1}H) and DMF-d_7: $\delta = 8.03$ ppm (^{1}H). 2D methods (^{1}H,^{1}H-COSY, ^{1}H,^{13}C-HMQC, ^{1}H,^{13}C-HMBC) were used to support and confirm the assignment.

Liquid chromatography mass spectrometry

LC–MS analysis was performed on an Agilent 6220 time-of-flight mass spectrometer (Agilent Technologies) in extended dynamic range mode equipped with a Dual-ESI source, operating with a spray voltage of 2.5 kV. Nitrogen served both as nebulizer gas and dry gas. Nitrogen was generated by a nitrogen generator NGM 11. Samples were dissolved in ACN/water 1:1 and introduced with a 1200 HPLC system consisting of an autosampler, degasser, binary pump, column oven (40 °C) and diode array detector (Agilent Technologies, at 200 nm, 220 nm 254 nm and 280 nm) using a C18 Hypersil Gold column (length: 50 mm, diameter: 2.1 mm, particle size: 1.9 µm).

The mass axis was externally calibrated with ESI-L Low Concentration Tuning Mix (Agilent Technologies) as calibration standard. The mass spectra are recorded in both profile and centroid mode with the MassHunter Workstation Acquisition B.04.00 software (Agilent Technologies). MassHunter Qualitative Analysis B.07.00 software (Agilent Technologies) was used for processing and averaging of several single spectra.

Analytical run protocol:

Time / min	Eluent A[a] / %	Eluent B[b] / %	Flow rate / $mL \cdot min^{-1}$
0.0	98	2	0.3
10.0	2	98	0.3
11.0	2	98	0.3
11.5	98	2	0.3
15.0	98	2	0.3

[a] Eluent A: water/acetonitrile/formic acid, 94.9:5:0.1, v/v/v
[b] Eluent B: acetonitrile/water/formic acid, 94.9:5:0.1, v/v/v

High resolution mass spectrometry

Accurate Mass nano-ESI measurements are performed using a Q-IMS-TOF mass spectrometer Synapt G2Si (Waters GmbH) in resolution mode, interfaced to a nano-ESI ion source. Nitrogen serves both as the nebulizer gas and the dry gas for nano-ESI. Nitrogen is generated by a nitrogen generator NGM 11. Samples were dissolved in CAN or ACN/water mixtures (if necessary acidified using formic acid) and introduced by static nano-ESI using in-house pulled glass emitters.

The mass axis was externally calibrated with ESI-L Low Concentration Tuning Mix (Agilent Technologies) as calibration standard and internally calibrated with the protonated LeuEnk ion as internal calibration standard. Scan accumulation and data processing was performed with MassLynx 4.1 (Waters GmbH) on a PC Workstation. The spectra shown here were generated by the accumulation and averaging of several single spectra. Determination of exact masses were performed using centroided data.

Reverse-phase high-pressure liquid chromatography

Purification by preparative RP-HPLC was done on a Shimadzu Nexera series consisting of a system controller (SCL-40), UV–vis detector (SPD-40V, at 200 nm, 220 nm 254 nm and 280 nm) and a degassing unit (DGU-405). As column a Shimadzu Phenomenex Luna C18 (length: 250.0 mm, diameter: 21.2 mm, particle size: 5.0 µm) was used.

Preparative run protocol:

Time / min	Eluent A[a] / %	Eluent B[b] / %	Flow rate / mL·min^{-1}
0.0	100	0	10.0
5.0	100	0	10.0
65.0	0	100	10.0
75.0	0	100	10.0

[a] Eluent A: water/acetonitrile/TFA, 94.9:5:0.1, v/v/v

[b] Eluent B: acetonitrile/water/TFA, 94.9:5:0.1, v/v/v

Conditions screening

Table S1: Detailed screening conditions for the Suzuki–Miyaura cross-coupling between bromide **2** and phenylboronic acid (**8a**) in regard to catalyst, base, solvent and temperature.

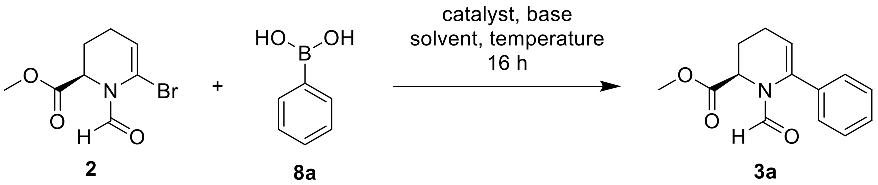

Catalyst[a]	Base[b]	Solvent	Temperature	Conversion
$PdCl_2(PPh_3)_2$	Et_3N	DMF	rt	72%
$PdCl_2(PPh_3)_2$	Na_2HPO_4	DMF	rt	65%
$PdCl_2(PPh_3)_2$	Na_2HPO_4	H_2O	40 °C	21%[c]
$PdCl_2(PPh_3)_2$	Na_2HPO_4	H_2O	60 °C	12%[c]
$PdCl_2(PPh_3)_2$	Na_2HPO_4	Phosphate buffer[d]	40 °C	39%
$PdCl_2(PPh_3)_2$	Na_2HPO_4	Phosphate buffer[d]	60 °C	32%[c]
$PdCl_2(PPh_3)_2$	Na_2HPO_4	Phosphate buffer[d]	80 °C	24%[c]
$PdCl_2(PPh_3)_2$	Cs_2CO_3	DMF	rt	88%
$PdCl_2(PPh_3)_2$	K_2CO_3	DMF	rt	81%
$PdCl_2(PPh_3)_2$	$NaHCO_3$	DMF	rt	70%
Pd Nanoparticles[e]	Na_2HPO_4	H_2O	40 °C	17%[c]
Pd Nanoparticles[e]	Na_2HPO_4	H_2O	60 °C	9%[c]
Pd Nanoparticles[e]	Na_2HPO_4	Phosphate buffer[d]	40 °C	80%
Pd Nanoparticles[e]	Na_2HPO_4	Phosphate buffer[d]	60 °C	71%[c]
Pd Nanoparticles[e]	Na_2HPO_4	Phosphate buffer[d]	80 °C	55%[c]
$Pd(PPh_3)_4$	Et_3N	DMF	rt	61%
$Pd(PPh_3)_4$	Cs_2CO_3	DMF	rt	75%
$Pd(PPh_3)_4$	K_2CO_3	DMF	rt	76%
$Pd(PPh_3)_4$	$NaHCO_3$	DMF	rt	63%
$Pd(OAc)_2$	Et_3N	DMF	rt	-
$Pd(OAc)_2$	Cs_2CO_3	DMF	rt	15%
$Pd(OAc)_2$	K_2CO_3	DMF	rt	13%
$Pd(OAc)_2$	$NaHCO_3$	DMF	rt	7%
$Pd(dppf)Cl_2$	Et_3N	DMF	rt	67%
$Pd(dppf)Cl_2$	Cs_2CO_3	DMF	rt	99%
$Pd(dppf)Cl_2$	K_2CO_3	DMF	rt	99%
$Pd(dppf)Cl_2$	$NaHCO_3$	DMF	rt	82%
XPhos Pd G2	K_2CO_3	DMF	rt	80%
XPhos Pd G2	K_2CO_3	H_2O	rt	71%
XPhos Pd G2	K_2CO_3	H_2O	40 °C	66%

[a]10 mol % of catalyst was used; [b]2.0 equiv of base was used except for Et_3N with 4.0 equiv; [c]observation of saponification of the methyl ester; [d]pH = 7.0 (0.5 M); [e]Pd Nanoparticles were produced by dissolving and stirring $PdCl_2$ in iPrOH for 16 d at rt and used in 100 mM batches.

Conformational overview

Table S2: Overview of all relevant compounds with their chemical shifts δ, multiplicity, coupling constants J and dihedral angles ϕ for both protons H^2 and H^6 as well as the resulting conformation for the compound.

Product	Class<br>δ (H^2)<br>/ ppm	$^3J(H^2,H^{3,pro\text{-}S})$<br>$^3J(H^2,H^{3,pro\text{-}R})$<br>/ Hz	$\phi(H^2,H^{3,pro\text{-}S})$<br>$\phi(H^2,H^{3,pro\text{-}R})$<br>/ °	Class<br>δ (H^6)<br>/ ppm	$^3J(H^6,H^{5,pro\text{-}S})$<br>$^3J(H^6,H^{5,pro\text{-}R})$<br>/ Hz	$\phi(H^6,H^{5,pro\text{-}S})$<br>$\phi(H^6,H^{5,pro\text{-}R})$<br>/ °	Conf.
**(2*R*,6*S*)-9**	dd<br>5.03	5.7<br>3.6	300<br>60	dd<br>4.78	5.1<br>5.1	60<br>300	chair
**(2*R*,6*S*)-9**	d<br>4.17	0<br>5.8	90<br>210	d<br>5.81	5.9<br>0	30<br>270	half-chair
**(2*R*,6*R*)-9a**	dd<br>5.26	6.3<br>2.2	300<br>60	dd<br>4.71	11.7<br>3.3	180<br>60	chair
**(2*R*,6*S*)-9c**	dd<br>4.99	4.7<br>4.7	300<br>60	dd<br>4.67	5.2<br>5.2	60<br>300	chair
**(2*R*,6*S*)-9c**	d<br>4.15	0<br>5.8	90<br>210	d<br>5.71	5.7<br>0	30<br>270	half-chair
**(2*R*,6*S*)-9d**	dd<br>5.04	4.7<br>4.7	300<br>60	dd<br>4.85	5.1<br>5.1	60<br>300	chair
**(2*R*,6*S*)-9d**	d<br>4.22	0<br>5.9	90<br>210	d<br>5.81	5.8<br>0	30<br>270	half-chair
**(2*R*,6*R*)-9d**	dd<br>5.28	5.9<br>2.1	300<br>60	dd<br>4.60	11.2<br>3.3	180<br>60	chair
**(2*R*,6*S*)-9e**	dd<br>5.02	5.7<br>3.5	300<br>60	dd<br>4.74	5.0<br>5.0	60<br>300	chair
**(2*R*,6*S*)-9e**	d<br>4.14	0<br>4.7	90<br>210	d<br>5.75	5.9<br>0	30<br>270	half-chair
**(2*R*,6*R*)-9e**	dd<br>5.29	6.1<br>2.0	300<br>60	dd<br>4.55	11.5<br>3.2	180<br>60	chair
**(2*R*,6*S*)-9f**	dd<br>5.03	5.6<br>3.6	300<br>60	dd<br>4.76	5.1<br>5.1	60<br>300	chair
**(2*R*,6*S*)-9f**	d<br>4.17	0<br>5.7	90<br>210	d<br>5.78	5.9<br>0	30<br>270	half-chair
**(2*R*,6*S*)-9h**	dd<br>4.99	4.8<br>4.8	300<br>60	dd<br>4.81	5.0<br>5.0	60<br>300	chair
**(2*R*,6*S*)-9h**	d<br>4.24	0<br>5.6	90<br>210	d<br>5.68	4.1<br>0	30<br>270	half-chair
**(2*R*,6*R*)-9h**	dd<br>5.24	4.2<br>1.9	300<br>60	dd<br>4.62	11.6<br>3.2	180<br>60	chair
**(2*R*,6*S*)-9k**	dd<br>5.07	5.7<br>3.4	300<br>60	dd<br>4.95	5.1<br>5.1	60<br>300	chair
**(2*R*,6*S*)-9k**	d<br>4.21	0<br>5.7	90<br>210	d<br>5.96	5.9<br>0	30<br>270	half-chair
**(2*R*,6*S*)-13_{I}**	d<br>5.11	4.7<br>0	330<br>90	d<br>5.06	0<br>6.0	270<br>210	half-chair
**(2*R*,6*S*)-13_{II}**	d<br>4.68	0<br>5.8	90<br>210	d<br>5.39	4.8<br>0	30<br>270	half-chair
**(2*R*,6*R*)-13**	dd<br>4.97	4.2<br>4.2	300<br>60	dd<br>4.64	10.3<br>3.3	180<br>60	chair

Product	Class<br>δ (H^2)<br>/ ppm	^3J(H^2,H$^{3,\text{pro-}S}$)<br>^3J(H^2,H$^{3,\text{pro-}R}$)<br>/ Hz	ϕ(H^2,H$^{3,\text{pro-}S}$)<br>ϕ(H^2,H$^{3,\text{pro-}R}$)<br>/ °	Class<br>δ (H^6)<br>/ ppm	^3J(H^6,H$^{5,\text{pro-}S}$)<br>^3J(H^6,H$^{5,\text{pro-}R}$)<br>/ Hz	ϕ(H^6,H$^{5,\text{pro-}S}$)<br>ϕ(H^6,H$^{5,\text{pro-}R}$)<br>/ °	Conf.
**(2*R*,6*S*)-10a**	dd<br>4.87	5.3<br>5.3	300<br>60	dd<br>4.75	7.2<br>4.4	60<br>300	chair
**(2*R*,6*S*)-10a**	d<br>4.23	0<br>5.0	90<br>210	d<br>5.77	5.7<br>0	30<br>270	half-chair
**(2*R*,6*S*)-11a**	dd<br>4.03	3.3<br>12.9	60<br>180	dd<br>4.16	2.8<br>12.8	300<br>180	chair
**(2*R*,6*S*)-11c**	dd<br>4.04	2.8<br>12.8	60<br>180	dd<br>4.11	3.6<br>12.7	300<br>180	chair
**(2*R*,6*S*)-11d**	dd<br>3.51	2.7<br>11.0	60<br>180	m[a]<br>3.74	2.3<br>10.7	300<br>180	chair
**(2*R*,6*S*)-11f**	dd<br>4.04	3.4<br>11.2	60<br>180	dd<br>4.23	3.3<br>11.2	300<br>180	chair
**(2*R*,6*S*)-11j**	dd<br>4.10	3.4<br>12.9	60<br>180	dd<br>4.18	2.6<br>13.1	300<br>180	chair

[a]Calculated coupling constants due to signal overlay

Half-chair and twist-boat conformation of cyclohexane

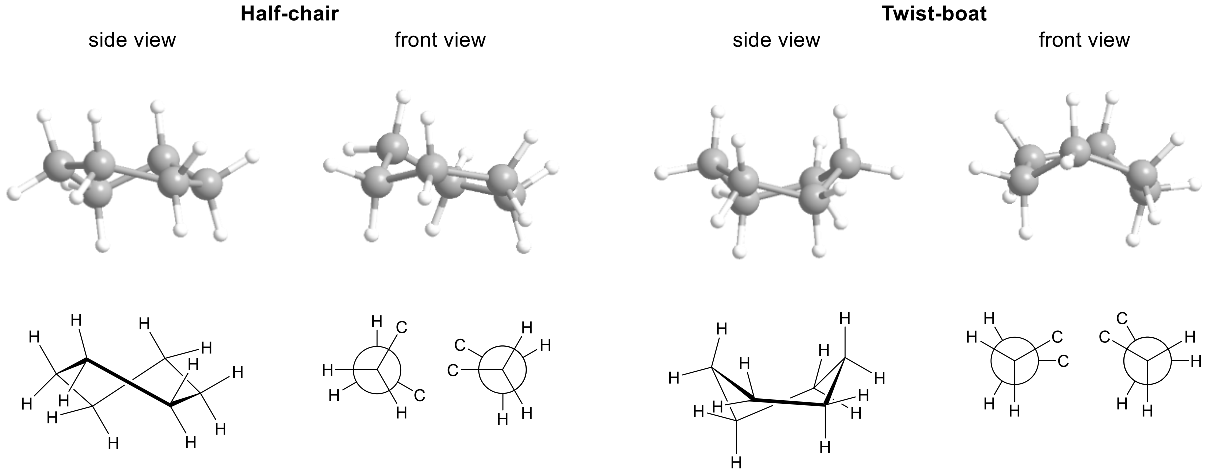

Figure S1: Side and front view of cyclohexane as ball-and-stick model [1], Lewis structure (side) and Newman projection (front) for **left:** the half-chair and **right:** the twist-boat conformation for a better visualisation.

General synthetic procedures

Suzuki–Miyaura cross-coupling reaction (**GSP 1**):

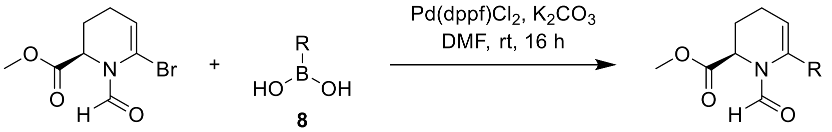

2 **8** **3**

Bromide (**2**, 1.0 equiv), K_2CO_3 (4.0 equiv) and a boronic acid (**8**, 1.5 equiv) were dissolved in DMF (5-10 mL) with an addition of water (100 µL) and degassed by freeze-pump-thaw. Pd(dppf)Cl_2 (2.5–5.0 mol %) was added in argon counterflow and the resulting mixture was stirred at room temperature for 16 h. The reaction was quenched by addition of water (5 mL) and filtered through a pad of Celite. The crude product mixture was concentrated under reduced pressure and purified by column chromatography (SiO_2, cHex/EtOAc 3:1 v/v) to obtain product **3**.

Catalytic hydrogenation reaction (**GSP 2**):

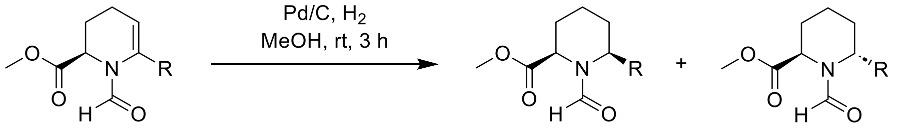

3 **(2*R*,6*S*)-9** **(2*R*,6*R*)-9**

Cross-coupled product (**3**, 1.0 equiv) was dissolved in MeOH (30–50 mL). Pd/C (10 mol %) was added and the resulting mixture was stirred at room temperature under H_2 atmosphere (1 atm, balloon). After 3 h the reaction progress was monitored every hour by LC–MS until full conversion. The suspension was filtered through a pad of Celite, washed with MeOH (10–20 mL) and concentrated under reduced pressure. Purification and separation of both diastereomers was done by PR-HPLC to obtain product **9**.

Reduction of the acyliminium intermediate (**GSP 3**):

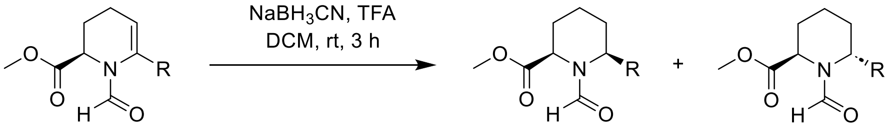

3 **(2*R*,6*S*)-9 / (2*R*,6*S*)-13** **(2*R*,6*R*)-9 / (2*R*,6*R*)-13**

×

Cross-coupled product (**3**, 1.0 equiv) and $NaBH_3CN$ (5.0 equiv) were suspended in DCM (10 mL). TFA (10.0 equiv) was added and the resulting mixture was stirred at room temperature. After 3 h the reaction progress was monitored every hour by LC–MS until full conversion. The reaction was quenched by addition of water (20 mL) and extracted with DCM (3 × 10 mL). The combined organic phases were washed with saturated Na_2CO_3 solution (20 mL) as well es brine (20 mL), dried over $MgSO_4$ and concentrated under reduced pressure. Purification and separation of both diastereomers was done by PR-HPLC to obtain product **9**.

N-formyl and methyl ester deprotection (**GSP 4**):

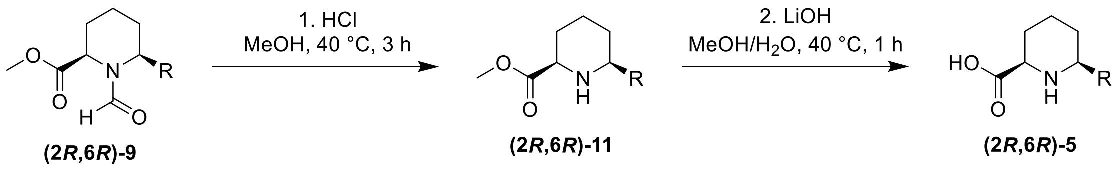

Hydrogenation product (**9**, 1.0 equiv) was dissolved in MeOH (10-20 mL). Concentrated HCl (1-2 mL) was added and the resulting mixture was stirred at 40 °C. After 3 h the reaction progress was monitored every hour by c until full conversion. If needed, product **11** can be obtained at this point by quenching with a $NaHCO_3$ solution until a neutral pH value is reached. Afterwards the mixture is extracted with DCM (3 × 10 mL) and the combined organic phases were dried over $MgSO_4$, concentrated under reduced pressure and purified by column chromatography (SiO_2, cHex/EtOAc 3:1 v/v). Otherwise, the mixture is treated with LiOH and stirred at 40 °C for 1 h. Afterwards the reaction is frozen and lyophilized. The resulting crude is suspended in DMF (5 mL) and filtered through a pad of Celite, washed with DMF (5 mL) and concentrated under reduced pressure. Purification was done by PR-HPLC to obtain product **5**.

Suzuki–Miyaura cross-coupling reaction (conditions screening)

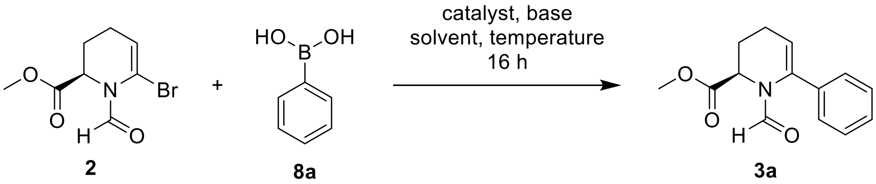

Bromide (**2**, 1.0 equiv), base (2.0–4.0 equiv) and phenylboronic acid (**8a**, 1.5 equiv) were prepared as stock solutions in DMF or water. To a mixture of the stock solutions DMF, water or phosphate buffer was added until a total volume of 200 µL was reached. The mixture was degassed by sonication, afterwards the catalyst was added and stirred for 16 h. The reaction was quenched by addition of TFA in water (100 µL) and filtered through a pad of Celite. The conversion was determined by LC–MS analysis.

Synthetic procedures and characterization

Dimethyl (*R*)-2-aminohexanedioate (**H-D-Aad(OMe)-OMe**)

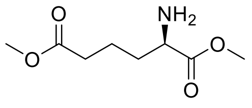

H-D-Aad(OMe)-OMe
$C_8H_{15}NO_4$
189.21 g · mol^{-1}

D-2-Aminoadipic acid (**1**, 5.51 g, 34.21 mmol, 1.0 equiv) was dissolved in MeOH (50 mL). TMS-Cl (20 mL, 16.92 g, 154.94 mmol, 4.5 equiv) was added dropwise and the mixture was stirred for 16 h at room temperature. After 16 h the reaction was quenched by addition of water (50 mL) and washed with DCM (50 mL). The aqueous phase was treated with a $NaHCO_3$ solution until basified (pH ≈ 9) and extracted with DCM (3 × 50 mL). The combined organic phases were dried over $MgSO_4$ and concentrated under reduced pressure. **H-D-Aad(OMe)-OMe** was obtained as a slight yellow oil and used without further purification [2].

R_f (DCM/MeOH, 9:1, v/v) = 0.36

LC–MS: *m*/*z* = found 190.1097 $[M+H]^+$, calculated 190.1074 $[M+H]^+$.

^{1}H NMR (500 MHz, $CDCl_3$): δ = 3.72 (s, 3H, -CH-COO-CH_3), 3.67 (s, 3H, -CH_2-COO-CH_3), 3.45 (dd, ^3J = 7.9 Hz, ^3J = 4.4 Hz, 1H, -CH-), 2.43 – 2.30 (m, 2H, -CH_2-COO-), 1.83 – 1.65 (m, 3H, -CH_2-CH_2-CH-), 1.58 (m, 1H, -CH_2-CH_2-CH-).

^{13}C{^{1}H} NMR (126 MHz, $CDCl_3$): δ = 176.45 (-CH-COO-CH_3), 173.80 (-CH_2-COO-CH_3), 54.32 (-CH-), 52.16 (-CH-COO-CH_3), 51.72 (-CH_2-COO-CH_3), 34.35 (-CH_2-CH-), 33.80 (-CH_2-COO-), 21.31 (-CH_2-CH_2-CH-).

(*R*)-methyl 6-oxopipecolate (**7**)

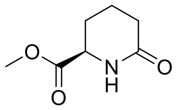

7
$C_7H_{11}NO_3$
157.17 g · mol^{-1}

H-D-Aad(OMe)-OMe was used without further purification and stirred for 3 h at 120 °C (without solvent) in a slight argon counterflow. After cooling to room temperature, the crude was purified by column chromatography (SiO_2, DCM/MeOH 9:1 v/v). Product (**7**, 4.73 g, 30.10 mmol, 88% over two steps) was obtained as a slight yellow oil [3].

R_f (DCM/MeOH, 9:1, v/v) = 0.58

LC–MS: *m*/*z* = found 158.0844 $[M+H]^+$, calculated 158.0812 $[M+H]^+$.

^{1}H NMR (500 MHz, $CDCl_3$): δ = 6.21 (s, 1H, -NH-), 4.09 (t, ^3J = 6.5 Hz, ^3J = 6.5 Hz, 1H, -CH-), 3.78 (s, 3H, -CH_3), 2.46 – 2.29 (m, 2H, -CH_2-CH-), 2.20 (m, 1H, -CH_2-CO-), 1.95 – 1.72 (m, 3H, -CH_2-CH_2-CO-).

^{13}C{^{1}H} NMR (126 MHz, $CDCl_3$): δ = 171.68 (-COO-), 171.39 (-CON-), 54.87 (-CH-), 52.82 (-CH_3), 31.17 (-CH_2-CH-), 25.51 (-CH_2-CO-), 19.64 (-CH_2-CH_2-CO-).

Methyl (*R*)-6-bromo-1-formyl-1,2,3,4-tetrahydropyridine-2-carboxylate (**2**)

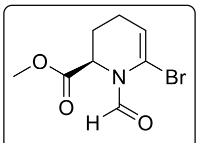

2

$C_8H_{10}BrNO_3$

248.08 g · mol^{-1}

To a stirred solution of DMF (2.7 mL, 2.55 g, 35.00 mmol, 5.0 equiv) in DCM (10 mL) a solution of $POBr_3$ (3.00 g, 10.50 mmol, 1.5 equiv) in DCM (10 mL) was added dropwise at room temperature. The resulting suspension was stirred and heated for 15 min to reflux and cooled to room temperature. Dissolved lactam (**7**, 1.05 g, 7.00 mmol, 1.0 equiv) in DCM (10 mL) was added to the suspension and stirred for 1 h to reflux. After cooling to room temperature, the reaction was quenched by addition of water (15 mL) and then extracted with water (3 × 15 mL). The combined aqueous phases were treated with a $NaHCO_3$ solution until basified (pH ≈ 9) and extracted with DCM (3 × 15 mL). The combined organic phases were dried over $MgSO_4$ and concentrated under reduced pressure. Product (**2**, 1.48 g, 5.95 mmol, 85%) was obtained as a slight yellow solid and used without further purification. If needed, product **2** can be purified by column chromatography (SiO_2, cHex/EtOAc 3:1 v/v) [4].

R_f (cHex/EtOAc 3:1 v/v) = 0.77

LC–MS: *m*/*z* = found 220.0036 [(M(Br^{79})−For)+H]$^+$ and 222.0034 [(M(Br^{81})−For)+H]$^+$, calculated 219.9968 [(M(Br^{79})−For)+H]$^+$ and 221.9948 [(M(Br^{81})−For)+H]$^+$.

^{1}H NMR (500 MHz, $CDCl_3$): δ = 9.01 (s, 1H, -CHO), 5.43 (t, ^3J = 4.2 Hz, ^3J = 4.2 Hz, 1H, -CH-COO-), 5.37 (dd, ^3J = 5.1, ^3J = 2.8, 1H, -CH=C-), 3.75 (s, 3H, -CH_3), 2.45 (m, 1H, -CH_2-CH-COO-), 2.22 – 2.10 (m, 2H, -CH_2-CH=C-), 1.89 (m, 1H, -CH_2-CH-COO-).

^{1}H NMR (600 MHz, DMSO-*d*$_6$): δ = 8.91 (s, 1H, -CHO), 5.49 (dd, ^3J = 5.6 Hz, ^3J = 2.9 Hz, 1H, -CH-COO-), 5.24 (dd, ^3J = 5.4, ^3J = 2.9, 1H, -CH=C-), 3.68 (s, 3H, -CH_3), 2.27 (m, 1H, -CH_2-CH-COO-), 2.15 (m, 1H, -CH_2-CH=C-), 2.02 (m, 1H, -CH_2-CH=C-), 1.88 (m, 1H, -CH_2-CH-COO-).

^{1}H NMR (600 MHz, MeOD): δ = 8.99 (s, 1H, -CHO), 5.51 (dd, ^3J = 6.1 Hz, ^3J = 2.9 Hz, 1H, -CH-COO-), 5.32 (m, 1H, -CH=C-), 3.74 (s, 3H, -CH_3), 2.42 (m, 1H, -CH_2-CH-COO-), 2.19 (m, 1H, -CH_2-CH=C-), 2.12 (m, 1H, -CH_2-CH=C-), 1.94 (m, 1H, -CH_2-CH-COO-).

^{1}H NMR (600 MHz, DMF-*d*$_7$): δ = 9.00 (s, 1H, -CHO), 5.52 (dd, ^3J = 5.7 Hz, ^3J = 2.9 Hz, 1H, -CH-COO-), 5.31 (m, 1H, -CH=C-), 3.75 (s, 3H, -CH_3), 2.36 (m, 1H, -CH_2-CH-COO-), 2.21 (m, 1H, -CH_2-CH=C-), 2.10 (m, 1H, -CH_2-CH=C-), 1.97 (m, 1H, -CH_2-CH-COO-).

Methyl (*R*)-1-formyl-6-(phenylethynyl)-1,2,3,4-tetrahydropyridine-2-carboxylate (**4**)

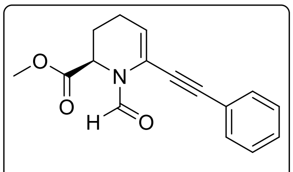

4
$C_{16}H_{15}NO_3$
269.30 g · mol^{-1}

Bromide (**2**, 0.25 g, 1.00 mmol, 1.0 equiv), K_2CO_3 (0.55 g, 4.00 mmol, 4.0 equiv) and phenylacetylene (**12**, 0.26 g, 274 µL, 2.5 equiv) were dissolved in DMF (5 mL) with an addition of water (100 µL) and degassed by freeze-pump-thaw. Pd(dppf)Cl_2 (36.6 mg, 0.05 mmol, 5 mol %) and CuI (19.1 mg, 0.10 mmol, 10 mol %) were added in argon counterflow and the resulting mixture was stirred at room temperature for 16 h. The reaction was quenched by addition of water (5 mL) and filtered through a pad of Celite. The crude product mixture was concentrated under reduced pressure and purified by column chromatography (SiO_2, cHex/EtOAc 3:1 v/v) to obtain product (**4**, 0.21 g, 0.78 mmol, 78%) as a slight brown oil [4].

R$_f$ (cHex/EtOAc 3:1 v/v) = 0.47

LC–MS: *m*/*z* = found 270.1194 $[M+H]^+$, calculated 270.1125 $[M+H]^+$.

^{1}H NMR (500 MHz, $CDCl_3$): δ = 9.19 (s, 1H, -CHO), 7.47 (dd, ^3J = 7.1 Hz, ^3J = 2.2 Hz, 2H, Ar_{meta}-H), 7.39 – 7.32 (m, 3H, $Ar_{ortho/para}$-H), 5.60 (dd, ^3J = 5.9 Hz, ^3J = 3.0 Hz, 1H, -CH-COO-), 5.28 – 5.23 (m, 1H, -CH=C-), 3.76 (s, 3H, -CH_3), 2.45 (m, 1H, -CH_2-CH-COO-), 2.29 – 2.11 (m, 2H, -CH_2-CH=C-), 1.88 (m, 1H, -CH_2-CH-COO-).

Methyl (*R*)-1-formyl-6-phenyl-1,2,3,4-tetrahydropyridine-2-carboxylate (**3a**)

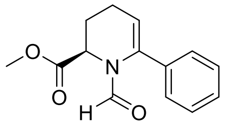

3a
$C_{14}H_{15}NO_3$
245.28 g · mol^{-1}

Bromide (**2**, 1.00 g, 4.05 mmol, 1.0 equiv), K_2CO_3 (2.24 g, 16.20 mmol, 4.0 equiv), boronic acid (**8a**, 0.77 g, 6.32 mmol, 1.5 equiv) and Pd(dppf)Cl_2 (75.0 mg, 0.10 mmol, 2.5 mol %) were combined in DMF (10 mL) according to **GSP 1**. Product (**3a**, 0.89 g, 3.65 mmol, 90%) was obtained as a slight brown oil [4].

R$_f$ (cHex/EtOAc 3:1 v/v) = 0.42

LC–MS: *m*/*z* = found 246.1201 $[M+H]^+$, calculated 246.1125 $[M+H]^+$.

^{1}H NMR (500 MHz, $CDCl_3$): δ = 8.29 (s, 1H, -CHO), 7.44 (d, ^3J = 7.4 Hz, 2H, Ar_{ortho}-H), 7.41 – 7.33 (m, 3H, $Ar_{meta/para}$-H), 5.49 (dd, ^3J = 4.0 Hz, ^3J = 4.0 Hz, 1H, -CH-COO-), 5.15 (dd, ^3J = 4.0 Hz, ^3J = 4.0 Hz, 1H, -CH=C-), 3.79 (s, 3H, -CH_3), 2.49 (m, 1H, -CH_2-CH-COO-), 2.31 – 2.20 (m, 2H, -CH_2-CH=C-), 1.98 (m, 1H, -CH_2-CH-COO-).

Methyl (*R*)-1-formyl-6-(*p*-tolyl)-1,2,3,4-tetrahydropyridine-2-carboxylate (**3b**)

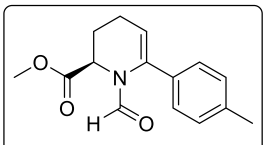

3b
$C_{15}H_{17}NO_3$
259.31 g · mol^{-1}

Bromide (**2**, 1.00 g, 4.05 mmol, 1.0 equiv), K_2CO_3 (2.24 g, 16.20 mmol, 4.0 equiv), boronic acid (**8b**, 0.86 g, 6.32 mmol, 1.5 equiv) and Pd(dppf)Cl_2 (75.0 mg, 0.10 mmol, 2.5 mol %) were combined in DMF (10 mL) according to **GSP 1**. Product (**3b**, 0.88 g, 3.40 mmol, 84%) was obtained as a slight brown oil.

R$_f$ (cHex/EtOAc 3:1 v/v) = 0.41

LC–MS: *m*/*z* = found 260.1311 [M+H]$^+$, calculated 260.1281 [M+H]$^+$.

^{1}H NMR (600 MHz, $CDCl_3$): δ = 8.30 (s, 1H, -CHO), 7.31 (d, ^3J = 8.1 Hz, 2H, Ar$_{ortho}$-H), 7.18 (d, ^3J = 7.7 Hz, 2H, Ar$_{meta}$-H), 5.48 (dd, ^3J = 5.0 Hz, ^3J = 3.1 Hz, 1H, -CH-COO-), 5.09 (dd, ^3J = 4.4 Hz, ^3J = 3.3 Hz, 1H, -CH$_2$-CH=C-), 3.78 (s, 3H, -COO-CH$_3$), 2.47 (m, 1H, -CH$_2$-CH-COO-), 3.37 (s, 3H, -C-CH$_3$), 2.29 – 2.17 (m, 2H, -CH$_2$-CH=C-), 1.96 (m, 1H, -CH$_2$-CH-COO-).

^{13}C{^{1}H} NMR (151 MHz, $CDCl_3$): δ = 171.03 (-COO-), 161.55 (-CHO), 138.71 (-C-CH$_3$), 137.47 (-CH$_2$-CH=C-), 133.60 (-CH$_2$-CH=C-C-), 129.60 (Ar$_{meta}$-C), 128.08 (Ar$_{ortho}$-C), 111.46 (-CH$_2$-CH=C-), 52.71 (-COO-CH$_3$), 50.40 (-CH-COO-), 23.83 (-CH$_2$-CH-COO-), 21.37 (-C-CH$_3$), 20.54 (-CH$_2$-CH=C-).

Methyl (*R*)-6-(3,5-dimethylphenyl)-1-formyl-1,2,3,4-tetrahydropyridine-2-carboxylate (**3c**)

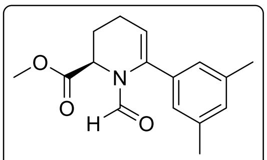

3c
$C_{16}H_{19}NO_3$
273.33 g · mol^{-1}

Bromide (**2**, 1.00 g, 4.05 mmol, 1.0 equiv), K_2CO_3 (2.24 g, 16.20 mmol, 4.0 equiv), boronic acid (**8c**, 0.95 g, 6.32 mmol, 1.5 equiv) and Pd(dppf)Cl_2 (75.0 mg, 0.10 mmol, 2.5 mol %) were combined in DMF (10 mL) according to **GSP 1**. Product (**3c**, 0.86 g, 3.16 mmol, 78%) was obtained as a slight brown oil.

R$_f$ (cHex/EtOAc 3:1 v/v) = 0.45

LC–MS: *m*/*z* = found 274.1482 [M+H]$^+$, calculated 274.1438 [M+H]$^+$.

^{1}H NMR (600 MHz, $CDCl_3$): δ = 8.30 (s, 1H, -CHO), 7.04 (s, 2H, Ar$_{ortho}$-H), 6.98 (s, 1H, Ar$_{para}$-H), 5.47 (dd, ^3J = 5.0 Hz, ^3J = 3.1 Hz, 1H, -CH-COO-), 5.10 (td, ^3J = 3.4 Hz, ^3J = 3.4 Hz, ^4J = 1.6 Hz, 1H, -CH$_2$-CH=C-), 3.78 (s, 3H, -COO-CH$_3$), 2.47 (m, 1H, -CH$_2$-CH-COO-), 3.32 (s, 6H, -C-CH$_3$), 2.25 – 2.19 (m, 2H, -CH$_2$-CH=C-), 1.96 (m, 1H, -CH$_2$-CH-COO-).

^{13}C{^{1}H} NMR (151 MHz, $CDCl_3$): δ = 170.94 (-COO-), 161.84 (-CHO), 138.53 (-C-CH$_3$), 137.67 (-CH$_2$-CH=C-), 136.28 (-CH$_2$-CH=C-C-), 130.42 (Ar$_{para}$-C), 126.00 (Ar$_{ortho}$-C), 111.43 (-CH$_2$-CH=C-), 52.71 (-COO-CH$_3$), 50.47 (-CH-COO-), 23.81 (-CH$_2$-CH-COO-), 21.41 (-C-CH$_3$), 20.51 (-CH$_2$-CH=C-).

Methyl(*R*)-1-formyl-6-(4-(trifluoromethyl)phenyl)-1,2,3,4-tetrahydropyridine-2-carboxylate (**3d**)

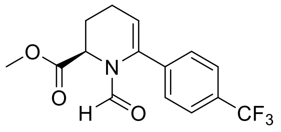

3d
$C_{15}H_{14}F_3NO_3$
313.28 g · mol^{-1}

Bromide (**2**, 1.00 g, 4.05 mmol, 1.0 equiv), K_2CO_3 (2.24 g, 16.20 mmol, 4.0 equiv), boronic acid (**8d**, 1.20 g, 6.32 mmol, 1.5 equiv) and Pd(dppf)Cl_2 (75.0 mg, 0.10 mmol, 2.5 mol %) were combined in DMF (10 mL) according to **GSP 1**. Product (**3d**, 1.13 g, 3.60 mmol, 89%) was obtained as a slight brown oil.

$\mathbf{R_f}$ (cHex/EtOAc 3:1 v/v) = 0.46

HRMS: *m*/*z* = found 336.0813 [M+Na]$^+$, calculated 336.08180 [M+Na]$^+$.

^{1}H NMR (600 MHz, $CDCl_3$): δ = 8.23 (s, 1H, -CHO), 7.66 (d, ^3J = 8.0 Hz, 2H, Ar$_{meta}$-H), 7.58 (d, ^3J = 8.1 Hz, 2H, Ar$_{ortho}$-H), 5.51 (dd, ^3J = 5.0 Hz, ^3J = 3.1 Hz, 1H, -CH-COO-), 5.24 (t, ^3J = 3.7 Hz, ^3J = 3.7 Hz, 1H, -CH$_2$-CH=C-), 3.79 (s, 3H, -CH$_3$), 2.50 (m, 1H, -CH$_2$-CH-COO-), 2.31 – 2.25 (m, 2H, -CH$_2$-CH=C-), 2.00 (m, 1H, -CH$_2$-CH-COO-).

^{13}C{^{1}H} NMR (151 MHz, $CDCl_3$): δ = 170.70 (-COO-), 161.33 (-CHO), 140.06 (-CH$_2$-CH=C-C-), 136.40 (-CH$_2$-CH=C-), 130.94 (q, ^2J = 32.8 Hz, -C-CF$_3$), 128.44 (Ar$_{ortho}$-C), 126.06 (q, ^3J = 3.7 Hz, Ar$_{meta}$-C), 124.01 (q, ^1J = 271.0 Hz, -CF$_3$), 114.40 (-CH$_2$-CH=C-), 52.87 (-CH$_3$), 50.52 (-CH-COO-), 23.78 (-CH$_2$-CH-COO-), 20.73 (-CH$_2$-CH=C-).

^{19}F{^{13}C} NMR (565 MHz, $CDCl_3$): δ = −62.73 (-CF$_3$).

Methyl (*R*)-1-formyl-6-(4-methoxyphenyl)-1,2,3,4-tetrahydropyridine-2-carboxylate (**3e**)

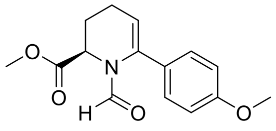

3e
$C_{15}H_{17}NO_4$
275.30 g · mol^{-1}

Bromide (**2**, 0.74 g, 3.00 mmol, 1.0 equiv), K_2CO_3 (1.66 g, 12.00 mmol, 4.0 equiv), boronic acid (**8e**, 0.68 g, 4.50 mmol, 1.5 equiv) and Pd(dppf)Cl_2 (55.0 mg, 0.08 mmol, 2.5 mol %) were combined in DMF (10 mL) according to **GSP 1**. Product (**3e**, 0.59 g, 2.13 mmol, 71%) was obtained as a slight brown oil.

$\mathbf{R_f}$ (cHex/EtOAc 3:1 v/v) = 0.42

LC–MS: *m*/*z* = found 276.1291 [M+H]$^+$, calculated 276.1230 [M+H]$^+$.

^{1}H NMR (500 MHz, $CDCl_3$): δ = 8.30 (s, 1H, -CHO), 7.35 (d, ^3J = 8.7 Hz, 2H, Ar$_{ortho}$-H), 6.90 (d, ^3J = 8.9 Hz, 2H, Ar$_{meta}$-H), 5.47 (dd, ^3J = 5.0 Hz, ^3J = 3.1 Hz, 1H, -CH-COO-), 5.05 (dd, ^3J = 3.9 Hz, ^3J = 3.9 Hz, 1H, -CH$_2$-CH=C-), 3.82 (s, 3H, -CO-CH$_3$), 3.77 (s, 3H, -COO-CH$_3$), 2.47 (m, 1H, -CH$_2$-CH-COO-), 2.30 – 2.14 (m, 2H, -CH$_2$-CH=C-), 1.95 (m, 1H, -CH$_2$-CH-COO-).

^{13}C{^{1}H} NMR (126 MHz, $CDCl_3$): δ = 171.04 (-COO-), 161.59 (-CHO), 160.01 (-CO-CH$_3$), 137.16 (-CH$_2$-CH=C-), 129.47 (Ar$_{ortho}$-C), 128.87 (-CH$_2$-CH=C-C-), 114.33 (Ar$_{meta}$-C), 111.09 (-CH$_2$-CH=C-), 55.49 (-CO-CH$_3$), 52.72 (-COO-CH$_3$), 50.46 (-CH-COO-), 23.87 (-CH$_2$-CH-COO-), 20.53 (-CH$_2$-CH=C-).

Methyl (*R*)-1-formyl-6-(4-(hydroxymethyl)phenyl)-1,2,3,4-tetrahydropyridine-2-carboxylate (**3f**)

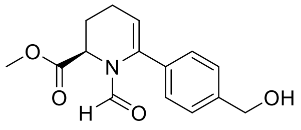

3f
$C_{15}H_{17}NO_4$
275.30 g · mol^{-1}

Bromide (**2**, 0.74 g, 3.00 mmol, 1.0 equiv), K_2CO_3 (1.66 g, 12.00 mmol, 4.0 equiv), boronic acid (**8f**, 0.68 g, 4.50 mmol, 1.5 equiv) and Pd(dppf)Cl_2 (55.0 mg, 0.08 mmol, 2.5 mol %) were combined in DMF (10 mL) according to **GSP 1**. Product (**3f**, 0.64 g, 2.34 mmol, 78%) was obtained as a slight brown oil.

R$_f$ (cHex/EtOAc 3:1 v/v) = 0.40

HRMS: *m*/*z* = found 276.1236 $[M+H]^+$, calculated 276.12304 $[M+H]^+$.

^{1}H NMR (600 MHz, $CDCl_3$): δ = 8.28 (s, 1H, -CHO), 7.43 (d, ^3J = 8.2 Hz, 2H, Ar_{ortho}-H), 7.39 (d, ^3J = 8.3 Hz, 2H, Ar_{meta}-H), 5.49 (dd, ^3J = 5.0 Hz, ^3J = 3.1 Hz, 1H, -CH-COO-), 5.14 (dd, ^3J = 4.3 Hz, ^3J = 3.3 Hz, 1H, -CH_2-CH=C-), 4.73 (s, 2H, -CH_2-OH), 3.79 (s, 3H, -CH_3), 2.49 (m, 1H, -CH_2-CH-COO-), 2.27 – 2.22 (m, 2H, -CH_2-CH=C-), 1.98 (m, 1H, -CH_2-CH-COO-).

^{13}C{^{1}H} NMR (151 MHz, $CDCl_3$): δ = 170.92 (-COO-), 161.60 (-CHO), 141.52 (-C-CH_2-OH), 137.22 (-CH_2-CH=C-C-), 135.82 (-CH_2-CH=C-), 127.51 (Ar_{ortho}-C), 128.40 (Ar_{meta}-C), 112.36 (-CH_2-CH=C-), 65.02 (CH_2-OH), 52.79 (-CH_3), 50.49 (-CH-COO-), 23.82 (-CH_2-CH-COO-), 20.60 (-CH_2-CH=C-).

Methyl (*R*)-6-(4-aminophenyl)-1-formyl-1,2,3,4-tetrahydropyridine-2-carboxylate (**3g**)

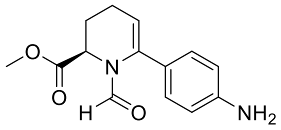

3g
$C_{14}H_{16}N_2O_3$
260.29 g · mol^{-1}

Bromide (**2**, 0.37 g, 1.50 mmol, 1.0 equiv), K_2CO_3 (0.83 g, 6.00 mmol, 4.0 equiv), boronic acid (**8g**, 0.35 g, 2.25 mmol, 1.5 equiv) and Pd(dppf)Cl_2 (27.5 mg, 0.04 mmol, 2.5 mol %) were combined in DMF (10 mL) according to **GSP 1**. Product (**3g**, 0.21 g, 0.83 mmol, 55%) was obtained as a slight brown oil.

R$_f$ (cHex/EtOAc 3:1 v/v) = 0.35

LC–MS: *m*/*z* = found 261.177 $[M+H]^+$, calculated 261.1234 $[M+H]^+$.

^{1}H NMR (500 MHz, $CDCl_3$): δ = 8.35 (s, 1H, -CHO), 7.21 (d, ^3J = 8.2 Hz, 2H, Ar_{meta}-H), 6.67 (d, ^3J = 8.2 Hz, 2H, Ar_{ortho}-H), 5.46 (dd, ^3J = 3.9 Hz, ^3J = 3.9 Hz, 1H, -CH-COO-), 5.02 (t, ^3J = 4.1 Hz, ^3J = 4.1 Hz, 1H, -CH_2-CH=C-), 3.77 (s, 3H, -CH_3), 2.45 (m, 1H, -CH_2-CH-COO-), 2.30 – 2.10 (m, 2H, -CH_2-CH=C-), 1.94 (m, 1H, -CH_2-CH-COO-).

^{13}C{^{1}H} NMR (126 MHz, $CDCl_3$): δ = 171.13 (-COO-), 161.73 (-CHO), 146.96 (-C-NH_2), 137.53 (-CH_2-CH=C-C-), 129.35 (Ar_{meta}-C), 126.53 (-CH_2-CH=C-), 115.15 (Ar_{ortho}-C), 110.15 (-CH_2-CH=C-), 52.68 (-CH_3), 50.44 (-CH-COO-), 23.94 (-CH_2-CH-COO-), 20.51 (-CH_2-CH=C-).

Methyl (*R*)-6-(3-aminophenyl)-1-formyl-1,2,3,4-tetrahydropyridine-2-carboxylate (**3h**)

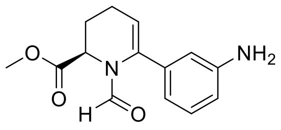

3h
$C_{14}H_{16}N_2O_3$
260.29 g·mol^{-1}

Bromide (**2**, 0.37 g, 1.50 mmol, 1.0 equiv), K_2CO_3 (0.83 g, 6.00 mmol, 4.0 equiv), boronic acid (**8h**, 0.35 g, 2.25 mmol, 1.5 equiv) and Pd(dppf)Cl_2 (27.5 mg, 0.04 mmol, 2.5 mol %) were combined in DMF (10 mL) according to **GSP 1**. Product (**3h**, 0.27 g, 1.04 mmol, 69%) was obtained as a slight brown oil.

$\mathbf{R_f}$ (cHex/EtOAc 3:1 v/v) = 0.36

HRMS: *m*/*z* = found 261.1244 [M+H]$^+$, calculated 261.12337 [M+H]$^+$.

^{1}**H NMR** (600 MHz, $CDCl_3$): δ = 8.32 (s, 1H, -CHO), 7.14 (t, ^3J = 7.8 Hz, 1H, -CH-CH-CH-), 6.80 (dd, ^3J = 7.7 Hz, ^4J = 1.5 Hz, 1H, Ar_{para}-H), 6.74 (s, 1H, -C-CH-C-), 6.64 (dd, ^3J = 8.0 Hz, ^4J = 2.5 Hz, 1H, Ar_{ortho}-H), 5.45 (dd, ^3J = 5.2 Hz, ^3J = 2.9 Hz, 1H, -CH-COO-), 5.11 (t, ^3J = 3.8 Hz, ^3J = 3.8 Hz, 1H, -CH_2-CH=C-), 3.77 (s, 3H, -CH_3), 2.46 (m, 1H, -CH_2-CH-COO-), 2.27 – 2.15 (m, 2H, -CH_2-CH=C-), 1.95 (m, 1H, -CH_2-CH-COO-).

^{13}**C{^{1}H} NMR** (151 MHz, $CDCl_3$): δ = 171.01 (-COO-), 161.73 (-CHO), 138.71 (-C-NH_2), 137.65 (-CH_2-CH=C-C-), 137.54 (-CH_2-CH=C-), 129.81 (-CH-CH-CH-), 118.38 (Ar_{para}-C), 115.37 (Ar_{ortho}-C), 114.52 (-C-CH-C-), 111.28 (-CH_2-CH=C-), 52.68 (-CH_3), 50.35 (-CH-COO-), 23.82 (-CH_2-CH-COO-), 20.48 (-CH_2-CH=C-).

Methyl (*R*)-6-(4-(diphenylamino)phenyl)-1-formyl-1,2,3,4-tetrahydropyridine-2-carboxylate (**3j**)

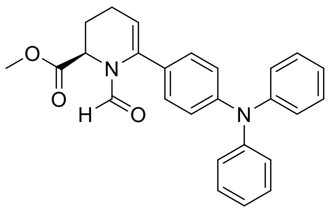

3j
$C_{26}H_{24}N_2O_3$
412.49 g·mol^{-1}

Bromide (**2**, 0.74 g, 3.00 mmol, 1.0 equiv), K_2CO_3 (1.66 g, 12.00 mmol, 4.0 equiv), boronic acid (**8j**, 1.30 g, 4.50 mmol, 1.5 equiv) and Pd(dppf)Cl_2 (55.0 mg, 0.08 mmol, 2.5 mol %) were combined in DMF (10 mL) according to **GSP 1**. Product (**3j**, 0.82 g, 1.98 mmol, 66%) was obtained as a slight brown oil.

$\mathbf{R_f}$ (cHex/EtOAc 3:1 v/v) = 0.42

LC–MS: *m*/*z* = found 413.2024 [M+H]$^+$, calculated 413.1860 [M+H]$^+$.

^{1}**H NMR** (600 MHz, $CDCl_3$): δ = 8.41 (s, 1H, -CHO), 7.30 – 7.27 (m, 4H, Ar_{meta}-H), 7.16 – 7.12 (m, 2H, Ar_{ortho}-H), 7.12 – 7.09 (m, 4H, Ar_{ortho}-H), 7.08 – 7.01 (m, 4H, Ar_{meta}-H + Ar_{para}-H), 5.48 (dd, ^3J = 5.0 Hz, ^3J = 3.1 Hz, 1H, -CH-COO-), 5.12 (t, ^3J = 4.0 Hz, ^3J = 4.0 Hz, 1H, -CH_2-CH=C-), 3.76 (s, 3H, -CH_3), 2.48 (m, 1H, -CH_2-CH-COO-), 2.28 – 2.19 (m, 2H, -CH_2-CH=C-), 1.96 (m, 1H, -CH_2-CH-COO-).

^{13}**C{^{1}H} NMR** (151 MHz, $CDCl_3$): δ = 171.03 (-COO-), 161.67 (-CHO), 148.40 (Ar_{para}-C), 147.49 (Ar_{ipso}-C), 137.26 (-CH_2-CH=C-), 129.75 (-CH_2-CH=C-C-), 129.51 (Ar_{meta}-C), 128.91 (Ar_{ortho}-C), 124.89 (Ar_{ortho}-C), 123.50 (Ar_{meta}-C), 123.13 (Ar_{para}-C), 111.24 (-CH_2-CH=C-), 52.73 (-CH_3), 50.41 (-CH-COO-), 23.91 (-CH_2-CH-COO-), 20.59 (-CH_2-CH=C-).

Methyl (*R*)-1-formyl-6-(naphthalen-2-yl)-1,2,3,4-tetrahydropyridine-2-carboxylate (**3k**)

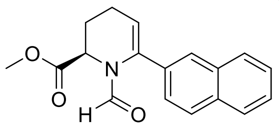

3k
$C_{18}H_{17}NO_3$
295.34 g·mol^{-1}

Bromide (**2**, 0.74 g, 3.00 mmol, 1.0 equiv), K_2CO_3 (1.66 g, 12.00 mmol, 4.0 equiv), boronic acid (**8k**, 0.77 g, 4.50 mmol, 1.5 equiv) and Pd(dppf)Cl_2 (55.0 mg, 0.08 mmol, 2.5 mol %) were combined in DMF (10 mL) according to **GSP 1**. Product (**3k**, 0.51 g, 1.74 mmol, 58%) was obtained as a slight brown oil.

R$_f$ (cHex/EtOAc 3:1 v/v) = 0.41

LC–MS: *m*/*z* = found 296.13 [M+H]$^+$, calculated 296.1281 [M+H]$^+$.

^{1}H NMR (600 MHz, $CDCl_3$): δ = 8.30 (s, 1H, -CHO), 7.90 (1H, -C-CH-C-), 7.88 – 7.82 (m, 3H, Ar-H), 7.56 (dd, ^3J = 8.5 Hz, ^4J = 1.8 Hz, 1H, Ar-H), 7.54 – 7.50 (m, 2H, Ar-H), 5.54 (dd, ^3J = 5.0 Hz, ^3J = 3.1 Hz, 1H, -CH-COO-), 5.29 (dd, ^3J = 3.8 Hz, ^3J = 1.2 Hz, 1H, -CH_2-CH=C-), 3.83 (s, 3H, -CH_3), 2.54 (m, 1H, -CH_2-CH-COO-), 2.34 – 2.25 (m, 2H, -CH_2-CH=C-), 2.04 (m, 1H, -CH_2-CH-COO-).

^{13}C{^{1}H} NMR (151 MHz, $CDCl_3$): δ = 171.87 (-COO-), 161.89 (-CHO), 137.46 (-CH_2-CH=C-), 133.68 (Ar-C-), 133.45 (-CH_2-CH=C-C-), 133.40 (Ar-C), 128.76 (Ar-CH), 128.25 (Ar-CH), 127.93 (Ar-CH), 127.58 (-C-CH-C-), 126.81 (-C-CH-C-), 126.83 (-C-CH-C-), 125.55 (-C-CH-C-), 113.12 (-CH_2-CH=C-), 52.85 (-CH_3), 50.64 (-CH-COO-), 23.86 (-CH_2-CH-COO-), 20.73 (-CH_2-CH=C-).

Methyl (*R*)-6-(anthracen-9-yl)-1-formyl-1,2,3,4-tetrahydropyridine-2-carboxylate (**3m**)

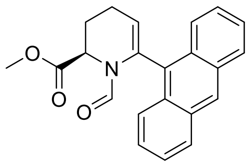

3m
$C_{22}H_{19}NO_3$
345.40 g·mol^{-1}

Bromide (**2**, 0.74 g, 3.00 mmol, 1.0 equiv), K_2CO_3 (1.66 g, 12.00 mmol, 4.0 equiv), boronic acid (**8m**, 1.00 g, 4.50 mmol, 1.5 equiv) and Pd(dppf)Cl_2 (55.0 mg, 0.08 mmol, 2.5 mol %) were combined in DMF (10 mL) according to **GSP 1**. Product (**3m**, 0.54 g, 1.74 mmol, 58%) was obtained as a slight brown oil.

R$_f$ (cHex/EtOAc 3:1 v/v) = 0.44

LC–MS: *m*/*z* = found 346.1469 [M+H]$^+$, calculated 346.1438 [M+H]$^+$.

^{1}H NMR (600 MHz, $CDCl_3$): δ = 8.52 (s, 1H, -C-CH-C-), 8.50 (dd, ^3J = 8.7 Hz, ^4J = 1.0 Hz, 1H, Ar-H), 8.09 (m, 1H, Ar-H), 8.05 – 7.99 (m, 2H, Ar-H), 7.68 (s, 1H, -CHO), 7.57 (dd, ^3J = 8.7 Hz, ^3J = 6.5 Hz, 1H, Ar-H), 7.53 – 7.46 (m, 3H, Ar-H), 5.44 (m, 1H, -CH-COO-), 5.13 (dd, ^3J = 6.0 Hz, ^3J = 1.3 Hz, 1H, -CH_2-CH=C-), 3.91 (s, 3H, -CH_3), 2.67 (m, 1H, -CH_2-CH-COO-), 2.45 – 2.34 (m, 2H, -CH_2-CH=C-), 2.29 (m, 1H, -CH_2-CH-COO-).

^{13}C{^{1}H} NMR (151 MHz, $CDCl_3$): δ = 171.03 (-COO-), 161.37 (-CHO), 132.67 (-CH_2-CH=C-C-), 131.53 (Ar-C-), 131.25 (Ar-C-), 130.87 (Ar-C-), 130.76 (Ar-C-), 128.88 (Ar-CH), 128.76 (Ar-CH), 128.53 (-C-CH-C-), 128.12 (-CH_2-CH=C-), 127.02 (Ar-CH), 126.89 (Ar-CH), 126.24 (Ar-CH), 125.89 (Ar-CH), 125.54 (Ar-CH), 125.35 (Ar-CH), 112.03 (-CH_2-CH=C-), 52.84 (-CH_3), 51.85 (-CH-COO-), 23.37 (-CH_2-CH-COO-), 20.38 (-CH_2-CH=C-).

Methyl (*R*)-6-(2-(dimethylamino)pyrimidin-5-yl)-1-formyl-1,2,3,4-tetrahydropyridine-2-carboxylate (**3p**)

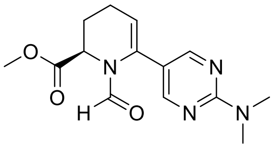

3p

$C_{14}H_{18}N_4O_3$

290.32 g · mol^{-1}

Bromide (**2**, 0.37 g, 1.50 mmol, 1.0 equiv), K_2CO_3 (0.83 g, 6.00 mmol, 4.0 equiv), boronic acid (**8p**, 0.56 g, 2.25 mmol, 1.5 equiv) and Pd(dppf)Cl_2 (27.5 mg, 0.04 mmol, 2.5 mol %) were combined in DMF (10 mL) according to **GSP 1**. Product (**3p**, 0.30 g, 1.05 mmol, 70%) was obtained as a slight brown oil.

R$_f$ (cHex/EtOAc 3:1 v/v) = 0.40

HRMS: m/z = found 291.1455 [M+H]$^+$, calculated 291.14517 [M+H]$^+$.

^{1}H NMR (600 MHz, $CDCl_3$): δ = 8.64 (s, 2H, Ar$_{ortho}$-H), 8.26 (s, 1H, -CHO), 5.49 (dd, ^3J = 5.0 Hz, ^3J = 3.0 Hz, 1H, -CH-COO-), 5.09 (dd, ^3J = 3.9 Hz, ^3J = 3.9 Hz, 1H, -CH$_2$-CH=C-), 3.79 (s, 3H, -COO-CH$_3$), 3.46 (s, 3H, -N-CH$_3$), 3.45 (s, 3H, -N-CH$_3$), 2.51 (m, 1H, -CH$_2$-CH-COO-), 2.33 – 2.23 (m, 2H, -CH$_2$-CH=C-), 2.00 (m, 1H, -CH$_2$-CH-COO-).

^{13}C{^{1}H} NMR (151 MHz, $CDCl_3$): δ = 171.35 (-COO-), 159.95 (-CHO), 154.32 (Ar$_{ortho}$-C), 152.72 (-C-N(CH$_3$)$_2$), 129.52 (-CH$_2$-CH=C-), 118.49 (-CH$_2$-CH=C-C-), 116.55 (-CH$_2$-CH=C-), 53.07 (-COO-CH$_3$), 50.63 (-CH-COO-), 39.11 (-N(CH$_3$)$_2$), 23.58 (-CH$_2$-CH-COO-), 20.78 (-CH$_2$-CH=C-).

Methyl (2*R*,6*S*/6*R*)-1-formyl-6-phenylpiperidine-2-carboxylate (**(2*R*,6*S*)-9a**/**(2*R*,6*R*)-9a**)

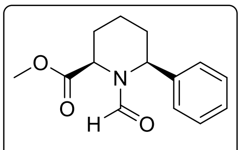

(2*R*,6*S*)-9a
$C_{14}H_{17}NO_3$
247.29 g · mol^{-1}

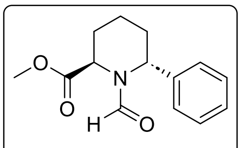

(2*R*,6*R*)-9a
$C_{14}H_{17}NO_3$
247.29 g · mol^{-1}

Cross-coupled product (**3a**, 0.89 g, 3.65 mmol, 1.0 equiv) and Pd/C (38.8 mg, 0.36 mmol, 10 mol %) were combined in MeOH (10 mL) according to **GSP 2**. Product (**(2*R*,6*S*)-9a**, 0.69 g, 2.81 mmol, 77%) and product (**(2*R*,6*R*)-9a**, 54.1 mg, 0.22 mmol, 6%) were obtained as colourless solids.

Cross-coupled product (**3a**, 0.25 g, 1.00 mmol, 1.0 equiv) and $NaBH_3CN$ (0.31 g, 5.00 mmol, 5.0 equiv) and TFA (1.14 g, 765 µL, 10.00 mmol, 10.0 equiv) were combined in DCM (10 mL) according to **GSP 3**. Product (**(2*R*,6*S*)-9a**, 89.0 mg, 0.36 mmol, 36%) and product (**(2*R*,6*R*)-9a**, 79.1 mg, 0.32 mmol, 32%) were obtained as colourless solids.

HRMS (**(2*R*,6*S*)-9a**): *m*/*z* = found 270.1103 $[M+Na]^+$, calculated 270.11006 $[M+Na]^+$.

HRMS (**(2*R*,6*R*)-9a**): *m*/*z* = found 270.1109 $[M+Na]^+$, calculated 270.11006 $[M+Na]^+$.

^{1}H NMR (**(2*R*,6*S*)-9a** chair, 600 MHz, $CDCl_3$): δ = 8.35 (s, 1H, -C$\underline{H}$O), 7.40 – 7.32 (m, 4H, $Ar_{ortho/meta}$-$\underline{H}$), 7.28 (m, 1H, Ar_{para}-$\underline{H}$), 5.03 (dd, ^3J = 5.7 Hz, ^3J = 3.6 Hz, 1H, -$C^2\underline{H}$-), 4.78 (dd, ^3J = 5.1 Hz, ^3J = 5.1 Hz, 1H, -C$\underline{H}$-), 3.29 (s, 3H, -C$\underline{H}_3$), 2.38 (m, 1H, -C$\underline{H}_2$-C^6H-), 2.23 (m, 1H, -C$\underline{H}_2$-C^2H-), 2.10 (m, 1H, -CH_2-C$\underline{H}_2$-CH_2-), 1.92 (m, 1H, -C$\underline{H}_2$-C^6H-), 1.81 – 1.72 (m, 2H, -C$\underline{H}_2$-C$\underline{H}_2$-C^2H-).

^{1}H NMR (**(2*R*,6*S*)-9a** half-chair, 600 MHz, $CDCl_3$): δ = 8.34 (s, 1H, -C$\underline{H}$O), 7.39 – 7.33 (m, 2H, Ar_{ortho}-$\underline{H}$), 7.29 – 7.26 (m, 2H, Ar_{meta}-$\underline{H}$), 7.20 (t, ^3J = 7.4 Hz, ^3J = 7.4 Hz, 1H, Ar_{para}-$\underline{H}$), 5.81 (d, ^3J = 5.9 Hz, 1H, -$C^6\underline{H}$-), 4.17 (d, ^3J = 5.8 Hz, 1H, -$C^2\underline{H}$-), 3.09 (s, 3H, -C$\underline{H}_3$), 2.45 (m, 1H, -C$\underline{H}_2$-C^6H-), 2.31 (m, 1H, -C$\underline{H}_2$-C^2H-), 1.84 (m, 1H, -C$\underline{H}_2$-C^6H-), 1.77 (m, 2H, -CH_2-C$\underline{H}_2$-CH_2-), 1.69 (m, 1H, -C$\underline{H}_2$-C^2H-).

^{1}H NMR (**(2*R*,6*R*)-9a**, 600 MHz, $CDCl_3$): δ = 8.81 (s, 1H, -C$\underline{H}$O), 7.43 – 7.30 (m, 5H, Ar-$\underline{H}$), 5.28 (dd, ^3J = 5.9 Hz, ^3J = 2.1 Hz, 1H, -$C^2\underline{H}$-), 4.60 (dd, ^3J = 11.2 Hz, ^3J = 3.3 Hz, 1H, -$C^6\underline{H}$-), 3.80 (s, 3H, -C$\underline{H}_3$), 2.36 (m, 1H, -C$\underline{H}_2$-C^2H-), 1.93 – 1.76 (m, 4H, -C$\underline{H}_2$-C$\underline{H}_2$-C$\underline{H}_2$-), 1.52 (m, 1H, -CH_2-C$\underline{H}_2$-CH_2-).

^{13}C{^{1}H} NMR (**(2*R*,6*S*)-9a** chair, 151 MHz, $CDCl_3$): δ = 171.31 (-$\underline{C}$OO-), 163.46 (-$\underline{C}$HO), 138.85 (-C^6H-$\underline{C}$-), 128.63 (Ar_{ortho}-$\underline{C}$), 127.86 (Ar_{para}-$\underline{C}$), 127.61 (Ar_{meta}-$\underline{C}$), 56.31 (-$\underline{C}^6$-), 51.83 (-$\underline{C}H_3$), 49.55 (-$\underline{C}^2$-), 28.03 (-$\underline{C}H_2$-C^6H-), 24.92 (-$\underline{C}H_2$-C^2H-), 17.77 (-CH_2-$\underline{C}H_2$-CH_2-).

^{13}C{^{1}H} NMR (**(2*R*,6*S*)-9a** half-chair, 151 MHz, $CDCl_3$): δ = 170.61 (-$\underline{C}$OO-), 164.27 (-$\underline{C}$HO), 138.54 (-C^6H-$\underline{C}$-), 128.15 (Ar_{ortho}-$\underline{C}$), 128.13 (Ar_{meta}-$\underline{C}$), 127.27 (Ar_{para}-$\underline{C}$), 54.64 (-$\underline{C}^2$-), 51.79 (-$\underline{C}H_3$), 49.02 (-$\underline{C}^6$-), 25.95 (-$\underline{C}H_2$-C^2H-), 25.48 (-$\underline{C}H_2$-C^6H-), 17.36 (-CH_2-$\underline{C}H_2$-CH_2-).

^{13}C{^{1}H} NMR (**(2*R*,6*R*)-9a**, 151 MHz, $CDCl_3$): δ = 171.67 (-$\underline{C}$OO-), 163.87 (-$\underline{C}$HO), 138.39 (-C^6H-$\underline{C}$-), 128.33 (Ar_{ortho}-$\underline{C}$), 128.72 (Ar_{para}-$\underline{C}$), 128.61 (Ar_{meta}-$\underline{C}$), 59.59 (-$\underline{C}^6$-), 52.60 (-$\underline{C}H_3$), 52.11 (-$\underline{C}^2$-), 33.58 (-$\underline{C}H_2$-C^6H-), 26.46 (-$\underline{C}H_2$-C^2H-), 21.38 (-CH_2-$\underline{C}H_2$-CH_2-).

Methyl (2*R*,6*S*)-6-(3,5-dimethylphenyl)-1-formylpiperidine-2-carboxylate (**(2*R*,6*S*)-9c**)

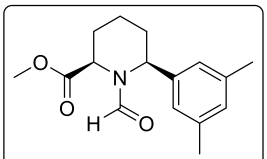

(2*R*,6*S*)-9c
$C_{16}H_{21}NO_3$
275.35 g · mol^{-1}

Cross-coupled product (**3c**, 0.86 g, 3.16 mmol, 1.0 equiv) and Pd/C (33.6 mg, 0.32 mmol, 10 mol %) were combined in MeOH (10 mL) according to **GSP 2**. Product (**(2*R*,6*S*)-9c**, 0.58 g, 2.12 mmol, 67%) was obtained as colourless solid.

LC–MS: *m*/*z* = found 276.1633 [M+H]$^+$, calculated 276.1594 [M+H]$^+$.

^{1}**H NMR** (**(2*R*,6*S*)-9c** chair, 600 MHz, $CDCl_3$): δ = 8.30 (s, 1H, -CHO), 6.96 (s, 2H, Ar_{ortho}-H), 6.90 (m, 1H, Ar_{para}-H), 4.99 (dd, ^3J = 4.7 Hz, ^3J = 4.7 Hz, 1H, -C^2H-), 4.67 (dd, ^3J = 5.2 Hz, ^3J = 5.2 Hz, 1H, -C^6H-), 3.35 (s, 3H, -COO-CH_3), 2.34 (m, 1H, -CH_2-C^6H-), 2.30 (s, 6H, -C-CH_3), 2.20 (m, 1H, -CH_2-C^2H-), 1.88 (m, 1H, -CH_2-C^6H-), 1.81 – 1.64 (m, 3H, -CH_2-CH_2-C^2H-).

^{1}**H NMR** (**(2*R*,6*S*)-9c** half-chair, 600 MHz, $CDCl_3$): δ = 8.34 (s, 1H, -CHO), 6.94 (s, 2H, Ar_{ortho}-H), 6.83 (s, 1H, Ar_{para}-H), 5.71 (d, ^3J = 5.7 Hz, 1H, -C^6H-), 4.15 (d, ^3J = 5.8 Hz, 1H, -C^2H-), 3.15 (s, 3H, -COO-CH_3), 2.45 (m, 1H, -CH_2-C^6H-), 2.40 (m, 1H, -CH_2-C^2H-), 2.33 (m, 1H, -CH_2-C^6H-), 2.28 (s, 6H, -C-CH_3), 1.78 – 1.71 (m, 3H, -CH_2-CH_2-C^6H-), 1.66 (m, 1H, -CH_2-C^2H-).

^{13}**C{^{1}H} NMR** (**(2*R*,6*S*)-9c** chair, 151 MHz, $CDCl_3$): δ = 171.50 (-COO-), 163.56 (-CHO), 138.85 (-C^6H-C-), 138.13 (Ar_{meta}-C), 129.42 (Ar_{para}-C), 125.36 (Ar_{ortho}-C), 56.49 (-C^6-), 51.79 (-COOO-CH_3), 49.71 (-C^2-), 28.36 (-CH_2-C^6H-), 24.94 (-CH_2-C^2H-), 21.54 (-C-CH_3), 17.93 (-CH_2-CH_2-CH_2-).

^{13}**C{^{1}H} NMR** (**(2*R*,6*S*)-9c** half-chair, 151 MHz, $CDCl_3$): δ = 170.75 (-COO-), 164.12 (-CHO), 137.47 (-C^6H-C-), 128.55 (Ar_{para}-C), 127.93 (Ar_{meta}-C), 125.76 (Ar_{ortho}-C), 54.55 (-C^2-), 51.68 (-COOO-CH_3), 49.08 (-C^6-), 25.95 (-CH_2-C^6H-), 25.72 (-CH_2-C^2H-), 21.57 (-C-CH_3), 17.39 (-CH_2-CH_2-CH_2-).

Methyl (2*R*,6*S*/6*R*)-1-formyl-6-(4-(trifluoromethyl)phenyl)piperidine-2-carboxylate (**(2*R*,6*S*)-9d**/**(2*R*,6*R*)-9d**)

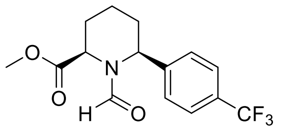

(2*R*,6*S*)-9d
$C_{15}H_{16}F_3NO_3$
315.29 g · mol^{-1}

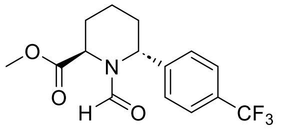

(2*R*,6*R*)-9d
$C_{15}H_{16}F_3NO_3$
315.29 g · mol^{-1}

Cross-coupled product (**3d**, 1.13 g, 3.60 mmol, 1.0 equiv) and Pd/C (38.4 mg, 0.36 mmol, 10 mol %) were combined in MeOH (10 mL) according to **GSP 2**. Product (**(2*R*,6*S*)-9d**, 0.93 g, 2.96 mmol, 82%) and product (**(2*R*,6*R*)-9d**, 45.5 mg, 0.14 mmol, 4%) were obtained as colourless solids.

HRMS (**(2*R*,6*S*)-9d**): *m*/*z* = found 338.0974 $[M+Na]^+$, calculated 338.09745 $[M+Na]^+$.

HRMS (**(2*R*,6*R*)-9d**): *m*/*z* = found 338.0974 $[M+Na]^+$, calculated 338.09745 $[M+Na]^+$.

^{1}H NMR (**(2*R*,6*S*)-9d** chair, 600 MHz, $CDCl_3$): δ = 8.36 (s, 1H, -CHO), 7.62 (d, ^3J = 8.1 Hz, 2H, Ar_{meta}-H), 7.56 – 7.52 (m, 2H, Ar_{ortho}-H), 5.04 (dd, ^3J = 4.7 Hz, ^3J = 4.7 Hz, 1H, -C^2H-), 4.78 (dd, ^3J = 5.1 Hz, ^3J = 5.1 Hz, 1H, -C^6H-), 3.33 (s, 3H, -CH_3), 2.37 (m, 1H, -CH_2-C^6H-), 2.23 (m, 1H, -CH_2-C^2H-), 2.04 (m, 1H, -CH_2-CH_2-CH_2-), 1.97 (m, 1H, -CH_2-C^6H-), 1.85 – 1.76 (m, 2H, -CH_2-CH_2-C^2H-).

^{1}H NMR (**(2*R*,6*S*)-9d** half-chair, 600 MHz, $CDCl_3$): δ = 8.36 (s, 1H, -CHO), 7.49 (d, ^3J = 8.2 Hz, 2H, Ar_{ortho}-H), 7.56 – 7.52 (m, 2H, Ar_{meta}-H), 5.81 (d, ^3J = 5.8 Hz, 1H, -C^6H-), 4.22 (d, ^3J = 5.9 Hz, 1H, -C^2H-), 3.12 (s, 3H, -CH_3), 2.42 (m, 1H, -CH_2-C^2H-), 2.33 (m, 1H, -CH_2-C^6H-), 2.16 (m, 1H, -CH_2-CH_2-CH_2-), 1.89 (m, 1H, -CH_2-C^6H-), 1.80 (m, 1H, -CH_2-CH_2-CH_2-), 1.72 (m, 1H, -CH_2-C^2H-).

^{1}H NMR (**(2*R*,6*R*)-9d**, 600 MHz, $CDCl_3$): δ = 7.81 (s, 1H, -CHO), 7.67 (d, ^3J = 8.1 Hz, 2H, Ar_{meta}-H), 7.51 (d, ^3J = 8.0 Hz, 2H, Ar_{ortho}-H), 5.26 (dd, ^3J = 6.3 Hz, ^3J = 2.2 Hz, 1H, -C^2H-), 4.60 (dd, ^3J = 11.7 Hz, ^3J = 3.3 Hz, 1H, -C^6H-), 3.80 (s, 3H, -CH_3), 2.36 (m, 1H, -CH_2-C^2H-), 1.93 – 1.76 (m, 4H, -CH_2-CH_2-CH_2-), 1.53 (m, 1H, -CH_2-CH_2-CH_2-).

^{13}C{^{1}H} NMR (**(2*R*,6*S*)-9d** chair, 151 MHz, $CDCl_3$): δ = 171.13 (-COO-), 163.71 (-CHO), 143.18 (-C^6H-C-), 130.22 (q, ^2J = 33.0 Hz, C-CF_3), 128.03 (Ar_{ortho}-C), 125.60 (q, ^3J = 3.7 Hz, Ar_{meta}-C), 124.02 (q, ^1J = 272.5 Hz, -CF_3), 56.03 (-C^6-), 51.94 (-CH_3), 49.76 (-C^2-), 27.96 (-CH_2-C^6H-), 24.75 (-CH_2-C^2H-), 17.51 (-CH_2-CH_2-CH_2-).

^{13}C{^{1}H} NMR (**(2*R*,6*S*)-9d** half-chair, 151 MHz, $CDCl_3$): δ = 170.38 (-COO-), 164.59 (-CHO), 142.81 (-C^6H-C-), 129.59 (q, ^2J = 32.2 Hz, C-CF_3), 128.52 (Ar_{ortho}-C), 125.06 (q, ^3J = 3.8 Hz, Ar_{meta}-C), 124.11 (q, ^1J = 271.2, -CF_3), 54.74 (-C^2-), 51.88 (-CH_3), 49.07 (-C^6-), 25.76 (-CH_2-C^6H-), 25.46 (-CH_2-C^2H-), 17.23 (-CH_2-CH_2-CH_2-).

^{13}C{^{1}H} NMR (**(2*R*,6*R*)-9d**, 151 MHz, $CDCl_3$): δ = 171.58 (-COO-), 163.72 (-CHO), 142.95 (-C^6H-C-), 130.98 (q, ^2J = 33.2 Hz, C-CF_3), 128.91 (Ar_{ortho}-C), 126.35 (q, ^3J = 3.8 Hz, Ar_{meta}-C), 123.95 (q, ^1J = 272.5 Hz, -CF_3), 58.54 (-C^6-), 52.69 (-CH_3), 52.24 (-C^2-), 33.84 (-CH_2-C^6H-), 26.34 (-CH_2-C^2H-), 21.08 (-CH_2-CH_2-CH_2-).

Methyl (2*R*,6*S*/6*R*)-1-formyl-6-(4-methoxyphenyl)piperidine-2-carboxylate **((2R,6*S*)-9e/(2*R*,6*R*)-9e)**

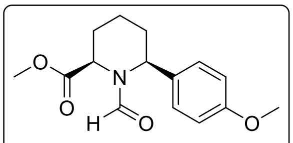

(2*R*,6*S*)-9e
$C_{15}H_{19}NO_4$
277.32 g·mol^{-1}

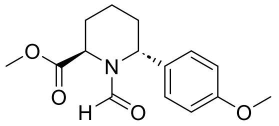

(2*R*,6*R*)-9e
$C_{15}H_{19}NO_4$
277.32 g·mol^{-1}

Cross-coupled product (**3e**, 0.59 g, 2.13 mmol, 1.0 equiv) and Pd/C (22.7 mg, 0.21 mmol, 10 mol %) were combined in MeOH (10 mL) according to **GSP 2**. Product (**(2*R*,6*S*)-9e**, 0.35 g, 1.26 mmol, 59%) and product (**(2*R*,6*S*)-9e**, 47.3 mg, 0.17 mmol, 8%) were obtained as colourless solids.

LC–MS (**(2*R*,6*S*)-9e**): *m*/*z* = found 278.1418 $[M+H]^+$, calculated 278.1387 $[M+H]^+$.

LC–MS (**(2*R*,6*R*)-9e**): *m*/*z* = found 278.1422 $[M+H]^+$, calculated 278.1387 $[M+H]^+$.

^{1}H NMR (**(2*R*,6*S*)-9e** chair, 600 MHz, $CDCl_3$): δ = 8.33 (s, 1H, -C$\underline{H}$O), 7.30 – 7.27 (m, 2H, Ar_{ortho}-$\underline{H}$), 6.87 (d, ^3J = 8.8 Hz, 2H, Ar_{meta}-$\underline{H}$), 5.02 (dd, ^3J = 5.7 Hz, ^3J = 3.5 Hz, 1H, -$C^2\underline{H}$-), 4.74 (dd, ^3J = 5.0 Hz, ^3J = 5.0 Hz, 1H, -$C^6\underline{H}$-), 3.80 (s, 3H, -CO-C$\underline{H}_3$), 3.33 (s, 3H, -COO-C$\underline{H}_3$), 2.39 (m, 1H, -C$\underline{H}_2$-C^2H-), 2.35 (m, 1H, -C$\underline{H}_2$-C^6H-), 2.09 (m, 1H, -CH_2-C$\underline{H}_2$-CH_2-), 1.90 (m, 1H, -C$\underline{H}_2$-C^6H-), 1.85 – 1.63 (m, 2H, -C$\underline{H}_2$-C$\underline{H}_2$-C^2H-).

^{1}H NMR (**(2*R*,6*S*)-9e** half-chair, 600 MHz, $CDCl_3$): δ = 8.32 (s, 1H, -C$\underline{H}$O), 7.30 – 7.27 (m, 2H, Ar_{ortho}-$\underline{H}$), 6.80 (d, ^3J = 8.8 Hz, 2H, Ar_{meta}-$\underline{H}$), 5.75 (d, ^3J = 5.9 Hz, 1H, -$C^6\underline{H}$-), 4.14 (d, ^3J = 4.7 Hz, 1H, -$C^2\underline{H}$-), 3.77 (s, 3H, -CO-C$\underline{H}_3$), 3.15 (s, 3H, -COO-C$\underline{H}_3$), 2.30 (m, 1H, -C$\underline{H}_2$-C^2H-), 2.27 – 2.19 (m, 2H, -C$\underline{H}_2$-C$\underline{H}_2$-C^6H-), 1.83 (m, 1H, -C$\underline{H}_2$-C^6H-), 1.80 – 1.66 (m, 2H, -C$\underline{H}_2$-C$\underline{H}_2$-C^2H-).

^{1}H NMR (**(2*R*,6*R*)-9e**, 600 MHz, $CDCl_3$): δ = 7.80 (s, 1H, -C$\underline{H}$O), 7.28 (d, ^3J = 8.7 Hz, 2H, Ar_{ortho}-$\underline{H}$), 6.92 (d, ^3J = 8.7 Hz, 2H, Ar_{meta}-$\underline{H}$), 5.29 (dd, ^3J = 6.1 Hz, ^3J = 2.0 Hz, 1H, -$C^2\underline{H}$-), 4.55 (dd, ^3J = 11.5 Hz, ^3J = 3.2 Hz, 1H, -$C^6\underline{H}$-), 3.82 (s, 3H, -CO-C$\underline{H}_3$), 3.79 (s, 3H, -COO-C$\underline{H}_3$), 2.35 (m, 1H, -C$\underline{H}_2$-C^2H-), 1.89 – 1.81 (m, 3H, -C$\underline{H}_2$-C$\underline{H}_2$-C^6H-), 1.78 (m, 1H, -C$\underline{H}_2$-C^2H-),1.51 (m, 1H, -CH_2-C$\underline{H}_2$-CH_2-).

^{13}C{^{1}H} NMR (**(2*R*,6*S*)-9e** chair, 151 MHz, $CDCl_3$): δ = 171.44 (-$\underline{C}$OO-), 163.23 (-$\underline{C}$HO), 159.11 (-$\underline{C}$O-CH_3), 130.77 (-C^6H-$\underline{C}$-), 128.88 (Ar_{ortho}-$\underline{C}$), 113.90 (Ar_{meta}-$\underline{C}$), 55.79 (-$\underline{C}^6$-), 55.45 (-CO-$\underline{C}H_3$), 51.89 (-COO-$\underline{C}H_3$), 49.43 (-$\underline{C}^2$-), 28.18 (-$\underline{C}H_2$-C^6H-), 24.96 (-$\underline{C}H_2$-C^2H-), 17.78 (-CH_2-$\underline{C}H_2$-CH_2-).

^{13}C{^{1}H} NMR (**(2*R*,6*S*)-9e** half-chair, 151 MHz, $CDCl_3$): δ = 170.79 (-$\underline{C}$OO-), 164.11 (-$\underline{C}$HO), 158.62 (-$\underline{C}$O-CH_3), 130.72 (-C^6H-$\underline{C}$-), 128.88 (Ar_{ortho}-$\underline{C}$), 113.42 (Ar_{meta}-$\underline{C}$), 55.38 (-CO-$\underline{C}H_3$), 54.49 (-$\underline{C}^2$-), 51.89 (-COO-$\underline{C}H_3$), 48.45 (-$\underline{C}^6$-), 25.96 (-$\underline{C}H_2$-C^6H-), 25.68 (-$\underline{C}H_2$-C^2H-), 17.38 (-CH_2-$\underline{C}H_2$-CH_2-).

^{13}C{^{1}H} NMR (**(2*R*,6*R*)-9e**, 151 MHz, $CDCl_3$): δ = 171.71 (-$\underline{C}$OO-), 163.68 (-$\underline{C}$HO), 159.81 (-$\underline{C}$O-CH_3), 130.09 (-C^6H-$\underline{C}$-), 129.79 (Ar_{ortho}-$\underline{C}$), 114.67 (Ar_{meta}-$\underline{C}$), 59.02 (-$\underline{C}^6$-), 55.49 (-CO-$\underline{C}H_3$), 52.58 (-COO-$\underline{C}H_3$), 52.04 (-$\underline{C}^2$-), 33.57 (-$\underline{C}H_2$-C^6H-), 26.49 (-$\underline{C}H_2$-C^2H-), 21.56 (-CH_2-$\underline{C}H_2$-CH_2-).

Methyl (2*R*,6*S*)-1-formyl-6-(4-(hydroxymethyl)phenyl)piperidine-2-carboxylate (**(2*R*,6*S*)-9f**

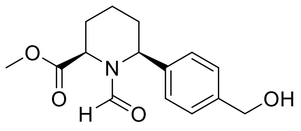

(2*R*,6*S*)-9f
$C_{15}H_{19}NO_4$
277.32 g · mol^{-1}

Cross-coupled product (**3f**, 0.64 g, 2.34 mmol, 1.0 equiv) and Pd/C (24.9 mg, 0.23 mmol, 10 mol %) were combined in MeOH (10 mL) according to **GSP 2**. Product (**(2*R*,6*S*)-9f**, 0.47 g, 1.68 mmol, 72%) was obtained as colourless solid.

HRMS (**(2*R*,6*S*)-9f**): *m*/*z* = found 300.1196 [M+Na]$^+$, calculated 300.12063 [M+Na]$^+$.

^{1}H NMR (**(2*R*,6*S*)-9f** chair, 600 MHz, $CDCl_3$): δ = 8.35 (s, 1H, -CHO), 7.30 – 7.23 (m, 2H, Ar_{ortho}-H), 7.16 (d, ^3J = 7.8 Hz, 2H, Ar_{meta}-H), 5.03 (dd, ^3J = 5.6 Hz, ^3J = 3.6 Hz, 1H, -C^2H-), 4.76 (dd, ^3J = 5.1 Hz, ^3J = 5.1 Hz, 1H, -C^6H-), 3.33 (s, 3H, -COO-CH_3), 2.38 (m, 1H, -CH_2-C^6H-), 2.34 (s, 2H, -CH_2-OH), 2.24 (m, 1H, -CH_2-C^2H-), 2.10 (m, 1H, -CH_2-CH_2-CH_2-), 1.91 (m, 1H, -CH_2-C^6H-), 1.81 – 1.72 (m, 2H, -CH_2-CH_2-C^2H-).

^{1}H NMR (**(2*R*,6*S*)-9f** half-chair, 600 MHz, $CDCl_3$): δ = 8.35 (s, 1H, -CHO), 7.30 – 7.23 (m, 2H, Ar_{ortho}-H), 7.09 (d, ^3J = 7.9 Hz, 2H, Ar_{meta}-H), 5.78 (d, ^3J = 5.9 Hz, 1H, -C^6H-), 4.17 (d, ^3J = 5.7 Hz, 1H, -C^2H-), 3.13 (s, 3H, -COO-CH_3), 2.32 (m, 1H, -CH_2-CH_2-CH_2-), 2.31 (s, 2H, -CH_2-OH), 2.29 – 2.19 (m, 2H, -CH_2-CH_2-CH_2-), 1.83 (m, 1H, -CH_2-C^6H-), 1.76 (m, 1H, -CH_2-CH_2-CH_2-), 1.69 (m, 1H, -CH_2-C^2H-).

^{13}C{^{1}H} NMR (**(2*R*,6*S*)-9f** chair, 151 MHz, $CDCl_3$): δ = 171.34 (-COO-), 163.41 (-CHO), 137.54 (-C-CH_2-OH), 135.76 (-C^6H-C-), 129.25 (Ar_{meta}-C), 127.52 (Ar_{ortho}-C), 56.15 (-C^6-), 51.80 (-COO-CH_3), 49.56 (-C^2-), 28.11 (-CH_2-C^6H-), 24.90 (-CH_2-C^2H-), 21.11 (-C-CH_2-OH), 17.76 (-CH_2-CH_2-CH_2-).

^{13}C{^{1}H} NMR (**(2*R*,6*S*)-9f** half-chair, 151 MHz, $CDCl_3$): δ = 170.66 (-COO-), 164.21 (-CHO), 136.79 (-C-CH_2-OH), 135.43 (-C^6H-C-), 128.18 (Ar_{meta}-C), 128.02 (Ar_{ortho}-C), 54.58 (-C^2-), 51.75 (-COO-CH_3), 48.85 (-C^6-), 25.93 (-CH_2-C^6H-), 25.58 (-CH_2-C^2H-), 21.09 (-C-CH_2-OH), 17.34 (-CH_2-CH_2-CH_2-).

Methyl(2*R*,6*S*/6*R*)-6-(3-aminophenyl)-1-formylpiperidine-2-carboxylate
((2*R*,6*S*)-9h/(2*R*,6*R*)-9h)

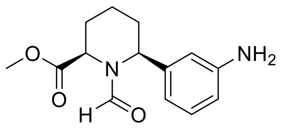

(2*R*,6*S*)-9h
$C_{14}H_{18}N_2O_3$
262.31 g · mol^{-1}

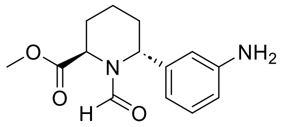

(2*R*,6*R*)-9h
$C_{14}H_{18}N_2O_3$
262.31 g · mol^{-1}

Cross-coupled product (**3h**, 0.27 g, 1.04 mmol, 1.0 equiv) and Pd/C (11.0 mg, 0.10 mmol, 10 mol %) were combined in MeOH (10 mL) according to **GSP 2**. Product (**(2*R*,6*S*)-9h**, 0.12 g, 0.46 mmol, 44%) and product (**(2*R*,6*R*)-9h**, 8.1 mg, 0.03 mmol, 3%) were obtained as colourless solids.

HRMS (**(2*R*,6*S*)-9h**): *m*/*z* = found 263.1395 $[M+H]^+$, calculated 263.13902 $[M+H]^+$.

HRMS (**(2*R*,6*R*)-9h**): *m*/*z* = found 263.1395 $[M+H]^+$, calculated 263.13902 $[M+H]^+$.

^{1}H NMR (**(2*R*,6*S*)-9h** chair, 600 MHz, $CDCl_3$): δ = 8.35 (s, 1H, -C$\underline{H}$O), 7.48 (s, 1H, -C-C$\underline{H}$-C-), 7.37 (t, ^3J = 7.9 Hz, ^3J = 7.9 Hz, 1H, -CH-C$\underline{H}$-CH-), 7.31 (d, ^3J = 8.0 Hz, 1H, Ar_{para}-$\underline{H}$), 7.28 (m, 1H, Ar_{ortho}-$\underline{H}$), 4.99 (dd, ^3J = 4.8 Hz, ^3J = 4.8 Hz, 1H, -$C^2\underline{H}$-), 4.81 (dd, ^3J = 5.0 Hz, ^3J = 5.0 Hz, 1H, -$C^6\underline{H}$-), 3.32 (s, 3H, -$C\underline{H}_3$), 2.33 (m, 1H, -$C\underline{H}_2$-C^6H-), 2.19 (m, 1H, -$C\underline{H}_2$-C^2H-), 2.01 (m, 1H, -CH_2-$C\underline{H}_2$-CH_2-), 1.95 (m, 1H, -$C\underline{H}_2$-C^6H-), 1.83 – 1.70 (m, 2H, -$C\underline{H}_2$-$C\underline{H}_2$-C^2H-).

^{1}H NMR (**(2*R*,6*S*)-9h** half-chair, 600 MHz, $CDCl_3$): δ = 8.30 (s, 1H, -C$\underline{H}$O), 7.53 (s, 1H, -C-C$\underline{H}$-C-), 7.28 (m, 1H, -CH-C$\underline{H}$-CH-), 7.35 – 7.32 (m, 2H, $Ar_{ortho/para}$-$\underline{H}$), 5.68 (d, ^3J = 4.1 Hz, 1H, -$C^6\underline{H}$-), 4.24 (d, ^3J = 5.6 Hz, 1H, -$C^2\underline{H}$-), 3.16 (s, 3H, -$C\underline{H}_3$), 2.37 – 2.28 (m, 2H, -$C\underline{H}_2$-CH_2-$C\underline{H}_2$-), 2.12 (m, 1H, -CH_2-$C\underline{H}_2$-CH_2-), 1.85 (m, 1H, -$C\underline{H}_2$-C^6H-), 1.82 – 1.70 (m, 2H, -$C\underline{H}_2$-$C\underline{H}_2$-C^2H-).

^{1}H NMR (**(2*R*,6*R*)-9h**, 600 MHz, $CDCl_3$): δ = 7.77 (s, 1H, -C$\underline{H}$O), 7.40 (t, ^3J = 7.8 Hz, ^3J = 7.8 Hz, 1H, Ar_{meta}-$\underline{H}$), 7.32 (d, ^3J = 7.3 Hz, 1H, Ar_{ortho}-$\underline{H}$), 7.28 – 7.23 (m, 2H, -C-C$\underline{H}$-C-C$\underline{H}$-), 5.24 (dd, ^3J = 4.2 Hz, ^3J = 1.9 Hz, 1H, -$C^2\underline{H}$-), 4.62 (dd, ^3J = 11.6 Hz, ^3J = 3.2 Hz, 1H, -$C^6\underline{H}$-), 3.80 (s, 3H, -$C\underline{H}_3$), 2.36 (m, 1H, -$C\underline{H}_2$-C^2H-), 1.93 – 1.78 (m, 4H, -$C\underline{H}_2$-$C\underline{H}_2$-$C\underline{H}_2$-), 1.52 (m, 1H, -CH_2-$C\underline{H}_2$-CH_2-).

$^{13}C\{^1H\}$ NMR (**(2*R*,6*S*)-9h** chair, 151 MHz, $CDCl_3$): δ = 171.33 (-$\underline{C}$OO-), 164.52 (-$\underline{C}$HO), 141.14 (-C^6H-$\underline{C}$-), 133.89 (-$\underline{C}$-NH_2), 130.17 (Ar_{meta}-$\underline{C}$), 126.01 (Ar_{para}-C), 121.59 (-C-$\underline{C}$H-C-), 121.59 (Ar_{ortho}-C), 56.13 (-$\underline{C}^6$-), 52.28 (-$\underline{C}H_3$), 50.10 (-$\underline{C}^2$-), 27.72 (-$\underline{C}H_2$-C^6H-), 24.60 (-$\underline{C}H_2$-C^2H-), 17.40 (-CH_2-$\underline{C}H_2$-CH_2-).

$^{13}C\{^1H\}$ NMR (**(2*R*,6*S*)-9h** half-chair, 151 MHz, $CDCl_3$): δ = 170.59 (-$\underline{C}$OO-), 165.30 (-$\underline{C}$HO), 141.21 (-C^6H-$\underline{C}$-), 132.11 (-$\underline{C}$-NH_2), 129.75 (Ar_{meta}-$\underline{C}$), 126.82 (Ar_{para}-C), 123.17 (-C-$\underline{C}$H-C-), 121.76 (Ar_{ortho}-C), 55.07 (-$\underline{C}^2$-), 52.28 (-$\underline{C}H_3$), 49.42 (-$\underline{C}^6$-), 25.60 (-$\underline{C}H_2$-C^2H-), 25.45 (-$\underline{C}H_2$-C^6H-), 17.20 (-CH_2-$\underline{C}H_2$-CH_2-).

$^{13}C\{^1H\}$ NMR (**(2*R*,6*R*)-9h**, 151 MHz, $CDCl_3$): δ = 171.11 (-$\underline{C}$OO-), 164.36 (-$\underline{C}$HO), 139.72 (-C^6H-$\underline{C}$-), 134.97 (-$\underline{C}$-NH_2), 130.76 (Ar_{meta}-$\underline{C}$), 126.73 (-C-$\underline{C}$H-C-), 121.96 (Ar_{para}-C), 121.53 (Ar_{ortho}-C), 59.29 (-$\underline{C}^6$-), 52.67 (-$\underline{C}H_3$), 52.39 (-$\underline{C}^2$-), 32.93 (-$\underline{C}H_2$-C^6H-), 26.01 (-$\underline{C}H_2$-C^2H-), 20.84 (-CH_2-$\underline{C}H_2$-CH_2-).

Methyl (2*R*,6*S*)-1-formyl-6-(naphthalen-2-yl)piperidine-2-carboxylate (**(2*R*,6*S*)-9k**)

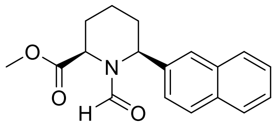

(2*R*,6*S*)-9k
$C_{18}H_{19}NO_3$
297.35 g·mol^{-1}

Cross-coupled product (**3k**, 0.51 g, 1.74 mmol, 1.0 equiv) and Pd/C (18.5 mg, 0.17 mmol, 10 mol %) were combined in MeOH (10 mL) according to **GSP 2**. Product (**(2*R*,6*S*)-9k**, 0.22 g, 0.75 mmol, 43%) was obtained as colourless solid.

LC–MS: *m*/*z* = found 298.1454 [M+H]$^+$, calculated 298.1438 [M+H]$^+$.

^{1}H NMR (**(2*R*,6*S*)-9k** chair, 600 MHz, $CDCl_3$): δ = 8.44 (s, 1H, -CHO), 7.85 – 7.80 (m, 2H, Ar-H), 7.77 (m, 1H, Ar-H), 7.52 – 7.43 (m, 4H, Ar-H), 5.07 (dd, ^3J = 5.7 Hz, ^3J = 3.4 Hz, 1H, -C^{2}H-), 4.95 (dd, ^3J = 5.1 Hz, ^3J = 5.1 Hz, 1H, -C^{6}H-), 3.16 (s, 3H, -CH$_3$), 2.53 (m, 1H, -CH$_2$-C^{6}H-), 2.26 (m, 1H, -CH$_2$-C^{2}H-), 2.17 (m, 1H, -CH$_2$-CH$_2$-CH$_2$-), 2.01 (m, 1H, -CH$_2$-C^{6}H-), 1.90 – 1.76 (m, 2H, -CH$_2$-CH$_2$-C^{2}H-).

^{1}H NMR (**(2*R*,6*S*)-9k** half-chair, 600 MHz, $CDCl_3$): δ = 8.41 (s, 1H, -CHO), 7.86 – 7.80 (m, 3H, Ar-H), 7.80 – 7.73 (m, 3H, Ar-H), 7.47 (m, 1H, Ar-H), 5.96 (d, ^3J = 5.9 Hz, 1H, -C^{6}H-), 4.21 (d, ^3J = 5.7 Hz, 1H, -C^{2}H-), 2.87 (s, 3H, -CH$_3$), 2.60 (m, 1H, -CH$_2$-C^{2}H-), 2.36 (m, 1H, -CH$_2$-C^{6}H-), 1.94 (m, 1H, -CH$_2$-C^{2}H-), 1.89 – 1.78 (m, 2H, -CH$_2$-CH$_2$-CH$_2$-), 1.74 (m, 1H, -CH$_2$-C^{6}H-).

^{13}C{^{1}H} NMR (**(2*R*,6*S*)-9k** chair, 151 MHz, $CDCl_3$): δ = 171.34 (-COO-), 163.41 (-CHO), 135.76 (-C^{6}H-C-), 133.16 (Ar-C), 132.78 (Ar-C), 128.22 (Ar-CH), 127.69 (Ar-CH), 126.62 (Ar-CH), 126.23 (Ar-CH), 56.67 (-C^6-), 51.86 (-CH$_3$), 49.81 (-C^2-), 28.12 (-CH$_2$-C^{6}H-), 24.94 (-CH$_2$-C^{2}H-), 17.85 (-CH$_2$-CH$_2$-CH$_2$-).

^{13}C{^{1}H} NMR (**(2*R*,6*S*)-9k** half-chair, 151 MHz, $CDCl_3$): δ = 170.41 (-COO-), 164.62 (-CHO), 135.89 (-C^{6}H-C-), 133.01 (Ar-C), 132.55 (Ar-C), 128.38 (Ar-CH), 127.70 (Ar-CH), 127.70 (Ar-CH), 127.51 (Ar-CH), 126.97 (Ar-CH), 126.46 (Ar-CH), 126.21 (Ar-CH), 54.90 (-C^2-), 51.77 (-CH$_3$), 49.38 (-C^6-), 26.02 (-CH$_2$-C^{6}H-), 25.61 (-CH$_2$-C^{2}H-), 17.47 (-CH$_2$-CH$_2$-CH$_2$-).

Methyl (2*R*,6*S*/6*R*)-1-formyl-6-(phenylethynyl)piperidine-2-carboxylate **((2*R*,6*S*)-13/(2*R*,6*R*)-13)**

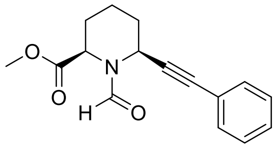

(2*R*,6*S*)-13
$C_{16}H_{17}NO_3$
271.32 g · mol^{-1}

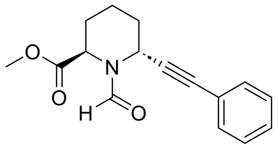

(2*R*,6*R*)-13
$C_{16}H_{17}NO_3$
271.32 g · mol-**1**

Cross-coupled product (**4**, 0.21 g, 0.78 mmol, 1.0 equiv) and $NaBH_3CN$ (0.25 g, 3.90 mmol, 5.0 equiv) and TFA (0.89 g, 600 µL, 7.83 mmol, 10.0 equiv) were combined in DCM (10 mL) according to **GSP 3**. Product (**(2*R*,6*S*)-13**, 67.7 mg, 0.25 mmol, 32%) and product (**(2*R*,6*R*)-13**, 55.0 mg, 0.20 mmol, 26%) were obtained as colourless solids [4].

LC–MS (**(2*R*,6*S*)-13**): *m*/*z* = found 272.1347 $[M+H]^+$, calculated 272.1281 $[M+H]^+$.

LC–MS (**(2*R*,6*R*)-13**): *m*/*z* = found 272.1331 $[M+H]^+$, calculated 272.1281 $[M+H]^+$.

^{1}H NMR (**(2*R*,6*S*)-13$_I$**, 500 MHz, DMSO-d_6): δ = 8.26 (s, 1H, -CHO), 7.43 – 7.36 (m, 5H, Ar-H), 5.10 (d, ^3J = 4.7 Hz, 1H, -C^{2}H-), 5.05 (d, ^3J = 6.0 Hz, 1H, -C^{6}H-), 3.47 (s, 3H, -CH_3), 2.23 (m, 1H, -CH_2-C^{2}H-), 2.01 (m, 1H, -CH_2-CH_2-CH_2-), 1.85 (m, 1H, -CH_2-C^{6}H-), 1.74 (m, 1H, -CH_2-C^{6}H-), 1.68 (m, 1H, -CH_2-CH_2-CH_2-), 1.45 (m, 1H, -CH_2-C^{2}H-).

^{1}H NMR (**(2*R*,6*S*)-13$_{II}$**, 500 MHz, DMSO-d_6): δ = 8.14 (s, 1H, -CHO), 7.43 – 7.36 (m, 5H, Ar-H), 5.38 (d, ^3J = 4.9 Hz, 1H, -C^{6}H-), 4.67 (d, ^3J = 5.8 Hz, 1H, -C^{2}H-), 3.51 (s, 3H, -CH_3), 2.23 (m, 1H, -CH_2-C^{2}H-), 2.01 (m, 1H, -CH_2-CH_2-CH_2-), 1.85 (m, 1H, -CH_2-C^{6}H-), 1.67 (m, 1H, -CH_2-CH_2-CH_2-), 1.62 – 1.53 (m, 2H, -CH_2-CH_2-CH_2-).

^{1}H NMR (**(2*R*,6*R*)-13**, 500 MHz, DMSO-d_6): δ = 8.74 (s, 1H, -CHO), 7.52 (dd, ^3J = 7.6 Hz, ^4J = 1.9 Hz, 2H, Ar_{ortho}-H), 7.48 – 7.37 (m, 3H, $Ar_{meta/para}$-H), 4.97 (dd, ^3J = 4.2 Hz, ^3J = 4.2 Hz, 1H, -C^{2}H-), 4.64 (dd, ^3J = 10.4 Hz, ^3J = 3.3 Hz, 1H, -C^{6}H-), 3.70 (s, 3H, -CH_3), 2.09 – 1.99 (m, 2H, -CH_2-CH_2-CH_2-), 1.75 (m, 1H, -CH_2-C^{6}H-), 1.72 – 1.64 (m, 2H, -CH_2-CH_2-C^{2}H-), 1.47 (m, 1H, -CH_2-CH_2-CH_2-).

^{13}C{^{1}H} NMR (**(2*R*,6*S*)-13$_I$**, 126 MHz, DMSO-d_6): δ = 171.23 (-COO-), 162.30 (-CHO), 131.80 (Ar_{ortho}-C), 129.30 (Ar_{para}-C), 129.18 (Ar_{meta}-C), 122.31 (Ar_{ipso}-C), 87.77 (-C^{6}H-C≡C-), 83.62 (-C^{6}H-C≡C-), 52.32 (-CH_3), 47.84 (-C^6-), 45.98 (-C^2-), 31.64 (-CH_2-C^{6}H-), 24.65 (-CH_2-C^{2}H-), 17.69 (-CH_2-CH_2-CH_2-).

^{13}C{^{1}H} NMR (**(2*R*,6*S*)-13$_{II}$**, 126 MHz, DMSO-d_6): δ = 171.73 (-COO-), 162.60 (-CHO), 131.82 (Ar_{ortho}-C), 129.21 (Ar_{para}-C), 129.15 (Ar_{meta}-C), 122.39 (Ar_{ipso}-C), 87.77 (-C^{6}H-C≡C-), 83.62 (-C^{6}H-C≡C-), 53.69 (-C^2-), 52.65 (-CH_3), 39.39 (-C^6-), 30.39 (-CH_2-C^{6}H-), 25.01 (-CH_2-C^{2}H-), 17.66 (-CH_2-CH_2-CH_2-).

^{13}C{^{1}H} NMR (**(2*R*,6*R*)-13**, 126 MHz, DMSO-d_6): δ = 170.63 (-COO-), 161.57 (-CHO), 131.56 (Ar_{ortho}-C), 129.20 (Ar_{para}-C), 128.75 (Ar_{meta}-C), 121.29 (Ar_{ipso}-C), 88.33 (-C^{6}H-C≡C-),84.83 (-C^{6}H-C≡C-), 52.40 (-CH_3), 50.84 (-C^2-), 46.18 (-C^6-), 32.74 (-CH_2-C^{6}H-), 25.59 (-CH_2-C^{2}H-), 20.23 (-CH_2-CH_2-CH_2-).

Methyl (2*R*,6*S*)-6-phenylpiperidine-2-carboxylate (**(2*R*,6*S*)-11a**)

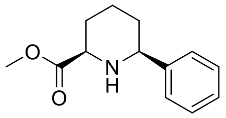

(2*R*,6*S*)-11a
$C_{13}H_{17}NO_2$
219.28 g · mol^{-1}

Hydrogenation product (**(2*R*,6*S*)-9a**, 0.69 g, 2.81 mmol, 1.0 equiv) was combined with HCl according to **GSP 4**. Product (**(2*R*,6*S*)-11a**, 0.54 g, 2.47 mmol, 88%) was obtained as colourless solid.

HRMS: *m*/*z* = found 220.1341 [M+H]$^+$, calculated 220.13321 [M+H]$^+$.

^{1}H NMR (600 MHz, $CDCl_3$): δ = 7.79 (dd, ^3J = 6.6 Hz, ^4J = 2.8 Hz, 2H, Ar$_{ortho}$-$\underline{H}$), 7.33 – 7.28 (m, 3H, Ar$_{meta/para}$-$\underline{H}$), 4.23 (dd, ^3J = 11.2 Hz, ^3J = 3.1 Hz, 1H, -C$^6\underline{H}$-), 4.04 (dd, ^3J = 11.2 Hz, ^3J = 3.4 Hz, 1H, -C$^2\underline{H}$-), 3.24 (s, 3H, -C$\underline{H}_3$), 2.56 (m, 1H, -C$\underline{H}_2$-C^{6}H-), 2.37 (m, 1H, -C$\underline{H}_2$-C^{2}H-), 2.16 (m, 1H, -C$\underline{H}_2$-C^{2}H-), 2.07 (m, 1H, -CH$_2$-C$\underline{H}_2$-CH$_2$-), 1.88 (m, 1H, -C$\underline{H}_2$-C^{6}H-), 1.68 (m, 1H, -CH$_2$-C$\underline{H}_2$-CH$_2$-).

^{13}C{^{1}H} NMR (151 MHz, $CDCl_3$): δ = 168.41 (-$\underline{C}$OO-), 136.28 (-C^{6}H-$\underline{C}$-), 129.31 (Ar$_{para}$-$\underline{C}$), 129.20 (Ar$_{ortho}$-$\underline{C}$), 128.83 (Ar$_{meta}$-$\underline{C}$), 62.32 (-$\underline{C}^6$-), 59.18 (-$\underline{C}^2$-), 53.13 (-$\underline{C}$H$_3$), 28.93 (-$\underline{C}$H$_2$-C^{6}H-), 24.38 (-$\underline{C}$H$_2$-C^{2}H-), 23.44 (-CH$_2$-$\underline{C}$H$_2$-CH$_2$-).

Methyl (2*R*,6*S*)-6-(*p*-tolyl)piperidine-2-carboxylate (**(2*R*,6*S*)-11b**)

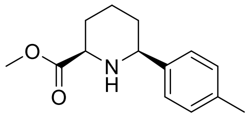

(2*R*,6*S*)-11b
$C_{14}H_{19}NO_2$
233.31 g · mol^{-1}

Hydrogenation product (**(2*R*,6*S*)-9b**, 0.62 g, 2.38 mmol, 1.0 equiv) was combined with HCl according to **GSP 4**. Product (**(2*R*,6*S*)-11b**, 0.34 g, 1.45 mmol, 61%) was obtained as colourless solid.

LC–MS: *m*/*z* = found 234.2928 [M+H]$^+$, calculated 234.1489 [M+H]$^+$.

^{1}H NMR (600 MHz, $CDCl_3$): δ = 7.56 – 7.41 (m, 2H, Ar$_{ortho}$-$\underline{H}$), 7.33 – 7.28 (m, 2H, Ar$_{meta}$-$\underline{H}$), 4.17 – 7.41 (m, 1H, -C$^6\underline{H}$-), 4.04 (dd, ^3J = 10.7 Hz, ^3J = 3.4 Hz, 1H, -C$^2\underline{H}$-), 3.38 (s, 3H, -COO-C$\underline{H}_3$), 2.36 (m, 1H, -C$\underline{H}_2$-C^{6}H-), 2.32 (s, 3H, -C-C$\underline{H}_3$), 2.26 (m, 1H, -C$\underline{H}_2$-C^{2}H-), 2.21 – 2.08 (m, 1H, -C$\underline{H}_2$-C$\underline{H}_2$-C^{2}H-), 1.98 (m, 1H, -C$\underline{H}_2$-C^{6}H-), 1.73 (m, 1H, -CH$_2$-C$\underline{H}_2$-CH$_2$-).

^{13}C{^{1}H} NMR (151 MHz, $CDCl_3$): δ = 168.48 (-$\underline{C}$OO-), 139.92 (-$\underline{C}$-CH$_3$), 132.38 (-C^{6}H-$\underline{C}$-), 129.75 (Ar$_{meta}$-$\underline{C}$), 128.54 (Ar$_{ortho}$-$\underline{C}$), 62.40 (-$\underline{C}^6$-), 59.28 (-$\underline{C}^2$-), 53.14 (-COO-$\underline{C}$H$_3$), 29.01 (-$\underline{C}$H$_2$-C^{6}H-), 25.03 (-$\underline{C}$H$_2$-C^{2}H-), 23.29 (-CH$_2$-$\underline{C}$H$_2$-CH$_2$-), 21.27 (-C-$\underline{C}$H$_3$).

Methyl (2*R*,6*S*)-6-(3,5-dimethylphenyl)piperidine-2-carboxylate (**(2*R*,6*S*)-11c**)

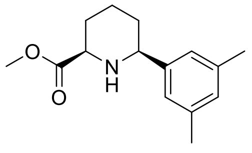

(2*R*,6*S*)-11c
$C_{15}H_{21}NO_2$
247.34 g · mol^{-1}

Hydrogenation product (**(2*R*,6*S*)-9c**, 0.58 g, 2.12 mmol, 1.0 equiv) was combined with HCl according to **GSP 4**. Product (**(2*R*,6*S*)-11c**, 0.30 g, 1.21 mmol, 57%) was obtained as colourless solid.

LC–MS: *m*/*z* = found 248.3923 $[M+H]^+$, calculated 248.1644 $[M+H]^+$.

^{1}H NMR (600 MHz, $CDCl_3$): δ = 7.23 – 7.15 (m, 2H, Ar$_{ortho}$-$\underline{H}$), 6.97 (m, 1H, -C-C$\underline{H}$-C-), 4.11 (dd, ^3J = 12.8 Hz, ^3J = 2.8 Hz, 1H, -C$^6\underline{H}$-), 4.04 (dd, ^3J = 12.7 Hz, ^3J = 3.6 Hz, 1H, -C$^2\underline{H}$-), 3.37 (s, 3H, -COO-C$\underline{H}_3$), 2.31 (m, 1H, -C$\underline{H}_2$-C^{6}H-), 2.27 (s, 6H, -C-C$\underline{H}_3$), 2.23 (m, 1H, -C$\underline{H}_2$-C^{2}H-), 2.18 – 2.09 (m, 2H, -C$\underline{H}_2$-C$\underline{H}_2$-C^{2}H-), 1.97 (m, 1H, -C$\underline{H}_2$-C^{6}H-), 1.73 (m, 1H, -CH$_2$-C$\underline{H}_2$-CH$_2$-).

^{13}C{^{1}H} NMR (151 MHz, $CDCl_3$): δ = 168.58 (-$\underline{C}$OO-), 138.84 (-$\underline{C}$-CH$_3$), 135.47 (-C^{6}H-$\underline{C}$-), 131.26 (-C-$\underline{C}$H-C-), 126.26 (Ar$_{ortho}$-$\underline{C}$), 62.58 (-$\underline{C}^6$-), 59.21 (-$\underline{C}^2$-), 53.11 (-COO-$\underline{C}$H$_3$), 29.26 (-$\underline{C}$H$_2$-C^{6}H-), 25.10 (-$\underline{C}$H$_2$-C^{2}H-), 23.36 (-CH$_2$-$\underline{C}$H$_2$-CH$_2$-), 21.19 (-C-$\underline{C}$H$_3$).

Methyl (2*R*,6*S*)-6-(4-(trifluoromethyl)phenyl)piperidine-2-carboxylate (**(2*R*,6*S*)-11d**)

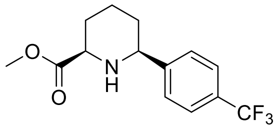

(2*R*,6*S*)-11d
$C_{14}H_{16}F_3NO_2$
287.28 g · mol^{-1}

Hydrogenation product (**(2*R*,6*S*)-9d**, 0.93 g, 2.96 mmol, 1.0 equiv) was combined with HCl according to **GSP 4**. Product (**(2*R*,6*S*)-11d**, 0.76 g, 2.63 mmol, 89%) was obtained as colourless solid.

LC–MS: *m*/*z* = found 288.1317 $[M+H]^+$, calculated 288.1206 $[M+H]^+$.

^{1}H NMR (500 MHz, $CDCl_3$): δ = 7.58 (d, ^3J = 8.0 Hz, 2H, Ar$_{meta}$-$\underline{H}$), 7.51 (d, ^3J = 8.0 Hz, 2H, Ar$_{ortho}$-$\underline{H}$), 3.75 – 3.71 (m, 4H, -C$^6\underline{H}$- and -C$\underline{H}_3$), 3.51 (dd, ^3J = 11.0 Hz, ^3J = 2.7 Hz, 1H, -C$^2\underline{H}$-), 2.22 (s, 1H, -N$\underline{H}$-), 2.10 (m, 1H, -C$\underline{H}_2$-C^{2}H-), 2.01 (m, 1H, -CH$_2$-C$\underline{H}_2$-CH$_2$-), 1.79 (m, 1H, -C$\underline{H}_2$-C^{6}H-), 1.61 – 1.42 (m, 3H, -C$\underline{H}_2$-C$\underline{H}_2$-C$\underline{H}_2$-).

^{13}C{^{1}H} NMR (126 MHz, $CDCl_3$): δ = 173.39 (-$\underline{C}$OO-), 148.67 (-C^{6}H-$\underline{C}$-), 129.66 (q, ^2J = 32.4 Hz, -$\underline{C}$-CF$_3$), 127.19 (Ar$_{ortho}$-$\underline{C}$), 125.52 (q, ^3J = 3.8 Hz, Ar$_{meta}$-$\underline{C}$), 123.25 (q, ^1J = 272.0 Hz, -CF$_3$), 61.48 (-$\underline{C}^6$-), 59.66 (-$\underline{C}^2$-), 52.20 (-$\underline{C}$H$_3$), 34.39 (-$\underline{C}$H$_2$-C^{6}H-), 28.44 (-$\underline{C}$H$_2$-C^{2}H-), 25.04 (-CH$_2$-$\underline{C}$H$_2$-CH$_2$-).

^{19}F{^{13}C} NMR (471 MHz, $CDCl_3$) δ = −62.43.

Methyl (2*R*,6*S*)-6-(4-(hydroxymethyl)phenyl)piperidine-2-carboxylate (**(2*R*,6*S*)-11f**)

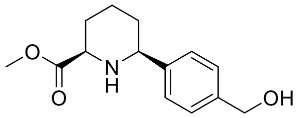

(2*R*,6*S*)-11f
$C_{14}H_{19}NO_3$
249.31 g·mol^{-1}

Hydrogenation product (**(2*R*,6*S*)-9f**, 0.47 g, 1.68 mmol, 1.0 equiv) was combined with HCl according to **GSP 4**. Product (**(2*R*,6*S*)-11f**, 0.39 g, 1.57 mmol, 93%) was obtained as colourless solid.

LC–MS: *m*/*z* = found 250.3253 [M+H]$^+$, calculated 250.1438 [M+H]$^+$.

^{1}H NMR (600 MHz, $CDCl_3$): δ = 7.40 (d, ^3J = 8.1 Hz, 2H, Ar_{ortho}-H), 7.12 (d, ^3J = 7.8 Hz, 2H Ar_{meta}-H), 4.14 (dd, ^3J = 12.8 Hz, ^3J = 2.8 Hz, 1H, -C^{6}H-), 4.01 (dd, ^3J = 11.9 Hz, ^3J = 3.3 Hz, 1H, -C^{2}H-), 3.32 (s, 3H, -CH_3), 2.29 (s, 2H, -CH_2-OH), 2.26 (m, 1H, -CH_2-C^{6}H-), 2.20 (m, 1H, -CH_2-C^{2}H-), 2.08 (m, 1H, -CH_2-CH_2-CH_2-), 2.01 (m, 1H, -CH_2-C^{2}H-), 1.92 (m, 1H, -CH_2-C^{6}H-), 1.69 (m, 1H, -CH_2-CH_2-CH_2-).

^{13}C{^{1}H} NMR (151 MHz, $CDCl_3$): δ = 168.79 (-COO-), 139.41 (-C-CH_2-OH), 133.10 (-C^{6}H-C-), 129.63 (Ar_{meta}-C), 128.31 (Ar_{ortho}-C), 61.83 (-C^6-), 58.94 (-C^2-), 52.85 (-CH_3), 29.18 (-CH_2-C^{6}H-), 25.15 (-CH_2-C^{2}H-), 23.35 (-CH_2-CH_2-CH_2-), 21.23 (-CH_2-OH).

Methyl (2*R*,6*S*)-6-(3-aminophenyl)piperidine-2-carboxylate (**(2*R*,6*S*)-11h**)

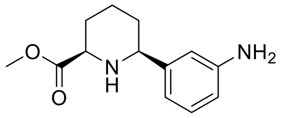

(2*R*,6*S*)-11h
$C_{13}H_{18}N_2O_2$
234.30 g·mol^{-1}

Hydrogenation product (**(2*R*,6*S*)-9h**, 0.12 g, 0.46 mmol, 1.0 equiv) was combined with HCl according to **GSP 4**. Product (**(2*R*,6*S*)-11h**, 62 mg, 0.26 mmol, 58%) was obtained as colourless solid.

HRMS: *m*/*z* = found 235.1438 [M+H]$^+$, calculated 235.14410 [M+H]$^+$.

^{1}H NMR (600 MHz, DMSO-d_6): δ = 9.60 (bs, 2H, -NH_2), 9.18 (bs, 1H, -NH-), 7.19 (t, ^3J = 7.6 Hz, ^3J = 7.6 Hz, 1H, Ar_{meta}-H), 6.91 – 6.70 (m, 3H, $Ar_{ortho/para}$-H), 4.29 (m, 1H, -C^{2}H-), 4.15 (m, 1H, -C^{6}H-), 3.78 (s, 3H, -CH_3), 2.15 (m, 1H, -CH_2-C^{2}H-), 1.93 (m, 1H, -CH_2-CH_2-CH_2-), 1.89 – 1.82 (m, 2H, -CH_2-C^{6}H-), 1.79 – 1.72 (m, 2H, -CH_2-CH_2-C^{6}H-).

^{13}C{^{1}H} NMR (151 MHz, DMSO-d_6): δ = 168.94 (-COO-), 154.19 (-C^{6}H-C-), 137.80 (-C-CH-C-), 129.54 (Ar-C), 114.79 (-C-NH_2), 60.07 (-C^6-), 57.30 (-C^2-), 52.96 (-CH_3), 28.86 (-CH_2-C^{6}H-), 25.03 (-CH_2-C^{2}H-), 22.17 (-CH_2-CH_2-CH_2-).

Methyl (2*R*,6*S*)-6-(4-(diphenylamino)phenyl)piperidine-2-carboxylate (**(2*R*,6*S*)-11j**)

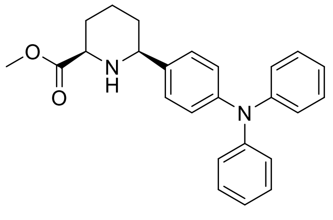

(2*R*,6*S*)-11j
$C_{25}H_{26}N_2O_2$
386.49 g·mol^{-1}

Hydrogenation product (**(2*R*,6*S*)-9j**, 0.42 g, 0.78 mmol, 1.0 equiv) was combined with HCl in MeOH (10 mL) according to **GSP 4**. Product (**(2*R*,6*S*)-11j**, 0.27 g, 0.69 mmol, 89%) was obtained as colourless solid.

LC–MS: *m*/*z* = found 387.5466 $[M+H]^+$, calculated 387.2067 $[M+H]^+$.

^{1}H NMR (600 MHz, $CDCl_3$): δ = 7.45 (d, ^3J = 8.7 Hz, 2H, Ar_{ortho}-H), 7.27 – 7.23 (m, 4H, Ar_{ortho}-H), 7.09 – 7.03 (m, 6H, $Ar_{meta/para}$-H), 6.98 (d, ^3J = 8.7 Hz, 2H, Ar_{meta}-H), 4.18 (dd, ^3J = 13.1 Hz, ^3J = 2.6 Hz, 1H, -C^6H-), 4.10 (dd, ^3J = 12.8 Hz, ^3J = 3.4 Hz, 1H, -C^2H-), 3.63 (s, 3H, -CH_3), 2.36 – 2.25 (m, 2H, -CH_2-CH_2-CH_2-), 2.19 – 2.11 (m, 2H, -CH_2-CH_2-C^2H-), 2.04 (m, 1H, -CH_2-C^6H-), 1.75 (m, 1H, -CH_2-CH_2-CH_2-).

^{13}C{^{1}H} NMR (151 MHz, $CDCl_3$): δ = 168.56 (-COO-), 149.40 (Ar_{para}-C), 147.20 (Ar_{ipso}-C), 129.60 (Ar_{ortho}-C), 127.68 (-C^6H-C-), 125.24 (Ar_{meta}-C), 123.92 (Ar_{para}-C), 122.19 (Ar_{meta}-C), 61.93 (-C^6-), 59.11 (-C^2-), 53.61 (-CH_3), 28.76 (-CH_2-C^6H-), 25.15 (-CH_2-C^2H-), 23.19 (-CH_2-CH_2-CH_2-).

(2*R*,6*S*)-6-Phenylpiperidine-2-carboxylic acid (**(2*R*,6*S*)-5a**)

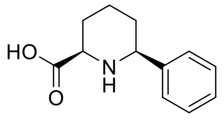

(2*R*,6*S*)-5a
$C_{12}H_{15}NO_2$
205.26 g · mol^{-1}

Deformylation product (**(2*R*,6*S*)-11a**, 0.69 g, 2.81 mmol, 1.0 equiv) was combined with LiOH in MeOH (20 mL) according to **GSP 4**. Product (**(2*R*,6*S*)-5a**, 0.42 g, 2.05 mmol, 83%) was obtained as colourless solid.

HRMS: *m*/*z* = found 206.1183 [M+H]$^+$, calculated 206.11756 [M+H]$^+$.

^{1}H NMR (600 MHz, DMSO-d_6): δ = 7.79 (d, ^3J = 7.3 Hz, 2H, Ar_{ortho}-H), 7.34 (t, ^3J = 7.6 Hz, ^3J = 7.6 Hz, 2H, Ar_{meta}-H), 7.28 (t, ^3J = 7.3 Hz, ^3J = 7.3 Hz, 1H, Ar_{para}-H), 3.78 (d, ^3J = 9.4 Hz, 1H, -C^6H-), 3.06 (d, ^3J = 11.6 Hz, 1H, -C^2H-), 2.01 (m, 1H, -CH_2-C^2H-), 1.84 (m, 1H, -CH_2-CH_2-CH_2-), 1.69 (m, 1H, -CH_2-C^6H-), 1.57 – 1.45 (m, 2H, -CH_2-CH_2-C^6H-), 1.39 (m, 1H, -CH_2-C^2H-).

^{13}C{^{1}H} NMR (151 MHz, DMSO-d_6): δ = 128.24 (Ar_{meta}-C), 127.43 (Ar_{para}-C), 127.04 (Ar_{ortho}-C), 61.16 (-C^2-), 60.42 (-C^6-), 32.42 (-CH_2-C^6H-), 27.48 (-CH_2-C^2H-), 24.51 (-CH_2-CH_2-CH_2-).

2*R*,6*S*)-6-(*p*-Tolyl)piperidine-2-carboxylic acid (**(2*R*,6*S*)-5b**)

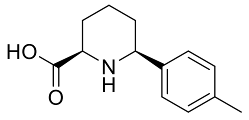

(2*R*,6*S*)-5b
$C_{13}H_{17}NO_2$
219.28 g · mol^{-1}

Deformylation product (**(2*R*,6*S*)-11b**, 0.62 g, 2.38 mmol, 1.0 equiv) was combined with LiOH in MeOH (20 mL) according to **GSP 4**. Product (**(2*R*,6*S*)-5b**, 0.25 g, 1.15 mmol, 79%) was obtained as colourless solid.

LC–MS: *m*/*z* = found 220.4127 [M+H]$^+$, calculated 220.1332 [M+H]$^+$.

^{1}H NMR (600 MHz, DMSO-d_6): δ = 7.51 (d, ^3J = 7.1 Hz, 2H, Ar_{ortho}-H), 7.30 (t, ^3J = 7.2 Hz, ^3J = 7.2 Hz, 2H, Ar_{meta}-H), 3.52 (d, ^3J = 9.4 Hz, 1H, -C^6H-), 3.01 (d, ^3J = 11.3 Hz, 1H, -C^2H-), 2.22 (s, 3H, -C-CH_3), 2.04 (m, 1H, -CH_2-C^2H-), 1.93 (m, 1H, -CH_2-CH_2-CH_2-), 1.77 (m, 1H, -CH_2-C^6H-), 1.57 – 1.44 (m, 2H, -CH_2-CH_2-C^6H-), 1.39 (m, 1H, -CH_2-C^2H-).

^{13}C{^{1}H} NMR (151 MHz, DMSO-d_6): δ = 138.81 (-C-CH_3), 134.67 (-C^6H-C-), 129.22 (Ar_{meta}-C), 128.44 (Ar_{ortho}-C), 60.83 (-C^2-), 58.31 (-C^6-), 33.79 (-CH_2-C^6H-), 28.68 (-CH_2-C^2H-), 25.22 (-CH_2-CH_2-CH_2-), 21.23 (-C-CH_3).

(2*R*,6*S*)-6-(3,5-Dimethylphenyl)piperidine-2-carboxylic acid (**(2*R*,6*S*)-5c**)

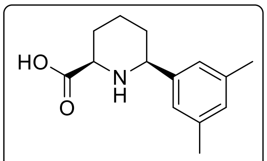

(2*R*,6*S*)-5c
$C_{14}H_{19}NO_2$
233.31 g · mol^{-1}

Deformylation product (**(2*R*,6*S*)-11c**, 0.58 g, 2.12 mmol, 1.0 equiv) was combined with LiOH in MeOH (20 mL) according to **GSP 4**. Product (**(2*R*,6*S*)-5c**, 0.23 g, 1.00 mmol, 83%) was obtained as colourless solid.

LC–MS: *m*/*z* = found 234.3518 [M+H]$^+$, calculated 234.1489 [M+H]$^+$.

^{1}H NMR (600 MHz, D_2O): δ = 7.28 – 7.19 (m, 2H, Ar_{ortho}-H), 6.99 (m, 1H, -C-CH-C-), 4.43 (m, 1H, -C^{6}H-), 4.33 (m, 1H, -C^{2}H-), 2.49 (m, 1H, -CH_2-C^{6}H-), 2.30 (s, 6H, -C-CH_3), 2.23 (m, 1H, -CH_2-C^{2}H-), 2.21 (m, 1H, -CH_2-CH_2-CH_2-), 2.14 (m, 1H, -CH_2-C^{6}H-), 2.11 – 1.98 (m, 2H, -CH_2-CH_2-C^{2}H-).

^{13}C{^{1}H} NMR (151 MHz, D_2O): δ = 164.51 (-COO-), 139.82 (-C^{6}H-C-), 138.69 (-C-CH_3), 131.75 (-C-CH-C-), 126.76 (Ar_{ortho}-C), 62.43 (-C^6-), 59.08 (-C^2-), 27.95 (-CH_2-C^{6}H-), 23.76 (-CH_2-C^{2}H-), 22.26 (-CH_2-CH_2-CH_2-), 21.07 (-C-CH_3).

(2*R*,6*S*)-6-(4-(Trifluoromethyl)phenyl)piperidine-2-carboxylic acid (**(2*R*,6*S*)-5d**)

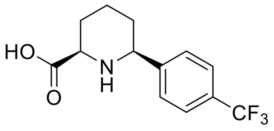

(2*R*,6*S*)-5d
$C_{13}H_{14}F_3NO_2$
273.26 g · mol^{-1}

Deformylation product (**(2*R*,6*S*)-11d**, 0.93 g, 2.96 mmol, 1.0 equiv) was combined with LiOH in MeOH (20 mL) according to **GSP 4**. Product (**(2*R*,6*S*)-5d**, 0.63 g, 2.29 mmol, 87%) was obtained as colourless solid.

LC–MS: *m*/*z* = found 274.1348 [M+H]$^+$, calculated 274.1049 [M+H]$^+$.

^{1}H NMR (600 MHz, D_2O): δ = 7.83 (d, ^3J = 8.2 Hz, 2H, Ar_{meta}-H), 7.68 (d, ^3J = 8.1 Hz, 2H, Ar_{ortho}-H), 4.45 (d, ^3J = 12.7 Hz, 1H, -C^{6}H-), 4.06 (m, 1H, -C^{2}H-), 2.42 (m, 1H, -CH_2-C^{2}H-), 2.21 – 2.11 (m, 2H, -CH_2-CH_2-C^{6}H-), 2.02 (m, 1H, -CH_2-C^{6}H-), 1.91 – 1.78 (m, 2H, -CH_2-CH_2-C^{2}H-).

^{13}C{^{1}H} NMR (151 MHz, D_2O): δ = 172.27 (-COO-), 139.99 (-C^{6}H-C-), 130.74 (q, ^2J = 30.0 Hz, -C-CF_3), 127.74 (Ar_{ortho}-C), 126.17 (q, ^3J = 3.8 Hz, Ar_{meta}-C), 123.86 (q, ^1J = 271.4 Hz, -CF_3), 60.24 (-C^6-), 59.44 (-C^2-), 28.78 (-CH_2-C^{6}H-), 25.46 (-CH_2-C^{2}H-), 22.52 (-CH_2-CH_2-CH_2-).

^{19}F{^{13}C} NMR (565 MHz, D_2O) δ = −62.65.

(2*R*,6*S*)-6-(4-Methoxyphenyl)piperidine-2-carboxylic acid (**(2*R*,6*S*)-5e**)

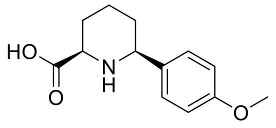

(2*R*,6*S*)-5e
$C_{13}H_{17}NO_3$
235.28 g · mol^{-1}

Hydrogenation product (**(2*R*,6*S*)-9e**, 0.35 g, 1.26 mmol, 1.0 equiv) was combined with first HCl and later LiOH in MeOH (20 mL) according to **GSP 4**. Product (**(2*R*,6*S*)-5e**, 0.19 g, 0.80 mmol, 64%) was obtained as colourless solid.

LC–MS: *m*/*z* = found 236.1342 $[M+H]^+$, calculated 236.1281 $[M+H]^+$.

^{1}H NMR (500 MHz, D_2O): δ = 7.46 (d, ^3J = 8.8 Hz, 2H, Ar_{ortho}-H), 7.07 (d, ^3J = 8.8 Hz, 2H, Ar_{meta}-H), 4.27 (dd, ^3J = 11.9 Hz, ^3J = 3.5 Hz, 1H, -C^6H-), 3.88 (d, ^3J = 12.5 Hz, ^3J = 3.4 Hz, 1H, -C^2H-), 3.85 (s, 3H, -CH_3), 2.36 (m, 1H, -CH_2-C^2H-), 2.11 (m, 1H, -CH_2-C^6H-), 2.07 – 1.95 (m, 2H, -CH_2-CH_2-C^6H-), 1.85 – 1.70 (m, 2H, -CH_2-CH_2-C^2H-).

^{13}C{^{1}H} NMR (126 MHz, D_2O): δ = 173.27 (-COO-), 159.54 (-C-OCH_3), 128.88 (Ar_{ortho}-C), 128.75 (-C^6H-C-), 114.55 (Ar_{meta}-C), 60.19 (-C^6-), 60.03 (-C^2-), 55.38 (-C-OCH_3), 28.62 (-CH_2-C^6H-), 25.74 (-CH_2-C^2H-), 22.78 (-CH_2-CH_2-CH_2-).

(2*R*,6*S*)-6-(4-(Hydroxymethyl)phenyl)piperidine-2-carboxylic acid (**(2*R*,6*S*)-5f**)

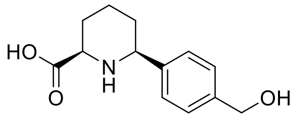

(2*R*,6*S*)-5f
$C_{13}H_{17}NO_3$
235.28 g · mol^{-1}

Deformylation product (**(2*R*,6*S*)-11f**, 0.47 g, 1.68 mmol, 1.0 equiv) was combined with LiOH in MeOH (20 mL) according to **GSP 4**. Product (**(2*R*,6*S*)-5f**, 0.34 g, 1.43 mmol, 91%) was obtained as colourless solid.

HRMS: *m*/*z* = found 236.1288 $[M+H]^+$, calculated 236.12812 $[M+H]^+$.

^{1}H NMR (600 MHz, $CDCl_3$): δ = 7.35 (d, ^3J = 7.9 Hz, 2H, Ar_{ortho}-H), 7.17 (d, ^3J = 7.5 Hz, 2H, Ar_{meta}-H), 3.93 (m, 1H, -C^6H-), 3.16 (m, 1H, -C^2H-), 2.34 (s, 2H, -CH_2-OH), 2.15 – 1.99 (m, 2H, -CH_2-CH_2-C^2H-), 1.96 – 1.85 (m, 2H, -CH_2-C^6H-), 1.75 (m, 1H, -CH_2-C^2H-), 1.48 (m, 1H, -CH_2-CH_2-CH_2-).

^{13}C{^{1}H} NMR (151 MHz, $CDCl_3$): δ = 171.07 (-COO-), 139.53 (-C-CH_2-OH), 132.88 (-C^6H-C-), 129.65 (Ar_{meta}-C), 128.55 (Ar_{ortho}-C), 61.87 (-C^6-), 58.42 (-C^2-), 28.98 (-CH_2-C^6H-), 24.51 (-CH_2-C^2H-), 23.18 (-CH_2-CH_2-CH_2-), 21.25 (-CH_2-OH).

(2*R*,6*S*)-6-(3-Aminophenyl)piperidine-2-carboxylic acid (**(2*R*,6*S*)-5h**)

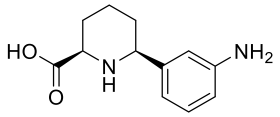

(2*R*,6*S*)-5h
$C_{12}H_{16}N_2O_2$
220.27 g · mol^{-1}

Deformylation product (**(2*R*,6*S*)-11h**, 0.12 g, 0.46 mmol, 1.0 equiv) was combined with LiOH in MeOH (20 mL) according to **GSP 4**. Product (**(2*R*,6*S*)-5h**, 40.7 mg, 0.18 mmol, 70%) was obtained as colourless solid.

HRMS: *m*/*z* = found 221.1283 $[M+H]^+$, calculated 221.12845 $[M+H]^+$.

^{1}H NMR (600 MHz, DMSO-d_6): δ = 6.91 (dd, ^3J = 7.7 Hz, ^3J = 7.7 Hz, 1H, Ar_{meta}-H), 6.60 (t, ^4J = 2.0 Hz, ^4J = 2.0 Hz, 1H, -C-CH-C-), 6.50 (dd, ^3J = 7.5 Hz, ^4J = 1.4 Hz, 1H, Ar_{ortho}-H), 6.51 (ddd, ^3J = 7.9 Hz, ^4J = 2.3 Hz, ^4J = 1.0 Hz, 1H, Ar_{para}-H), 3.41 (m, 1H, -C^6H-), 2.88 (dd, ^3J = 11.5 Hz, ^3J = 2.7 Hz, 1H, -C^2H-), 1.91 (m, 1H, -CH_2-C^2H-), 1.79 (m, 1H, -CH_2-CH_2-CH_2-), 1. (m, 1H, -CH_2-C^6H-), 1.40 (m, 1H, -CH_2-CH_2-CH_2-), 1.57 – 1.45 (m, 2H, -CH_2-CH_2-CH_2-).

^{13}C{^{1}H} NMR (151 MHz, DMSO-d_6): δ = 175.90 (-COO-), 148.45 (-C-NH_2), 146.23 (-C^6H-C-), 128.49 (Ar_{meta}-C), 114.37 (Ar_{ortho}-C), 112.46 (-C-CH-C-), 112.37 (Ar_{para}-C), 62.13 (-C^2-), 61.67 (-C^6-), 34.40 (-CH_2-C^6H-), 28.89 (-CH_2-C^2H-), 25.44 (-CH_2-CH_2-CH_2-).

(2*R*,6*S*)-6-(Phenylethynyl)piperidine-2-carboxylic acid (**(2*R*,6*S*)-6**)

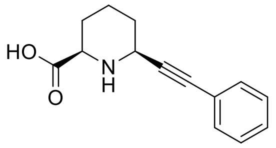

(2*R*,6*S*)-6
$C_{14}H_{15}NO_2$
229.28 g · mol-**1**

Hydrogenation product (**(2*R*,6*S*)-13**, 67.7 mg, 0.25 mmol, 1.0 equiv) was combined with first HCl and later LiOH in MeOH (20 mL) according to **GSP 4**. Product (**(2*R*,6*S*)-6**, 49.8 mg, 0.22 mmol, 87%) was obtained as colourless solid.

LC–MS: *m*/*z* = found 230.1271 $[M+H]^+$, calculated 223.1176 $[M+H]^+$.

^{1}H NMR (600 MHz, DMSO-d_6): δ = 7.40 (dd, ^3J = 6.8 Hz, ^4J = 3.0 Hz, 2H, Ar_{ortho}-$\underline{H}$), 7.37 – 7.33 (m, 3H, $Ar_{meta/para}$-$\underline{H}$), 4.11 (dd, ^3J = 4.2 Hz, ^3J = 4.2 Hz, 1H, -$C^6\underline{H}$-), 3.17 (dd, ^3J = 9.8 Hz, ^3J = 3.1 Hz, 1H, -$C^2\underline{H}$-), 1.82 (m, 1H, -$C\underline{H}_2$-C^2H-), 1.68 – 1.53 (m, 4H, -$C\underline{H}_2$-$C\underline{H}_2$-C^2H-), 1.34 (m, 1H, -$C\underline{H}_2$-C^2H-).

^{13}C{^{1}H} NMR (151 MHz, DMSO-d_6): δ = 176.56 (-$\underline{C}$OO-), 131.72 (Ar_{ortho}-$\underline{C}$), 129.05 (Ar_{meta}-$\underline{C}$), 128.49 (Ar_{para}-$\underline{C}$), 123.47 (Ar_{ipso}-$\underline{C}$), 92.63 (-C^6H-$\underline{C}$≡C-), 83.59 (-C^6H-C≡$\underline{C}$-), 56.31 (-$\underline{C}^2$-), 45.65 (-$\underline{C}^6$-), 31.15 (-$\underline{C}H_2$-C^6H-), 29.31 (-$\underline{C}H_2$-C^2H-), 21.77 (-CH_2-$\underline{C}H_2$-CH_2-).

(2*R*,6*S*)-1-formyl-6-phenylpiperidine-2-carboxylic acid (**(2*R*,6*S*)-10a**)

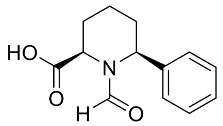

(2*R*,6*S*)-10a
$C_{13}H_{15}NO_3$
233.27 g · mol^{-1}

Hydrogenation product (**(2*R*,6*S*)-9a**, 89.0 mg, 0.36 mmol, 1.0 equiv) was dissolved in MeOH (2.5 mL) and water (2.5 mL). LiOH (43.1 mg, 1.5.0 equiv) was added and the resulting mixture was stirred at 40 °C for 1 h. Afterwards the reaction was frozen and lyophilized. The resulting crude was suspended in DMF (2 mL) and filtered through a pad of Celite, washed with DMF (2 mL) and concentrated under reduced pressure. Purification was done by PR-HPLC to obtain product (**(2*R*,6*S*)-10a**, 76.4 mg, 0.33 mmol, 91%).

LC–MS: *m*/*z* = found 234.1233 [M+H]$^+$, calculated 234.1125 [M+H]$^+$.

^{1}H NMR (**(2*R*,6*S*)-10a** chair, 600 MHz, $CDCl_3$): *δ* = 8.20 (s, 1H, -CHO), 7.41 – 7.33 (m, 4H, Ar$_{ortho/meta}$-H), 7.29 (m, 1H, Ar$_{para}$-H), 4.87 (dd, ^3J = 5.3 Hz, ^3J = 5.3 Hz, 1H, -C^{2}H-), 4.78 (dd, ^3J = 7.2 Hz, ^3J = 4.4 Hz, 1H, -C^{6}H-), 2.26 (m, 1H, -CH$_2$-C^{6}H-), 2.20 (m, 1H, -CH$_2$-C^{2}H-), 2.02 – 1.93 (m, 2H, -CH$_2$-CH$_2$-C^{6}H-), 1.89 (m, 1H, -CH$_2$-C^{2}H-), 1.75 (m, 1H, -CH$_2$-CH$_2$-CH$_2$-).

^{1}H NMR (**(2*R*,6*S*)-10a** half-chair, 600 MHz, $CDCl_3$): *δ* = 8.36 (s, 1H, -CHO), 7.41 – 7.33 (m, 2H, Ar$_{ortho}$-H), 7.23 (t, ^3J = 7.6 Hz, ^3J = 7.6 Hz, 2H, Ar$_{meta}$-H), 7.16 (t, ^3J = 7.1 Hz, ^3J = 7.1 Hz, 1H, Ar$_{para}$-H), 5.77 (d, ^3J = 5.8 Hz, 1H, -C^{6}H-), 4.23 (d, ^3J = 4.9 Hz, 1H, -C^{2}H-), 2.44 (m, 1H, -CH$_2$-C^{2}H-), 2.26 (m, 1H, -CH$_2$-C^{6}H-), 1.97 (m, 1H, -CH$_2$-CH$_2$-CH$_2$-), 1.89 (m, 1H, -CH$_2$-C^{6}H-), 1.80 – 1.69 (m, 2H, -CH$_2$-CH$_2$-C^{2}H-).

^{13}C{^{1}H} NMR (**(2*R*,6*S*)-10a** chair, 151 MHz, $CDCl_3$): *δ* = 174.52 (-COO-), 165.29 (-CHO), 139.68 (-C^{6}H-C-), 129.03 (Ar$_{meta}$-C), 128.21 (Ar$_{para}$-C), 127.37 (Ar$_{ortho}$-C), 57.57 (-C^6-), 51.25 (-C^2-), 29.37 (-CH$_2$-C^{6}H-), 24.25 (-CH$_2$-C^{2}H-), 17.56 (-CH$_2$-CH$_2$-CH$_2$-).

^{13}C{^{1}H} NMR (**(2*R*,6*S*)-10a** half-chair, 151 MHz, $CDCl_3$): *δ* = 173.89 (-COO-), 164.84 (-CHO), 138.23 (-C^{6}H-C-), 128.15 (Ar$_{meta}$-C), 128.06 (Ar$_{ortho}$-C), 127.41 (Ar$_{para}$-C), 54.74 (-C^2-), 49.84 (-C^6-), 25.78 (-CH$_2$-C^{6}H-), 25.66 (-CH$_2$-C^{2}H-), 17.19 (-CH$_2$-CH$_2$-CH$_2$-).

Assigned NMR Spectra

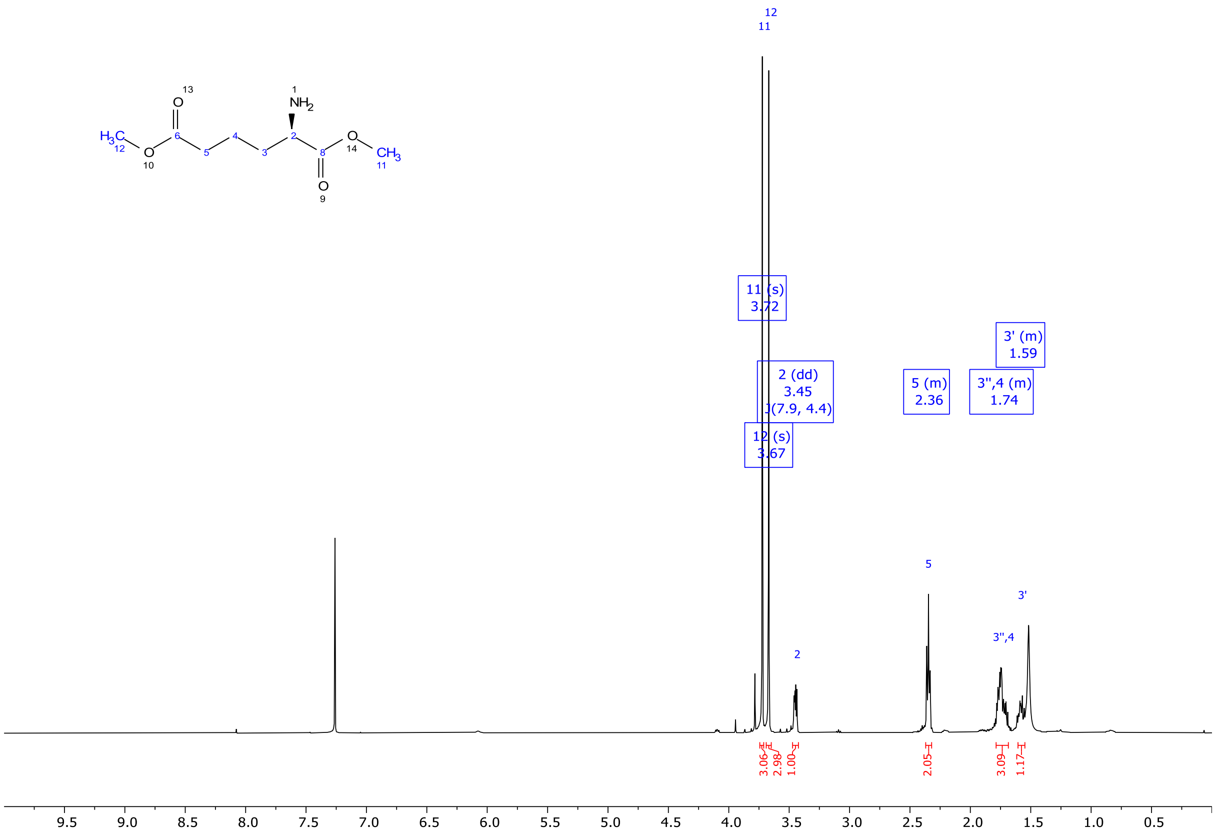

Figure S2: Assigned ^{1}H NMR (500 MHz, $CDCl_3$) of **H-D-Aad(OMe)-OMe**.

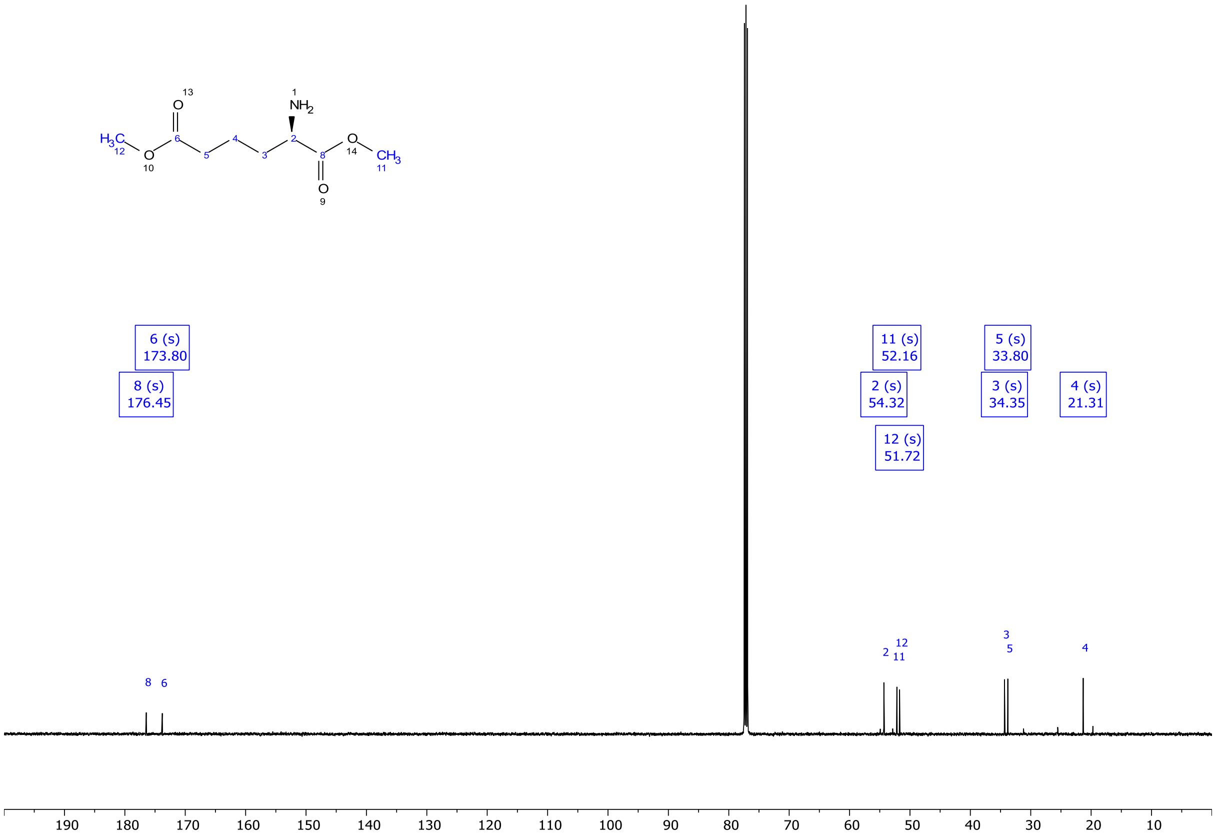

Figure S3: Assigned $^{13}C\{^{1}H\}$ NMR (126 MHz, $CDCl_3$) of **H-D-Aad(OMe)-OMe**.

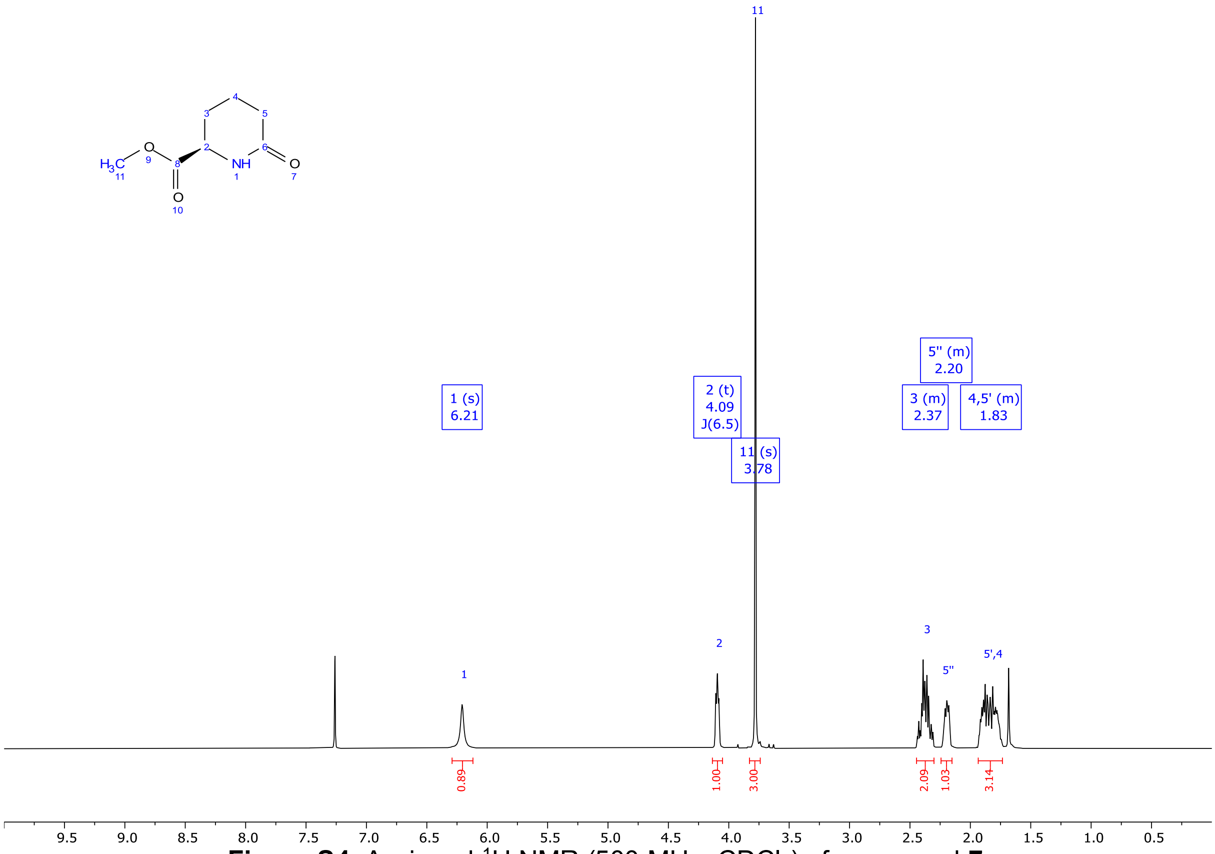

Figure S4: Assigned ^{1}H NMR (500 MHz, $CDCl_3$) of compound **7**.

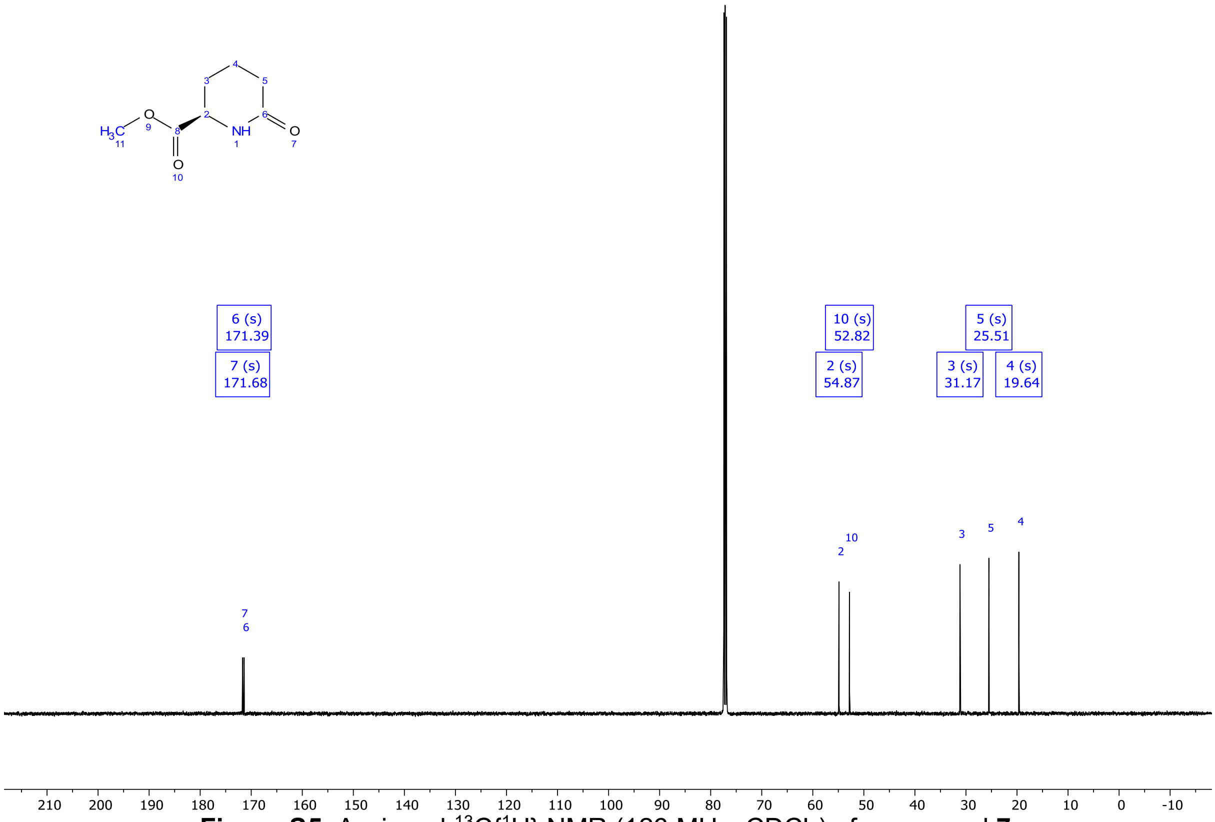

Figure S5: Assigned $^{13}C\{^{1}H\}$ NMR (126 MHz, $CDCl_3$) of compound **7**.

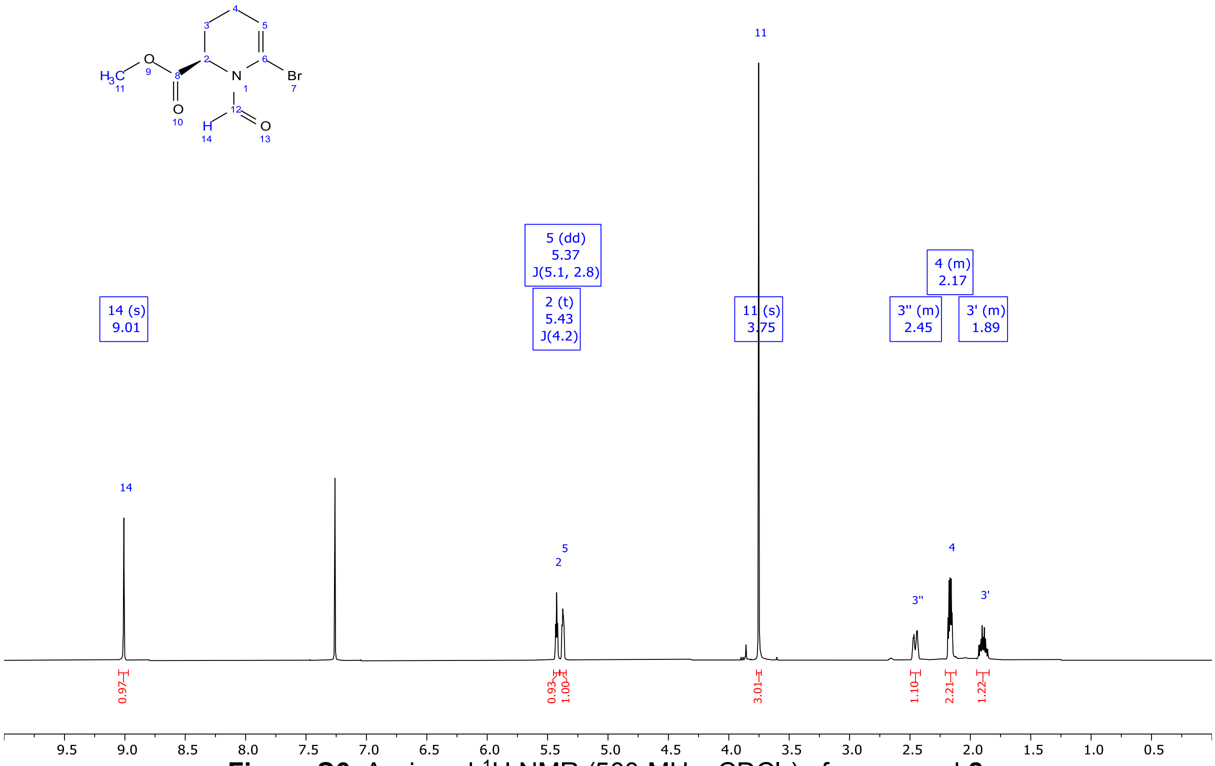

Figure S6: Assigned ^{1}H NMR (500 MHz, $CDCl_3$) of compound **2**.

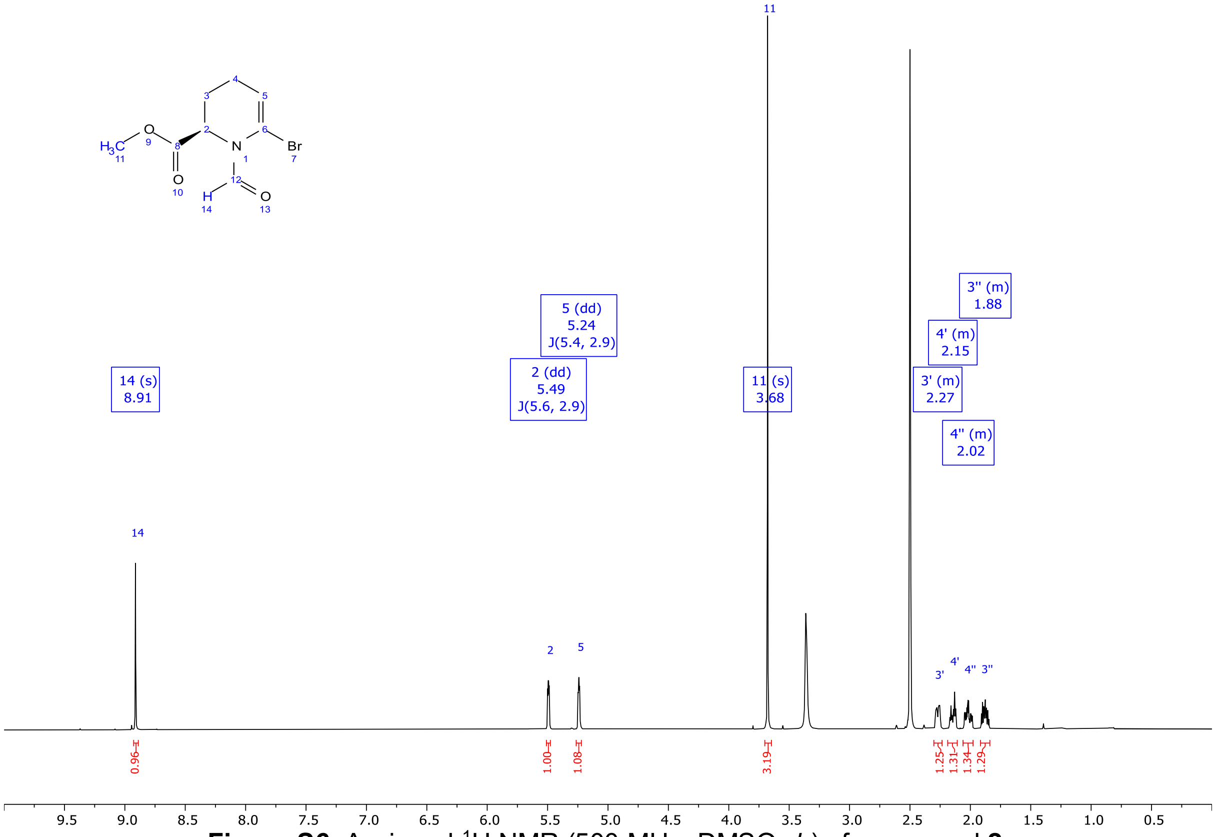

Figure S6: Assigned ^{1}H NMR (500 MHz, DMSO-d_6) of compound **2**.

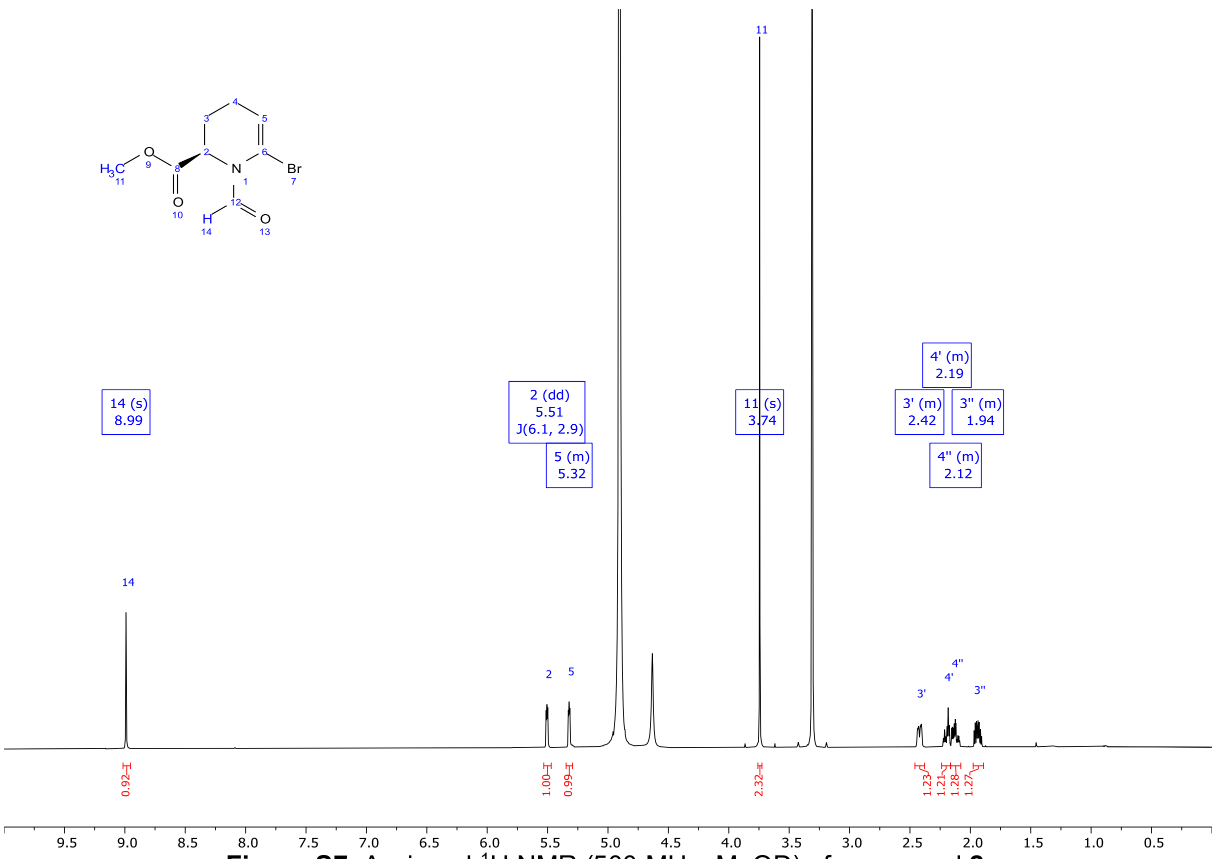

Figure S7: Assigned ^{1}H NMR (500 MHz, MeOD) of compound **2**.

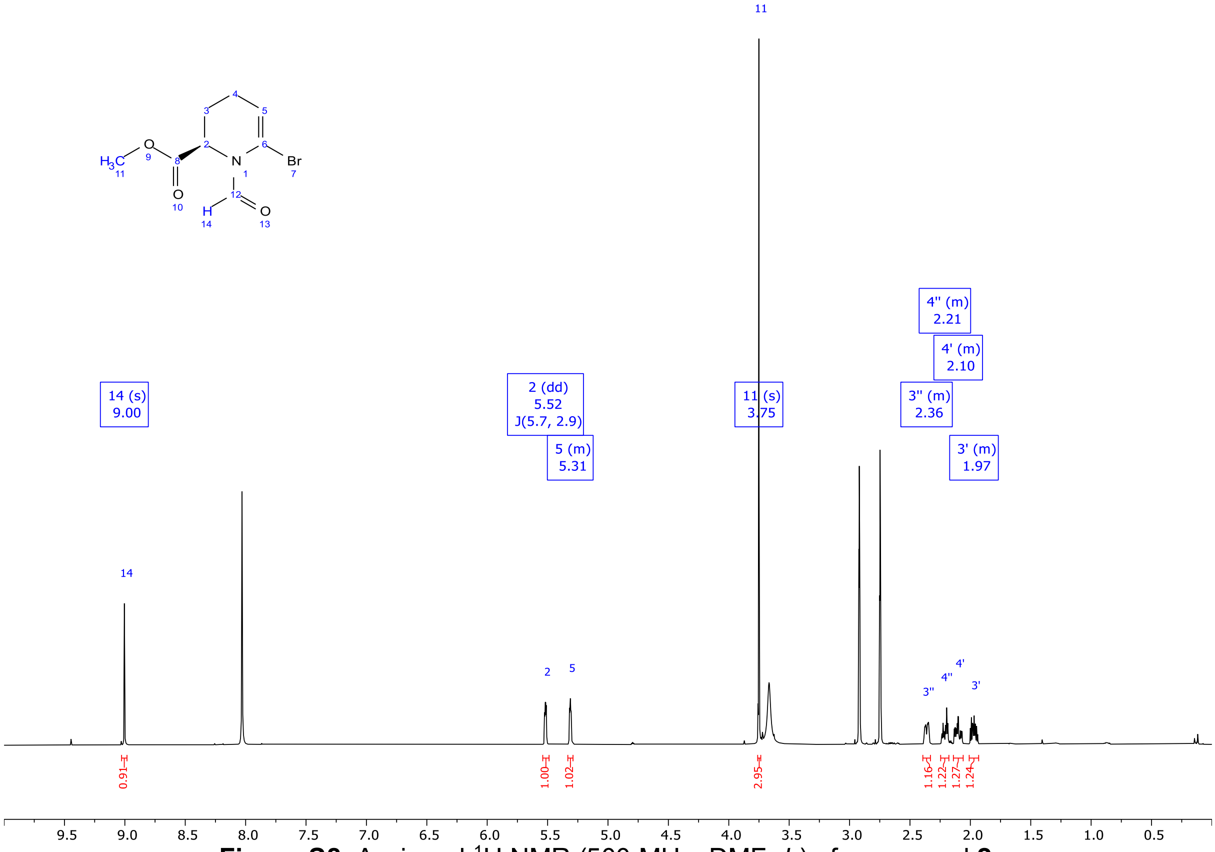

Figure S8: Assigned ^{1}H NMR (500 MHz, DMF-d_7) of compound **2**.

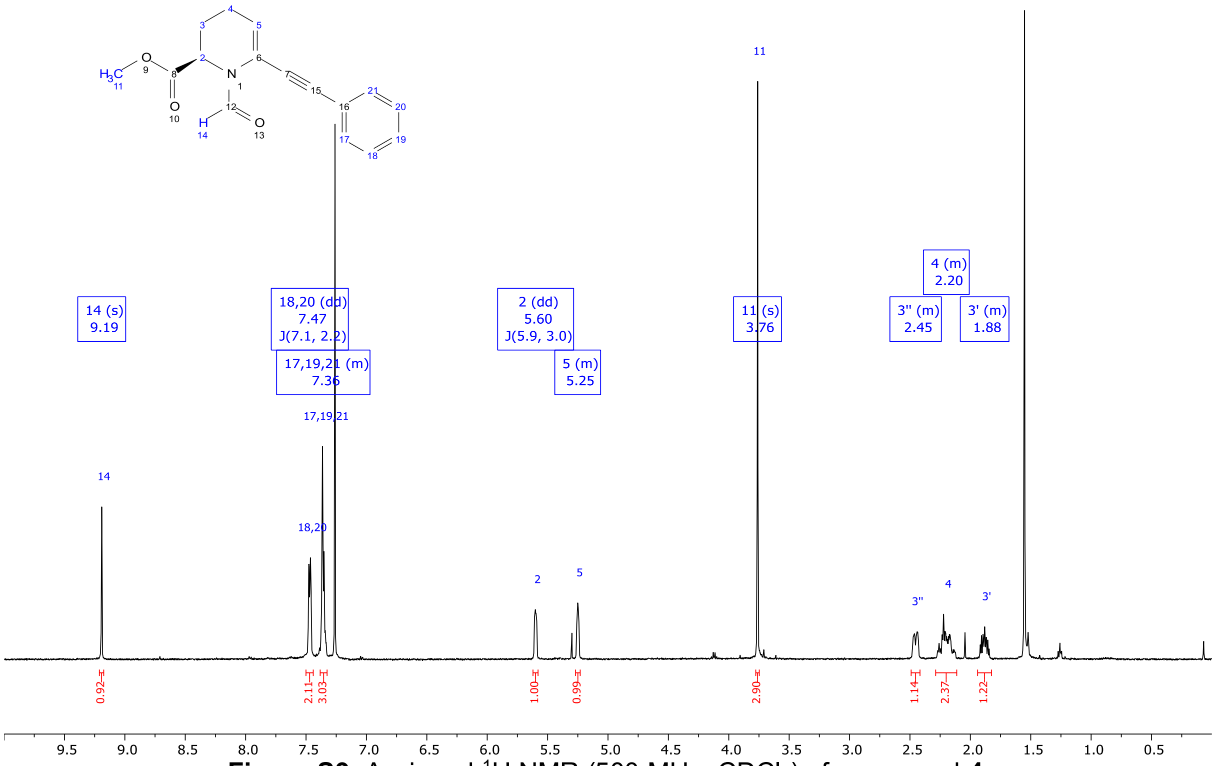

Figure S9: Assigned ^{1}H NMR (500 MHz, $CDCl_3$) of compound **4**.

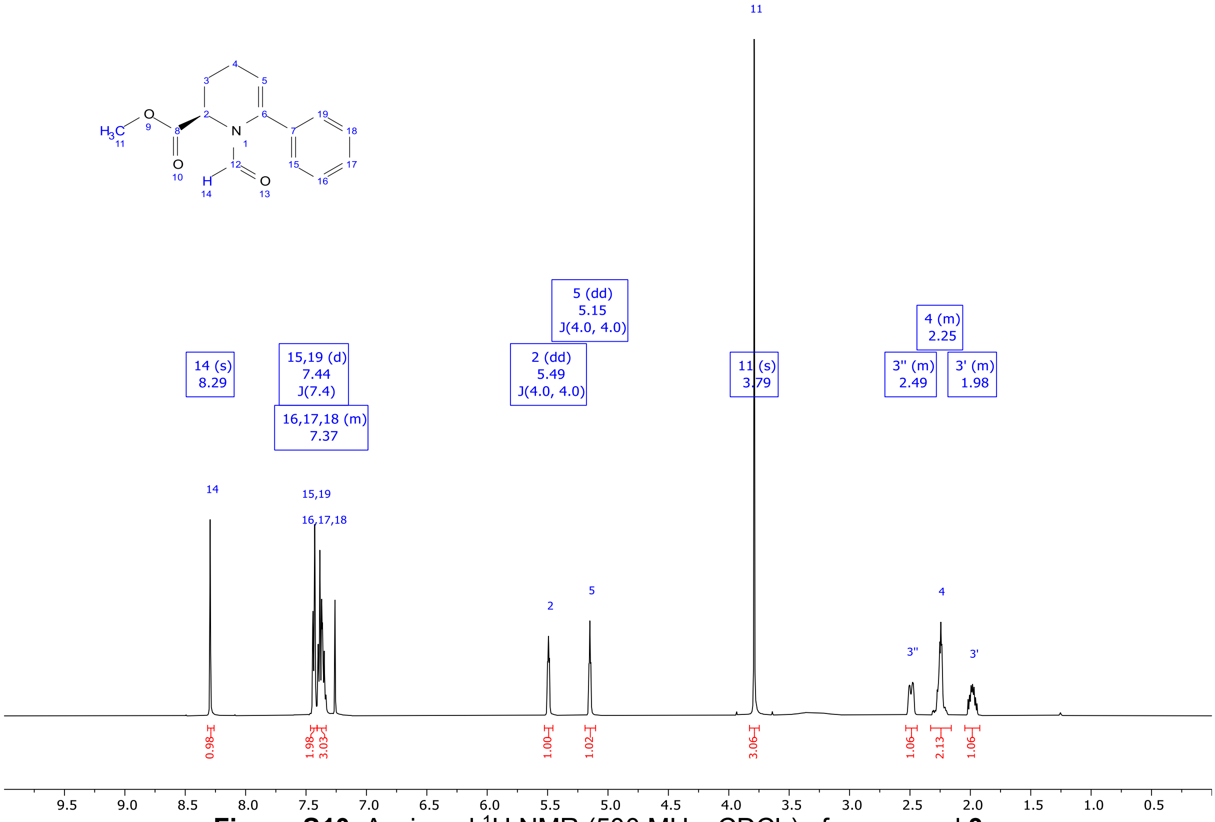

Figure S10: Assigned ^{1}H NMR (500 MHz, $CDCl_3$) of compound **3a**.

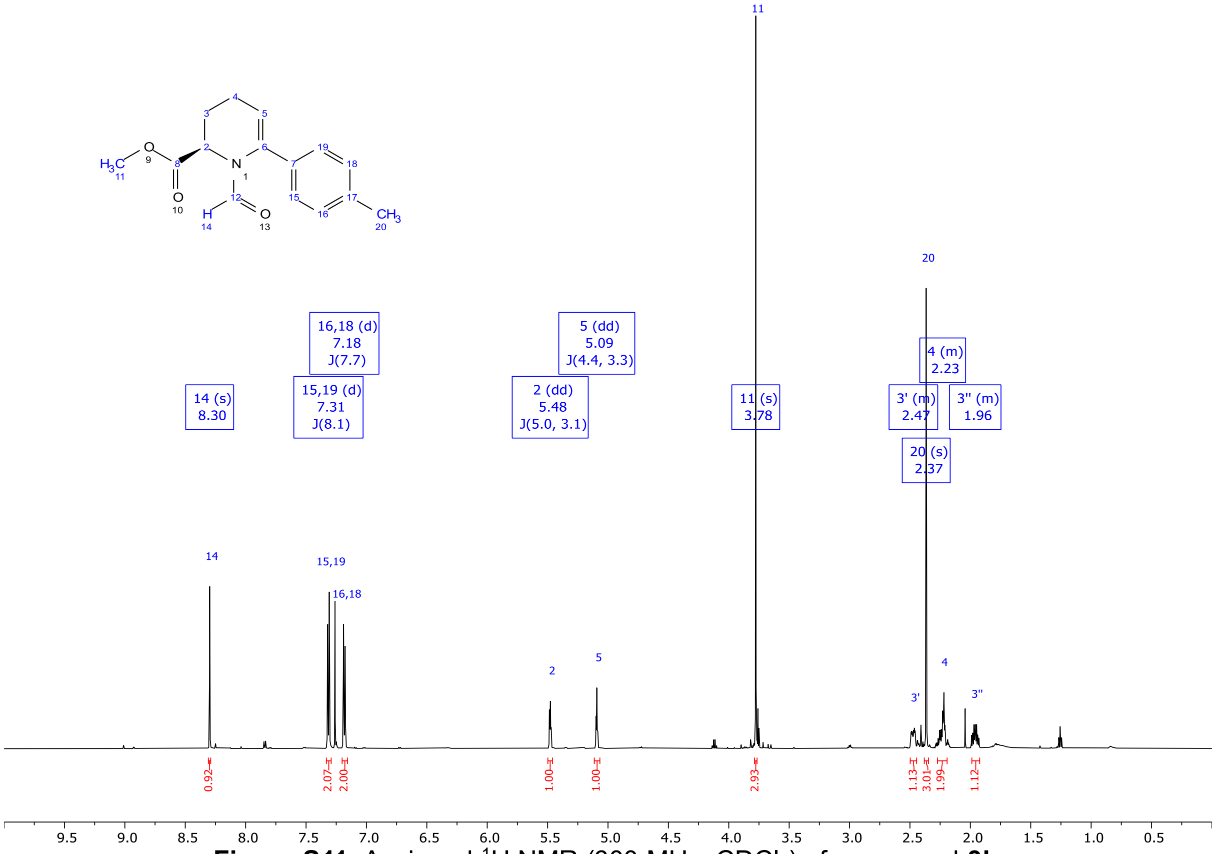

Figure S11: Assigned ^{1}H NMR (600 MHz, $CDCl_3$) of compound **3b**.

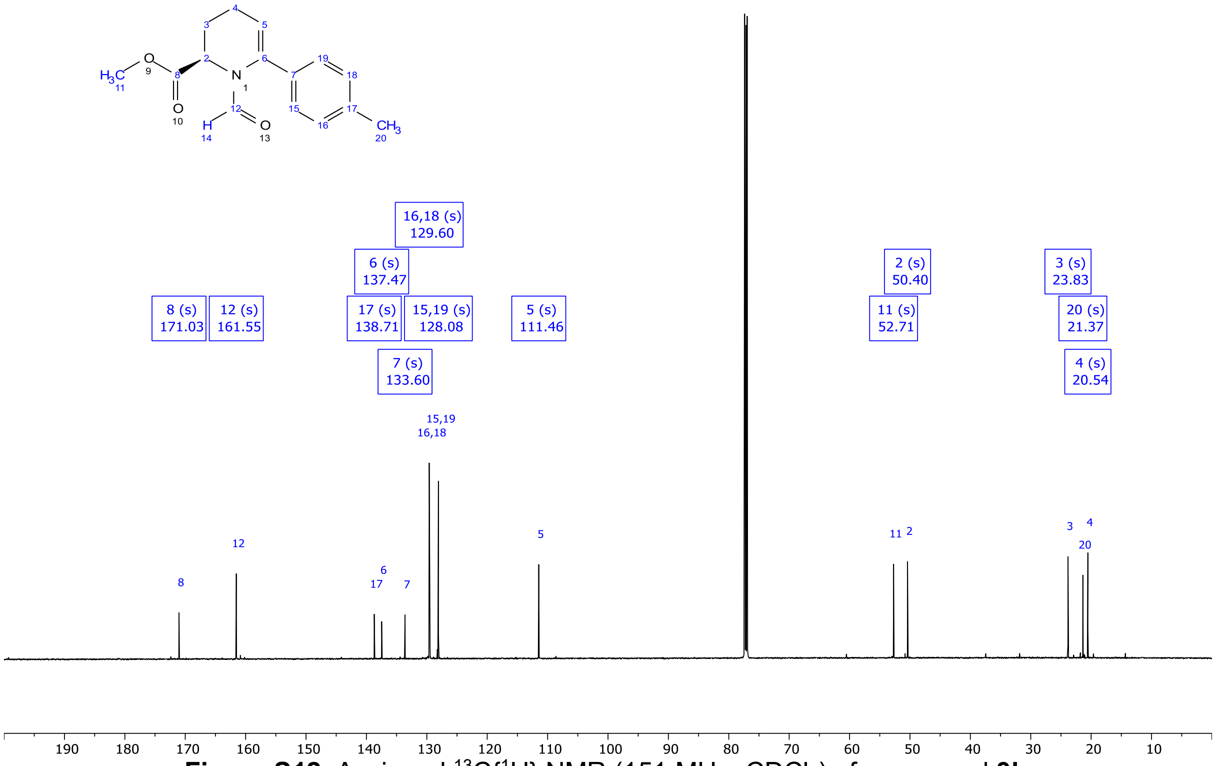

Figure S12: Assigned $^{13}C\{^{1}H\}$ NMR (151 MHz, $CDCl_3$) of compound **3b**.

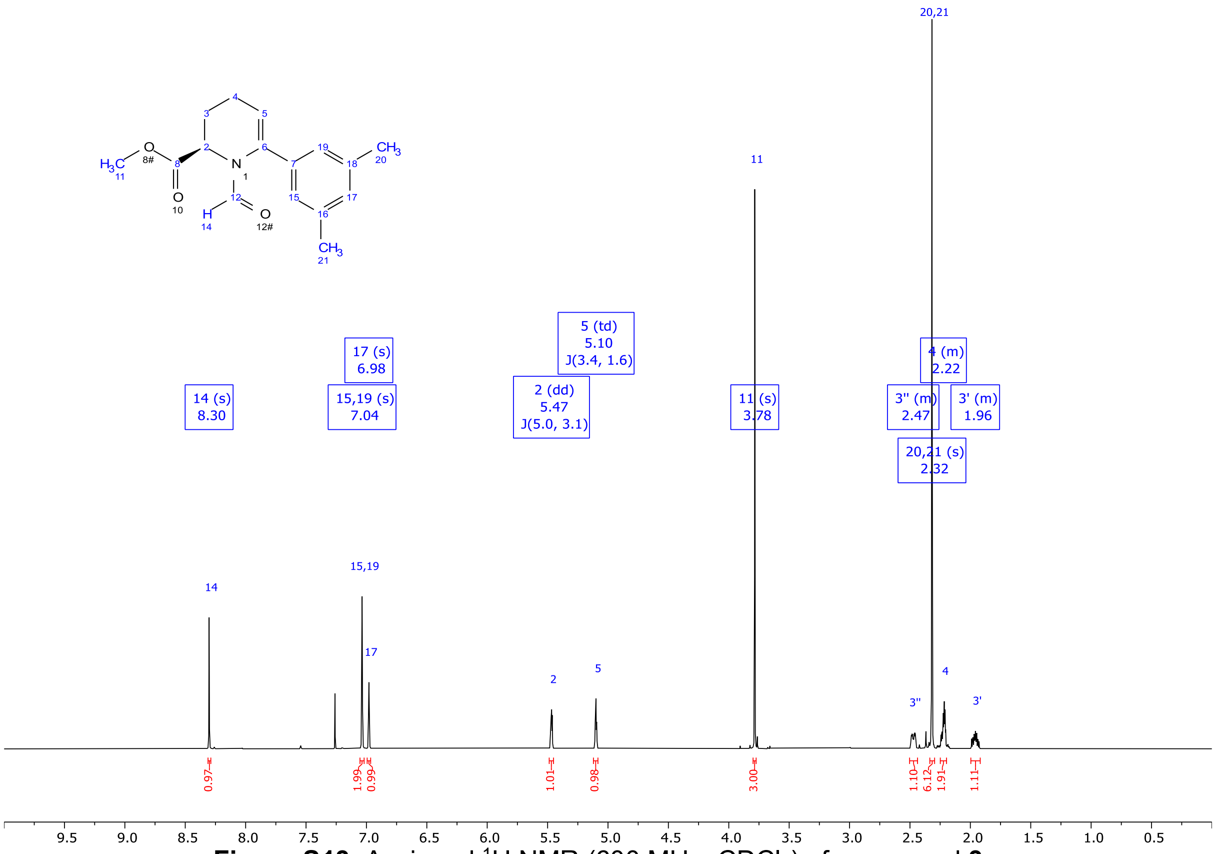

Figure S13: Assigned ^{1}H NMR (600 MHz, $CDCl_3$) of compound **3c**.

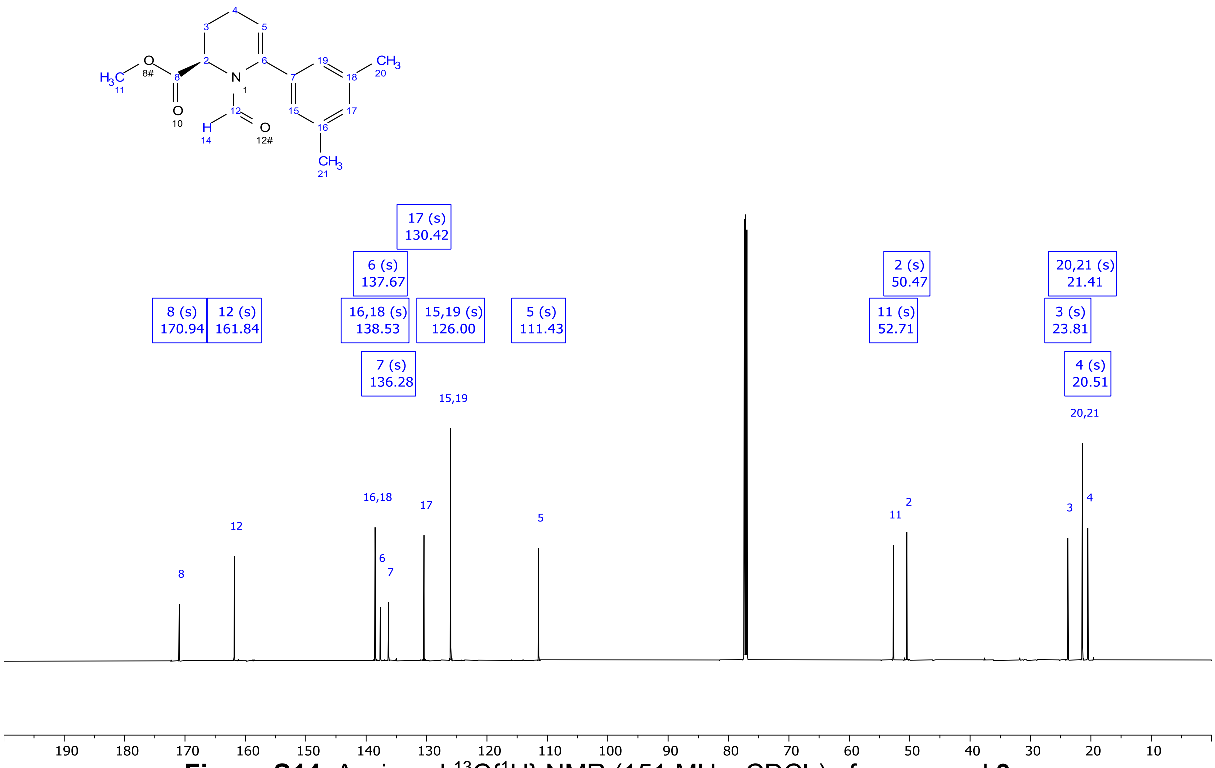

Figure S14: Assigned $^{13}C\{^{1}H\}$ NMR (151 MHz, $CDCl_3$) of compound **3c**.

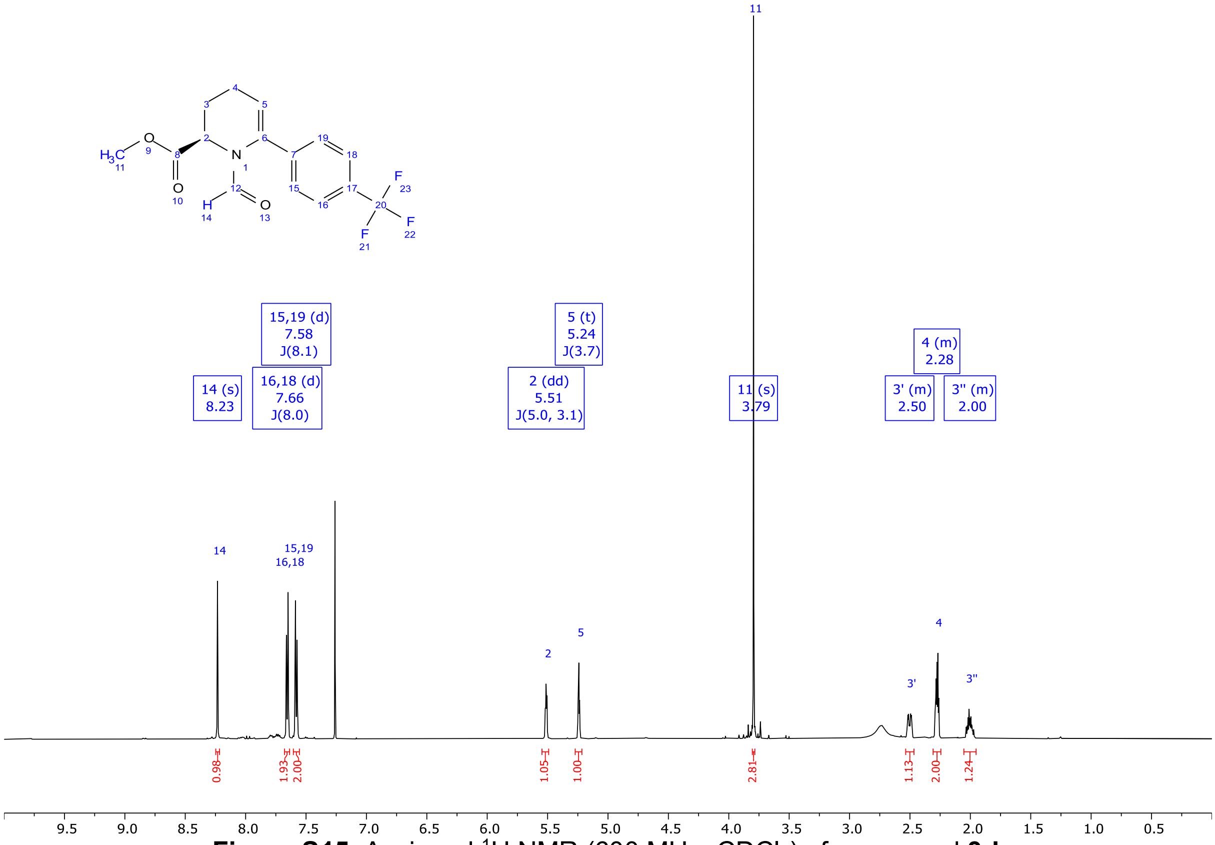

Figure S15: Assigned ^{1}H NMR (600 MHz, $CDCl_3$) of compound **3d**.

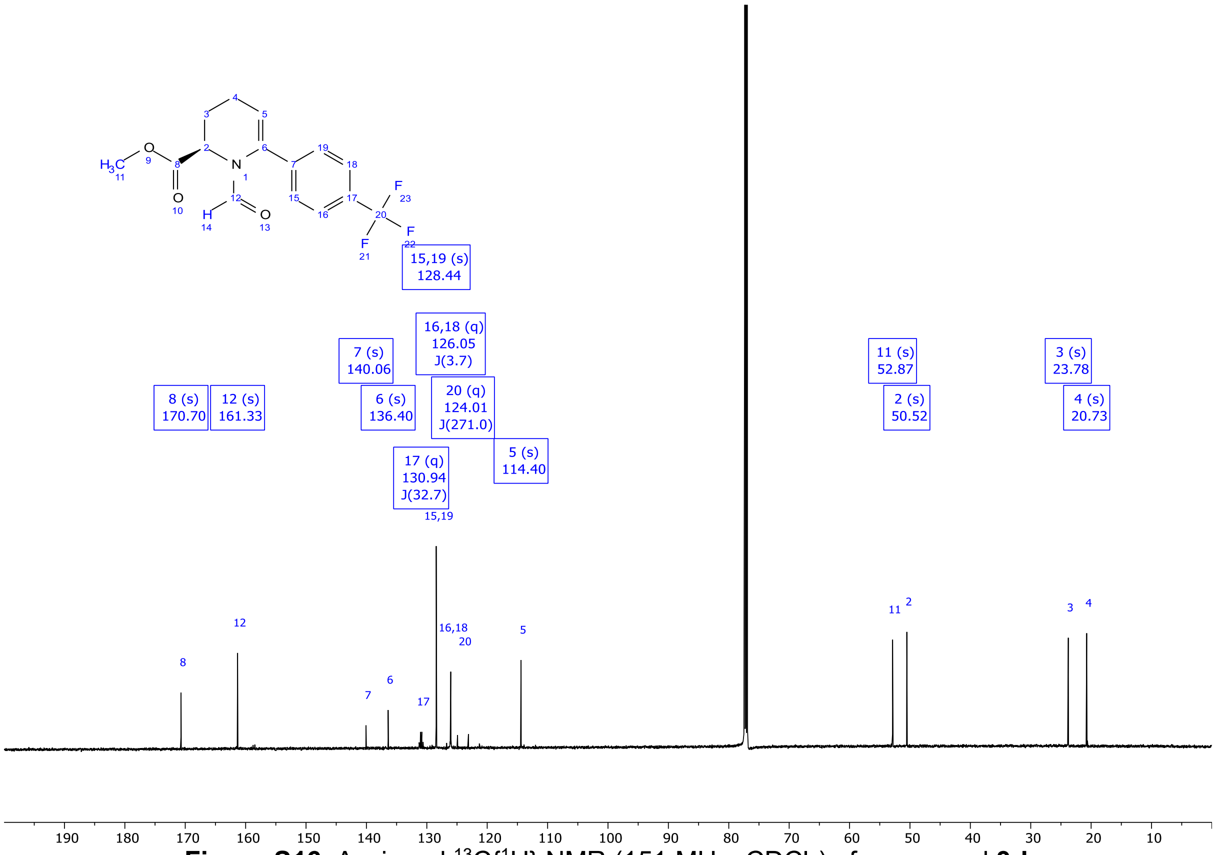

Figure S16: Assigned $^{13}C\{^{1}H\}$ NMR (151 MHz, $CDCl_3$) of compound **3d**.

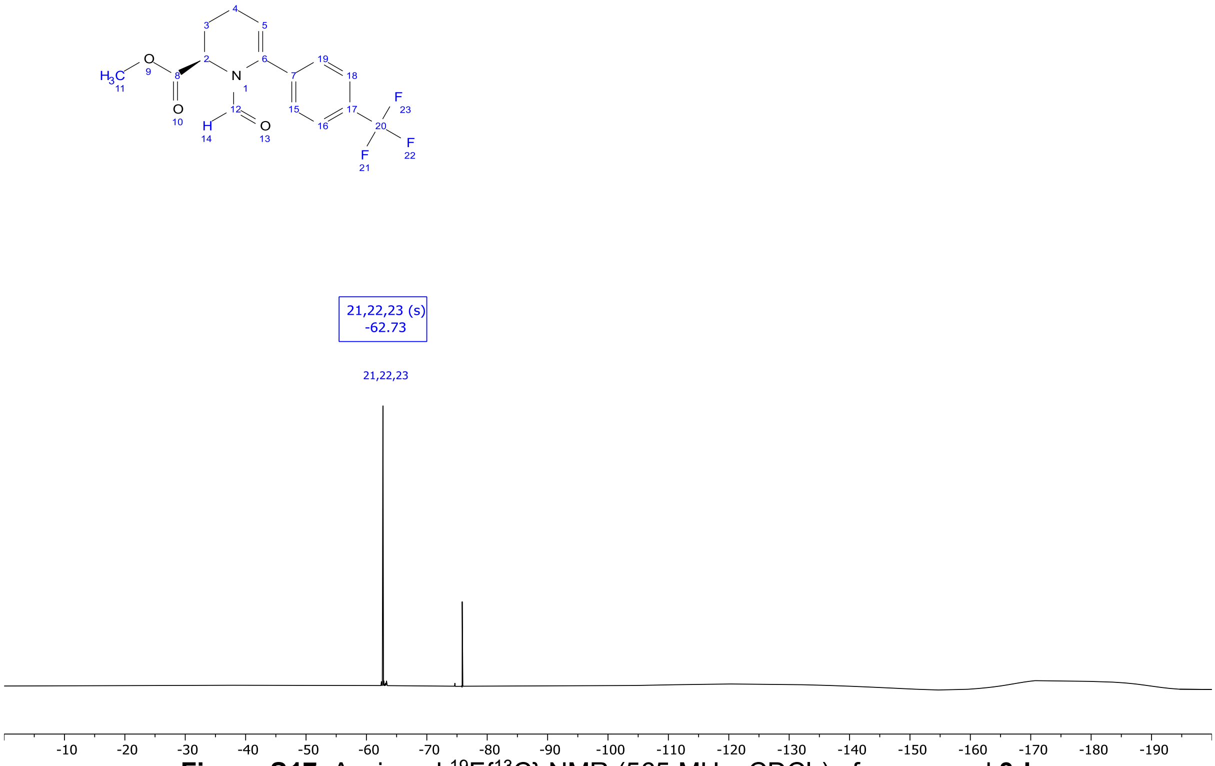

Figure S17: Assigned $^{19}F\{^{13}C\}$ NMR (565 MHz, $CDCl_3$) of compound **3d**.

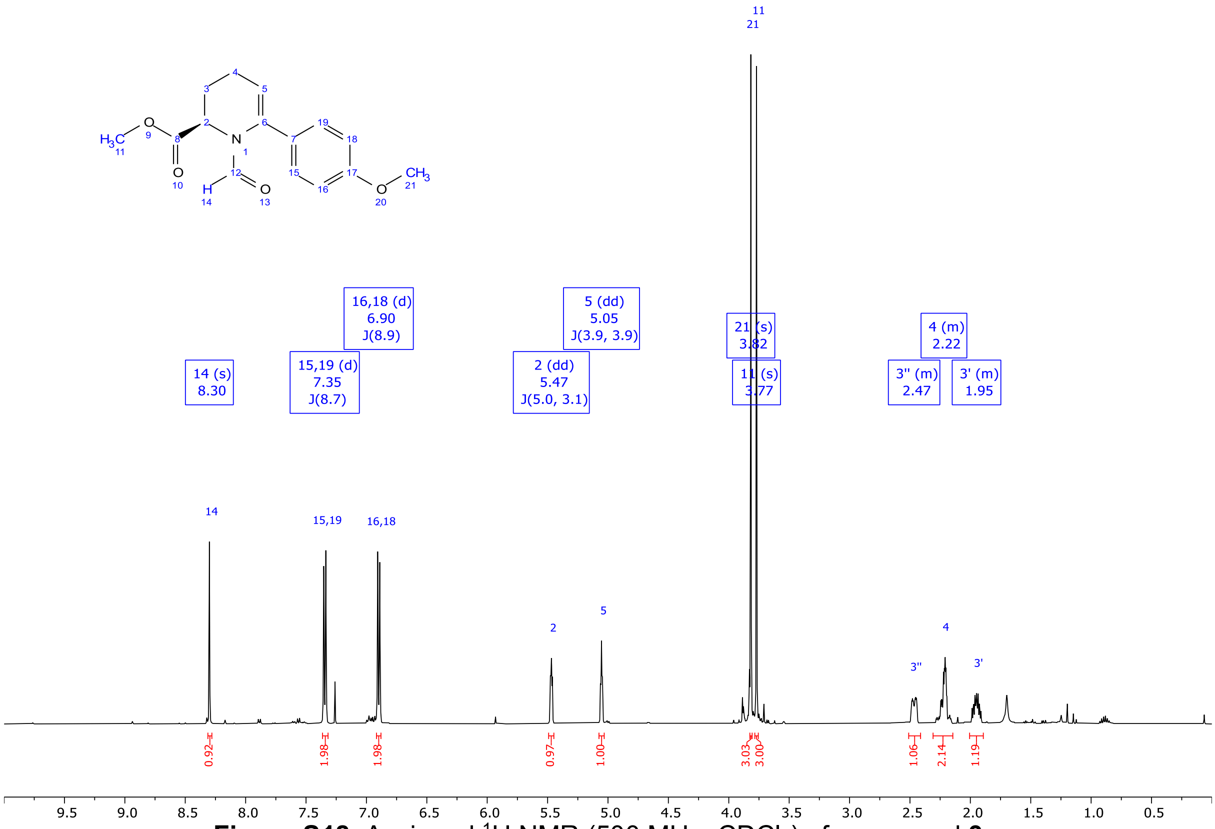

Figure S18: Assigned ^{1}H NMR (500 MHz, $CDCl_3$) of compound **3e**.

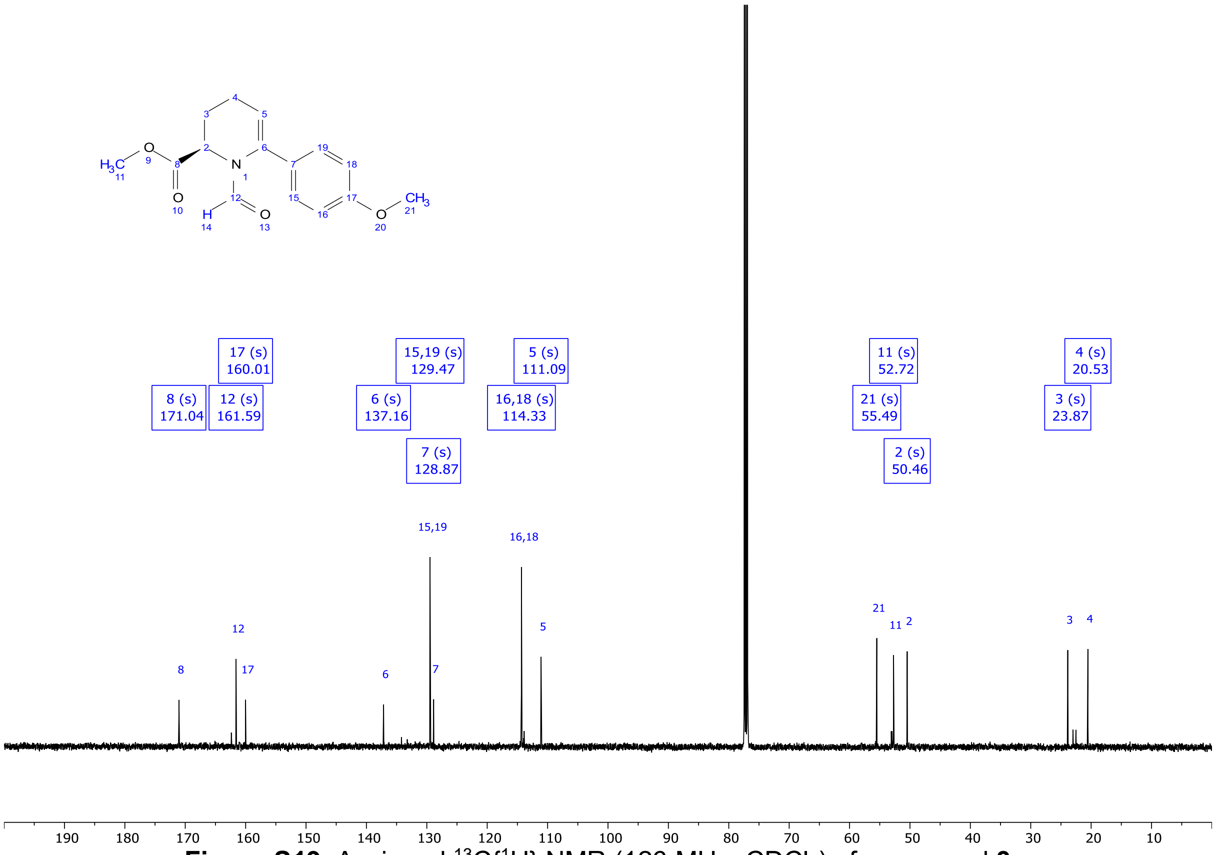

Figure S19: Assigned $^{13}C\{^{1}H\}$ NMR (126 MHz, $CDCl_3$) of compound **3e**.

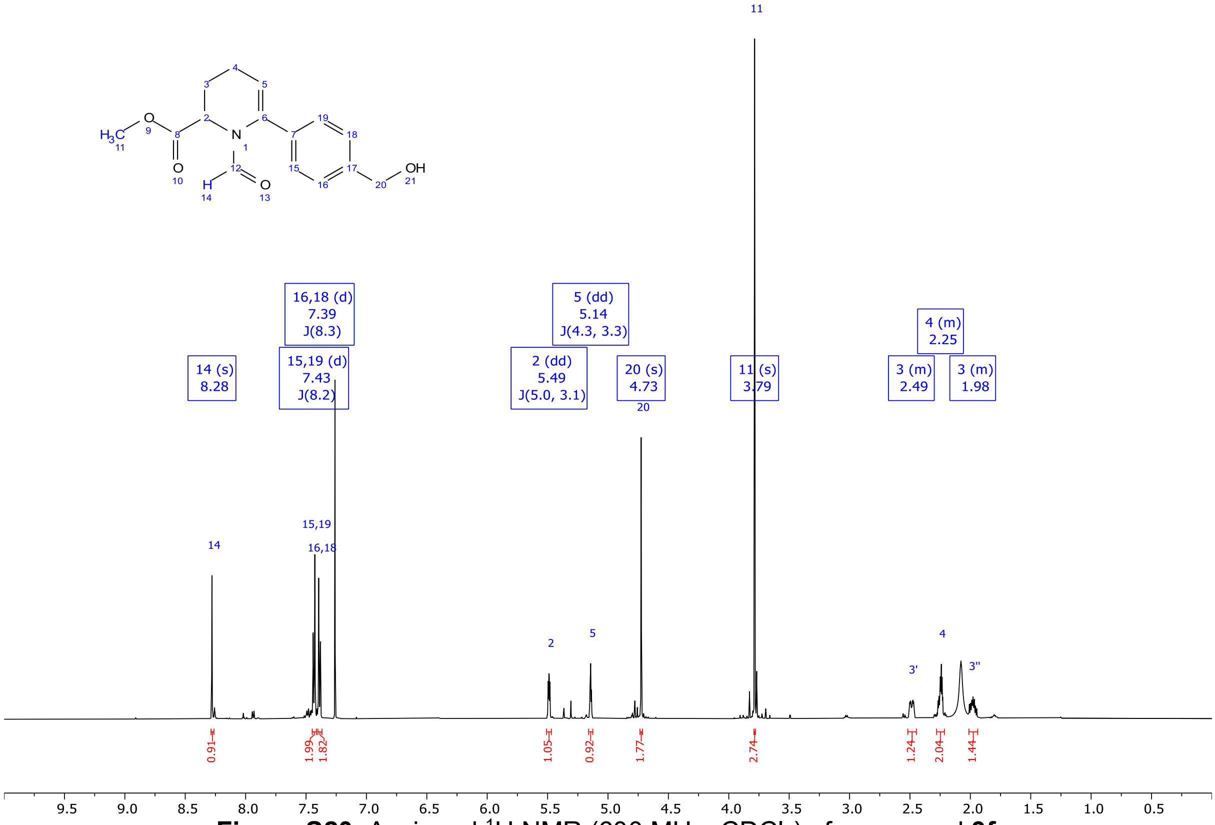

Figure S20: Assigned ^{1}H NMR (600 MHz, $CDCl_3$) of compound **3f**.

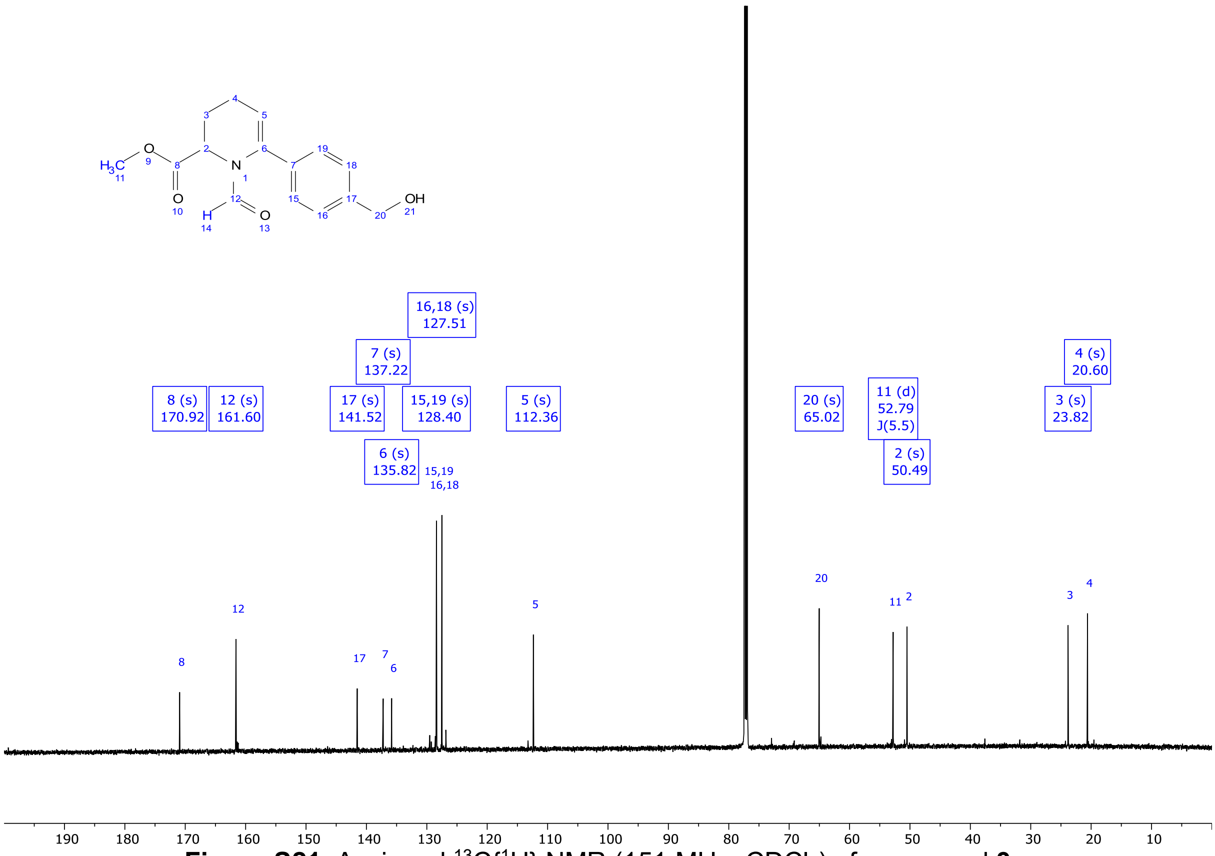

Figure S21: Assigned $^{13}C\{^{1}H\}$ NMR (151 MHz, $CDCl_3$) of compound **3e**.

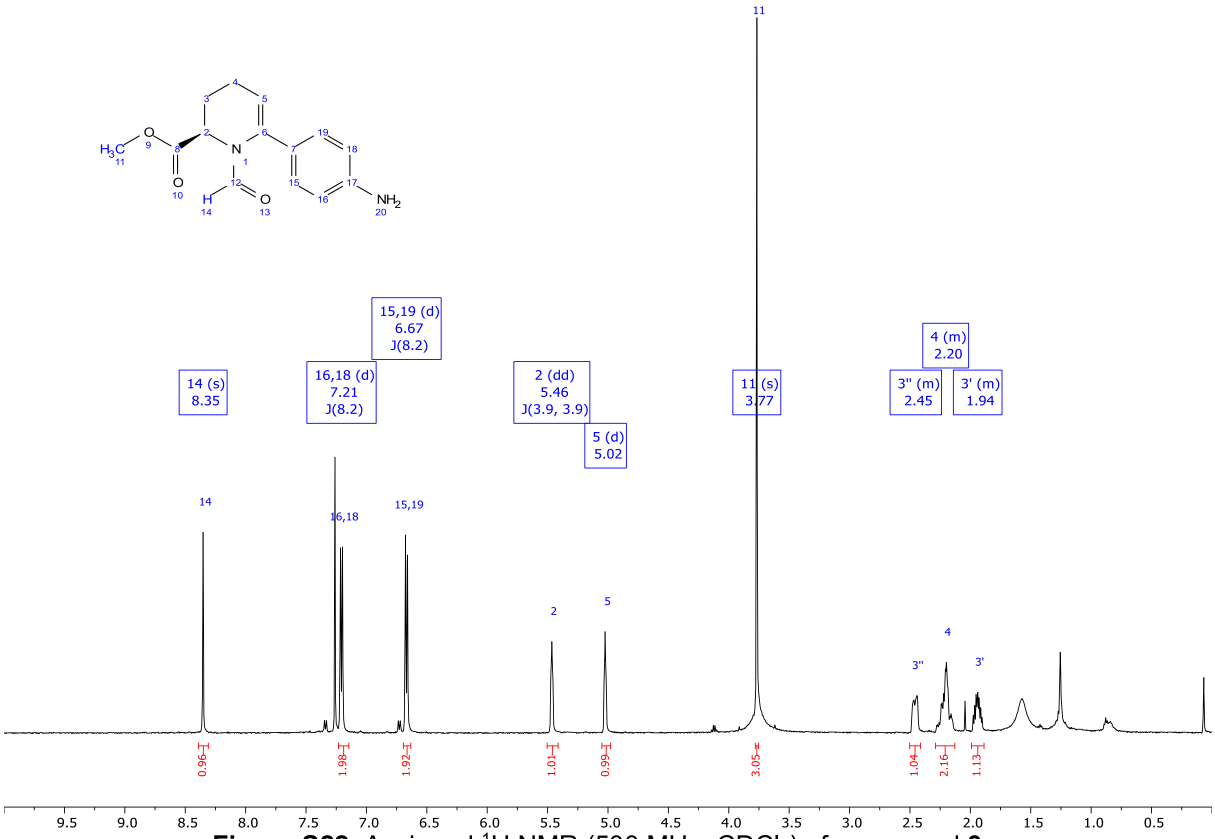

Figure S22: Assigned ^{1}H NMR (500 MHz, $CDCl_3$) of compound **3g**.

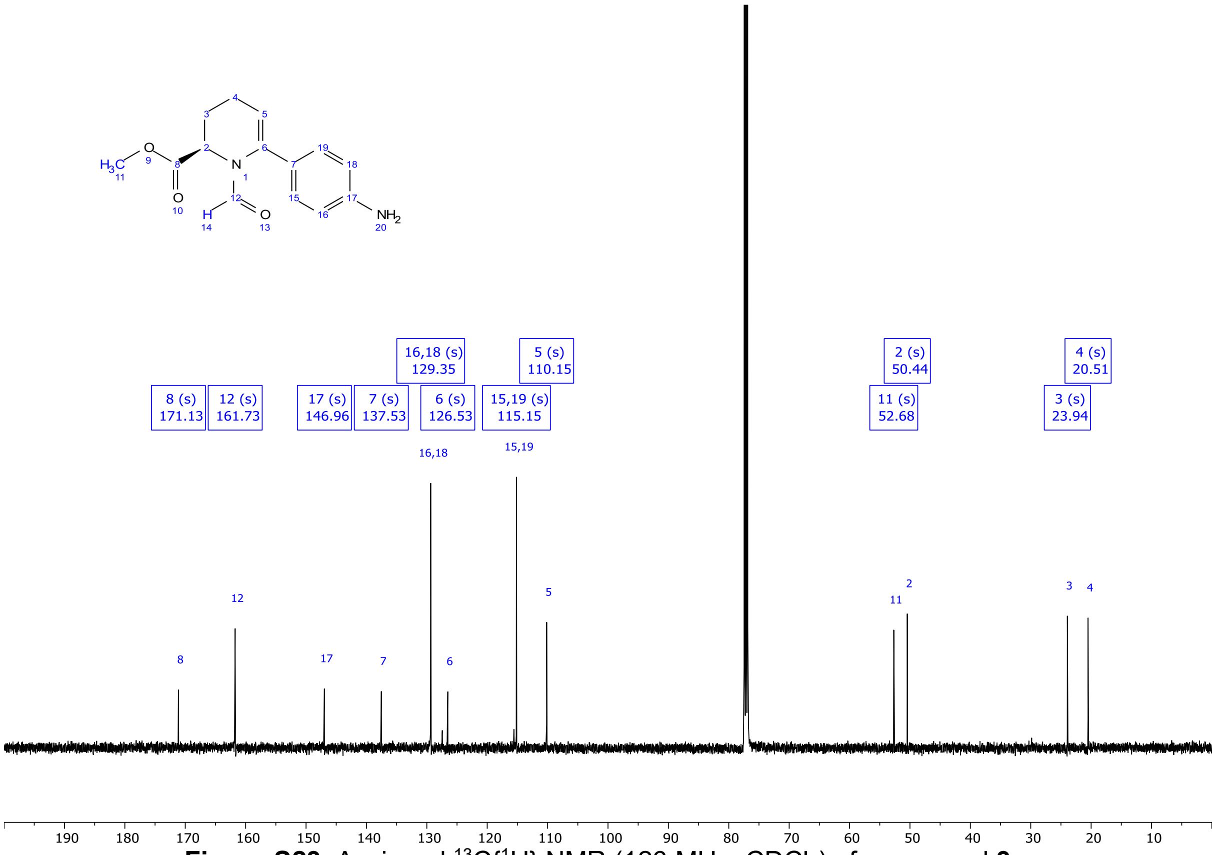

Figure S23: Assigned $^{13}C\{^{1}H\}$ NMR (126 MHz, $CDCl_3$) of compound **3g**.

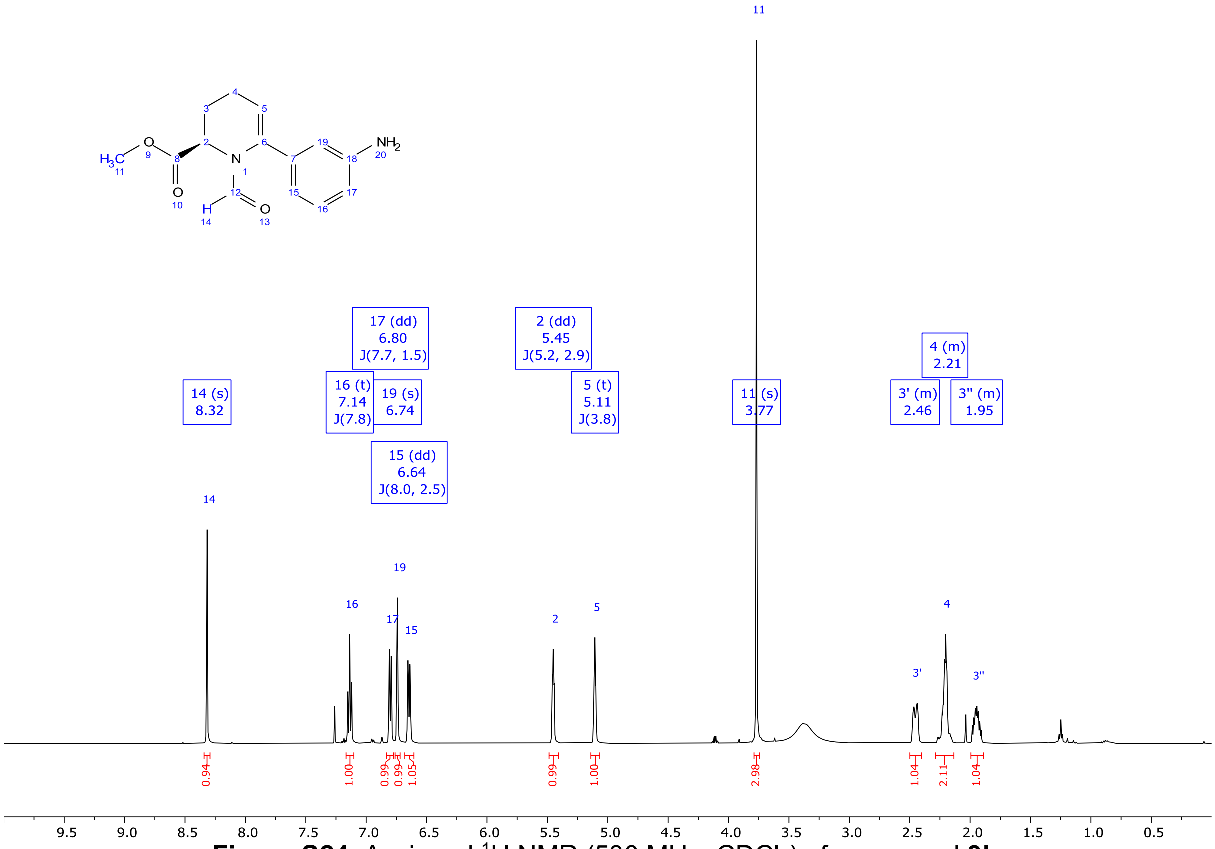

Figure S24: Assigned ^{1}H NMR (500 MHz, $CDCl_3$) of compound **3h**.

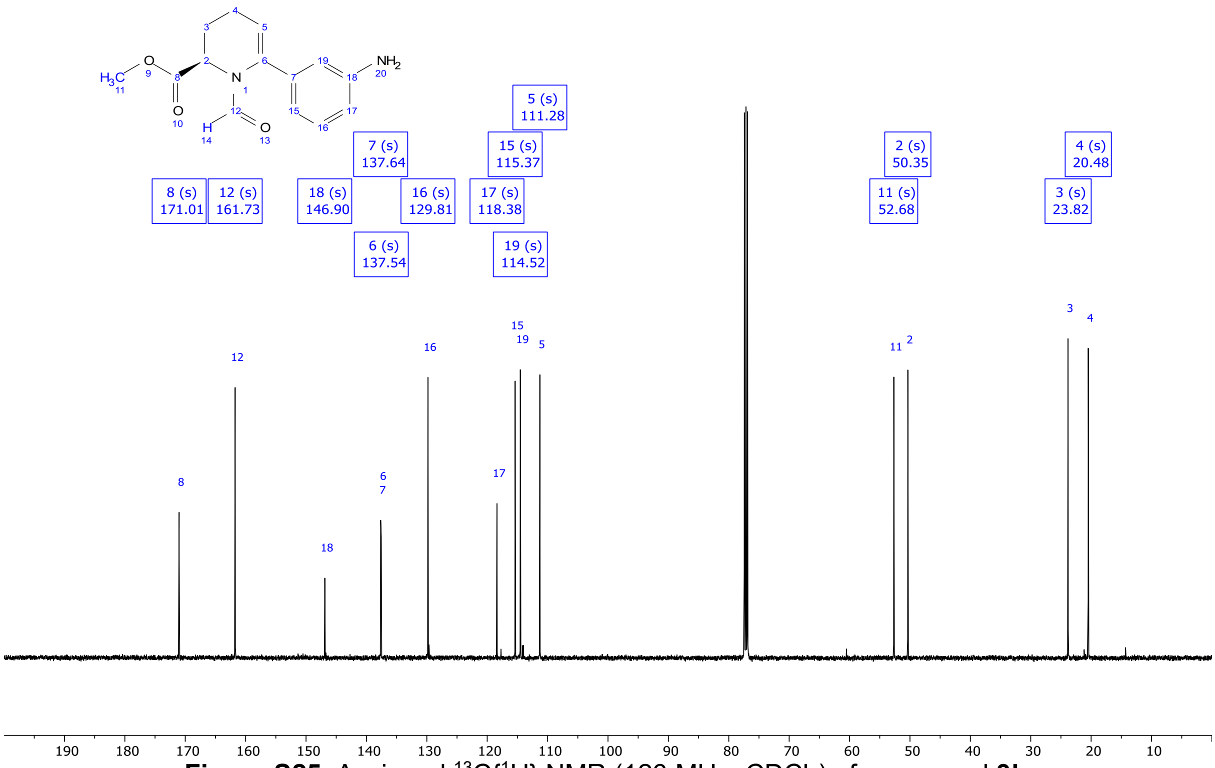

Figure S25: Assigned $^{13}C\{^{1}H\}$ NMR (126 MHz, $CDCl_3$) of compound **3h**.

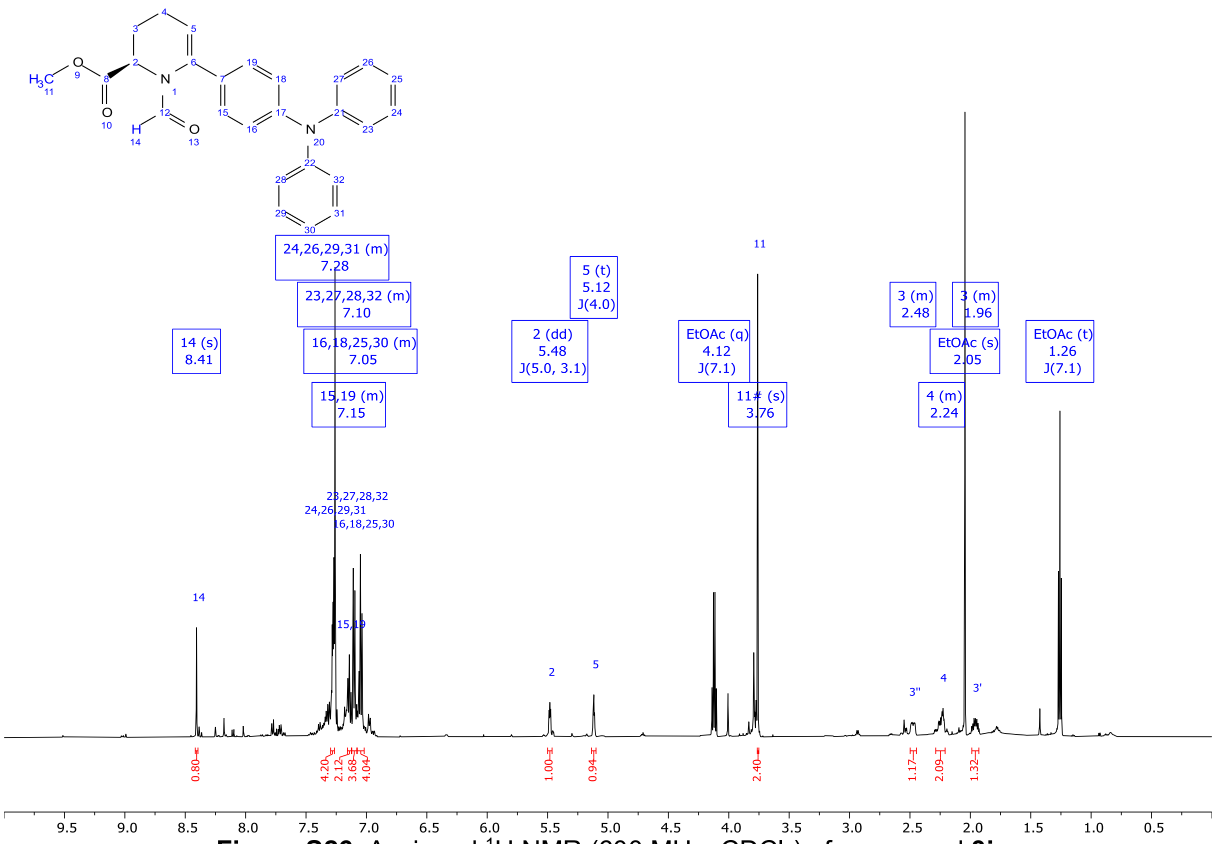

Figure S26: Assigned ^{1}H NMR (600 MHz, $CDCl_3$) of compound **3j**.

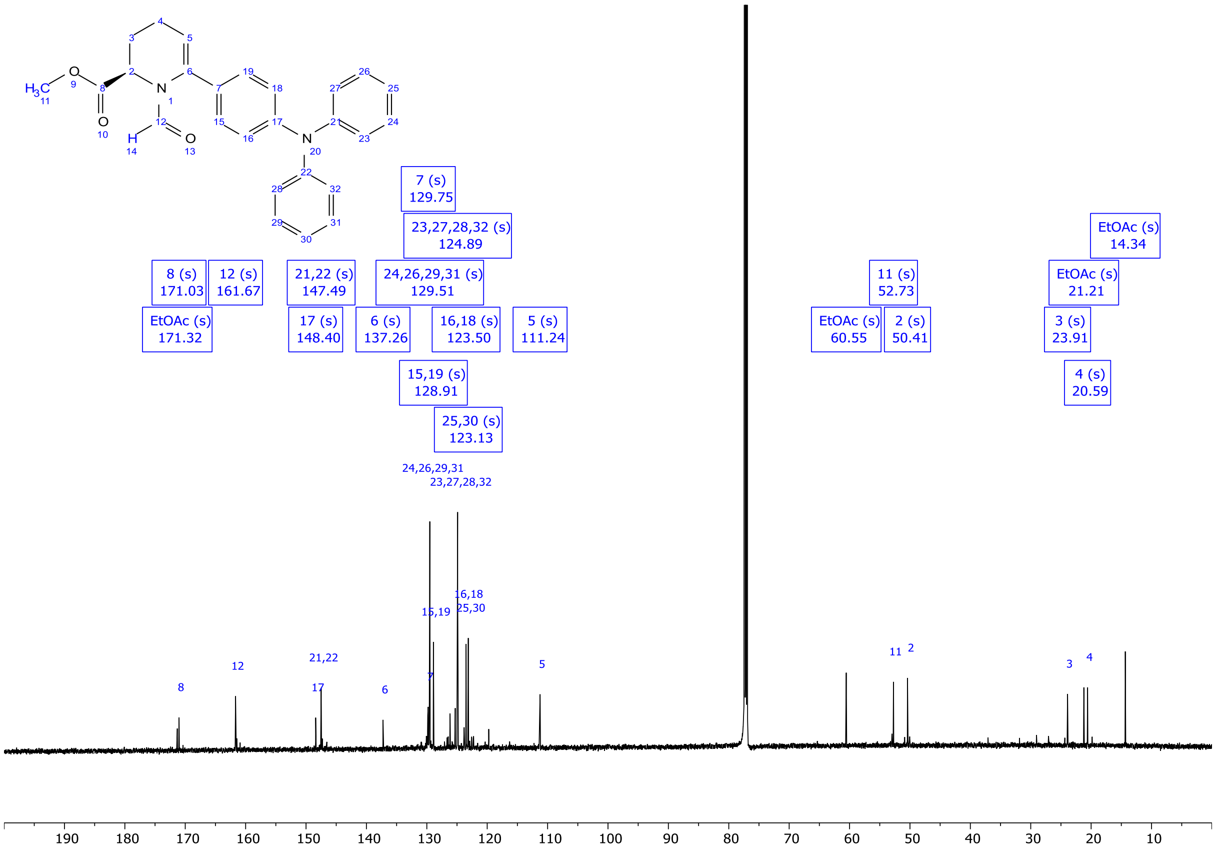

Figure S27: Assigned $^{13}C\{^{1}H\}$ NMR (151 MHz, $CDCl_3$) of compound **3j**.

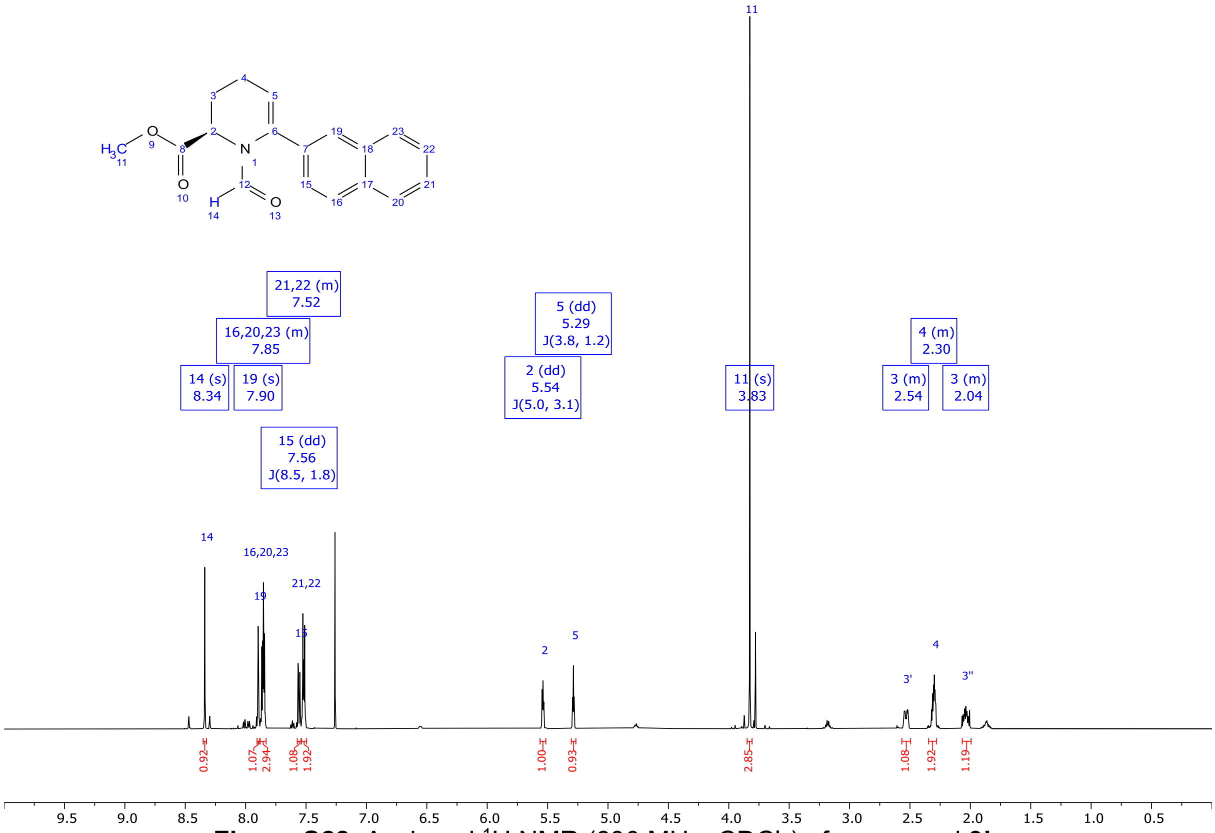

Figure S28: Assigned ^{1}H NMR (600 MHz, $CDCl_3$) of compound **3k**.

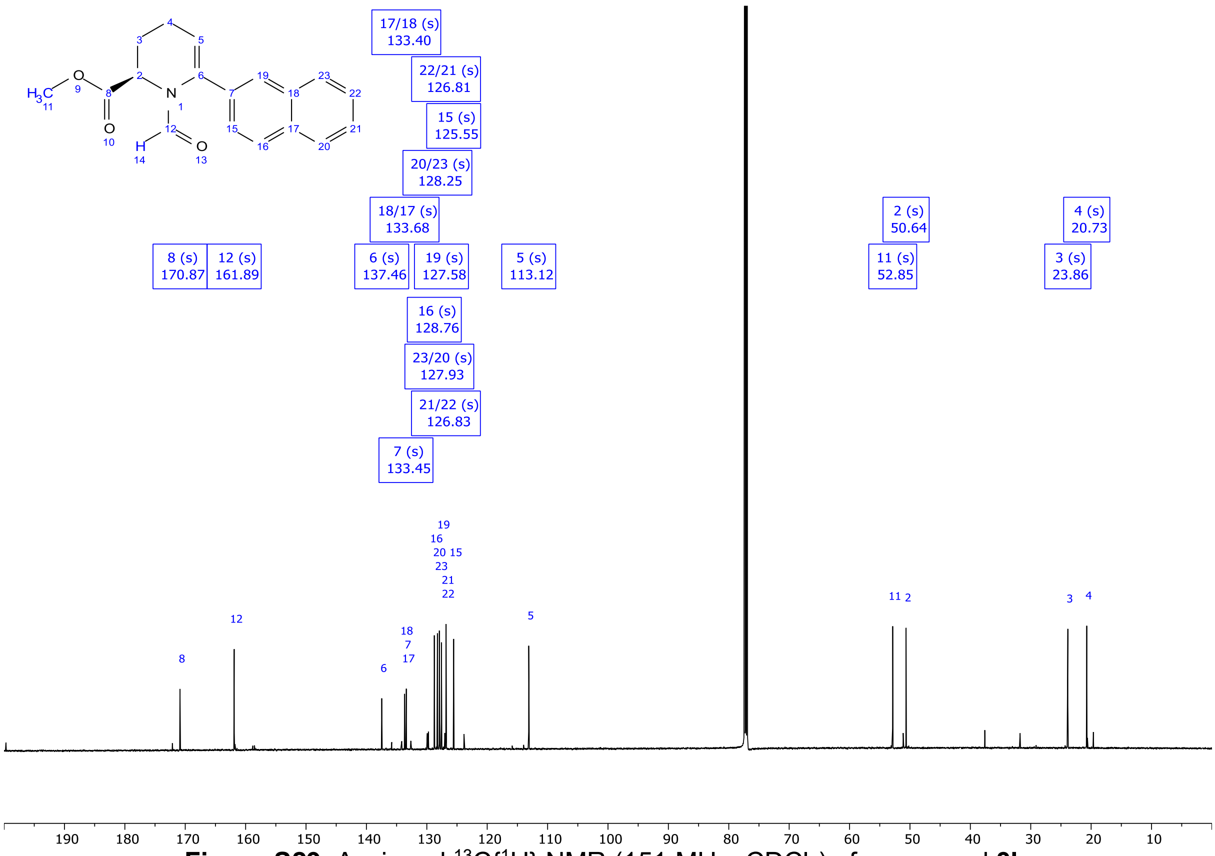

Figure S29: Assigned $^{13}C\{^{1}H\}$ NMR (151 MHz, $CDCl_3$) of compound **3k**.

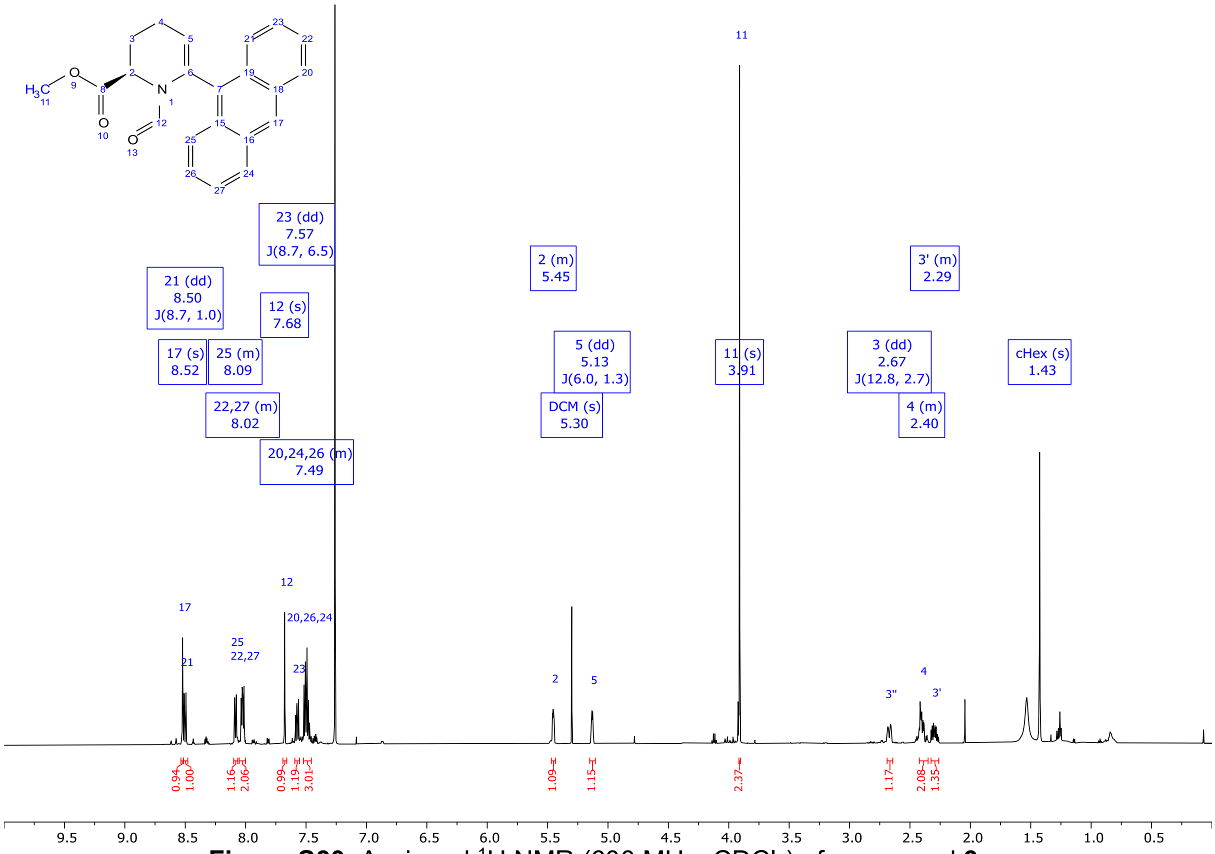

Figure S30: Assigned ^{1}H NMR (600 MHz, $CDCl_3$) of compound **3m**.

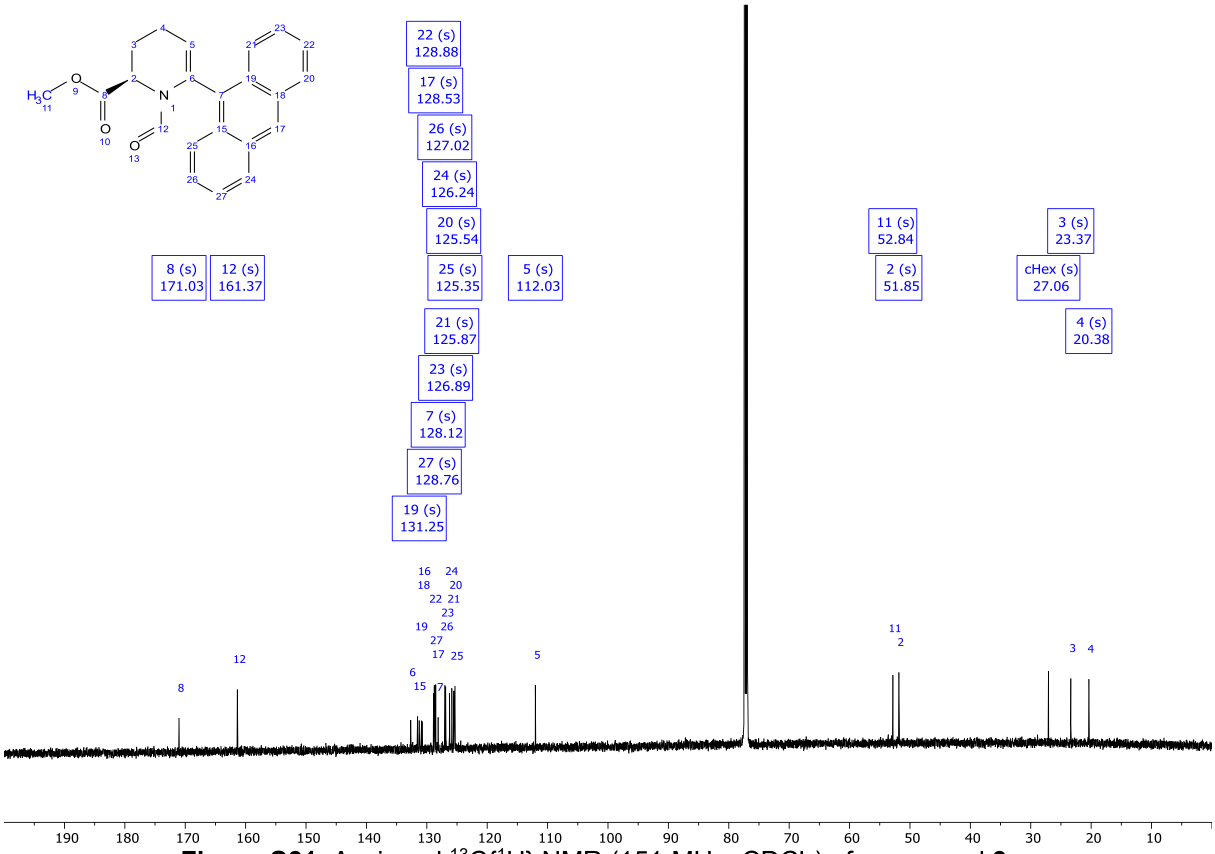

Figure S31: Assigned $^{13}C\{^{1}H\}$ NMR (151 MHz, $CDCl_3$) of compound **3m**.

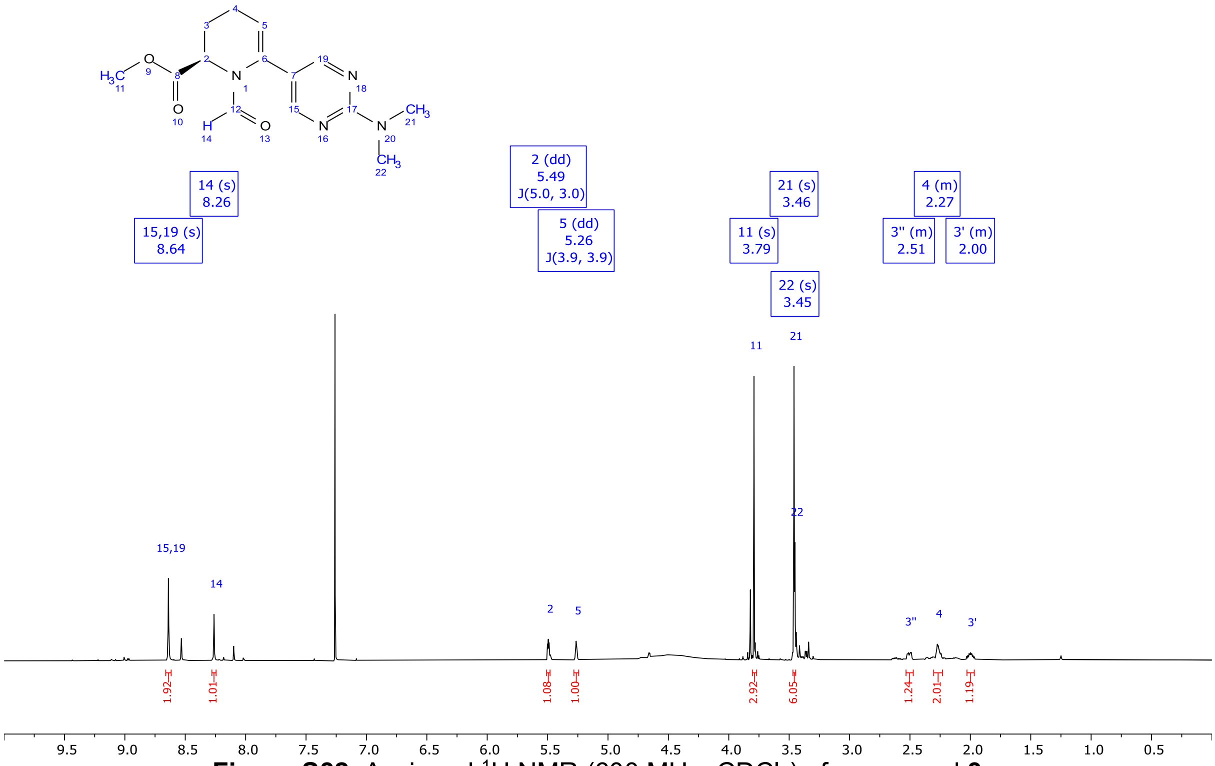

Figure S32: Assigned ^{1}H NMR (600 MHz, $CDCl_3$) of compound **3p**.

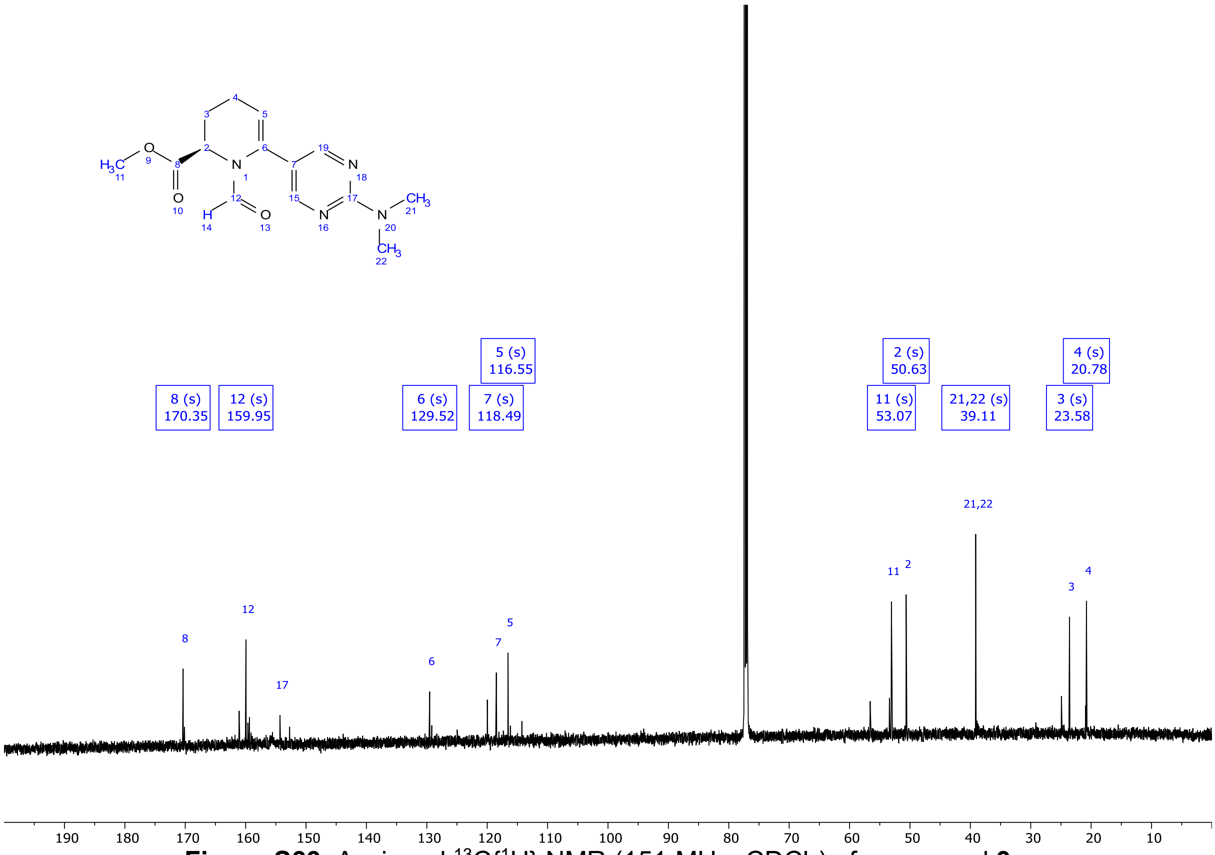

Figure S33: Assigned $^{13}C\{^{1}H\}$ NMR (151 MHz, $CDCl_3$) of compound **3p**.

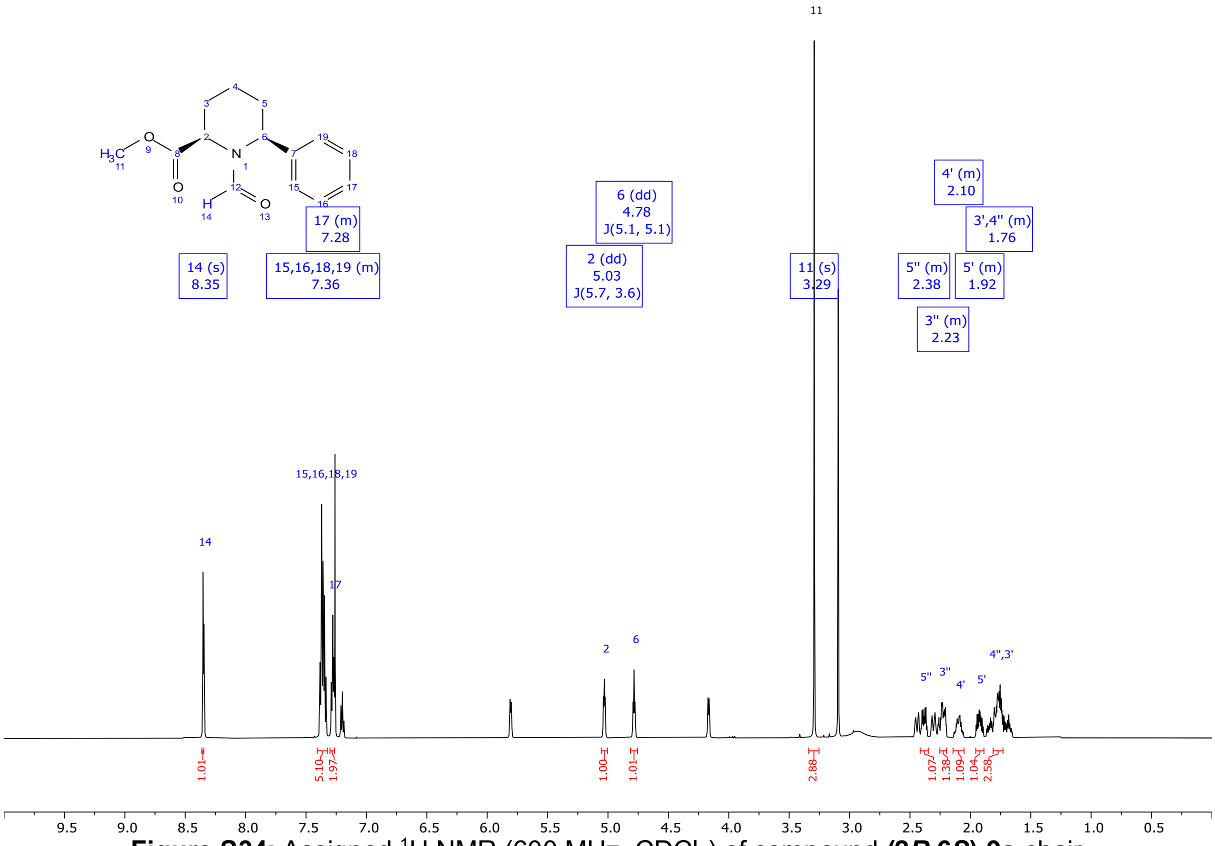

Figure S34: Assigned ^{1}H NMR (600 MHz, $CDCl_3$) of compound **(2*R*,6*S*)-9a** chair.

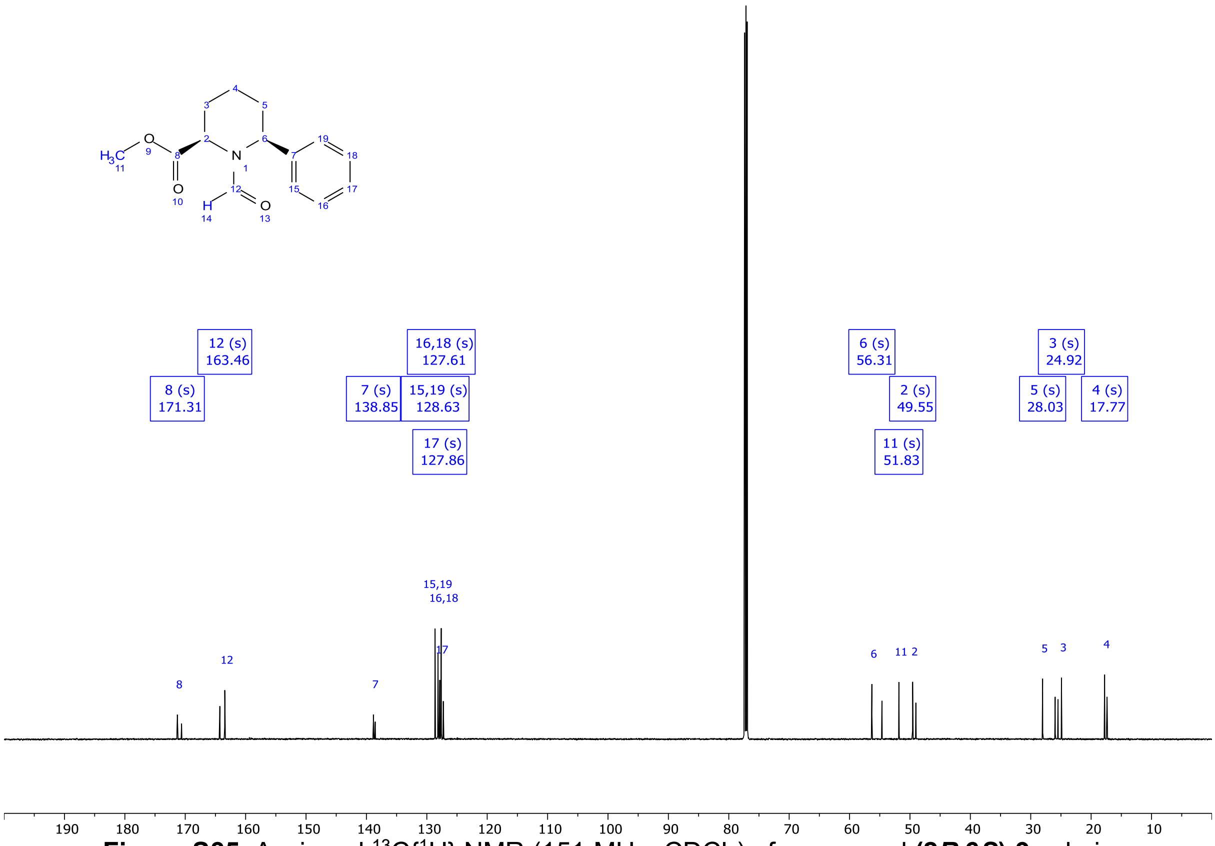

Figure S35: Assigned $^{13}C\{^{1}H\}$ NMR (151 MHz, $CDCl_3$) of compound **(2*R*,6*S*)-9a** chair.

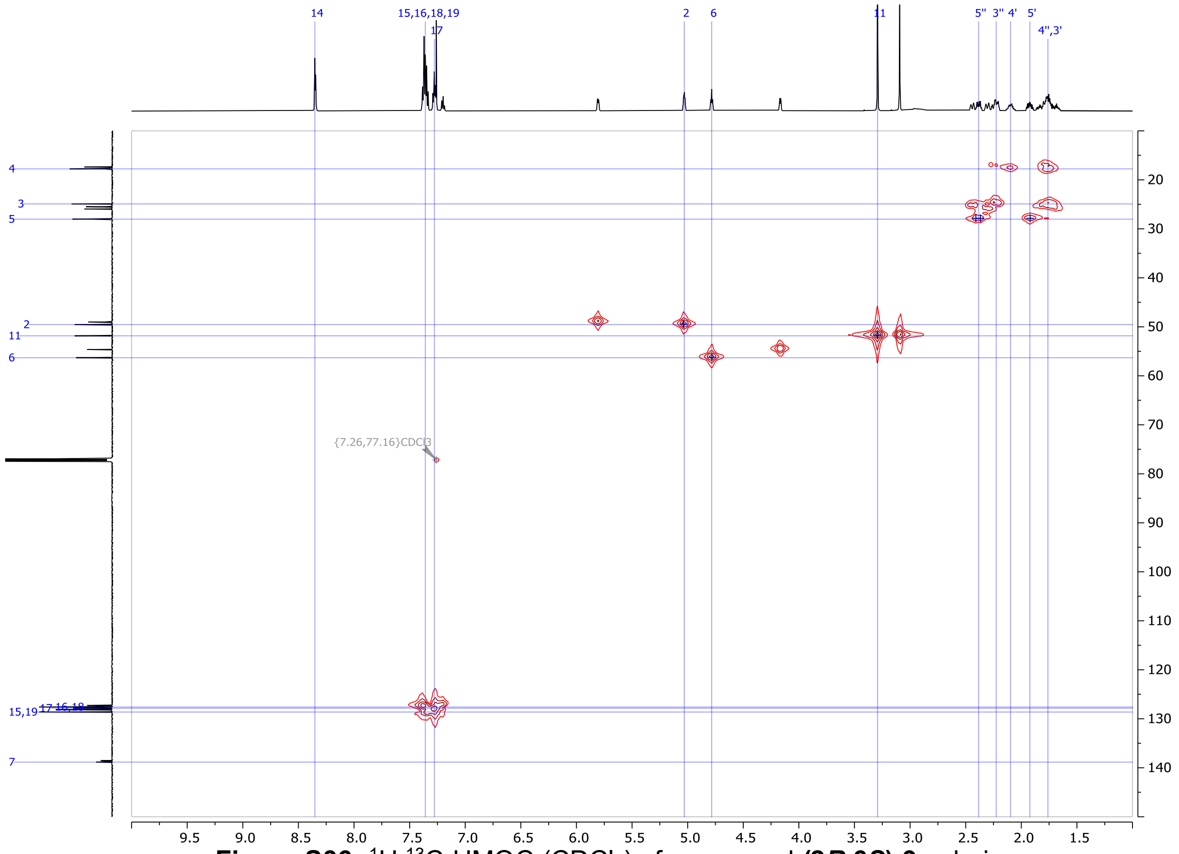

Figure S36: [1]H,[13]C-HMQC ($CDCl_3$) of compound **(2*R*,6*S*)-9a** chair.

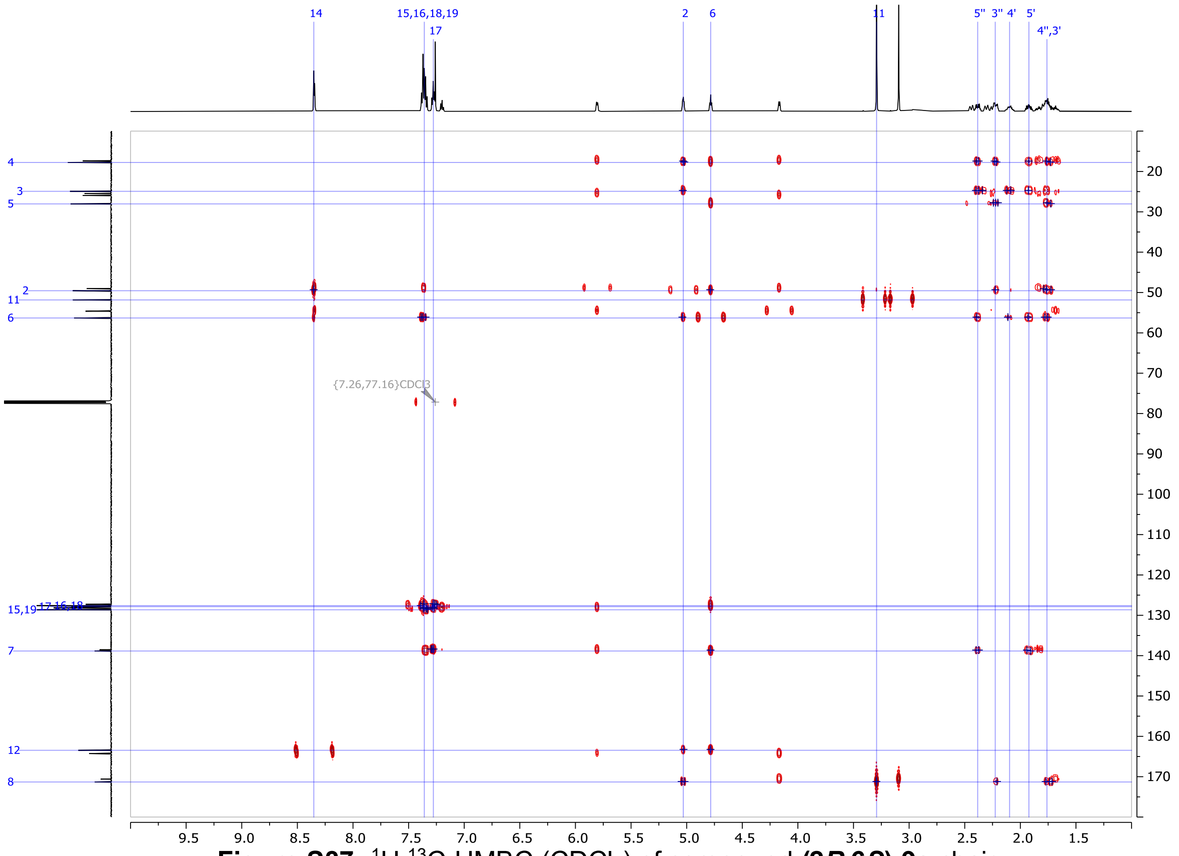

Figure S37: ^{1}H,^{13}C-HMBC ($CDCl_3$) of compound **(2*R*,6*S*)-9a** chair.

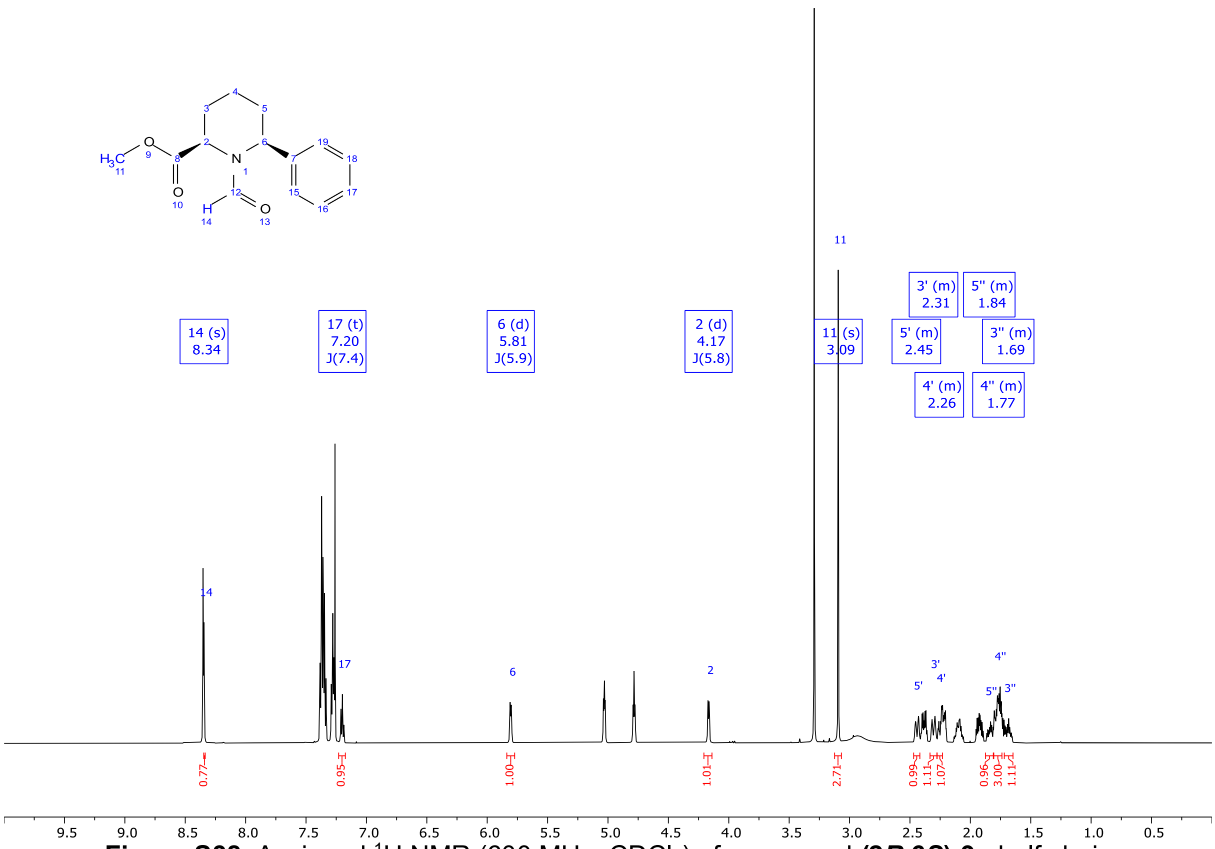

Figure S38: Assigned ^{1}H NMR (600 MHz, $CDCl_3$) of compound **(2*R*,6*S*)-9a** half-chair.

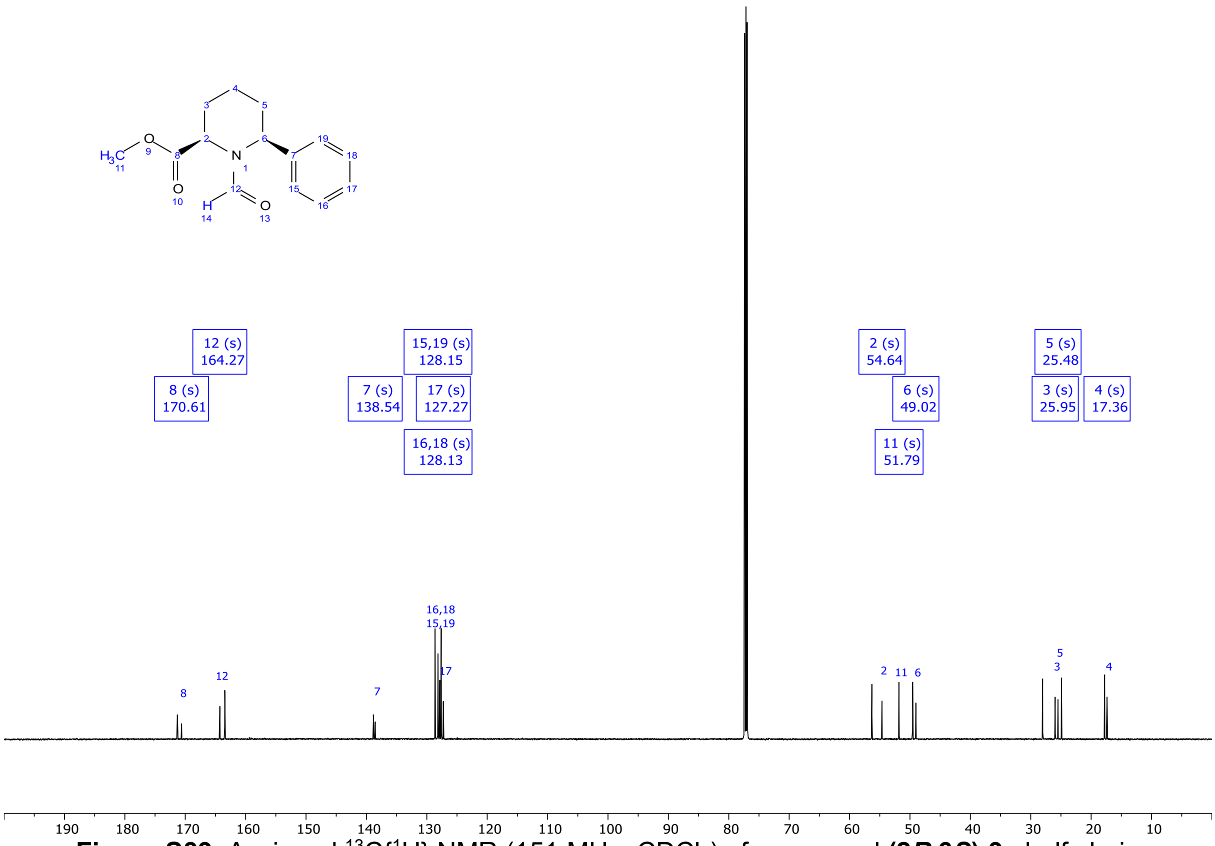

Figure S39: Assigned $^{13}C\{^{1}H\}$ NMR (151 MHz, $CDCl_3$) of compound **(2*R*,6*S*)-9a** half-chair.

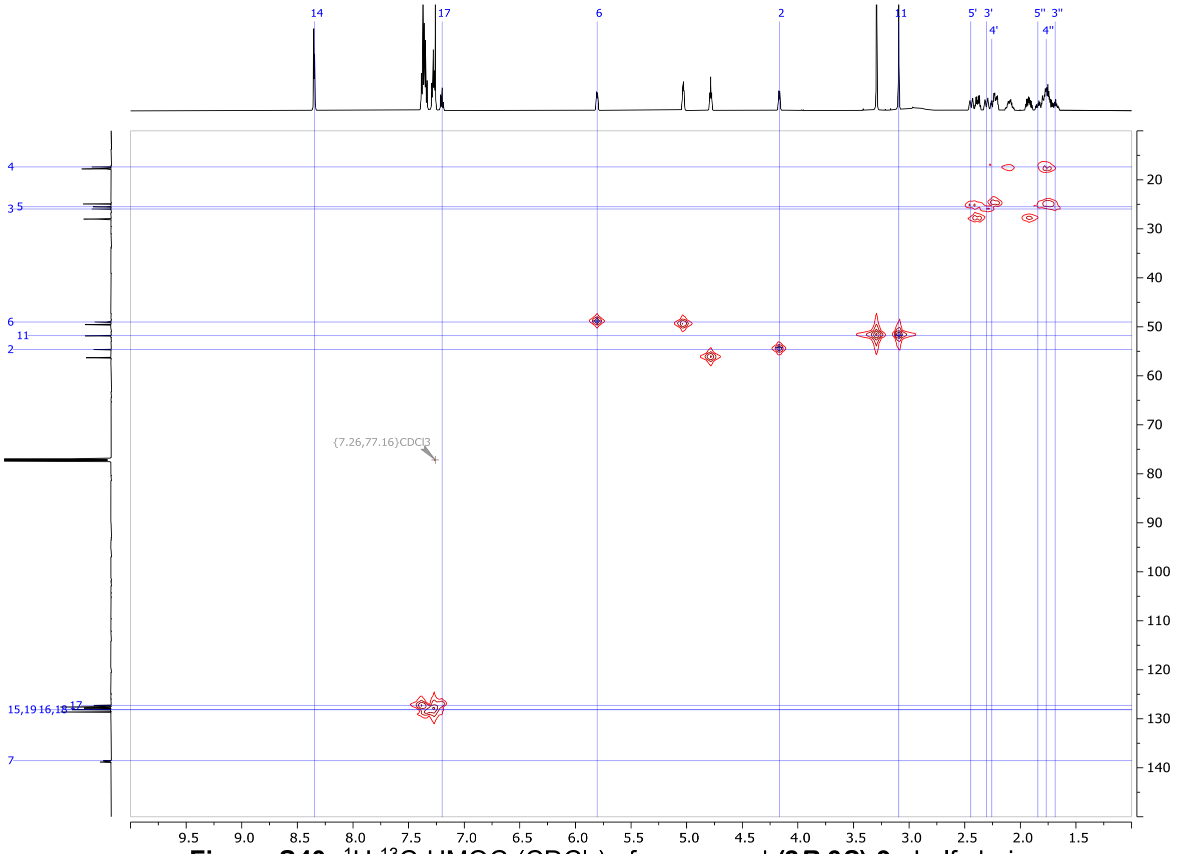

Figure S40: $^{1}H,^{13}C$-HMQC ($CDCl_3$) of compound **(2*R*,6*S*)-9a** half-chair.

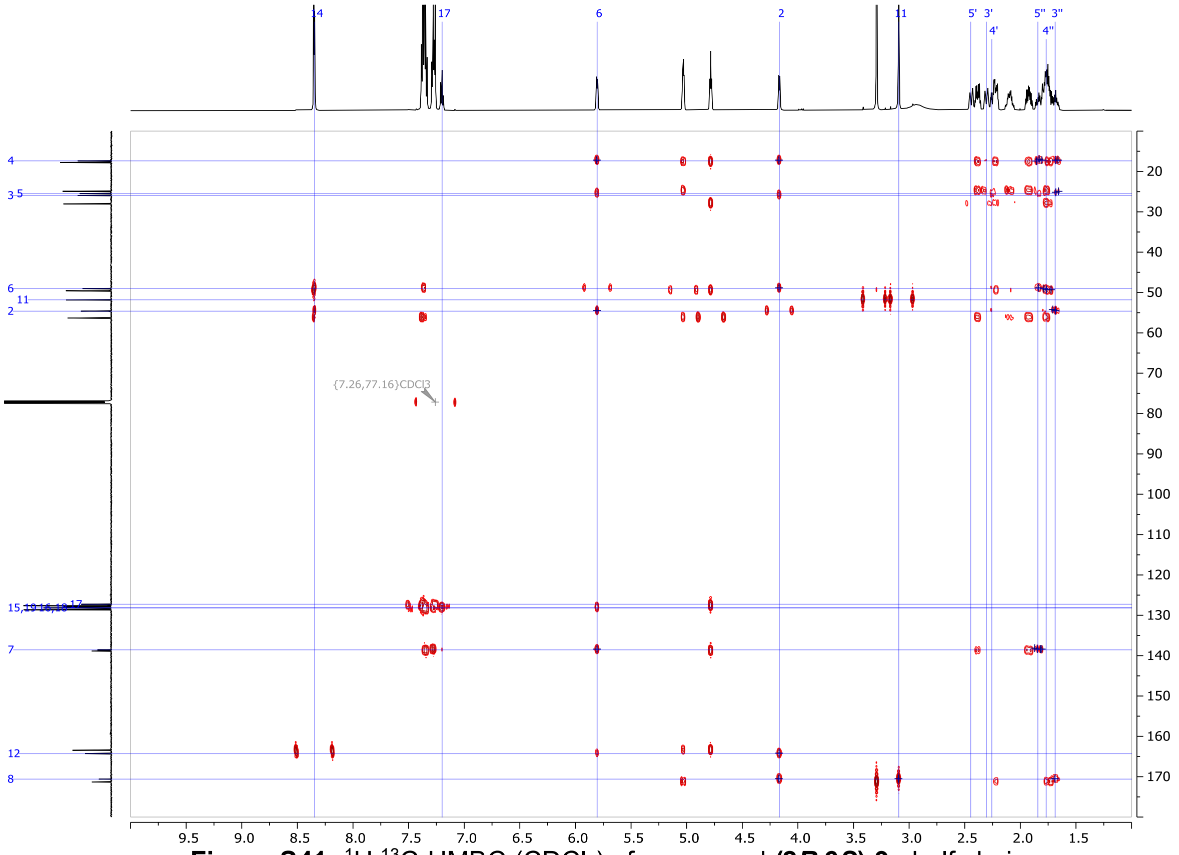

Figure S41: $^{1}H,^{13}C$-HMBC ($CDCl_3$) of compound **(2*R*,6*S*)-9a** half-chair.

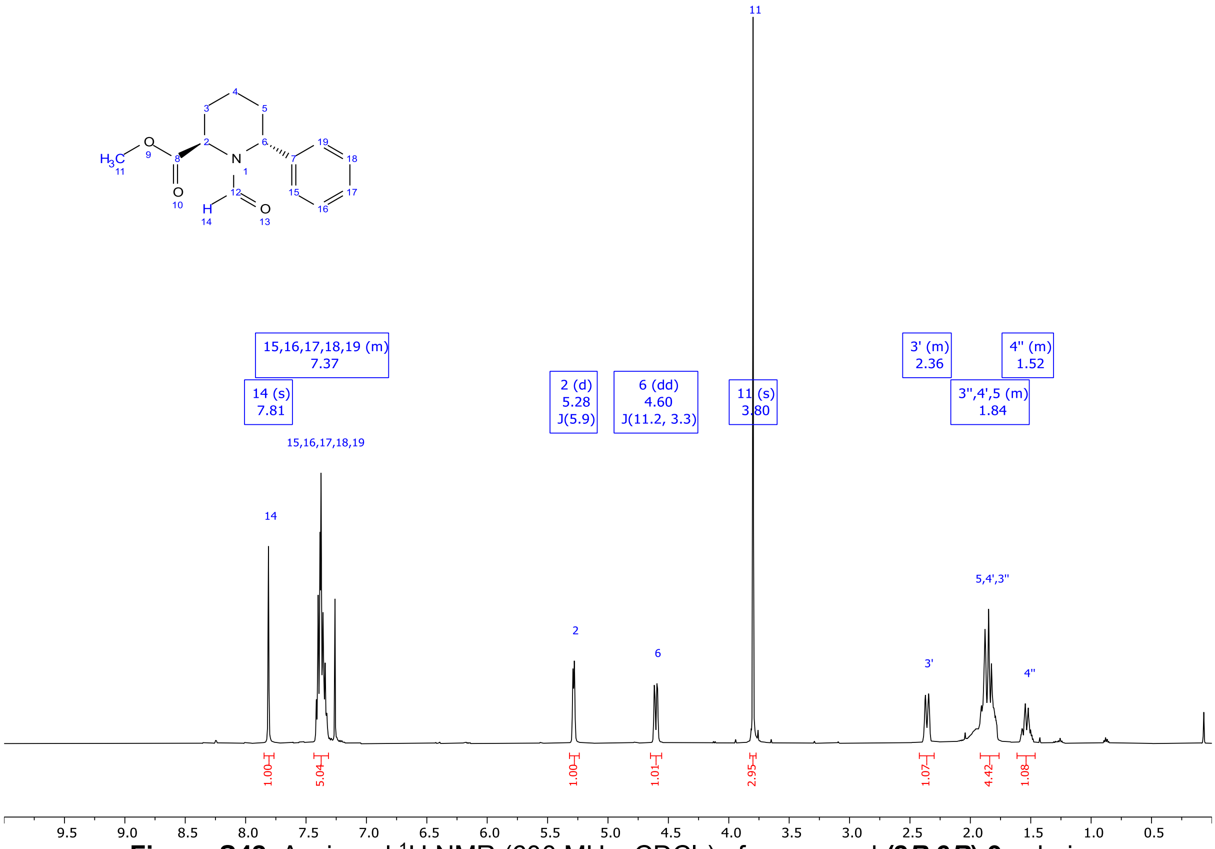

Figure S42: Assigned ^{1}H NMR (600 MHz, $CDCl_3$) of compound **(2*R*,6*R*)-9a** chair.

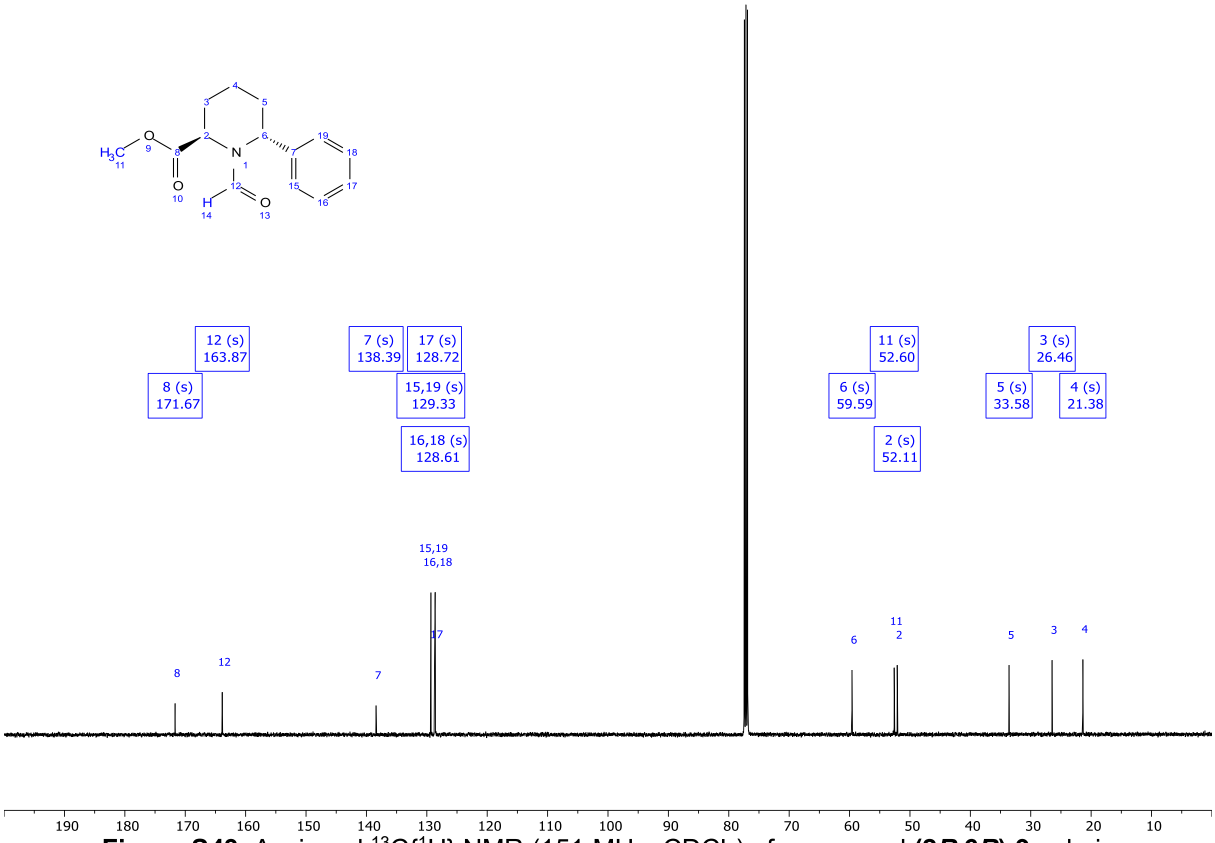

Figure S43: Assigned $^{13}C\{^{1}H\}$ NMR (151 MHz, $CDCl_3$) of compound **(2*R*,6*R*)-9a** chair.

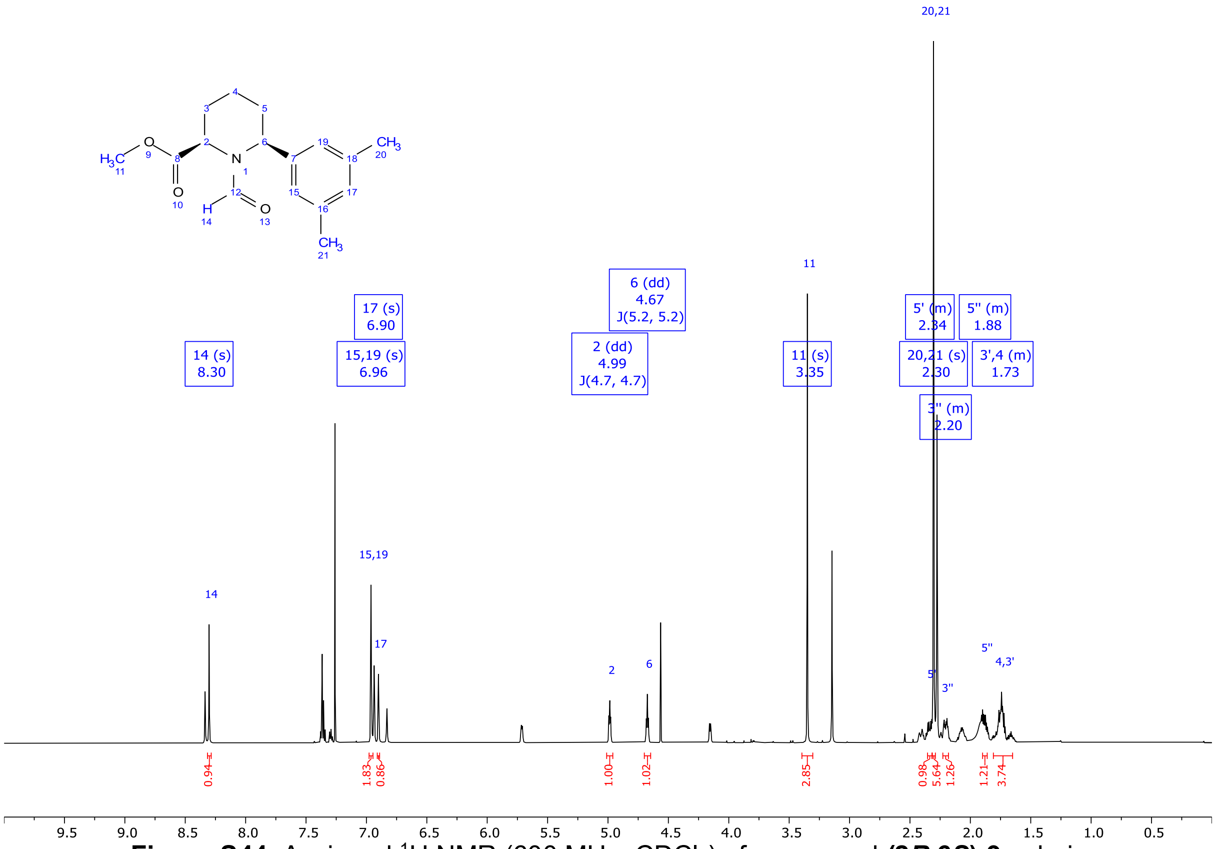

Figure S44: Assigned ^{1}H NMR (600 MHz, $CDCl_3$) of compound **(2*R*,6*S*)-9c** chair.

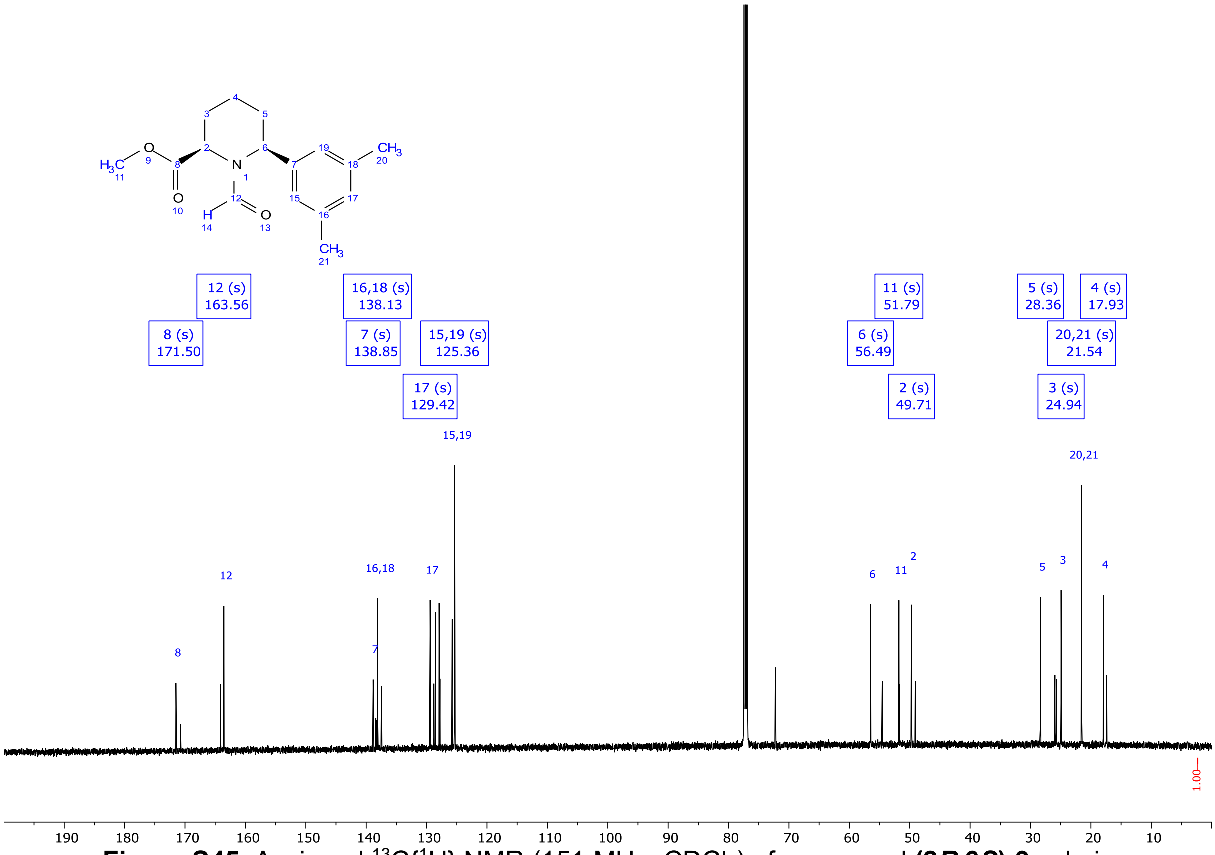

Figure S45: Assigned $^{13}C\{^{1}H\}$ NMR (151 MHz, $CDCl_3$) of compound **(2*R*,6*S*)-9c** chair.

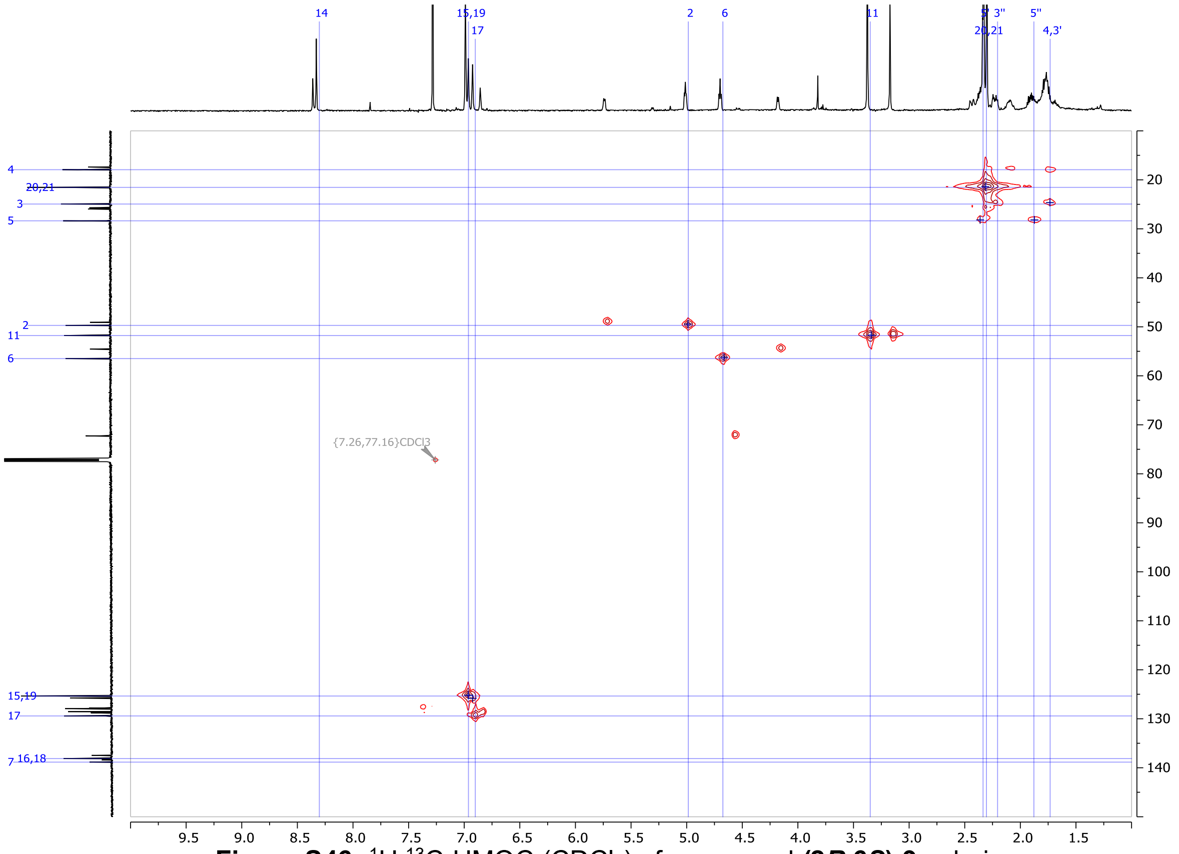

Figure S46: ^{1}H,^{13}C-HMQC (CDCl₃) of compound **(2*R*,6*S*)-9c** chair.

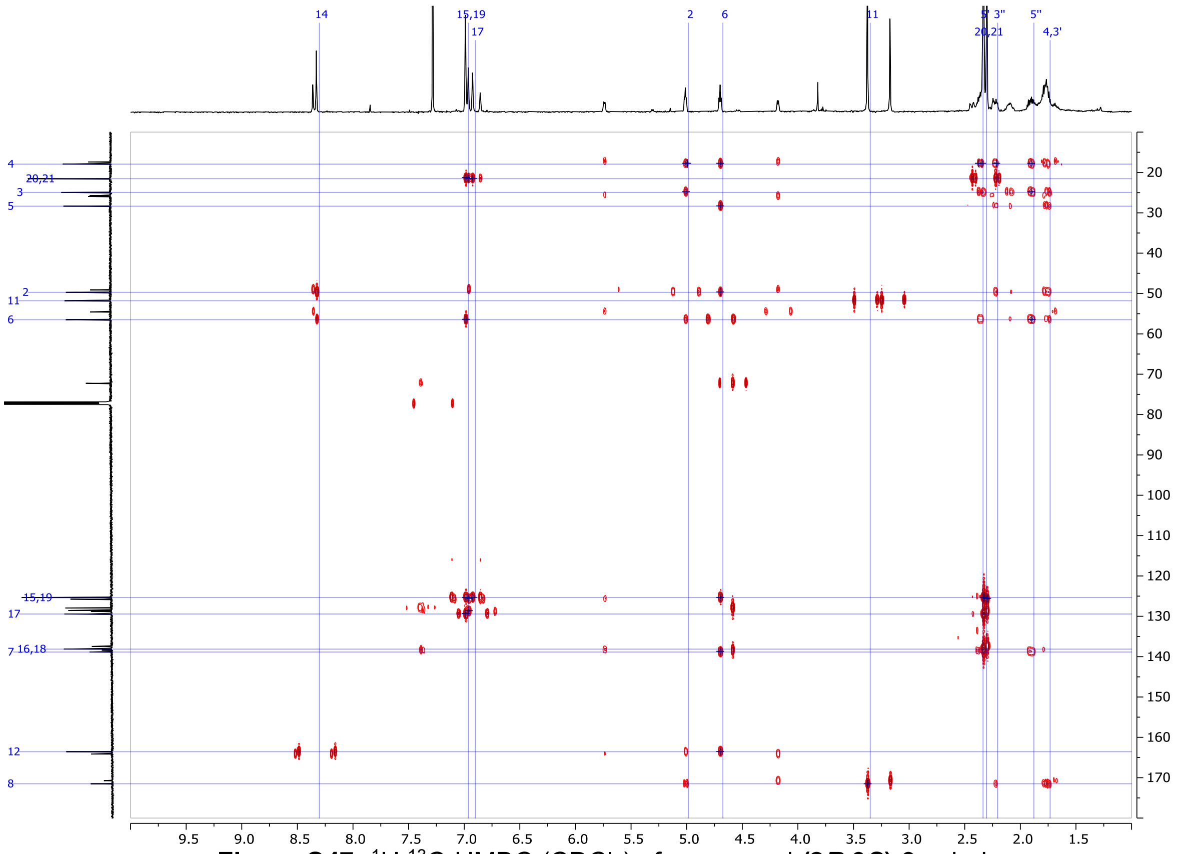

Figure S47: ^{1}H,^{13}C-HMBC (CDCl₃) of compound **(2*R*,6*S*)-9c** chair.

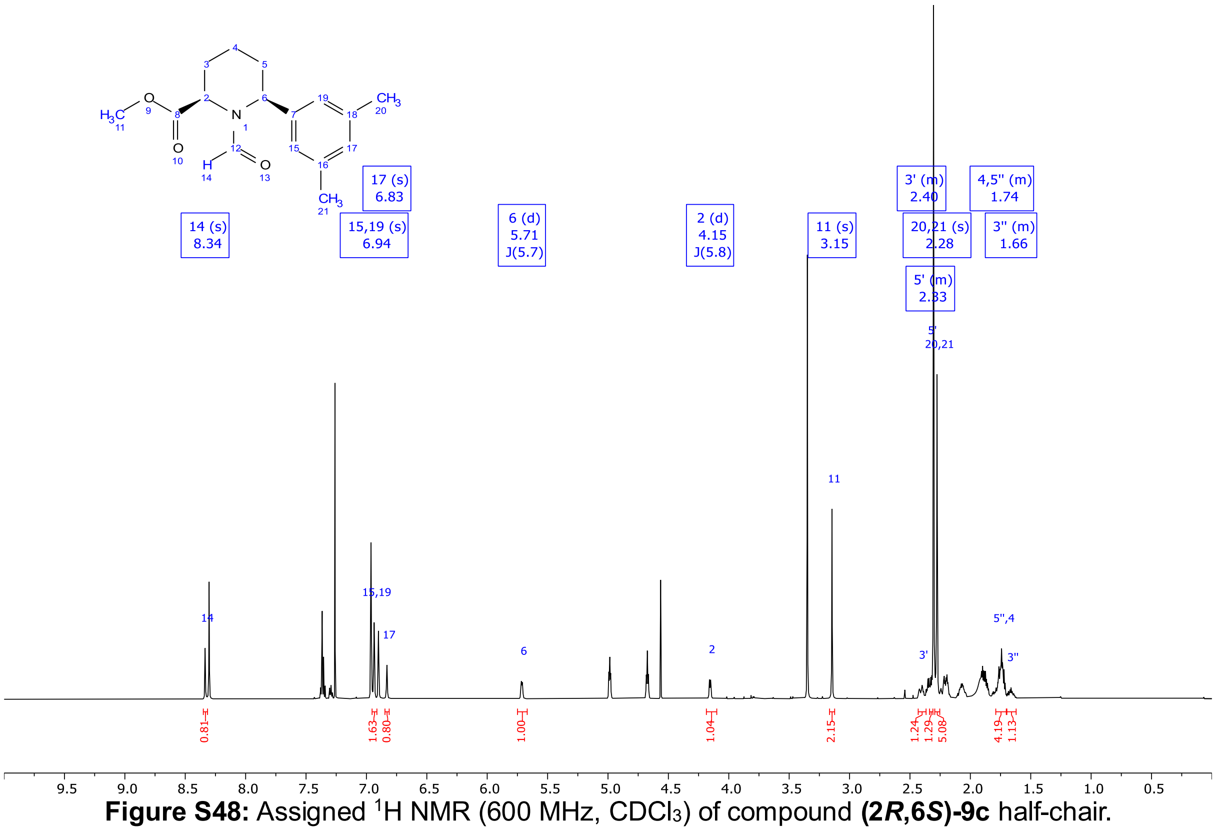

Figure S48: Assigned ^{1}H NMR (600 MHz, $CDCl_3$) of compound **(2*R*,6*S*)-9c** half-chair.

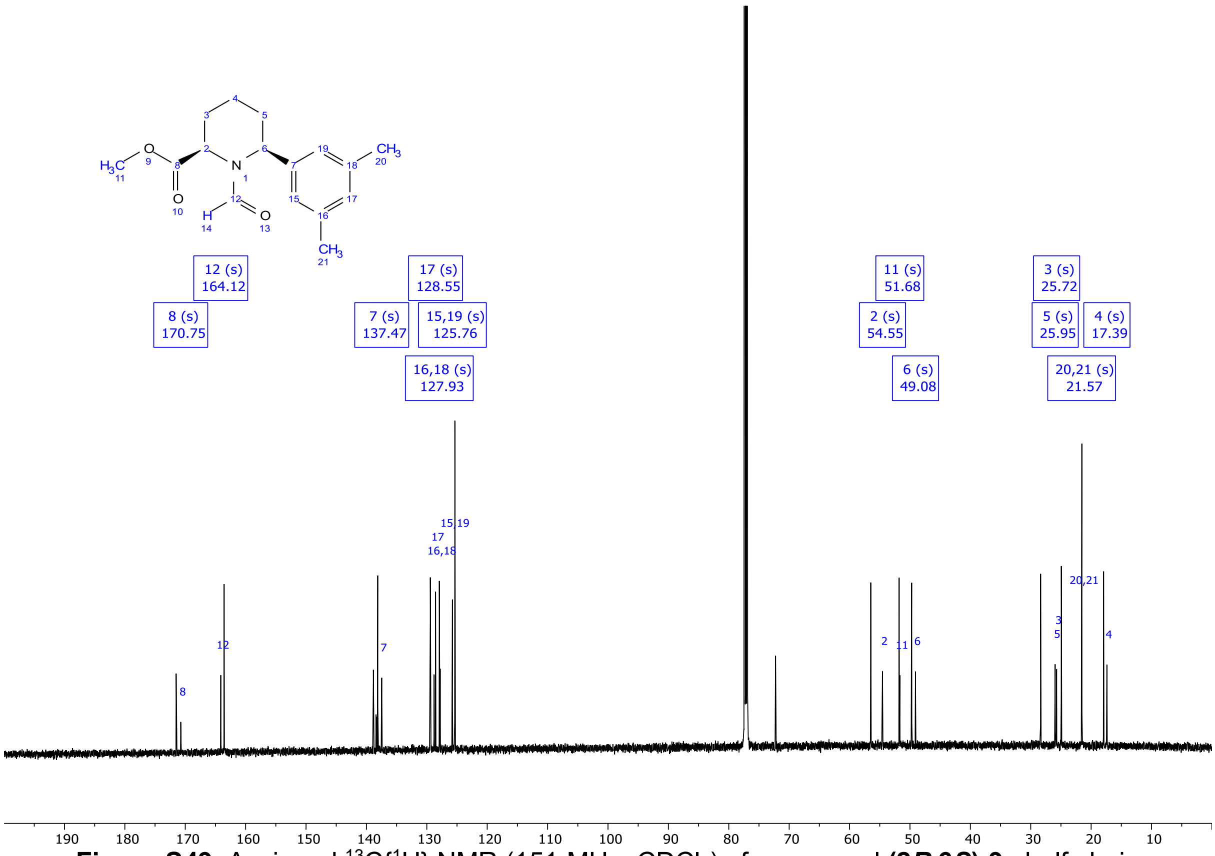

Figure S49: Assigned ^{13}C{^{1}H} NMR (151 MHz, $CDCl_3$) of compound **(2*R*,6*S*)-9c** half-chair.

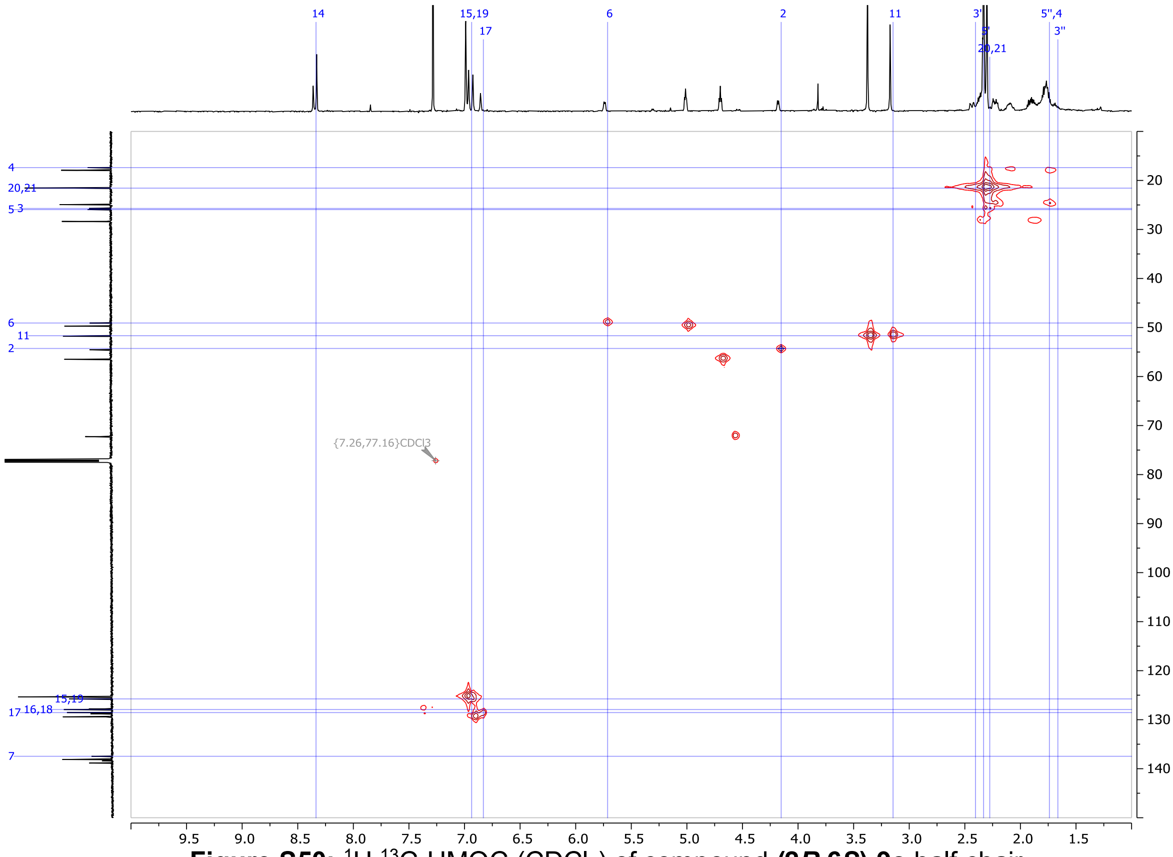

Figure S50: $^{1}H,^{13}C$-HMQC ($CDCl_3$) of compound **(2*R*,6*S*)-9c** half-chair.

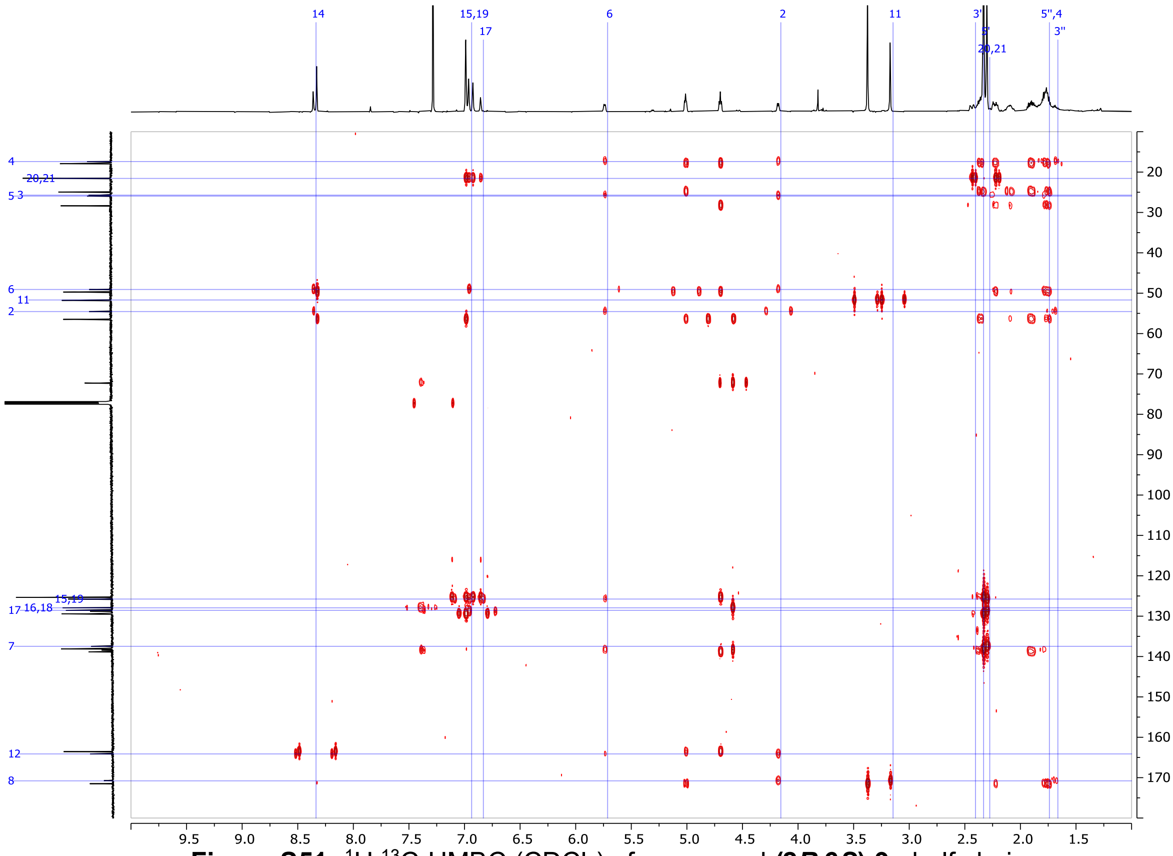

Figure S51: $^{1}H,^{13}C$-HMBC ($CDCl_3$) of compound **(2*R*,6*S*)-9c** half-chair.

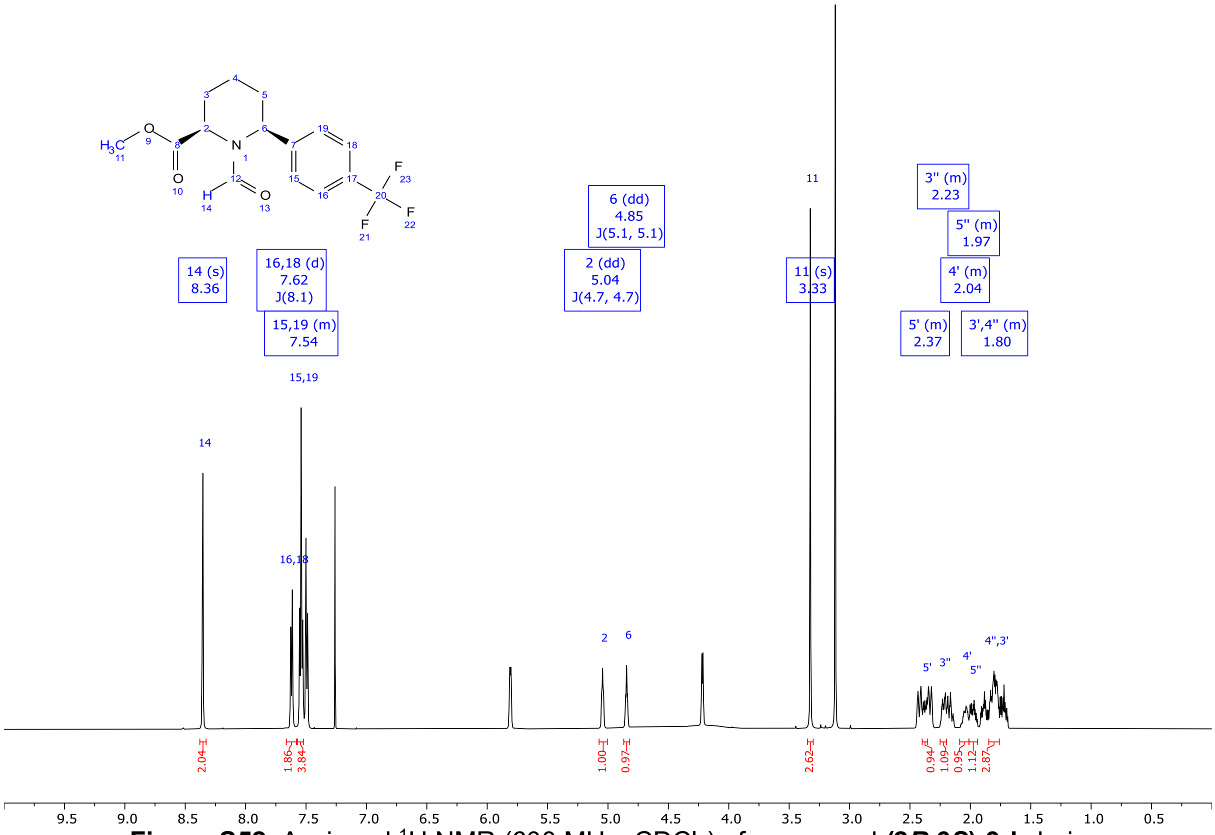

Figure S52: Assigned ^{1}H NMR (600 MHz, $CDCl_3$) of compound **(2*R*,6*S*)-9d** chair.

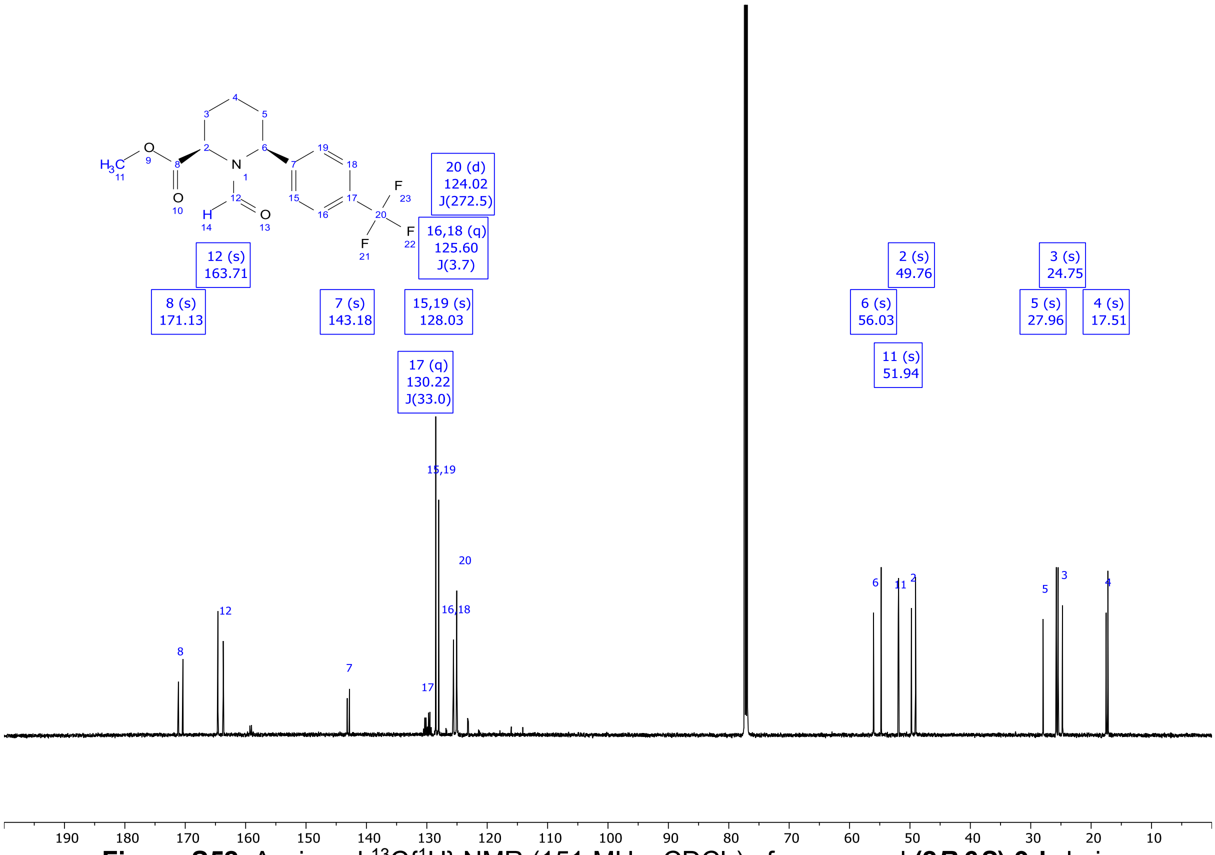

Figure S52: Assigned $^{13}C\{^{1}H\}$ NMR (151 MHz, $CDCl_3$) of compound **(2*R*,6*S*)-9d** chair.

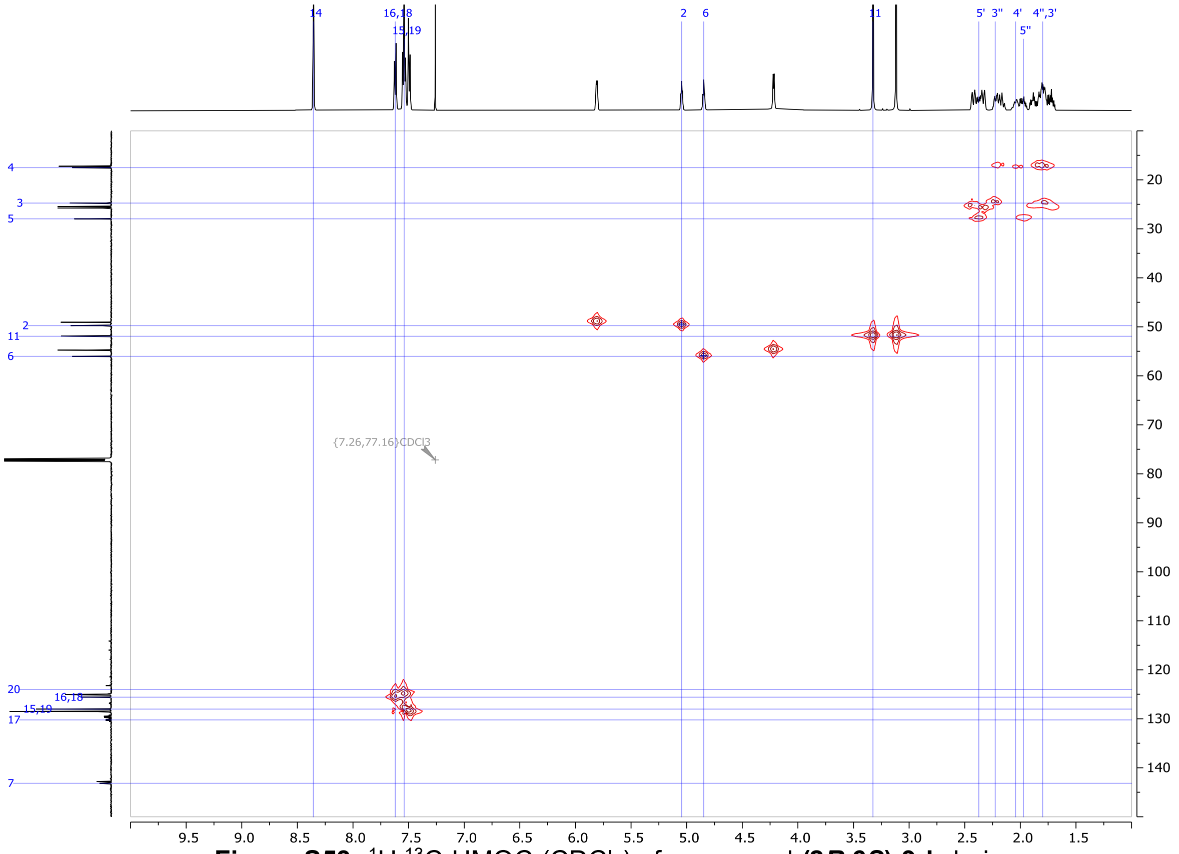

Figure S53: ^{1}H,^{13}C-HMQC ($CDCl_3$) of compound **(2*R*,6*S*)-9d** chair.

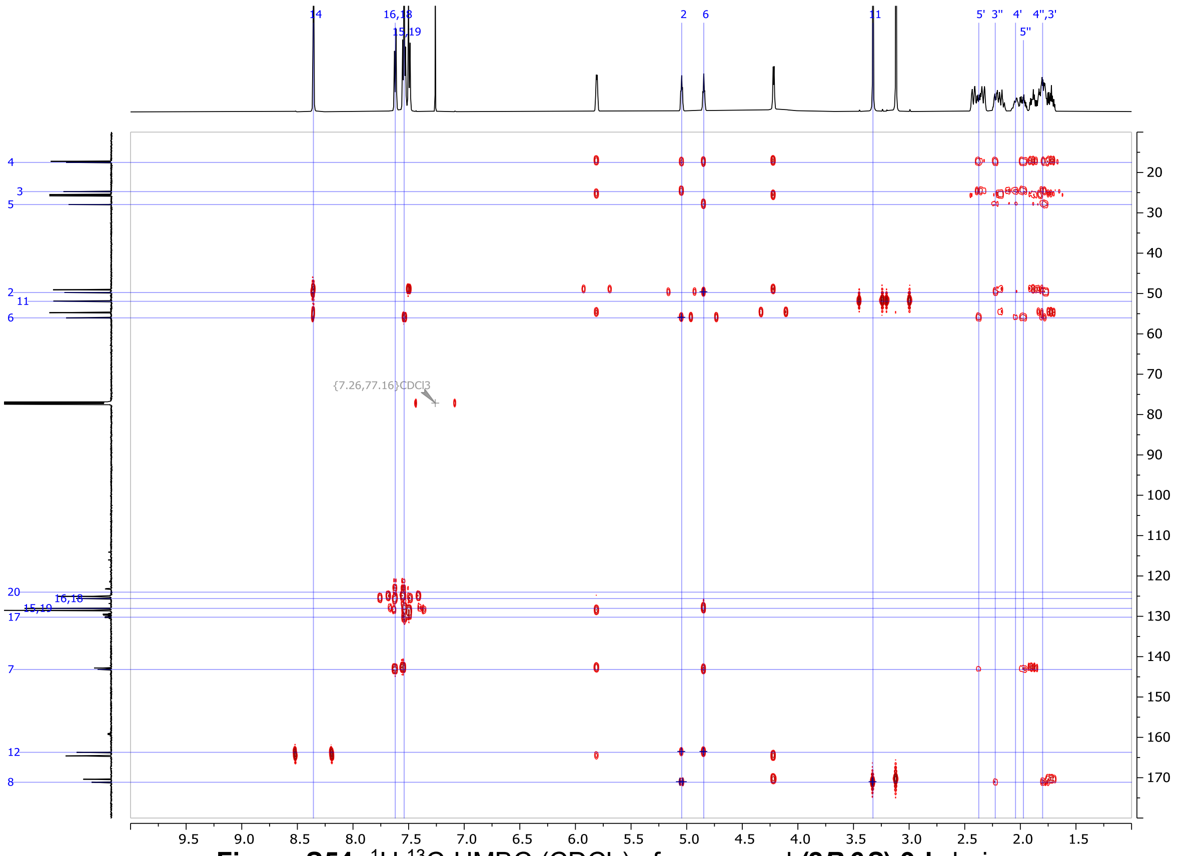

Figure S54: ^{1}H,^{13}C-HMBC ($CDCl_3$) of compound **(2*R*,6*S*)-9d** chair.

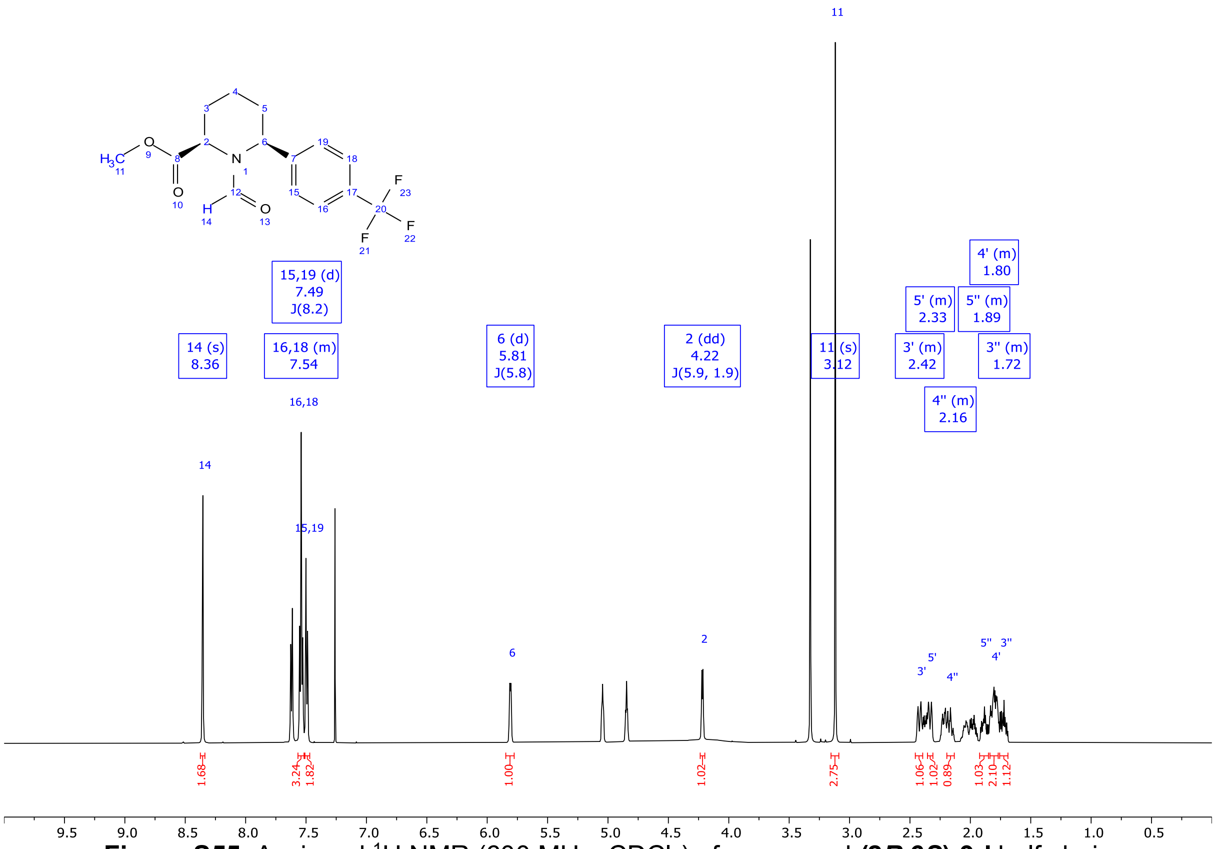

Figure S55: Assigned ^{1}H NMR (600 MHz, $CDCl_3$) of compound **(2*R*,6*S*)-9d** half-chair.

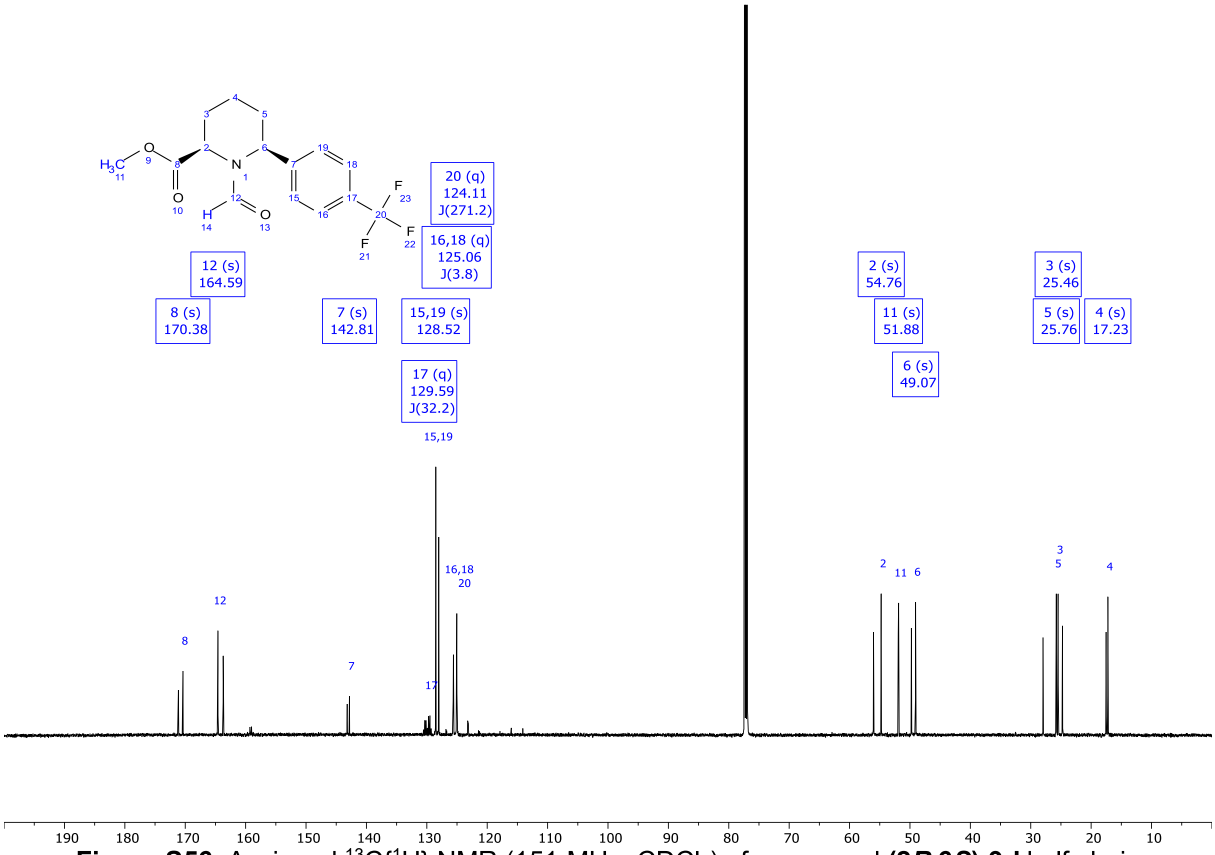

Figure S56: Assigned $^{13}C\{^{1}H\}$ NMR (151 MHz, $CDCl_3$) of compound **(2*R*,6*S*)-9d** half-chair.

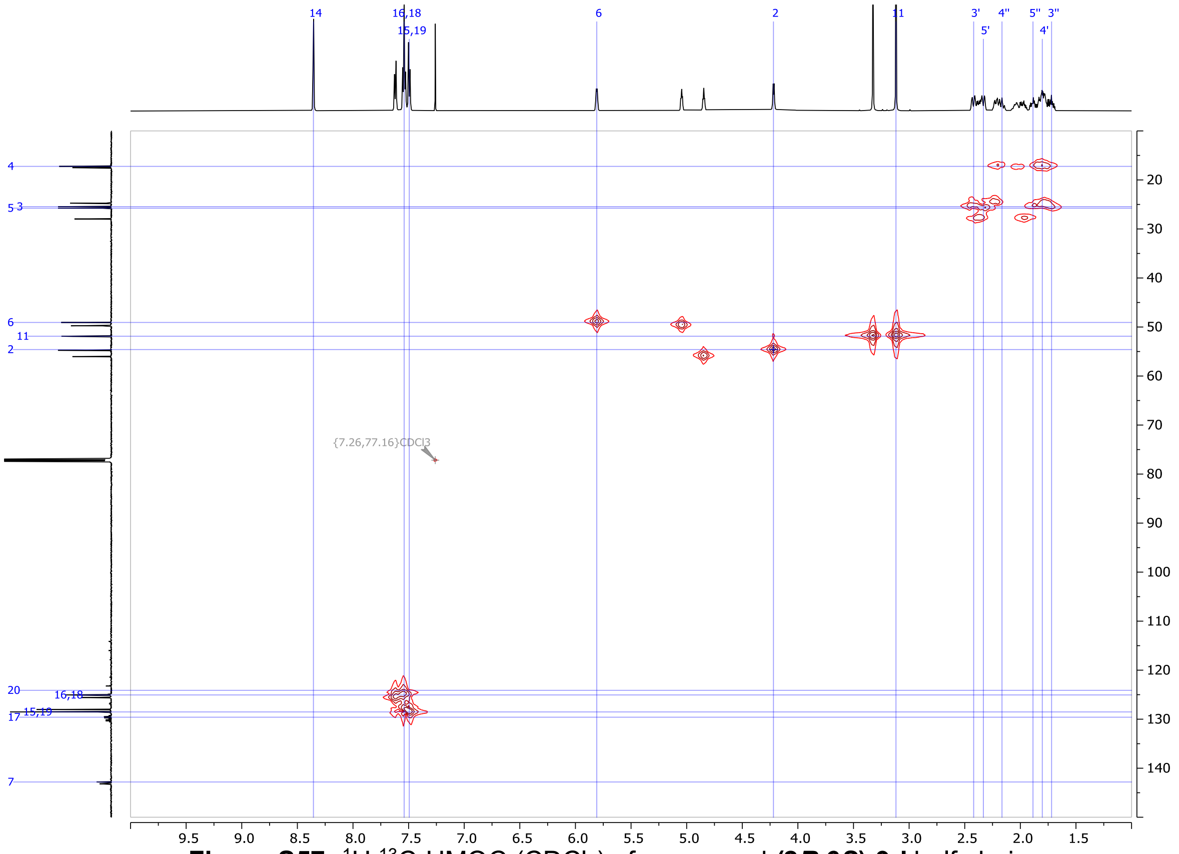

Figure S57: [1]H,[13]C-HMQC (CDCl3) of compound **(2*R*,6*S*)-9d** half-chair.

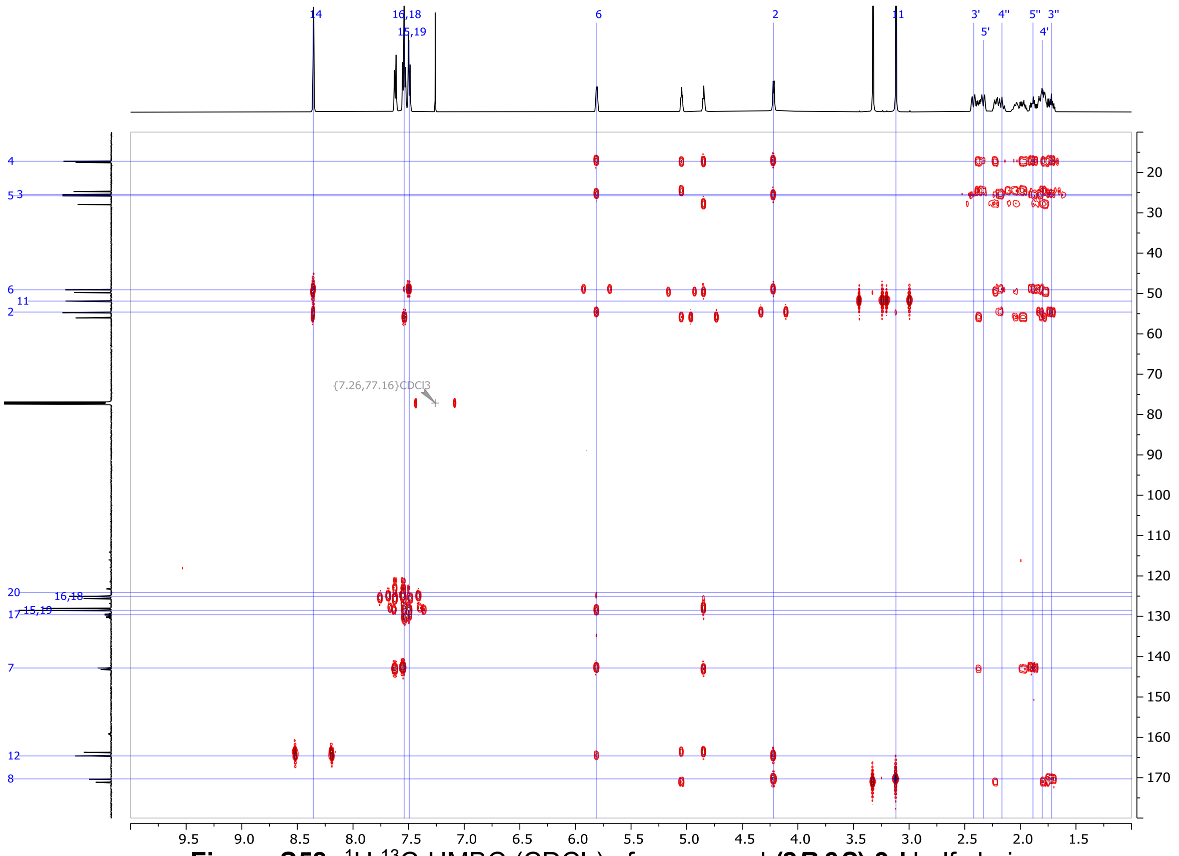

Figure S58: ^{1}H,^{13}C-HMBC (CDCl3) of compound **(2*R*,6*S*)-9d** half-chair.

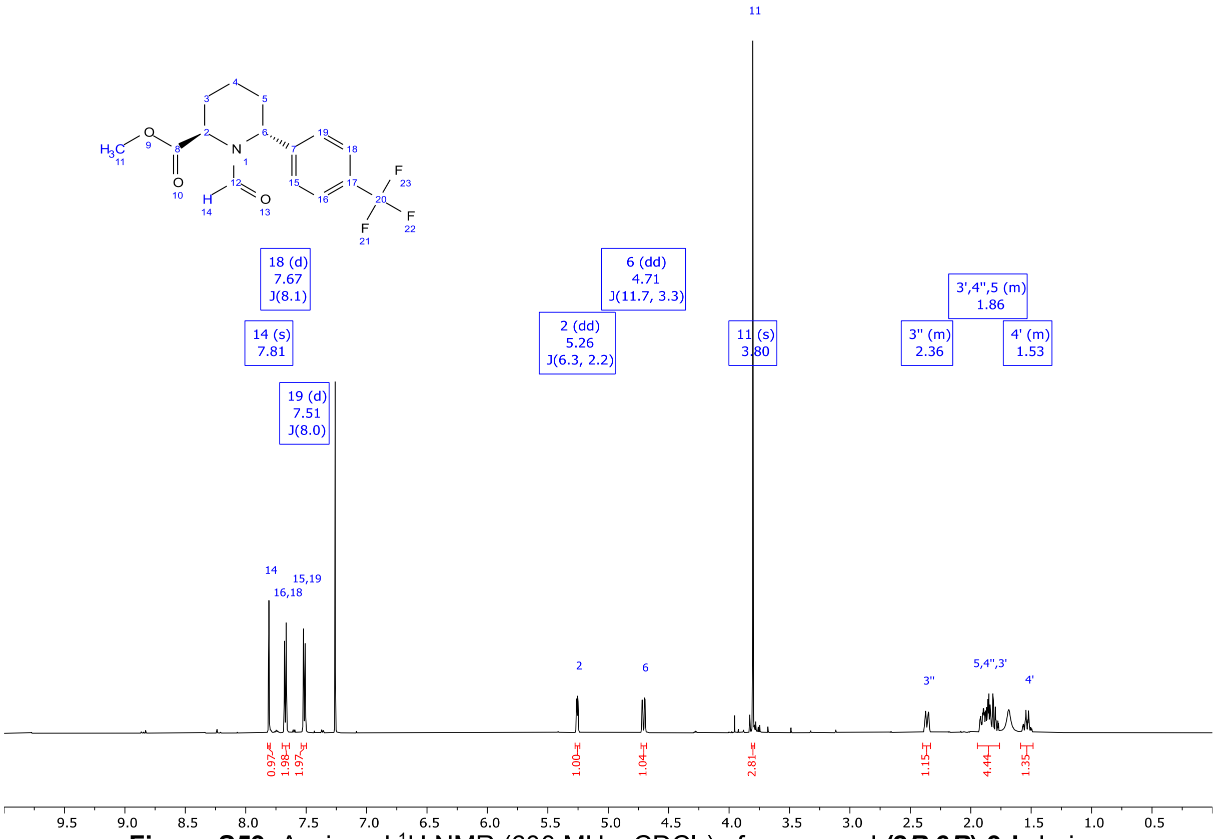

Figure S59: Assigned ^{1}H NMR (600 MHz, $CDCl_3$) of compound **(2*R*,6*R*)-9d** chair.

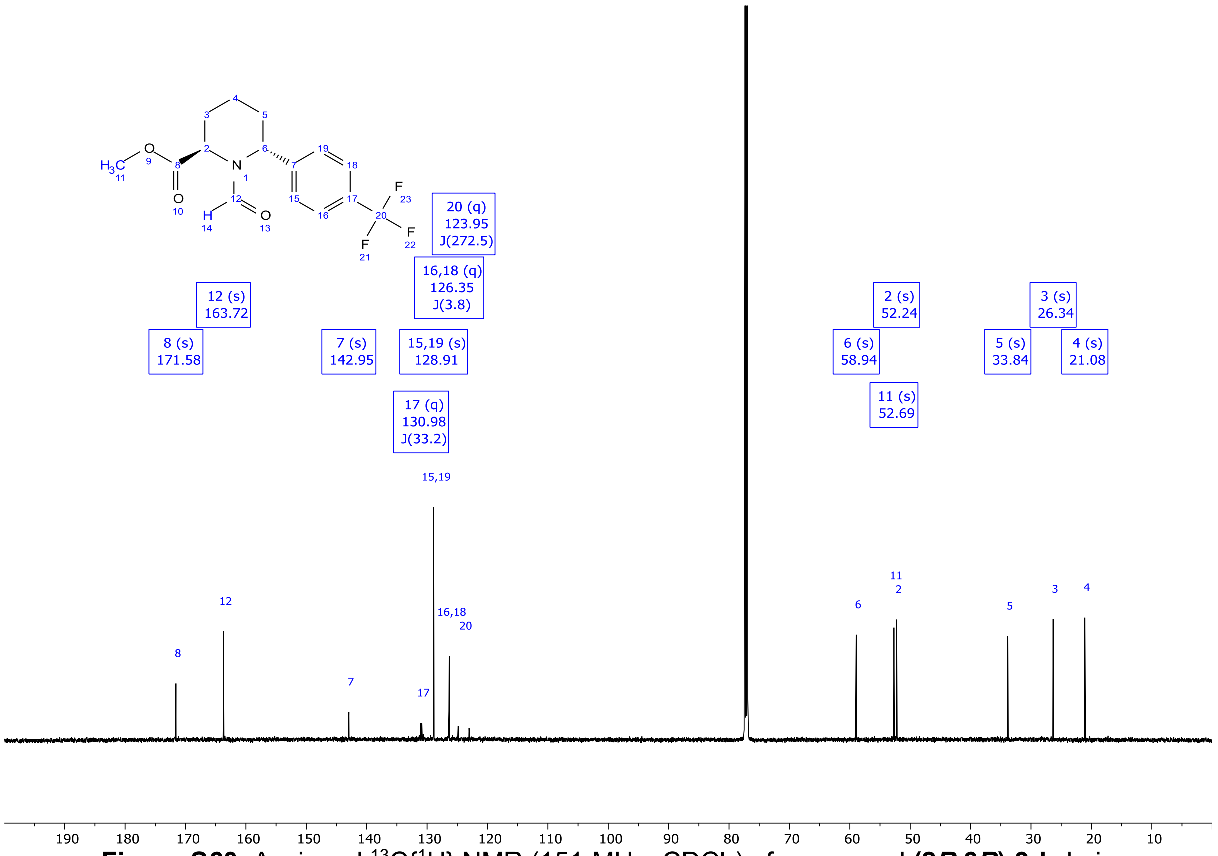

Figure S60: Assigned $^{13}C\{^{1}H\}$ NMR (151 MHz, $CDCl_3$) of compound **(2*R*,6*R*)-9d** chair.

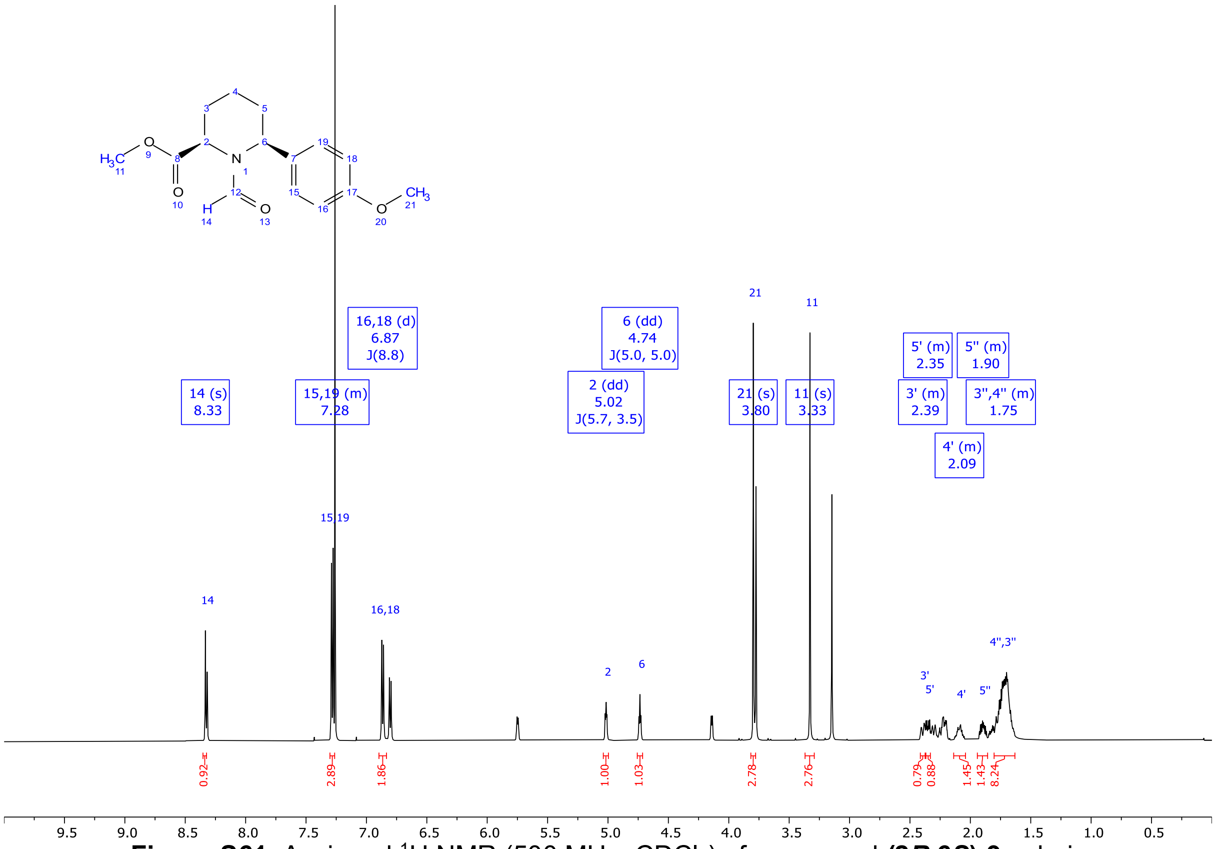

Figure S61: Assigned ^{1}H NMR (500 MHz, $CDCl_3$) of compound **(2*R*,6*S*)-9e** chair.

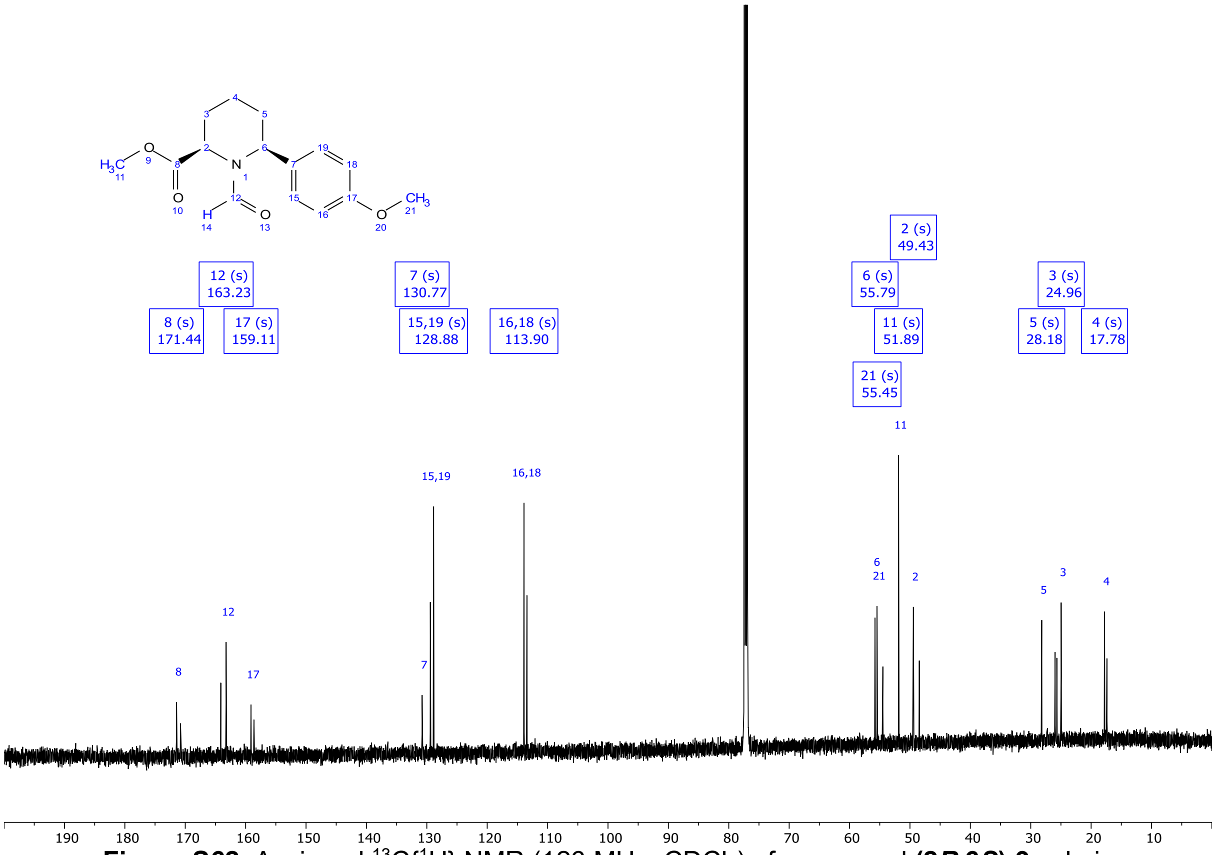

Figure S62: Assigned $^{13}C\{^{1}H\}$ NMR (126 MHz, $CDCl_3$) of compound **(2*R*,6*S*)-9e** chair.

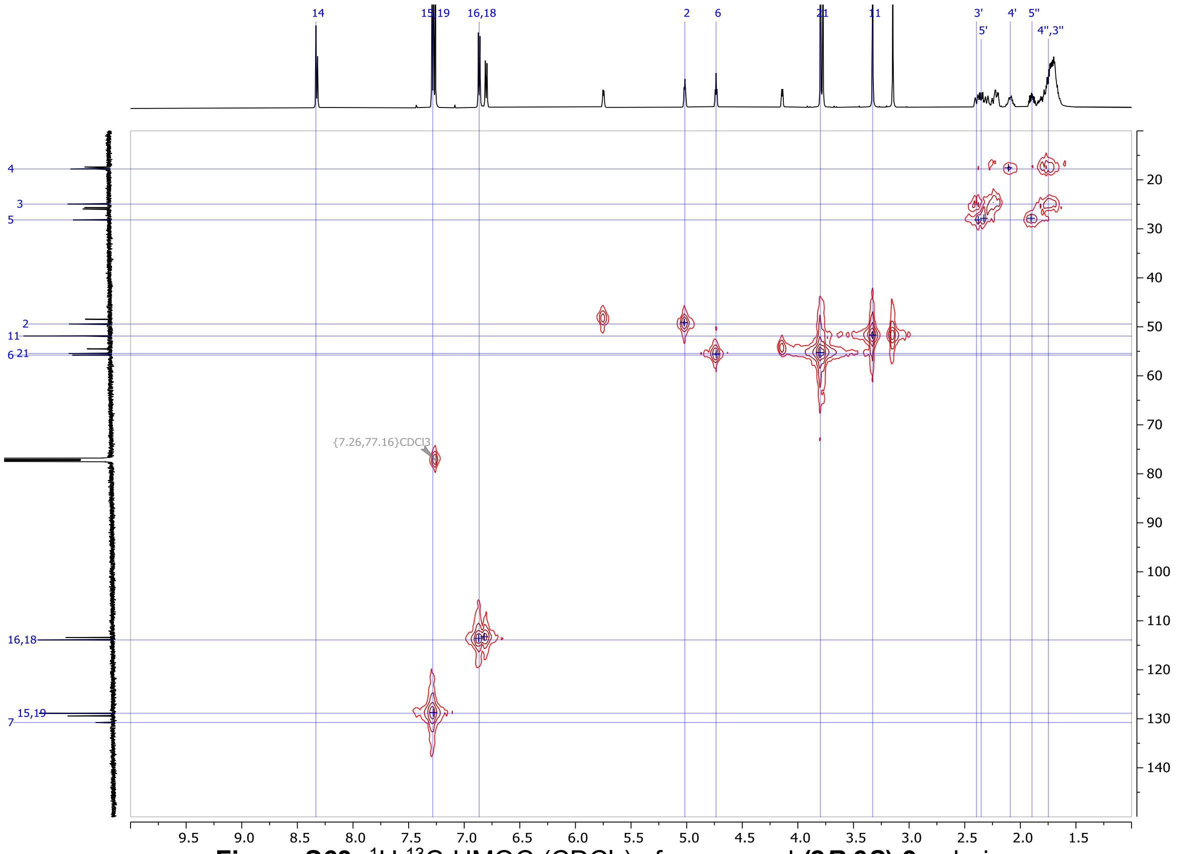

Figure S63: $^{1}H,^{13}C$-HMQC ($CDCl_3$) of compound **(2*R*,6*S*)-9e** chair.

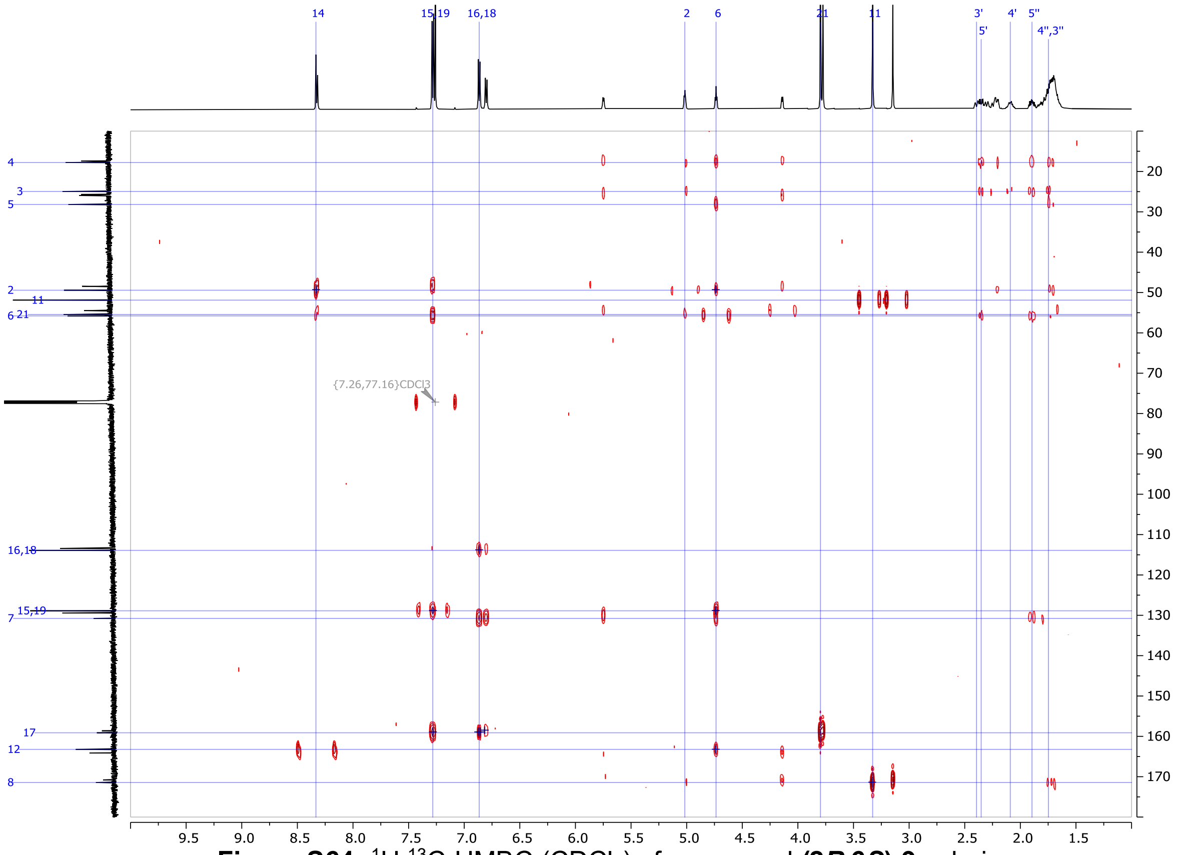

Figure S64: $^{1}H,^{13}C$-HMBC ($CDCl_3$) of compound **(2*R*,6*S*)-9e** chair.

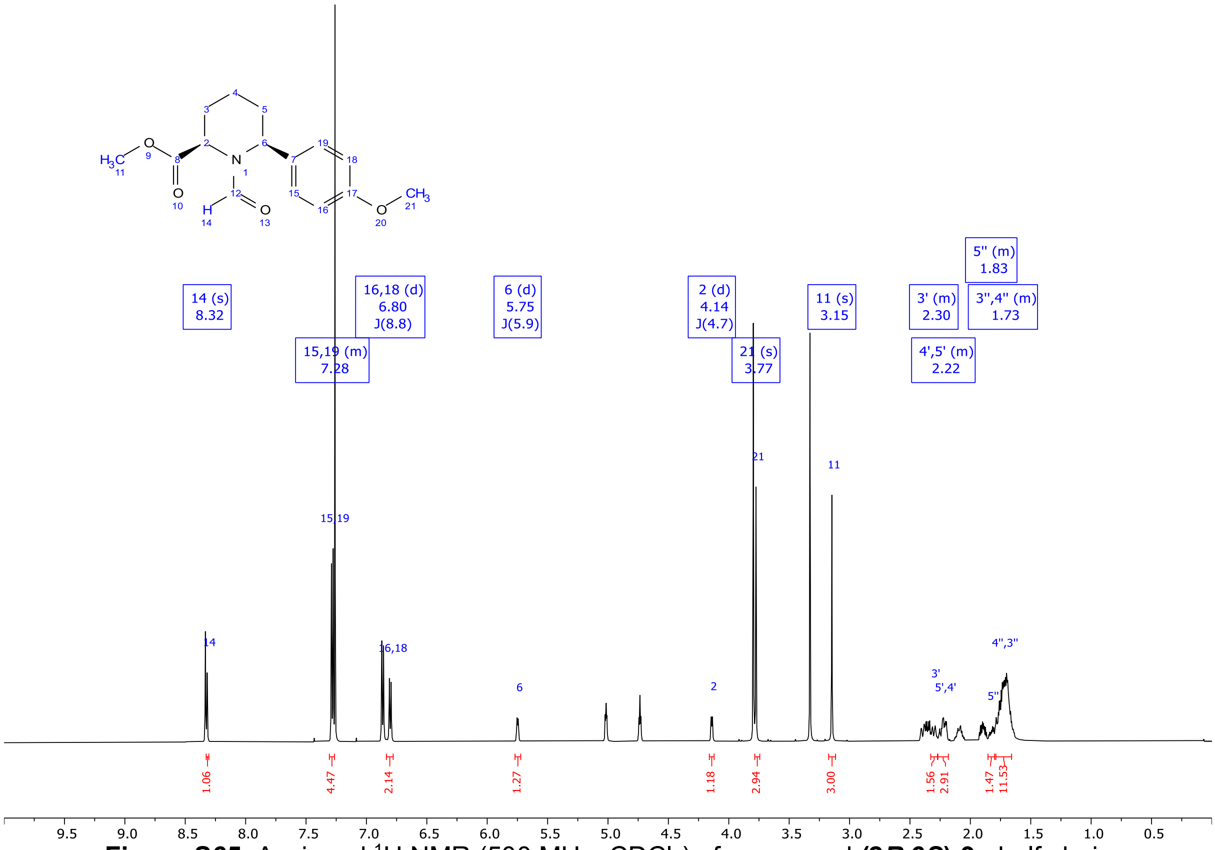

Figure S65: Assigned ^{1}H NMR (500 MHz, $CDCl_3$) of compound **(2*R*,6*S*)-9e** half-chair.

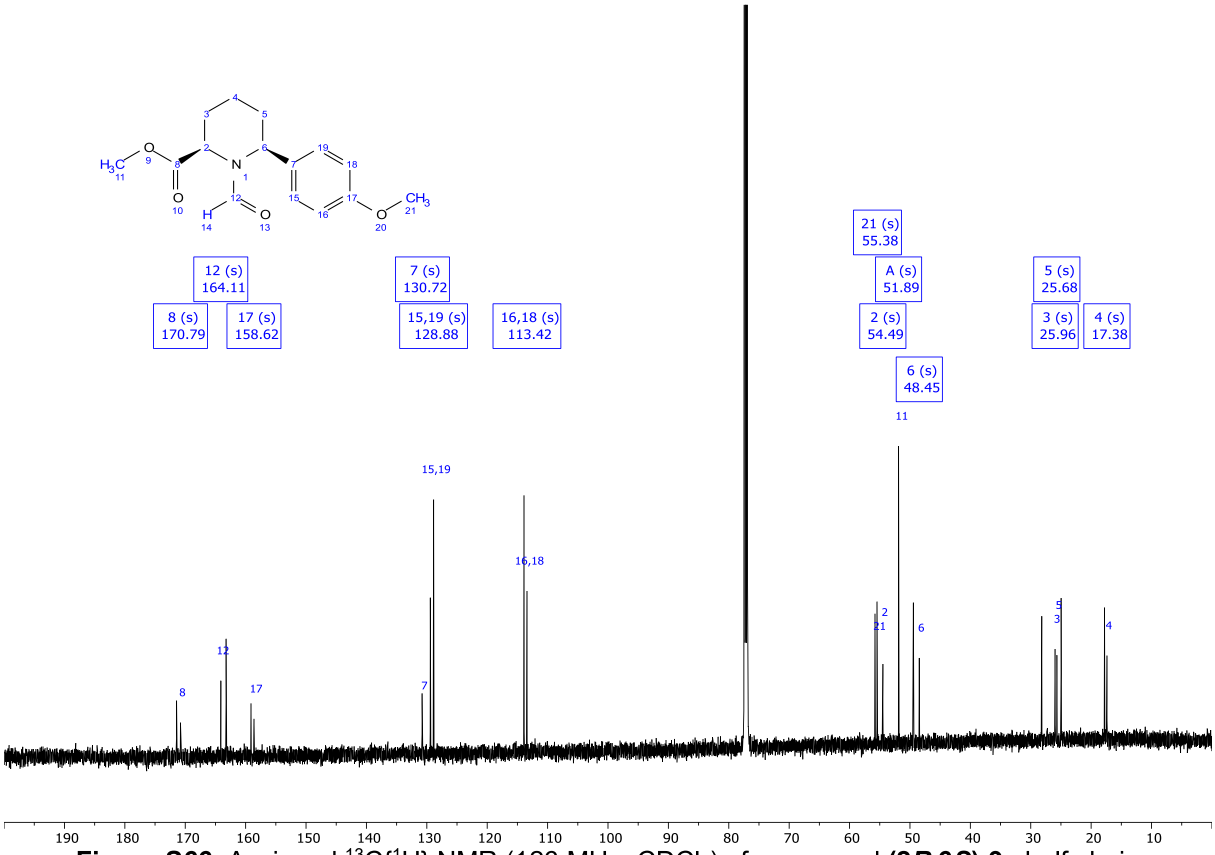

Figure S66: Assigned $^{13}C\{^{1}H\}$ NMR (126 MHz, $CDCl_3$) of compound **(2*R*,6*S*)-9e** half-chair.

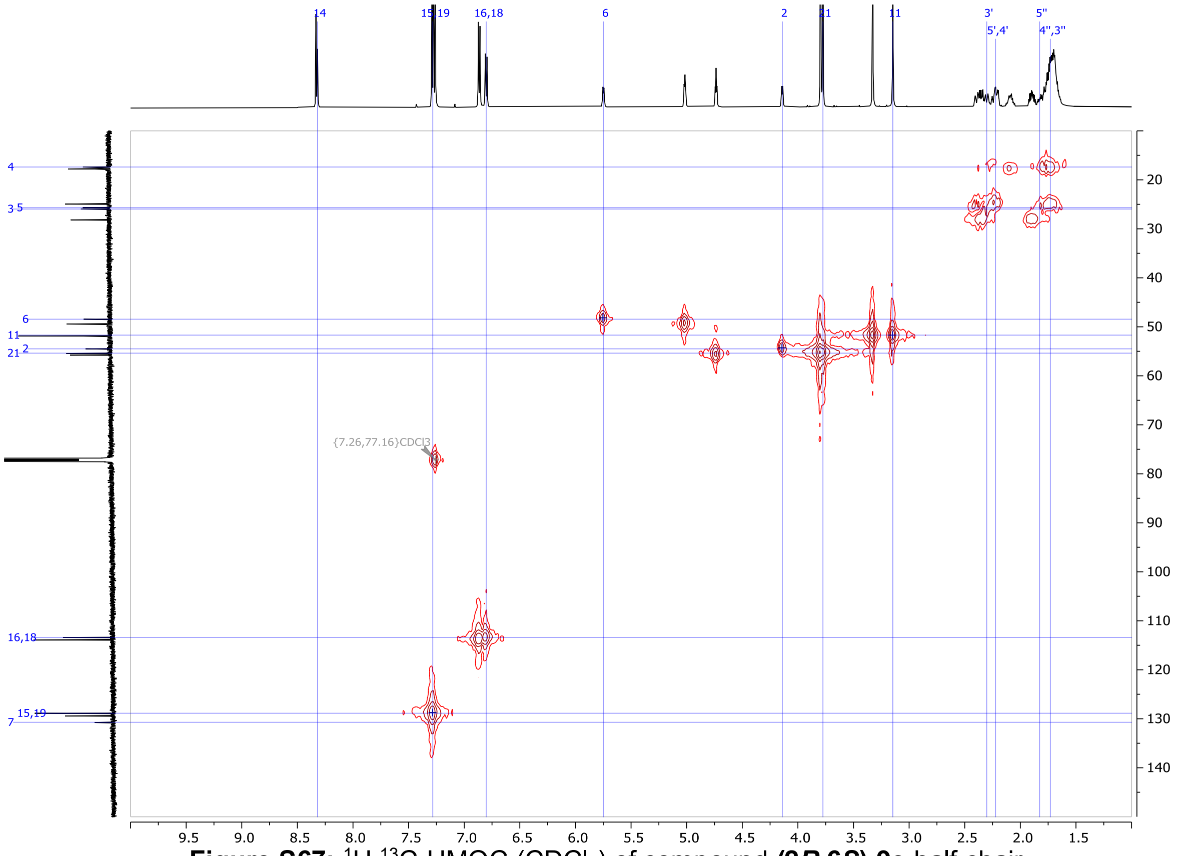

Figure S67: ^{1}H,^{13}C-HMQC ($CDCl_3$) of compound **(2*R*,6*S*)-9e** half-chair.

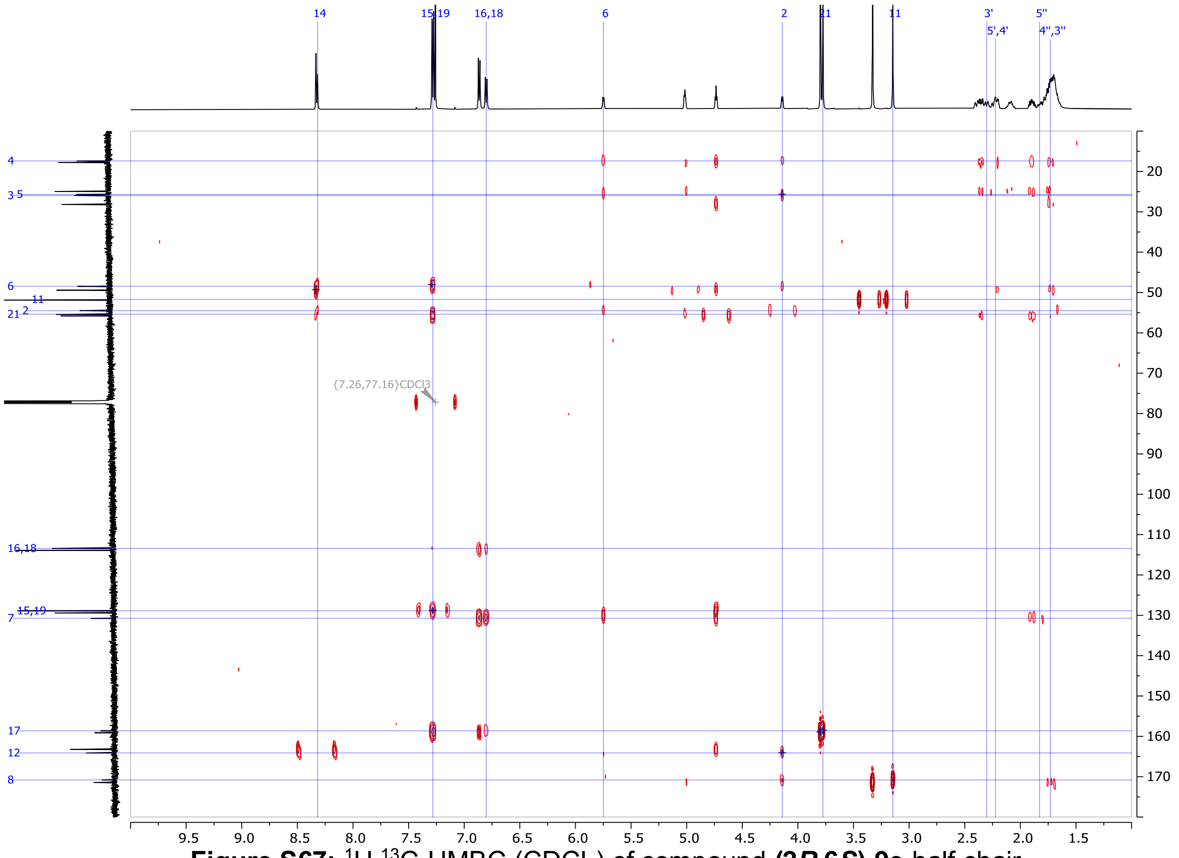

Figure S67: ^{1}H,^{13}C-HMBC ($CDCl_3$) of compound **(2*R*,6*S*)-9e** half-chair.

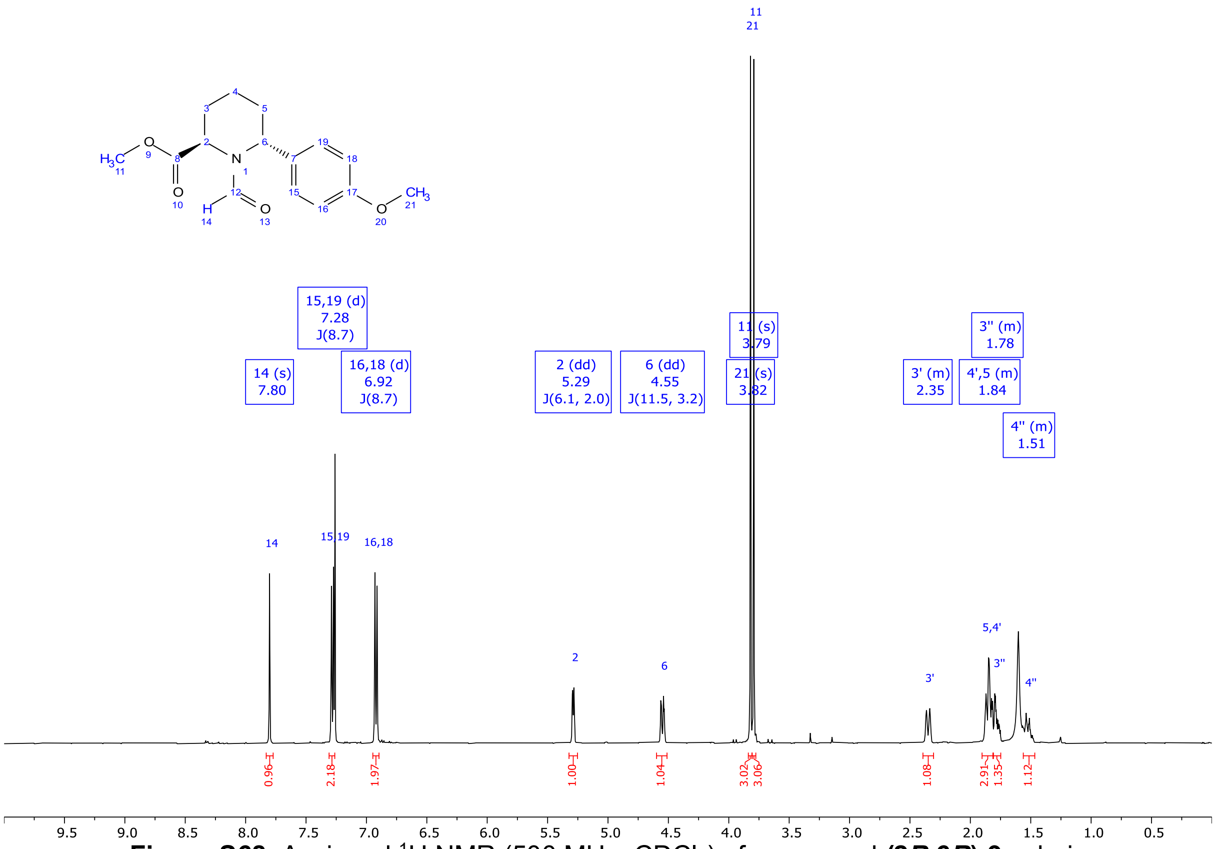

Figure S68: Assigned ^{1}H NMR (500 MHz, $CDCl_3$) of compound **(2*R*,6*R*)-9e** chair.

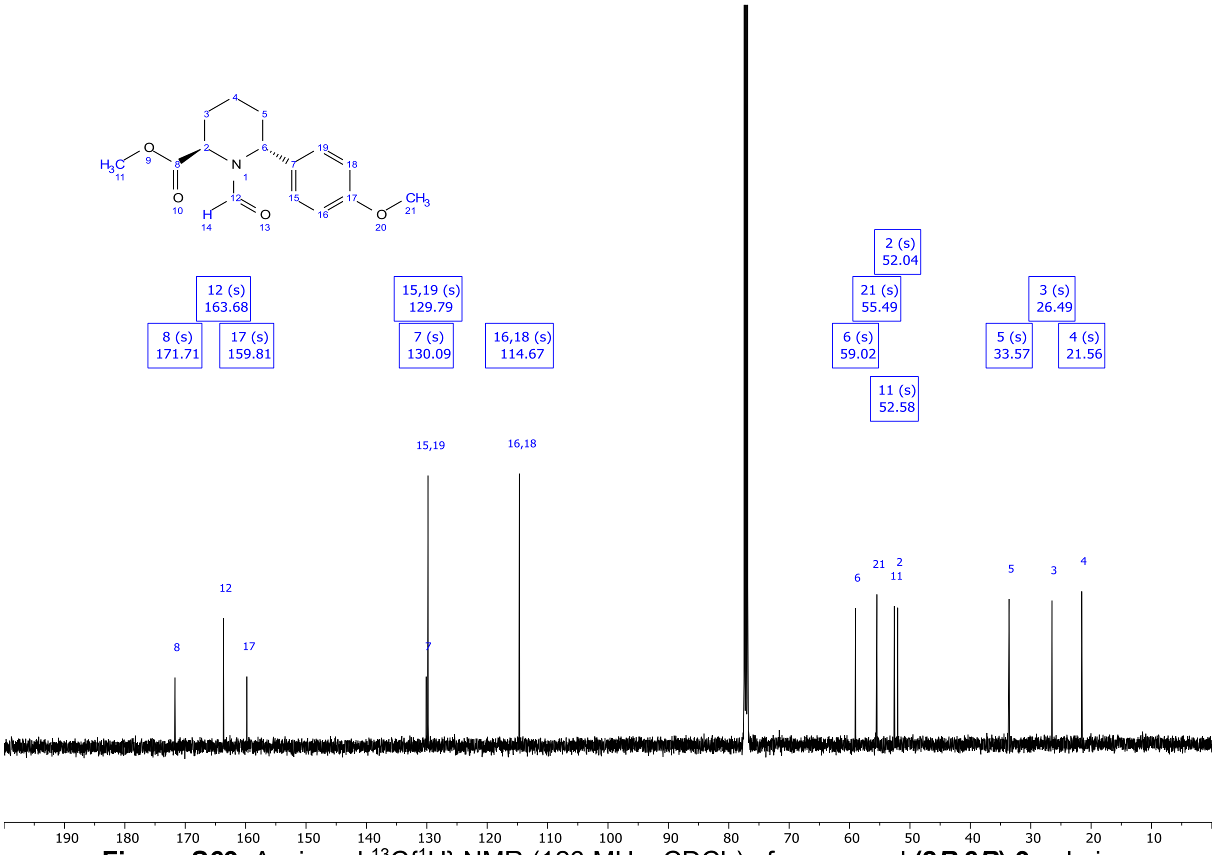

Figure S69: Assigned $^{13}C\{^{1}H\}$ NMR (126 MHz, $CDCl_3$) of compound **(2*R*,6*R*)-9e** chair.

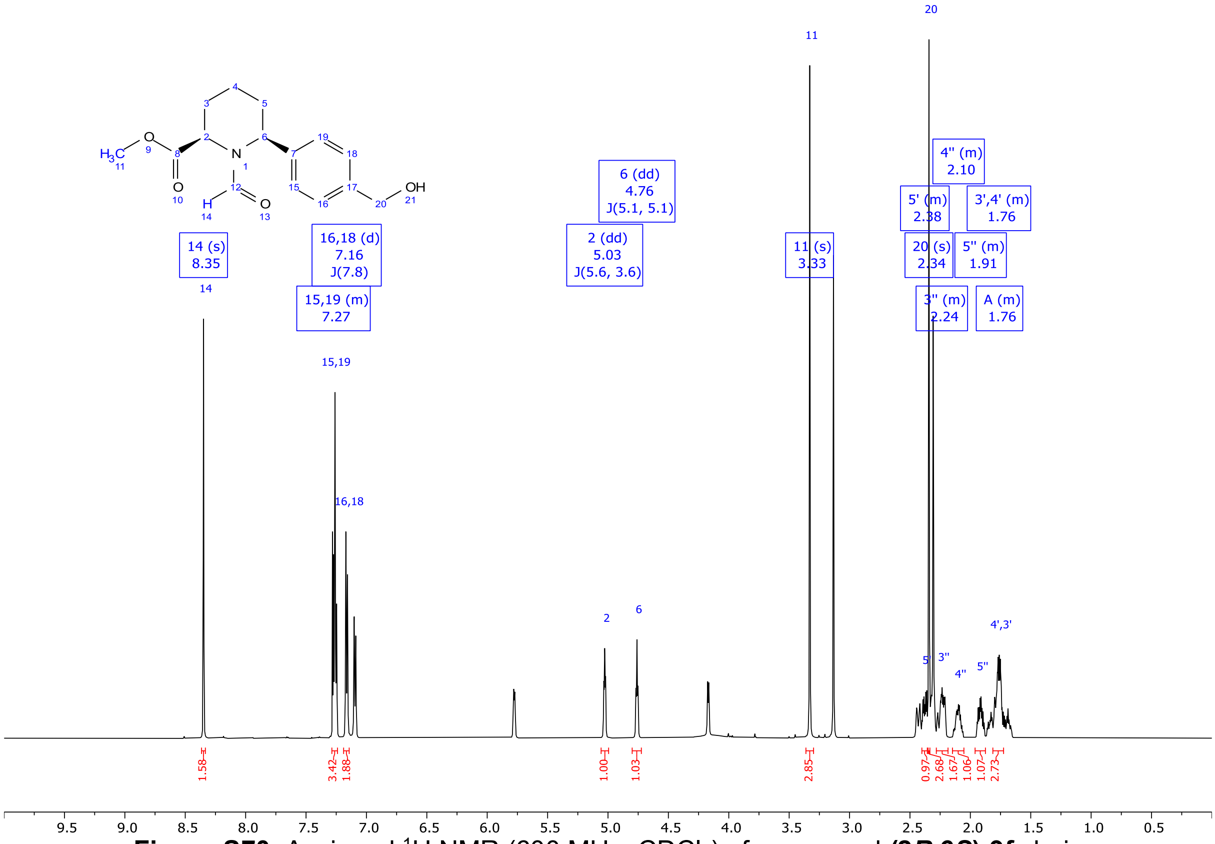

Figure S70: Assigned ^{1}H NMR (600 MHz, $CDCl_3$) of compound **(2*R*,6*S*)-9f** chair.

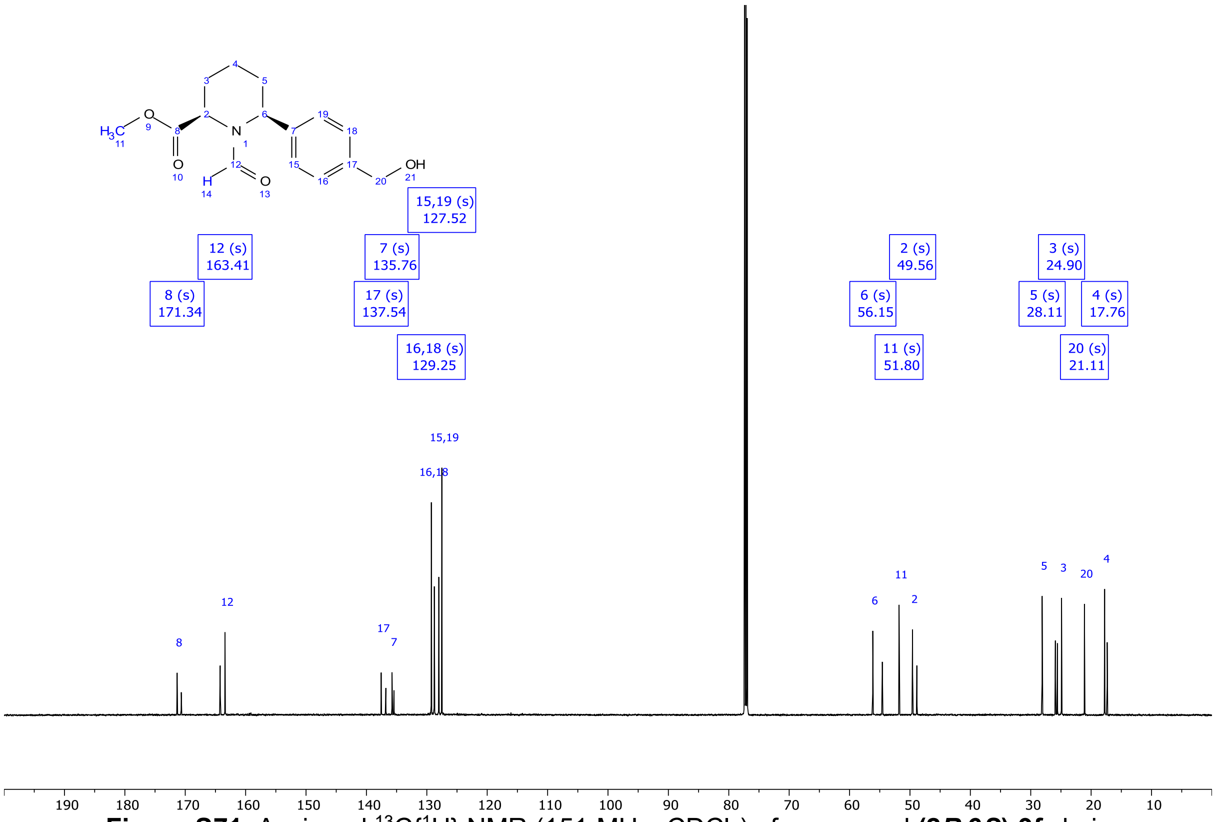

Figure S71: Assigned $^{13}C\{^{1}H\}$ NMR (151 MHz, $CDCl_3$) of compound **(2*R*,6*S*)-9f** chair.

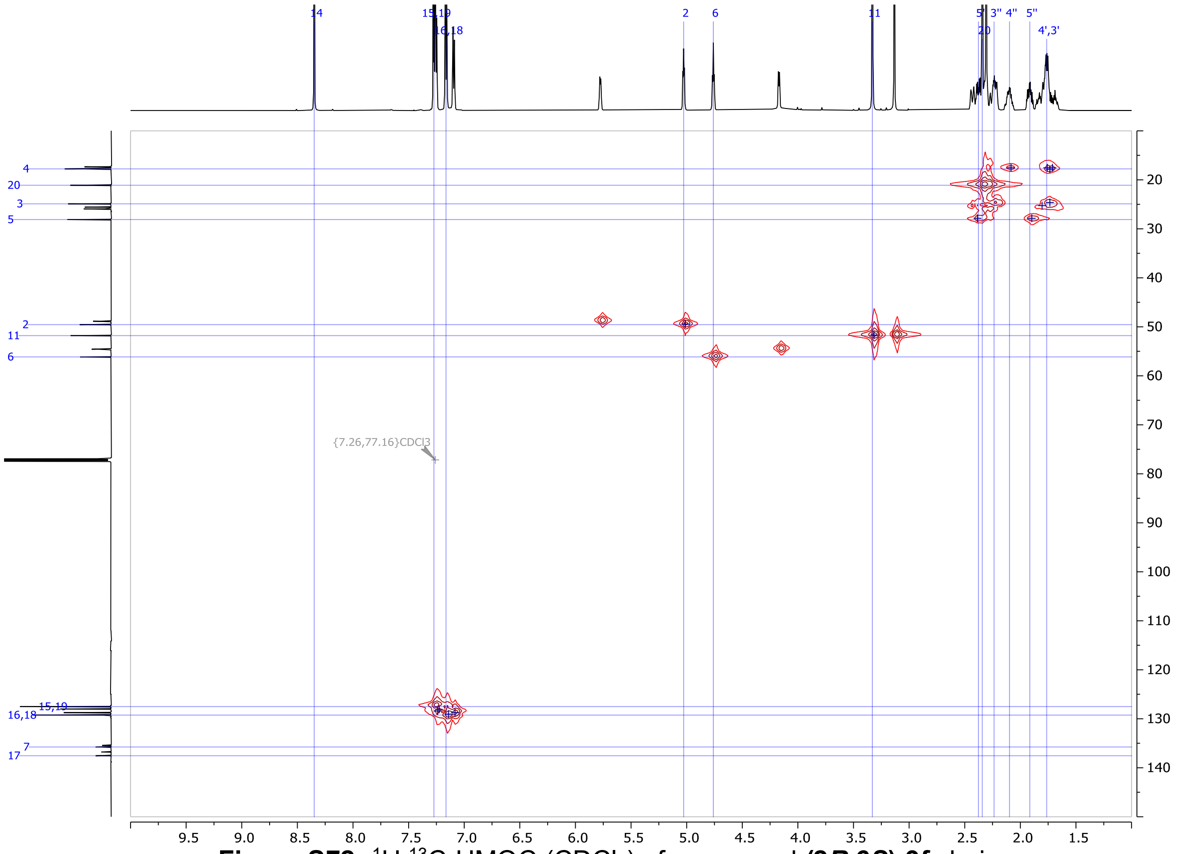

Figure S72: ^{1}H,^{13}C-HMQC (CDCl₃) of compound **(2*R*,6*S*)-9f** chair.

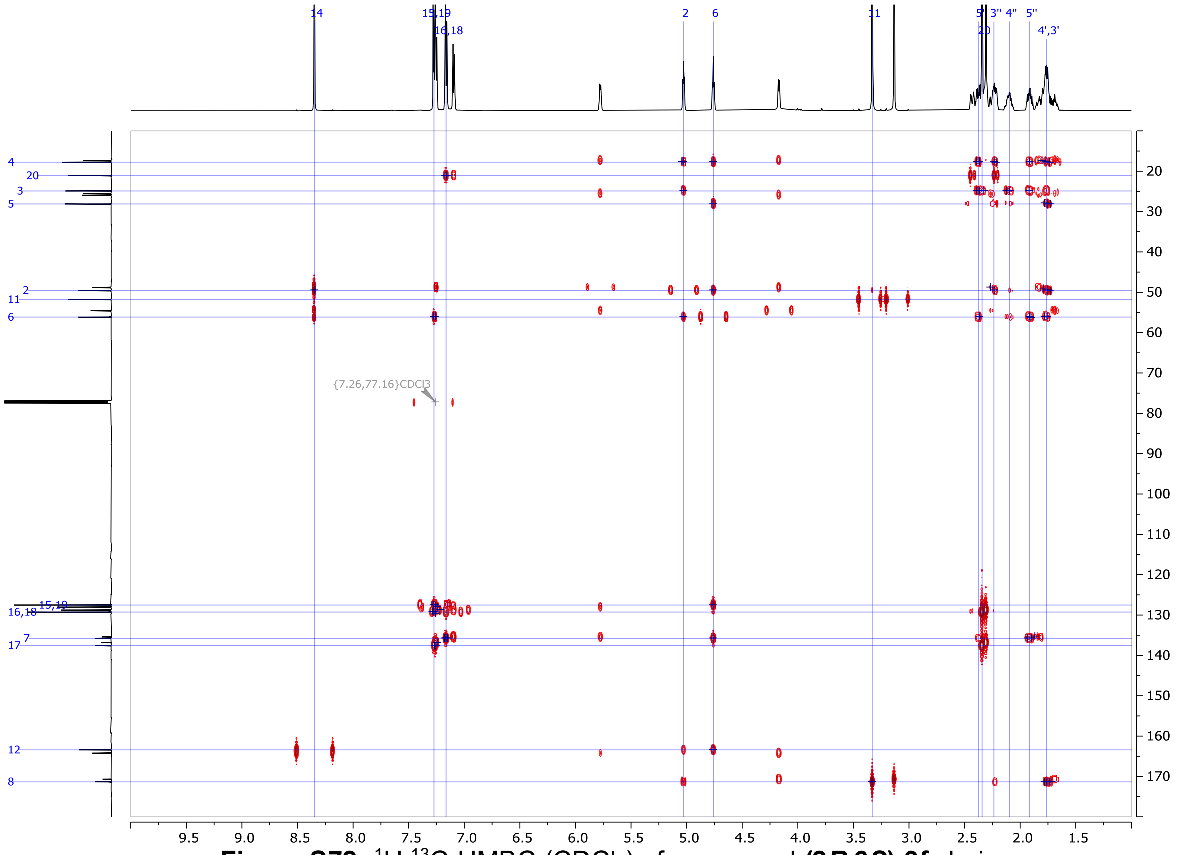

Figure S72: ^{1}H,^{13}C-HMBC (CDCl₃) of compound **(2*R*,6*S*)-9f** chair.

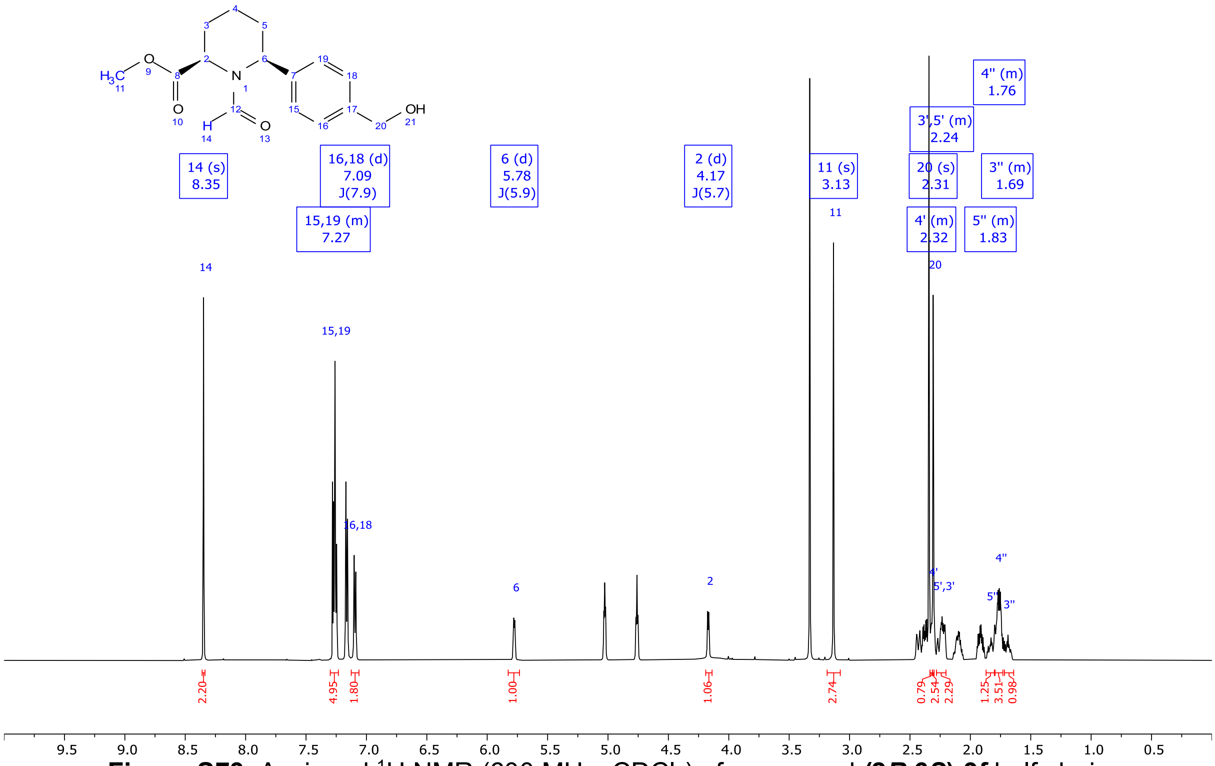

Figure S73: Assigned ^{1}H NMR (600 MHz, $CDCl_3$) of compound **(2*R*,6*S*)-9f** half-chair.

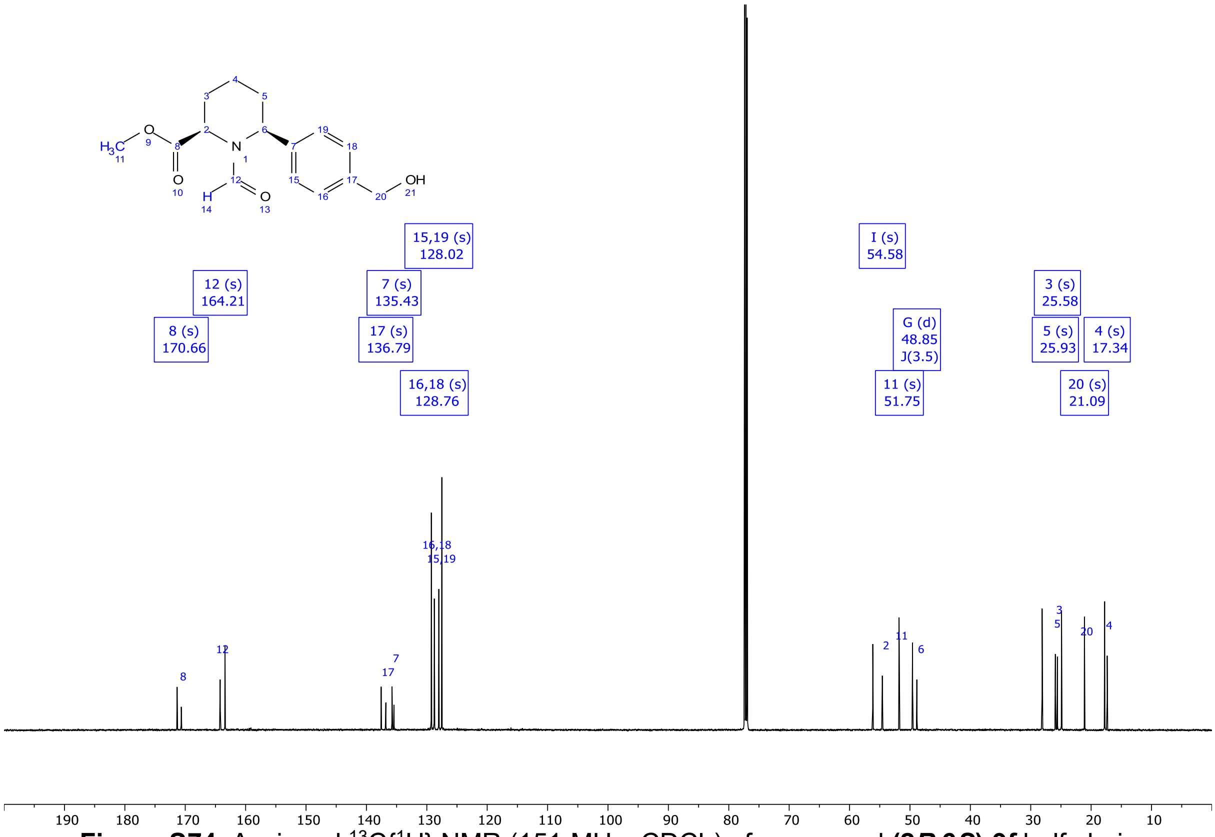

Figure S74: Assigned ^{13}C{^{1}H} NMR (151 MHz, $CDCl_3$) of compound **(2*R*,6*S*)-9f** half-chair.

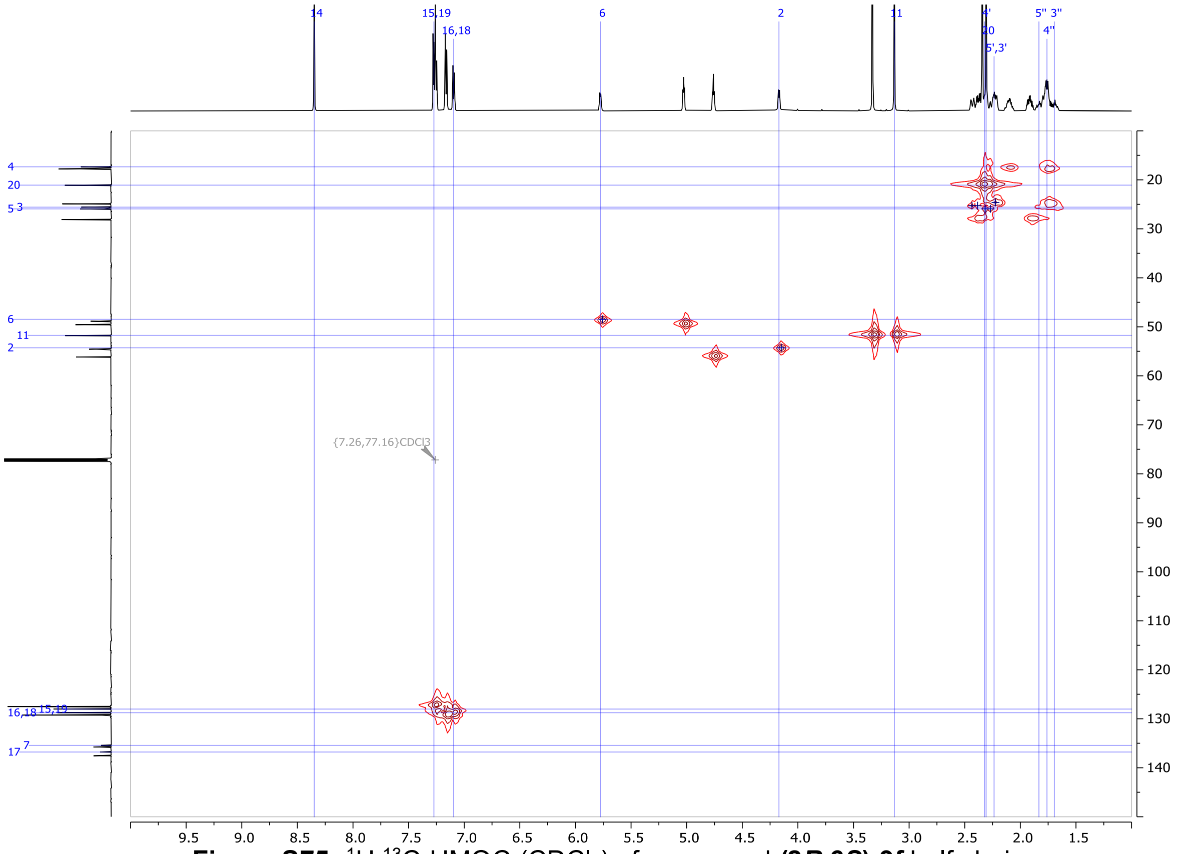

Figure S75: $^{1}H,^{13}C$-HMQC ($CDCl_3$) of compound **(2*R*,6*S*)-9f** half-chair.

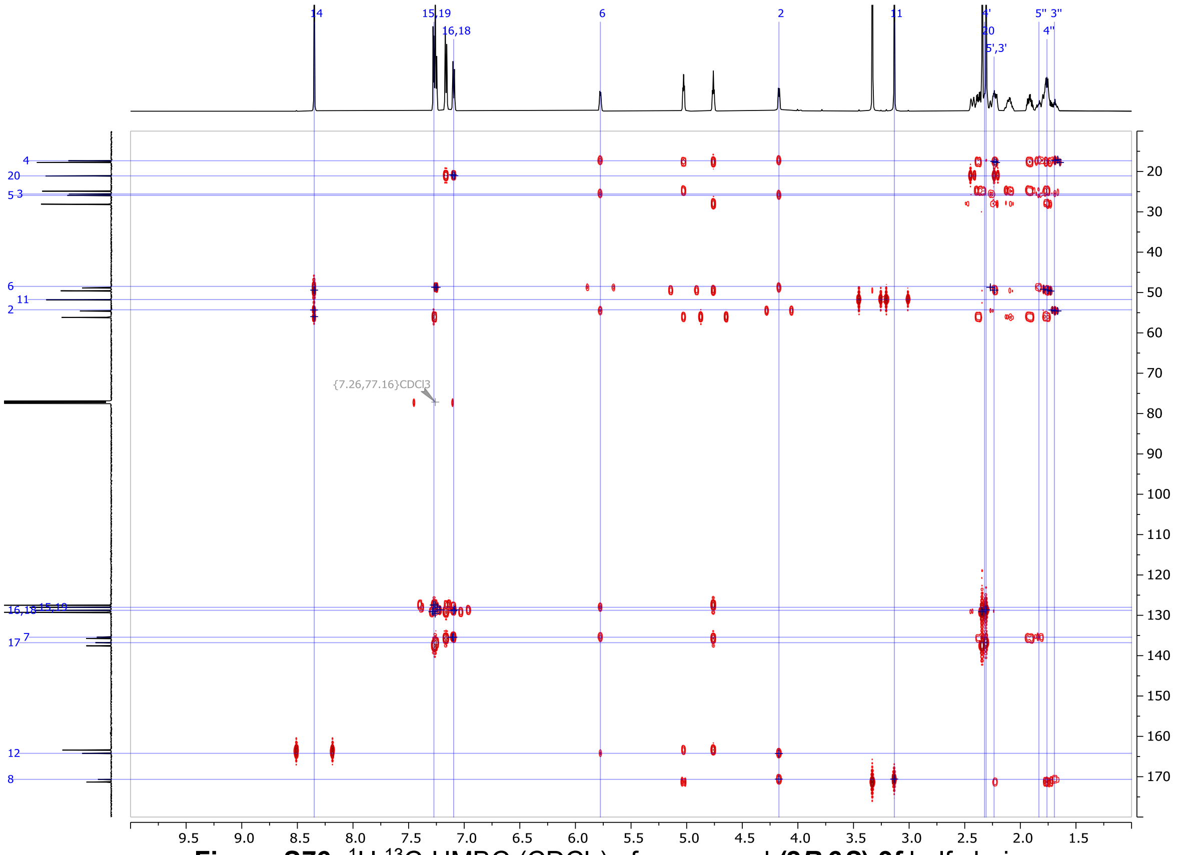

Figure S76: $^{1}H,^{13}C$-HMBC ($CDCl_3$) of compound **(2*R*,6*S*)-9f** half-chair.

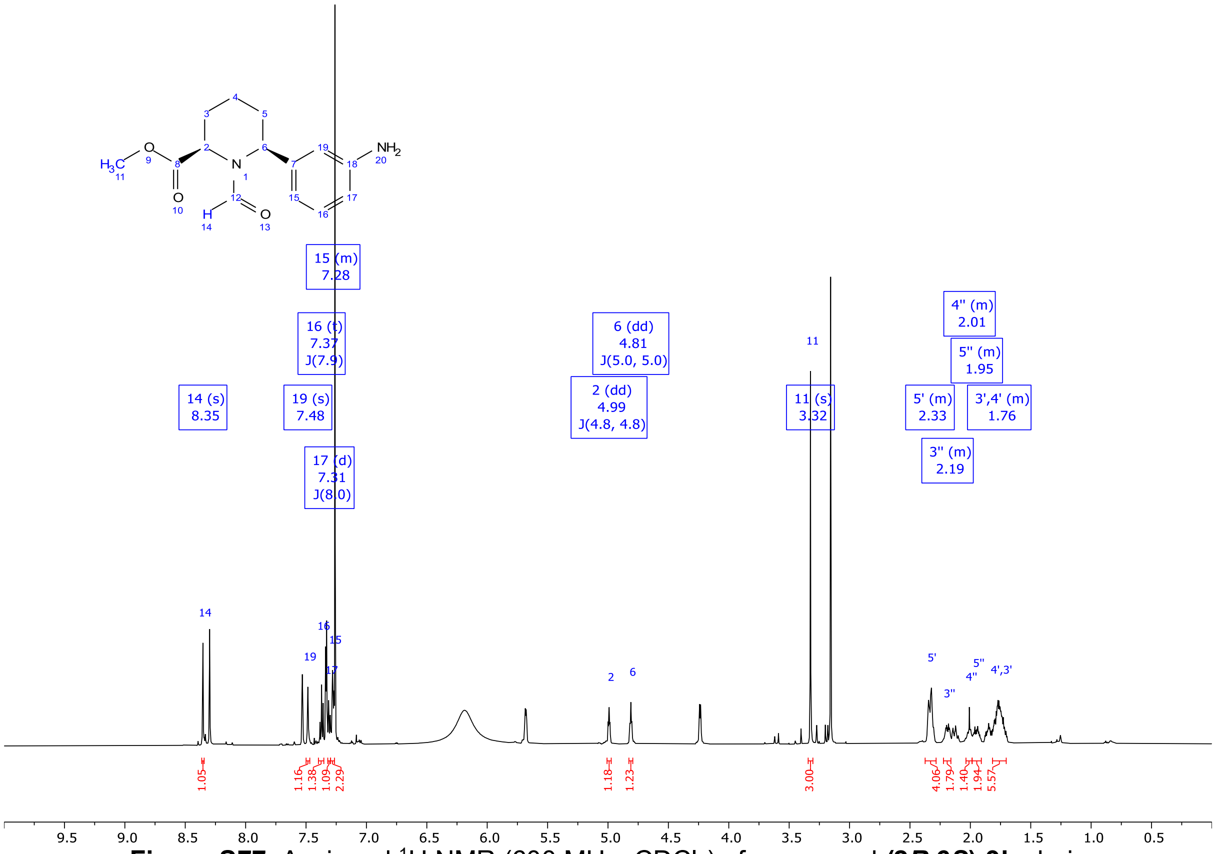

Figure S77: Assigned ^{1}H NMR (600 MHz, CDCl3) of compound **(2*R*,6*S*)-9h** chair.

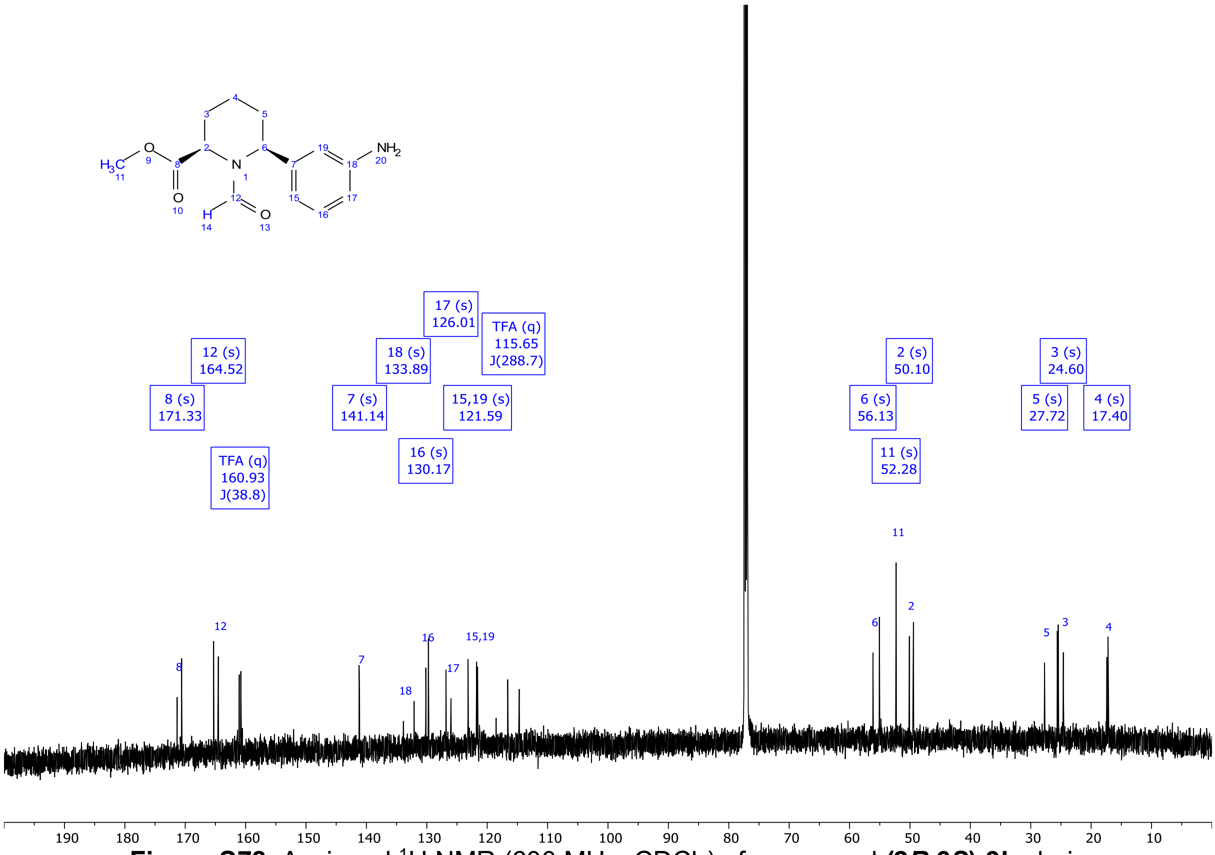

Figure S78: Assigned ^{1}H NMR (600 MHz, CDCl3) of compound **(2*R*,6*S*)-9h** chair.

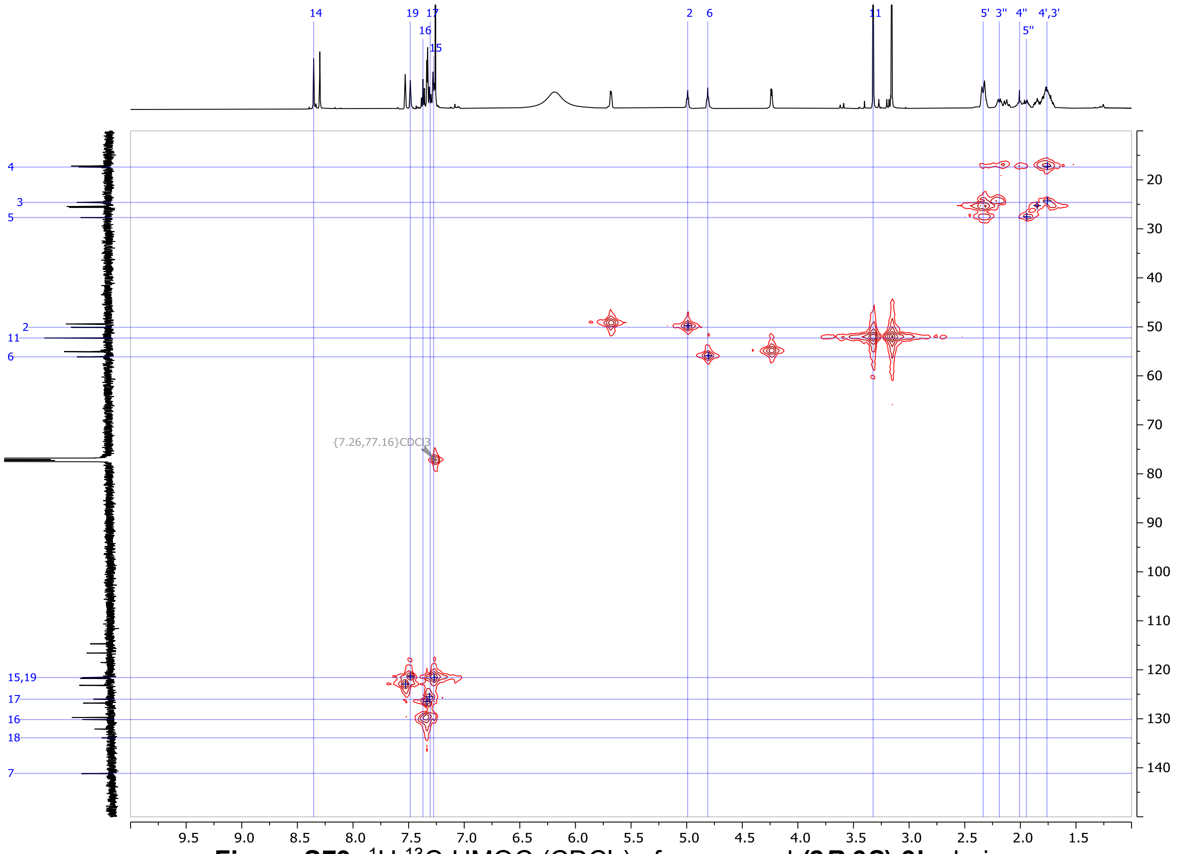

Figure S79: $^{1}H,^{13}C$-HMQC ($CDCl_3$) of compound **(2*R*,6*S*)-9h** chair.

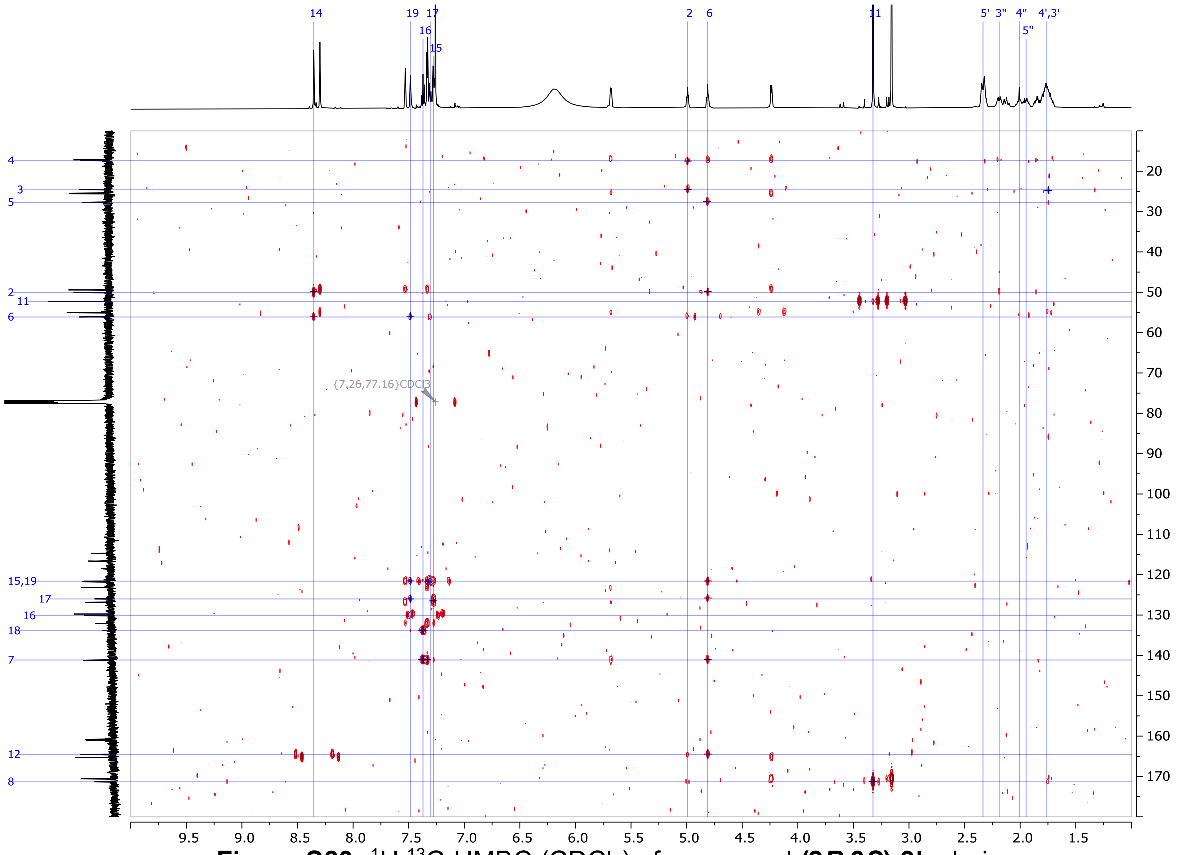

Figure S80: $^{1}H,^{13}C$-HMBC ($CDCl_3$) of compound **(2*R*,6*S*)-9h** chair.

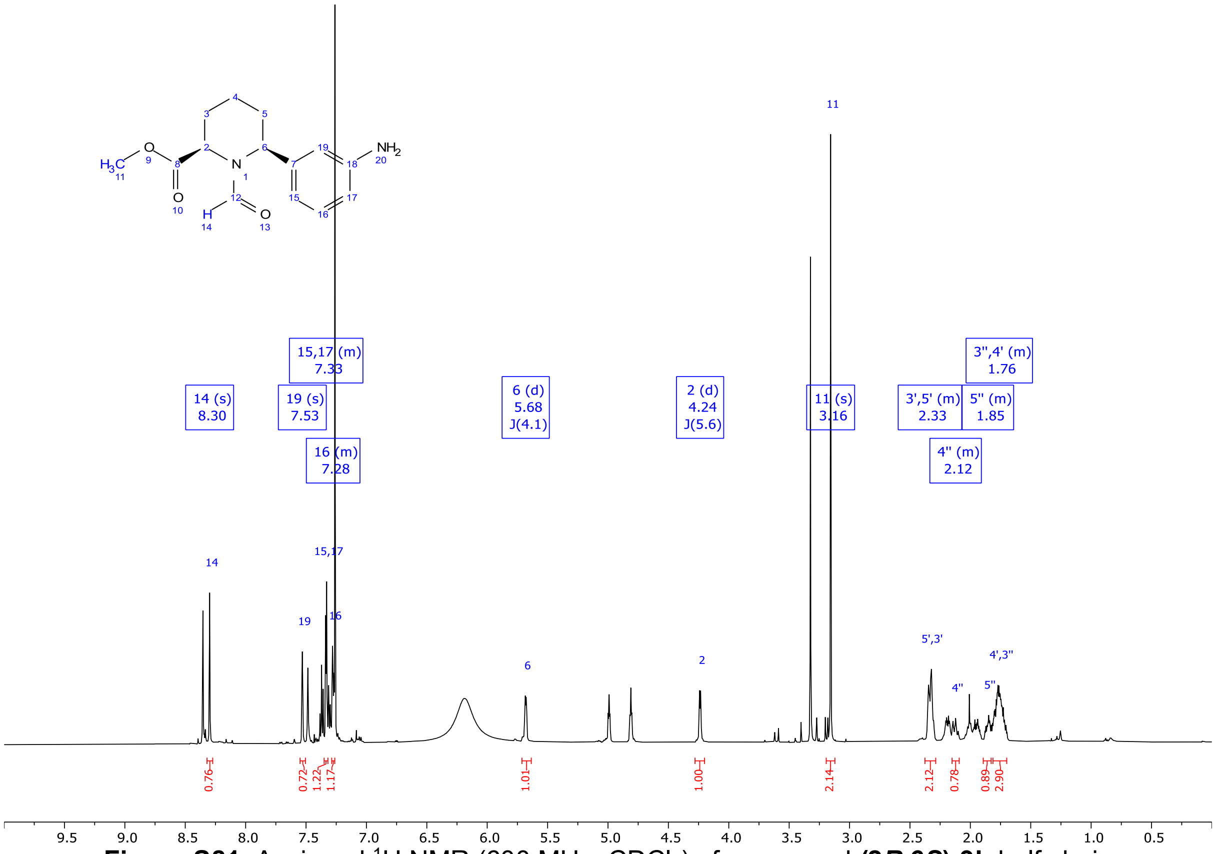

Figure S81: Assigned ^{1}H NMR (600 MHz, $CDCl_3$) of compound **(2*R*,6*S*)-9h** half-chair.

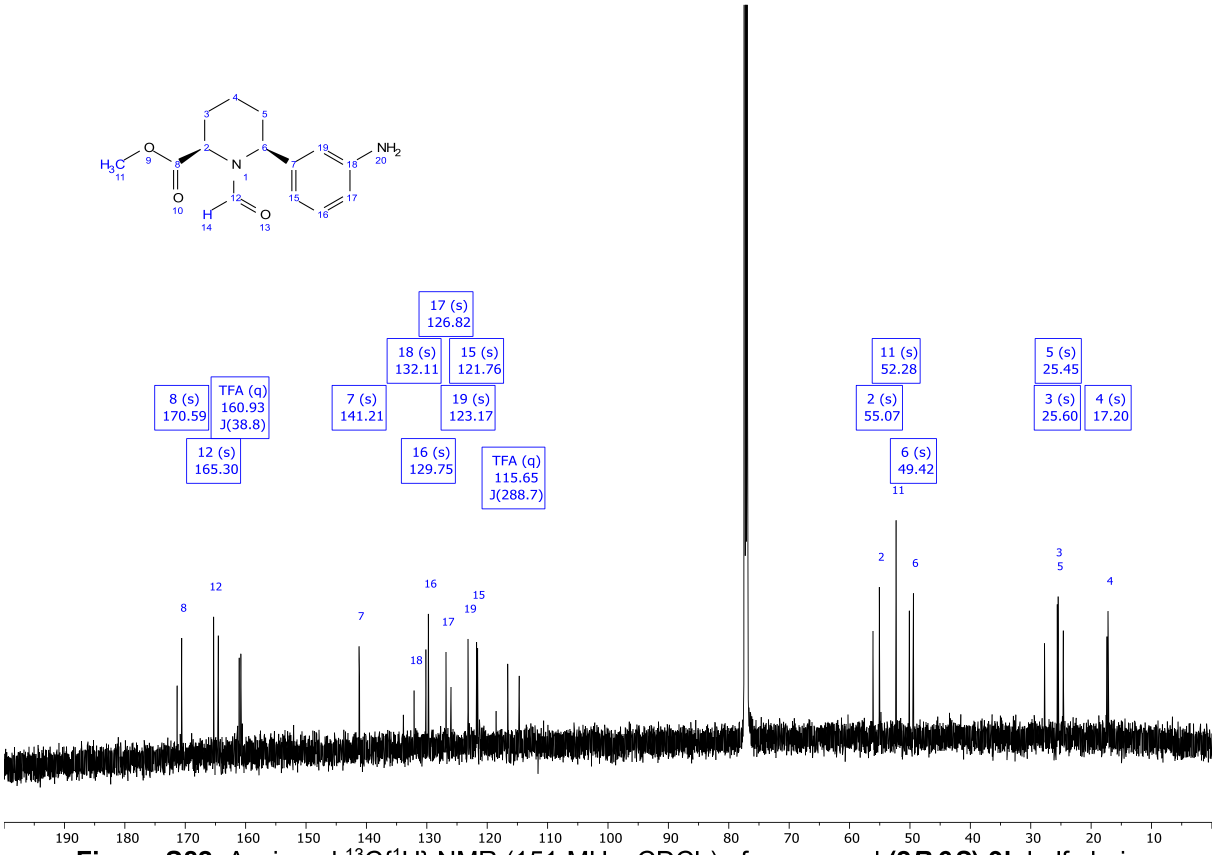

Figure S82: Assigned $^{13}C\{^{1}H\}$ NMR (151 MHz, $CDCl_3$) of compound **(2*R*,6*S*)-9h** half-chair.

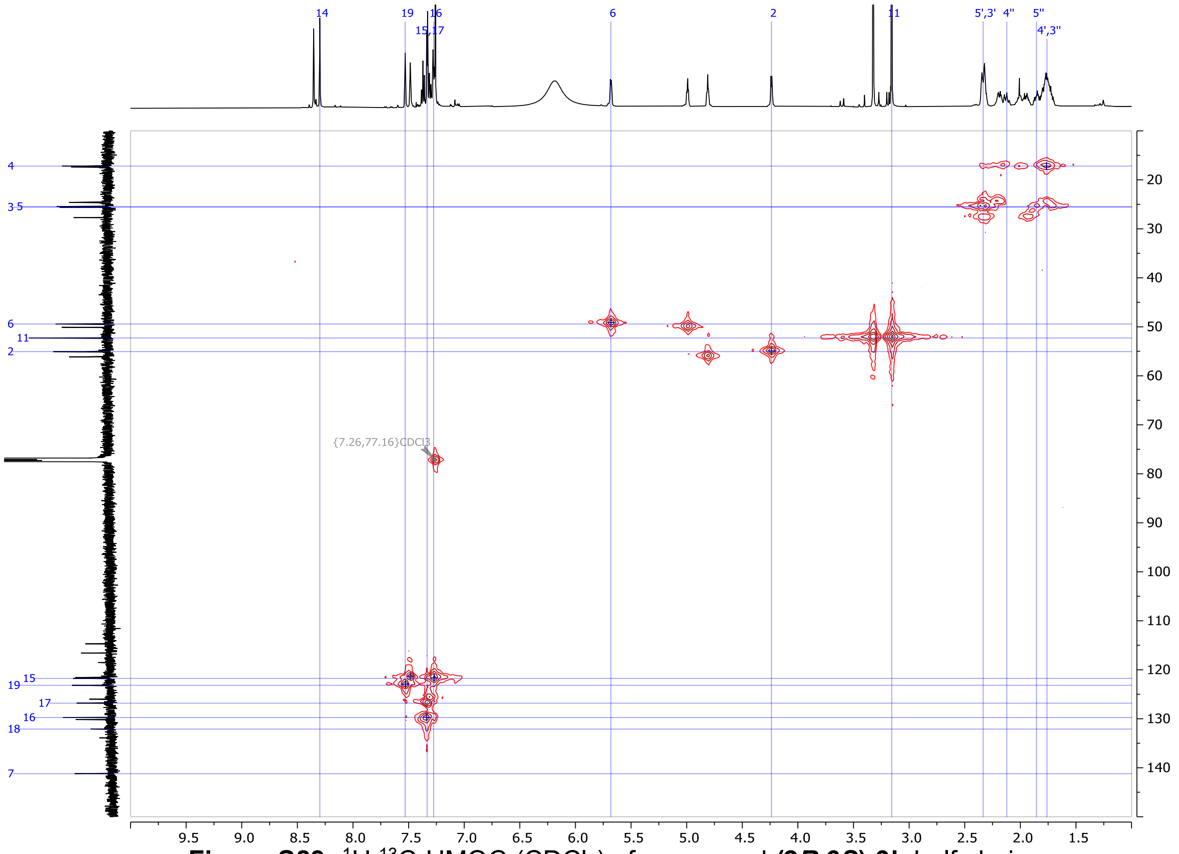

Figure S83: $^{1}H,^{13}C$-HMQC ($CDCl_3$) of compound **(2*R*,6*S*)-9h** half-chair.

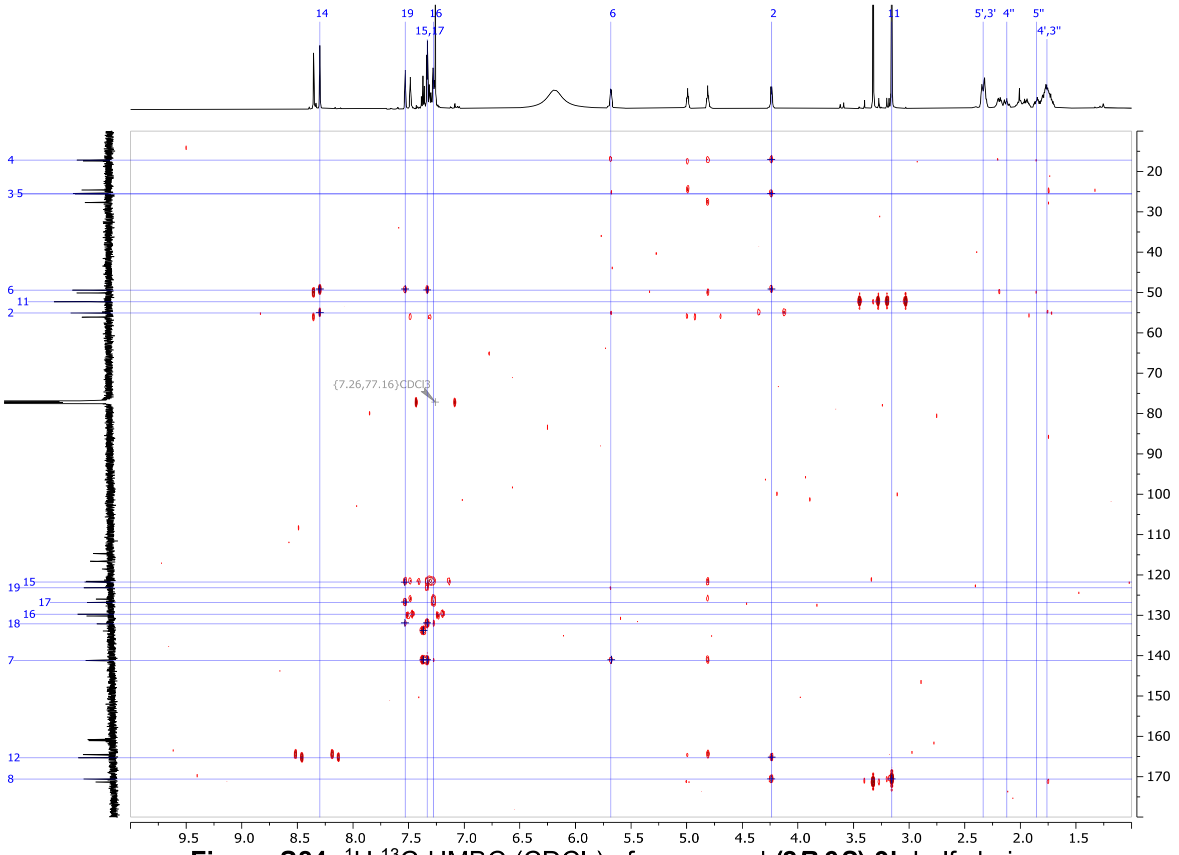

Figure S84: $^{1}H,^{13}C$-HMBC ($CDCl_3$) of compound **(2*R*,6*S*)-9h** half-chair.

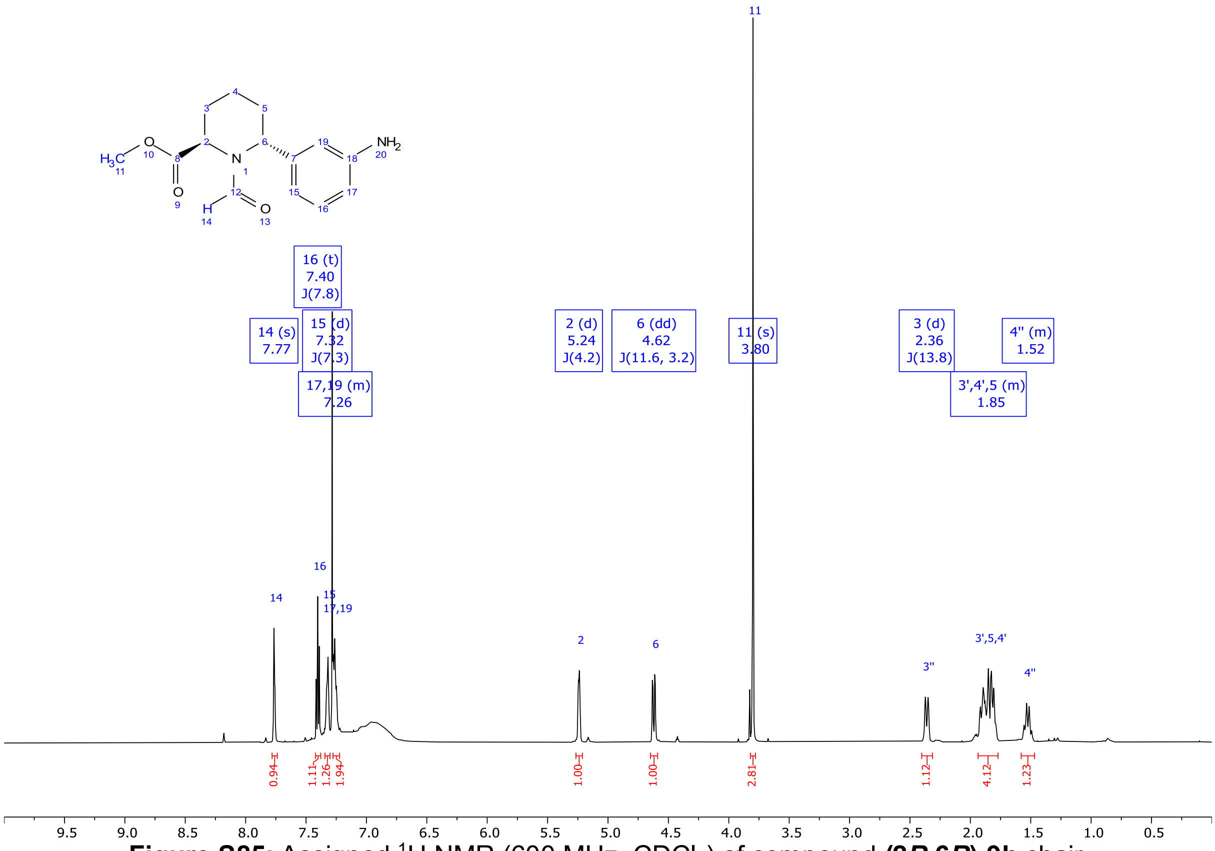

Figure S85: Assigned ^{1}H NMR (600 MHz, $CDCl_3$) of compound **(2*R*,6*R*)-9h** chair.

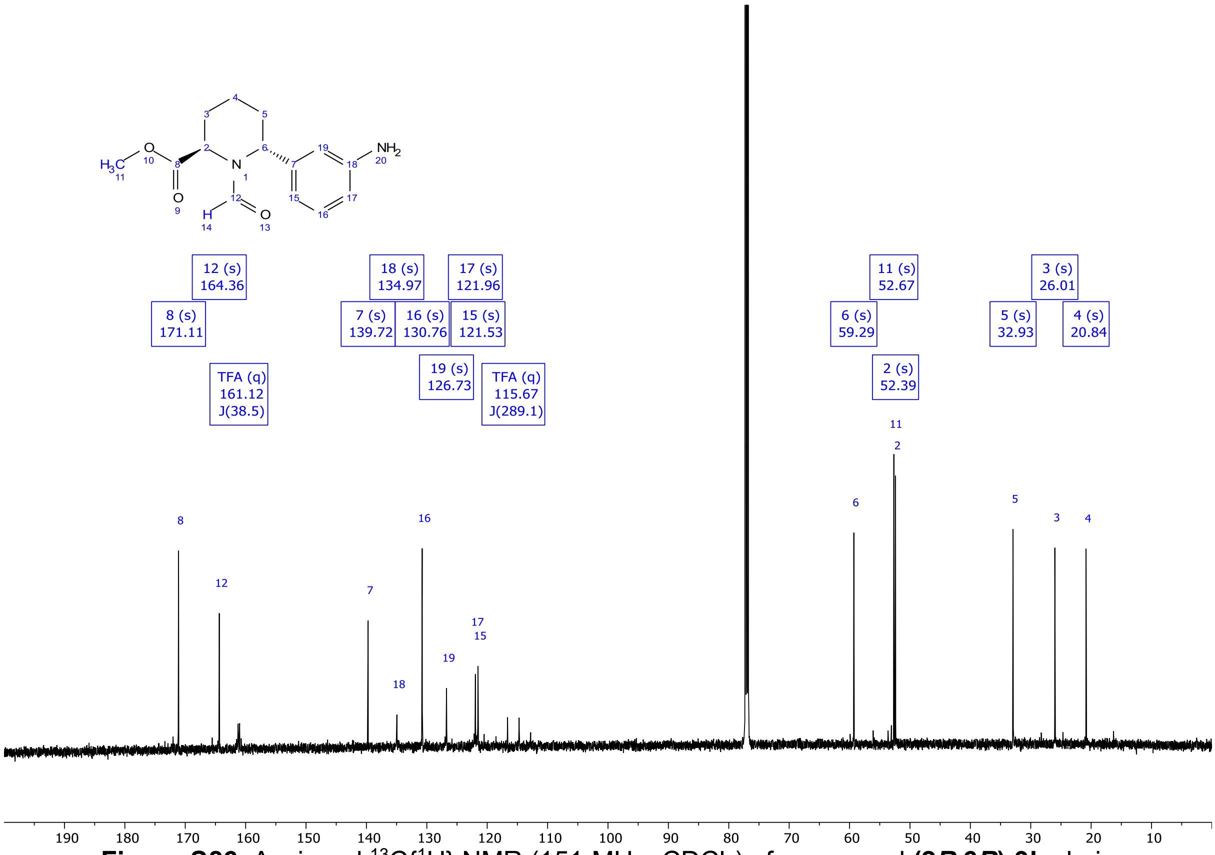

Figure S86: Assigned ^{13}C{^{1}H} NMR (151 MHz, $CDCl_3$) of compound **(2*R*,6*R*)-9h** chair.

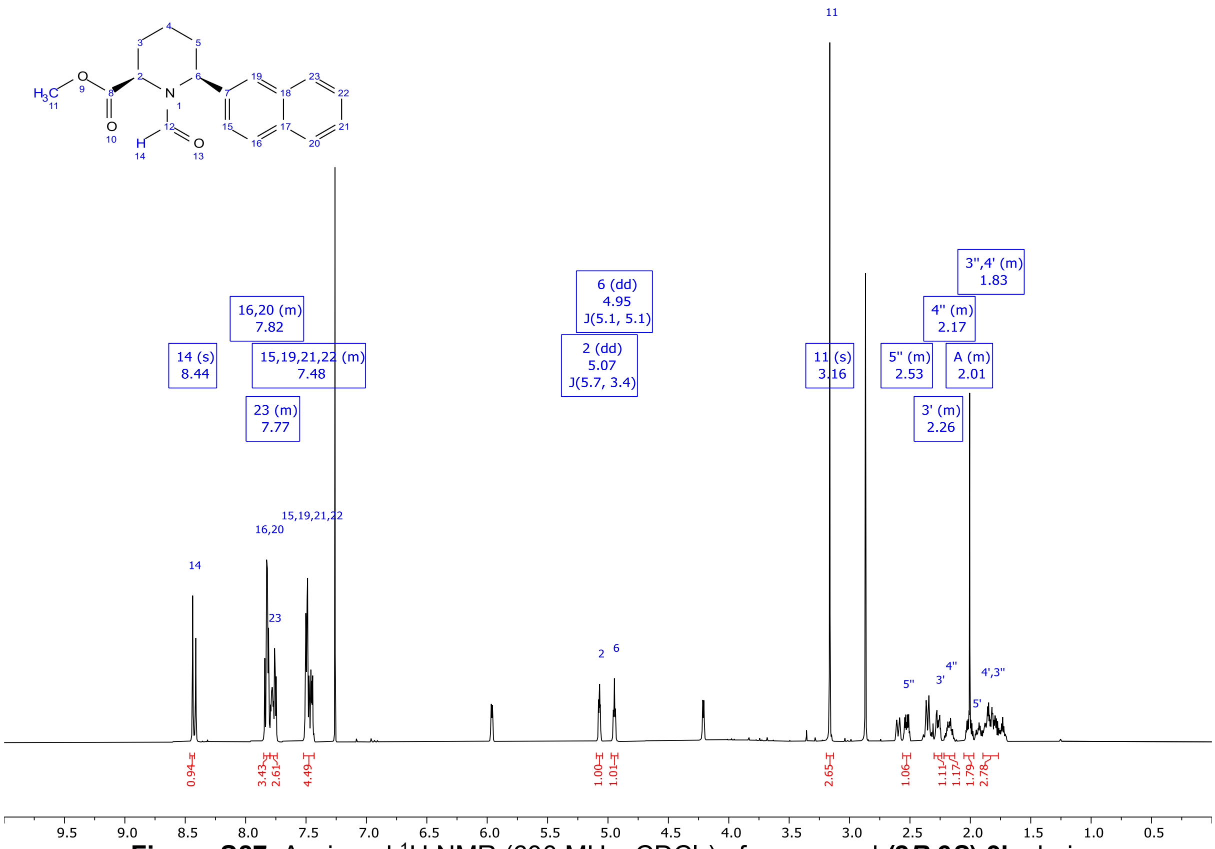

Figure S87: Assigned ^{1}H NMR (600 MHz, $CDCl_3$) of compound **(2*R*,6*S*)-9k** chair.

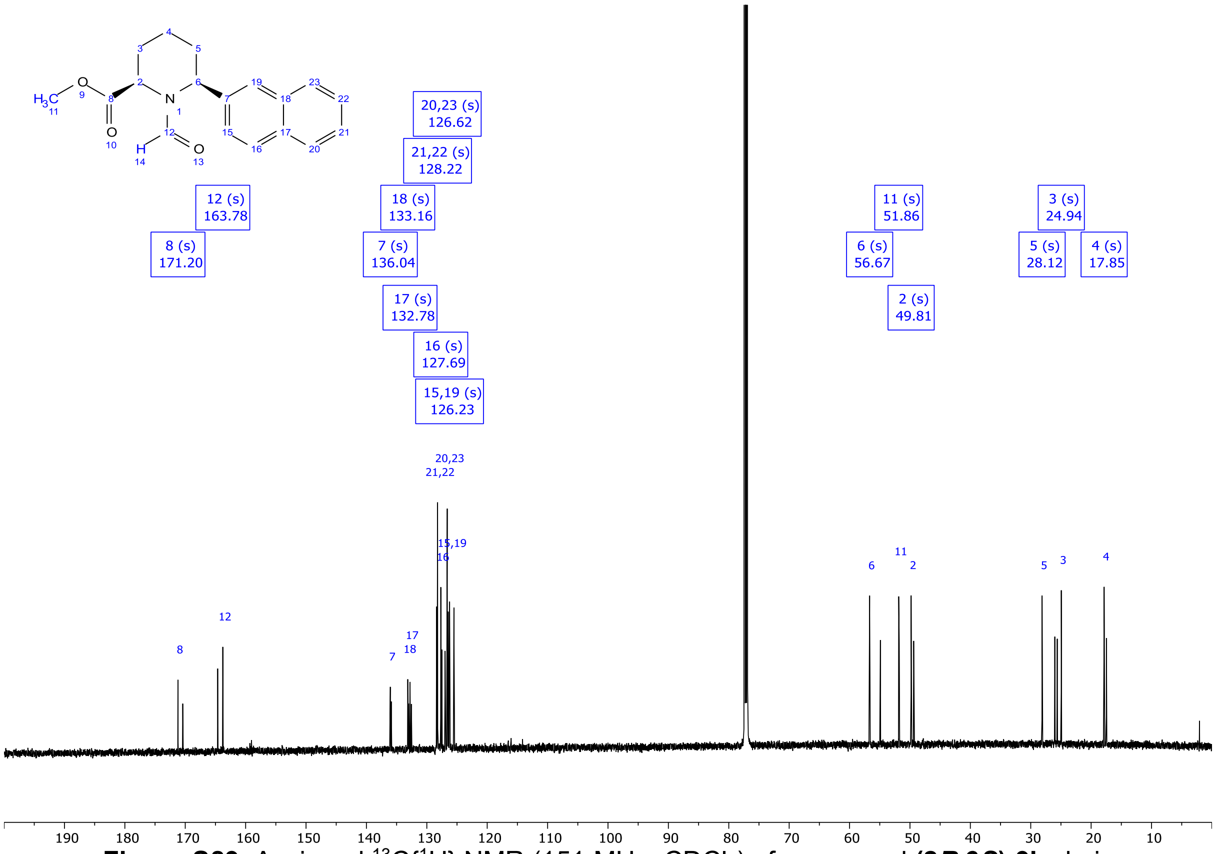

Figure S89: Assigned $^{13}C\{^{1}H\}$ NMR (151 MHz, $CDCl_3$) of compound **(2*R*,6*S*)-9k** chair.

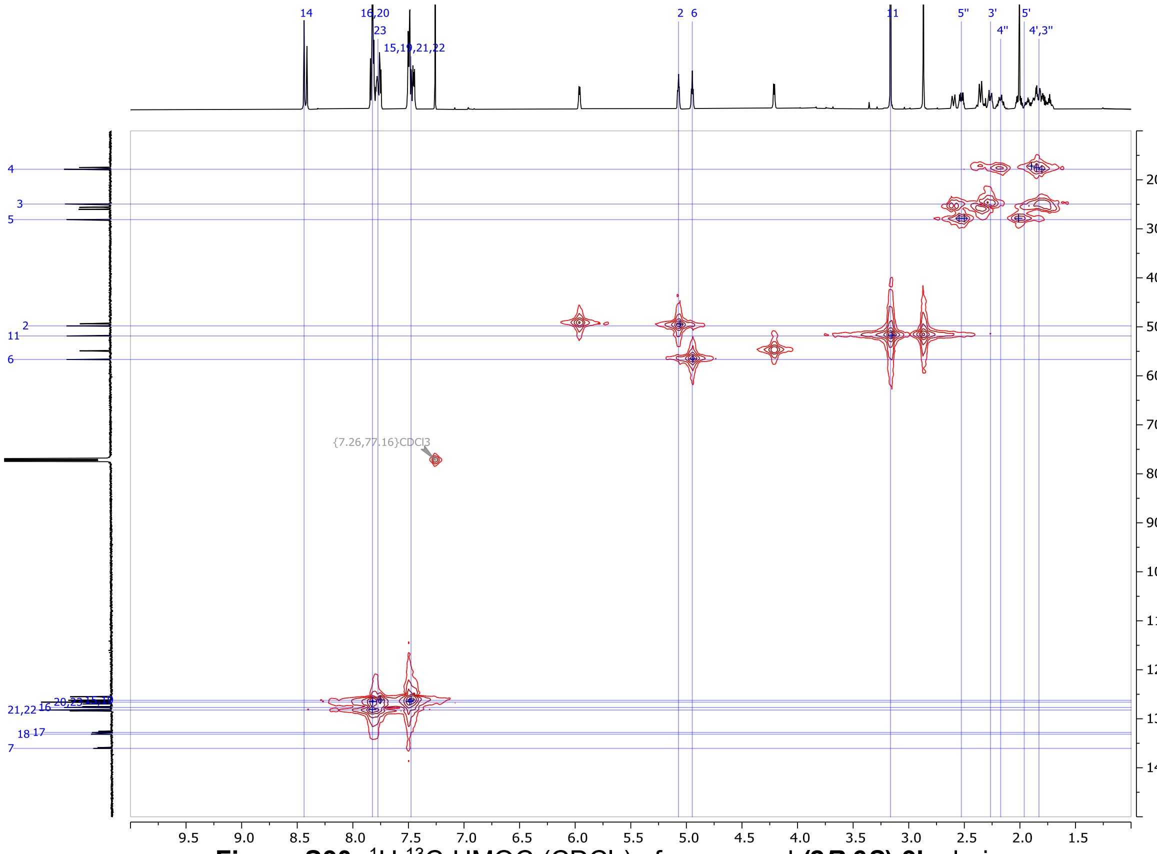

Figure S90: ^{1}H,^{13}C-HMQC (CDCl$_3$) of compound **(2*R*,6*S*)-9k** chair.

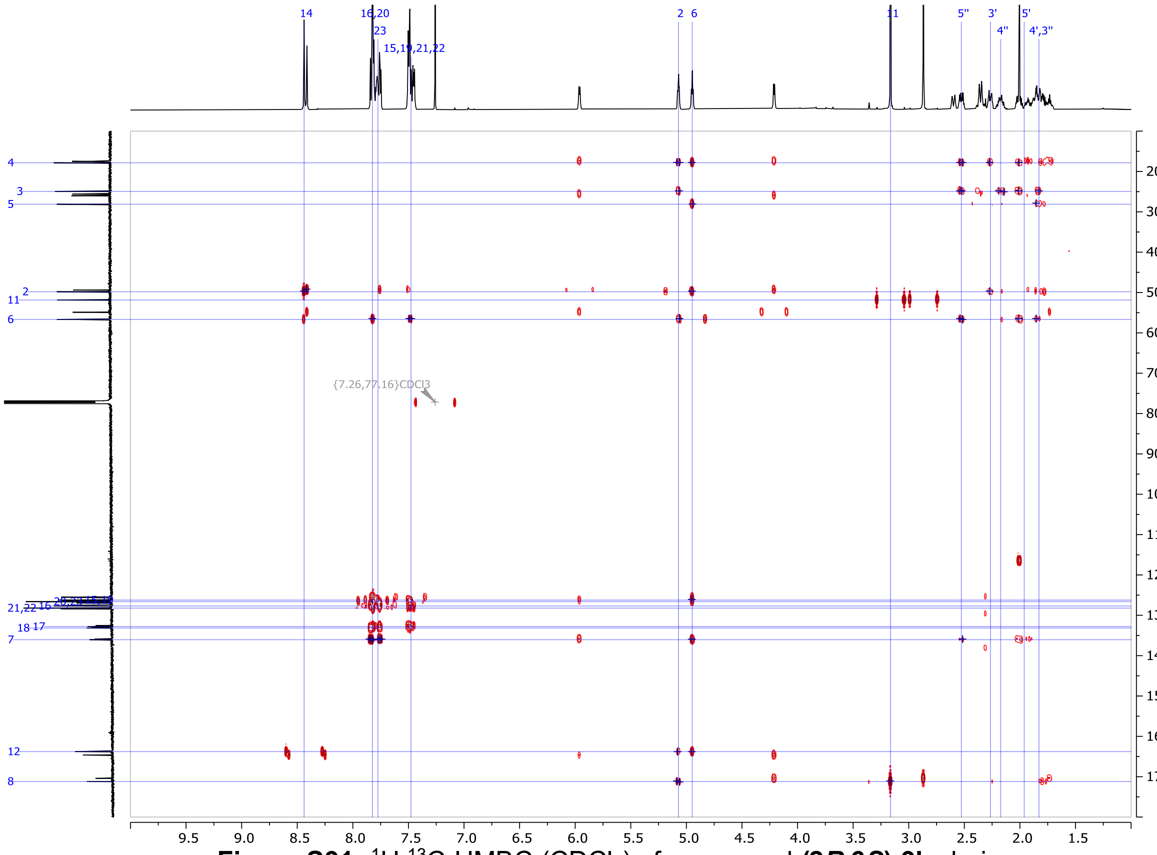

Figure S91: ^{1}H,^{13}C-HMBC (CDCl$_3$) of compound **(2*R*,6*S*)-9k** chair.

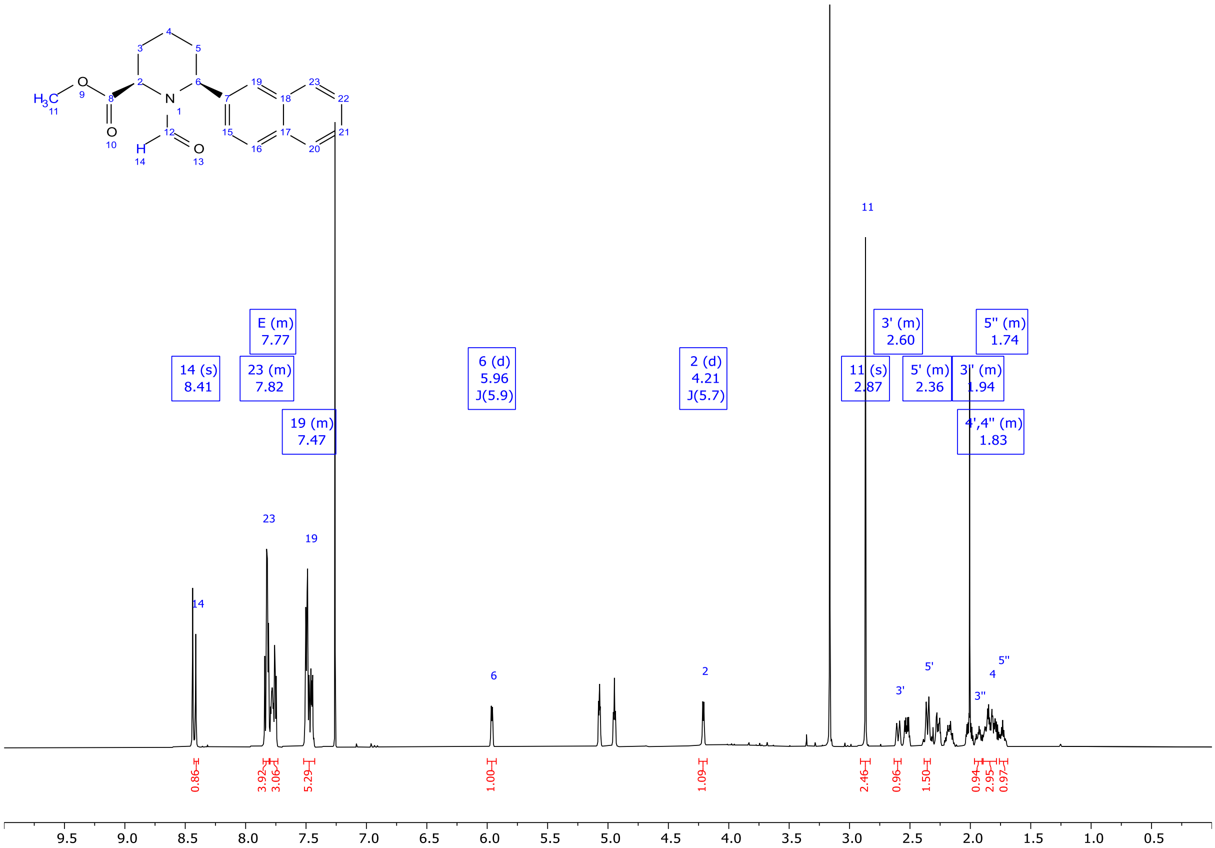

Figure S92: Assigned ^{1}H NMR (600 MHz, $CDCl_3$) of compound **(2*R*,6*S*)-9k** half-chair.

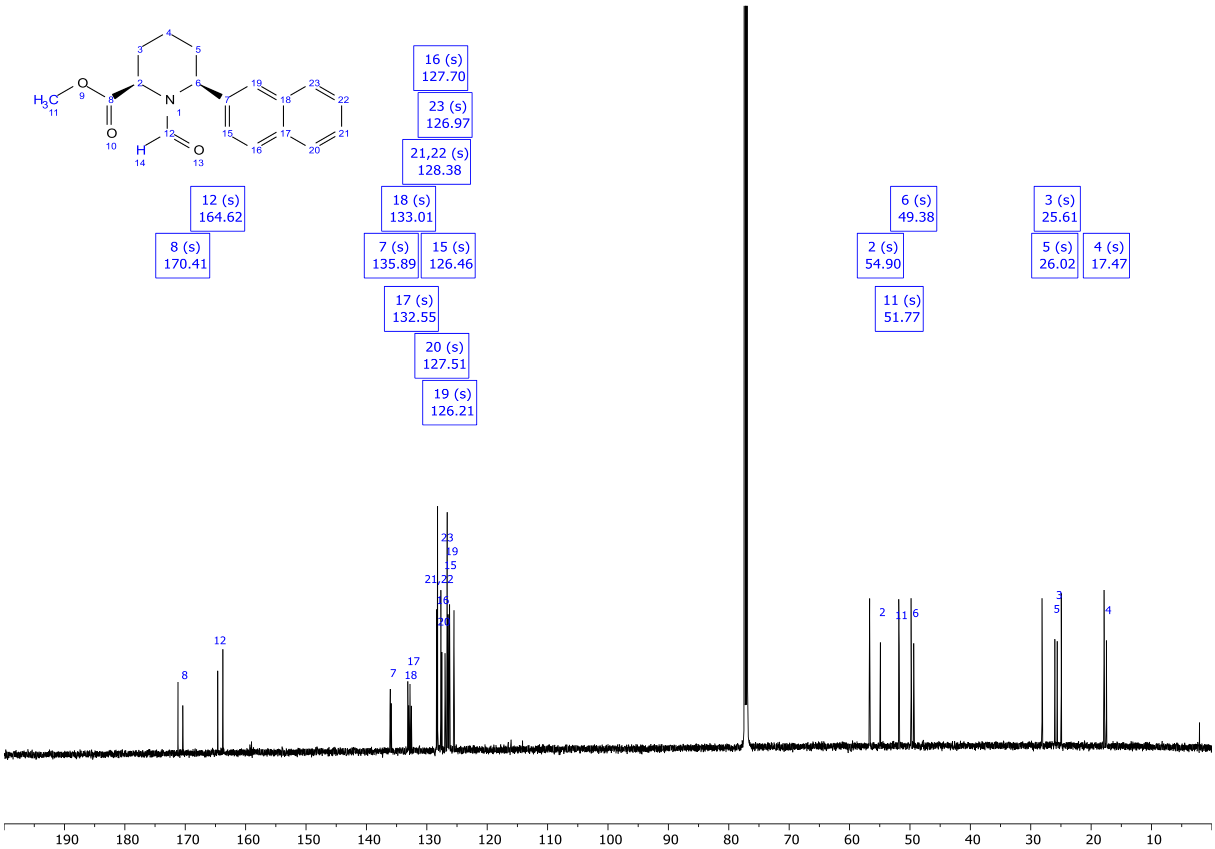

Figure S93: Assigned $^{13}C\{^{1}H\}$ NMR (151 MHz, $CDCl_3$) of compound **(2*R*,6*S*)-9k** half-chair.

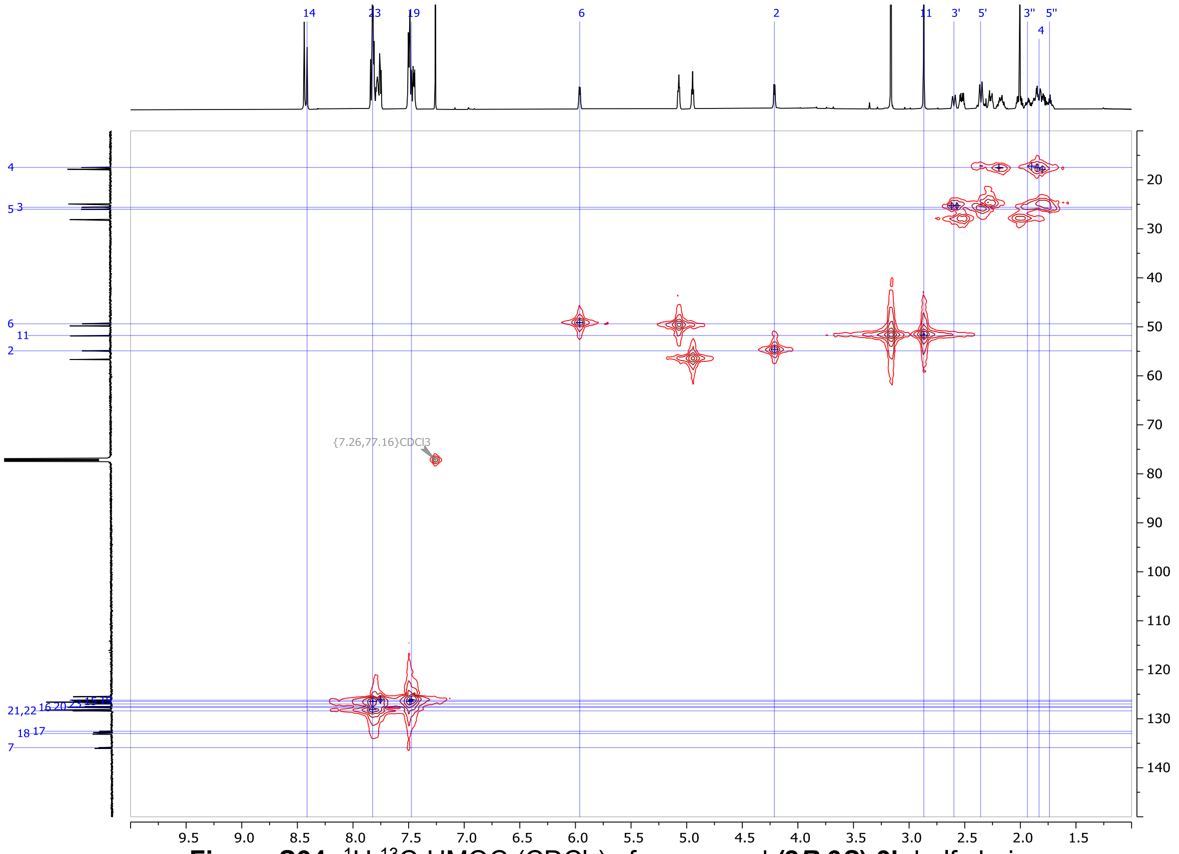

Figure S94: $^{1}H,^{13}C$-HMQC ($CDCl_3$) of compound **(2*R*,6*S*)-9k** half-chair.

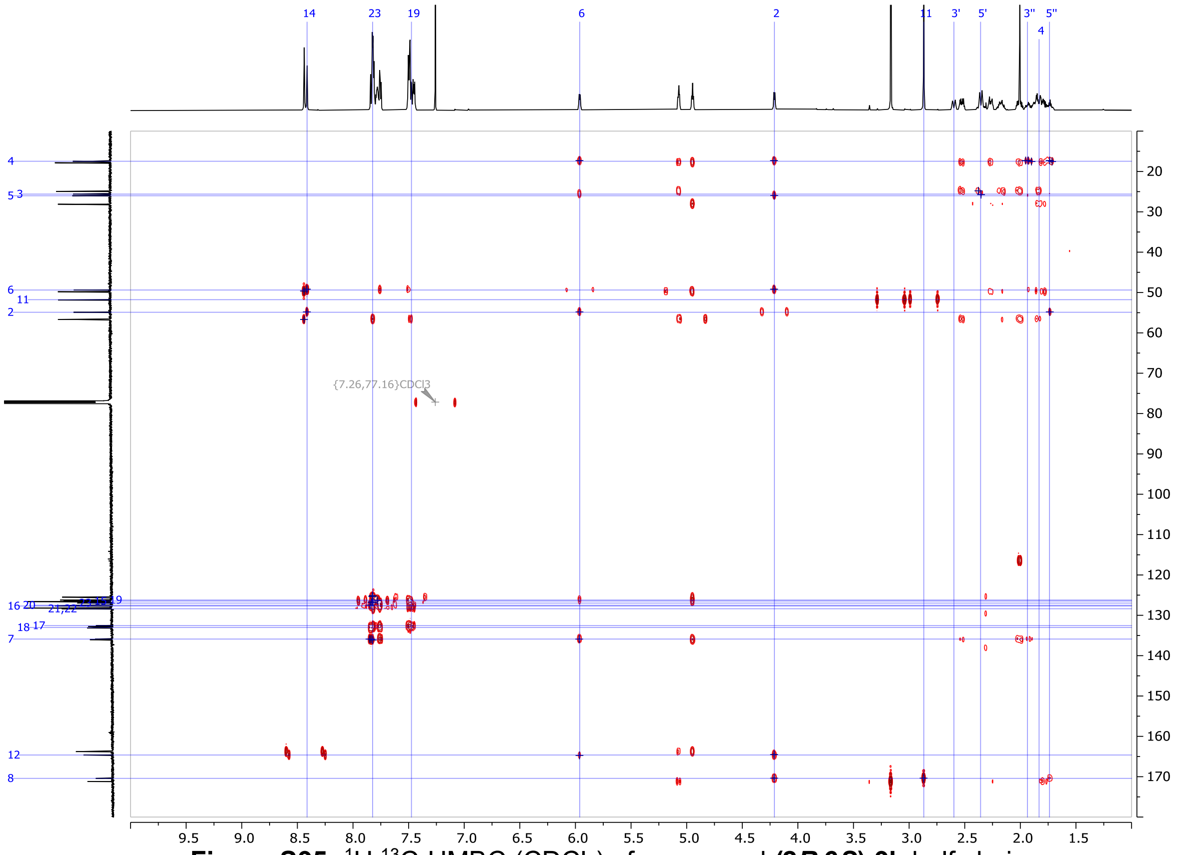

Figure S95: $^{1}H,^{13}C$-HMBC ($CDCl_3$) of compound **(2*R*,6*S*)-9k** half-chair.

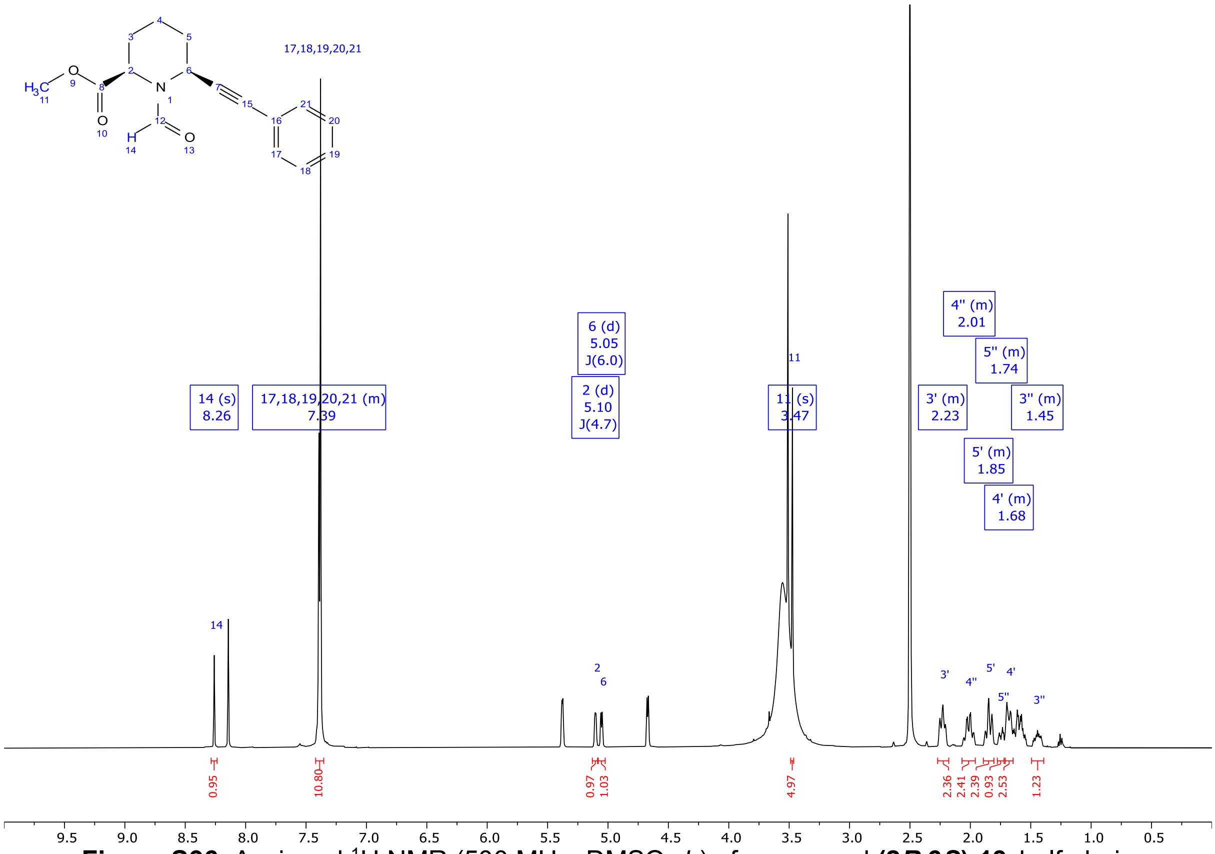

Figure S96: Assigned ^{1}H NMR (500 MHz, DMSO-d_6) of compound **(2*R*,6*S*)-13l** half-chair.

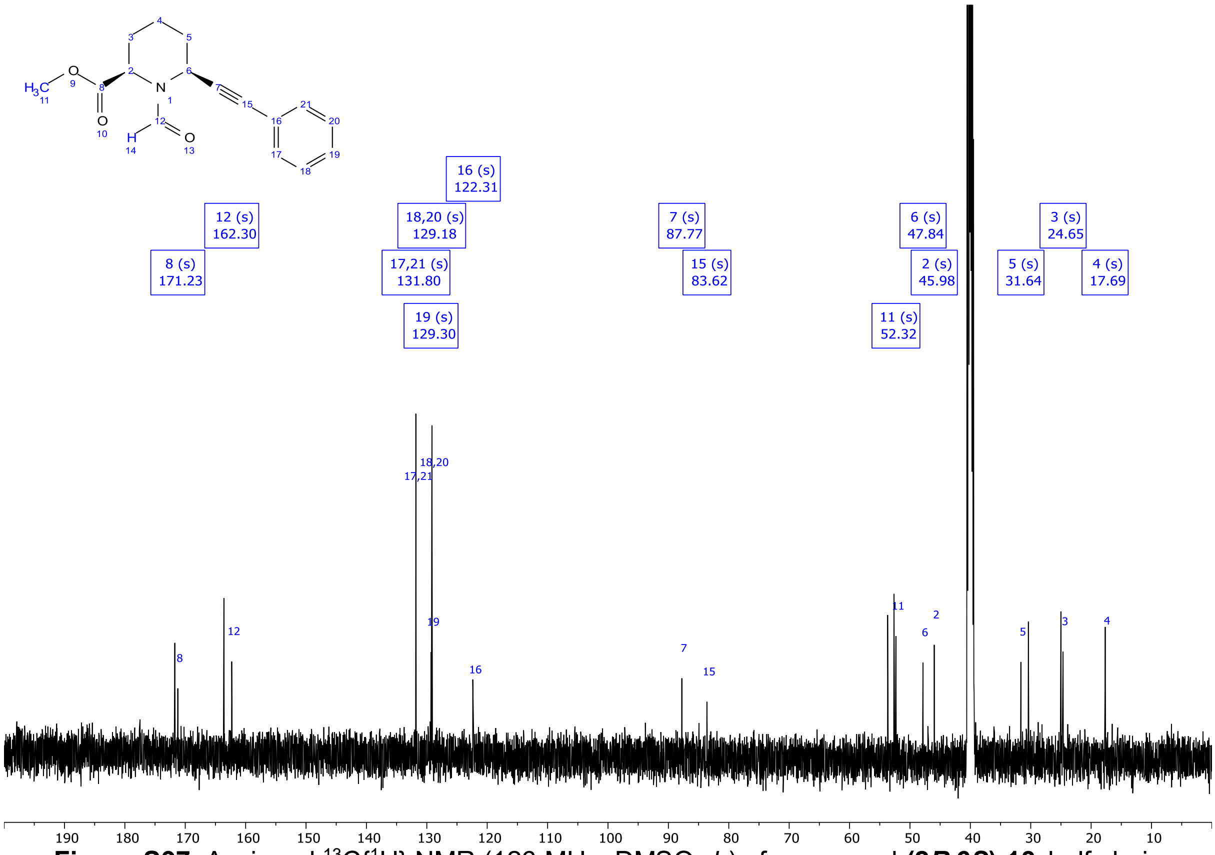

Figure S97: Assigned ^{13}C{^{1}H} NMR (126 MHz, DMSO-d_6) of compound **(2*R*,6*S*)-13l** half-chair.

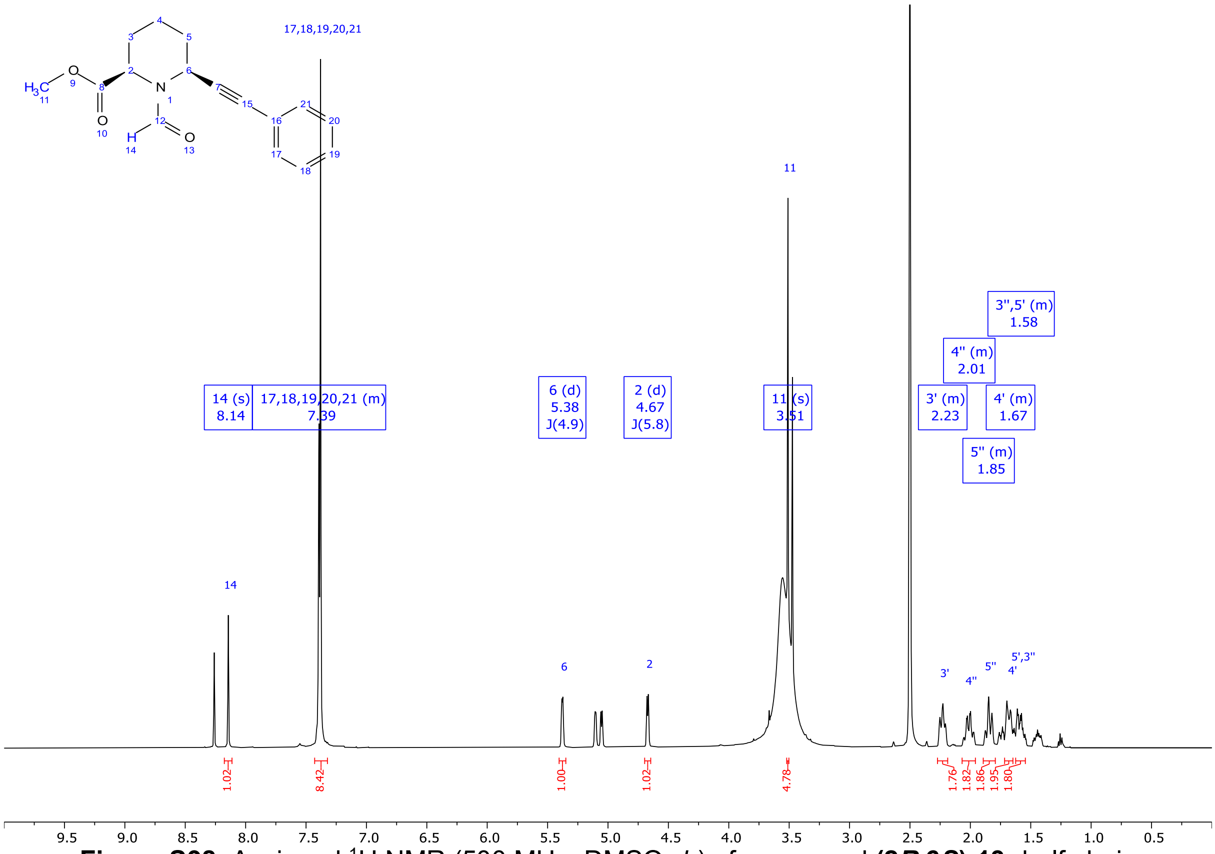

Figure S98: Assigned ^{1}H NMR (500 MHz, DMSO-d_6) of compound **(2*R*,6*S*)-13$_{II}$** half-chair.

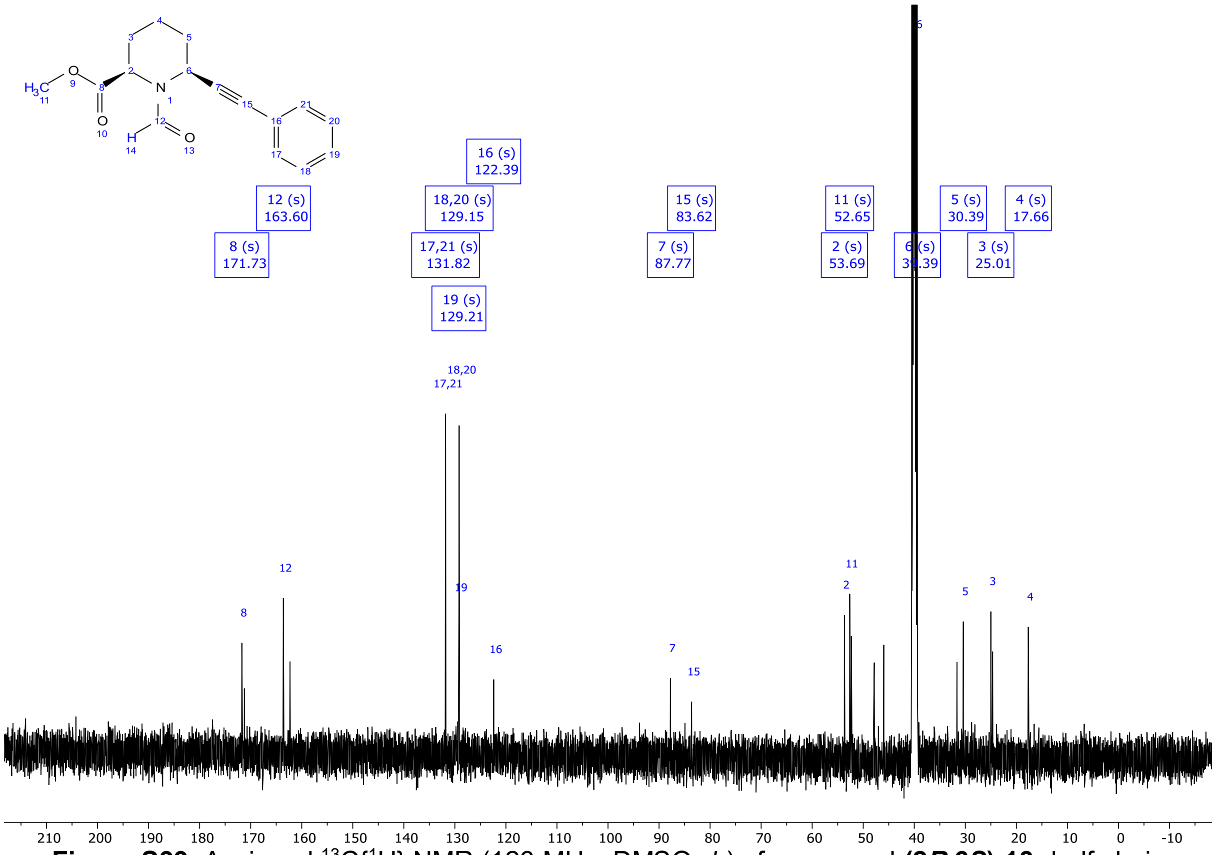

Figure S99: Assigned ^{13}C{^{1}H} NMR (126 MHz, DMSO-d_6) of compound **(2*R*,6*S*)-13$_{II}$** half-chair.

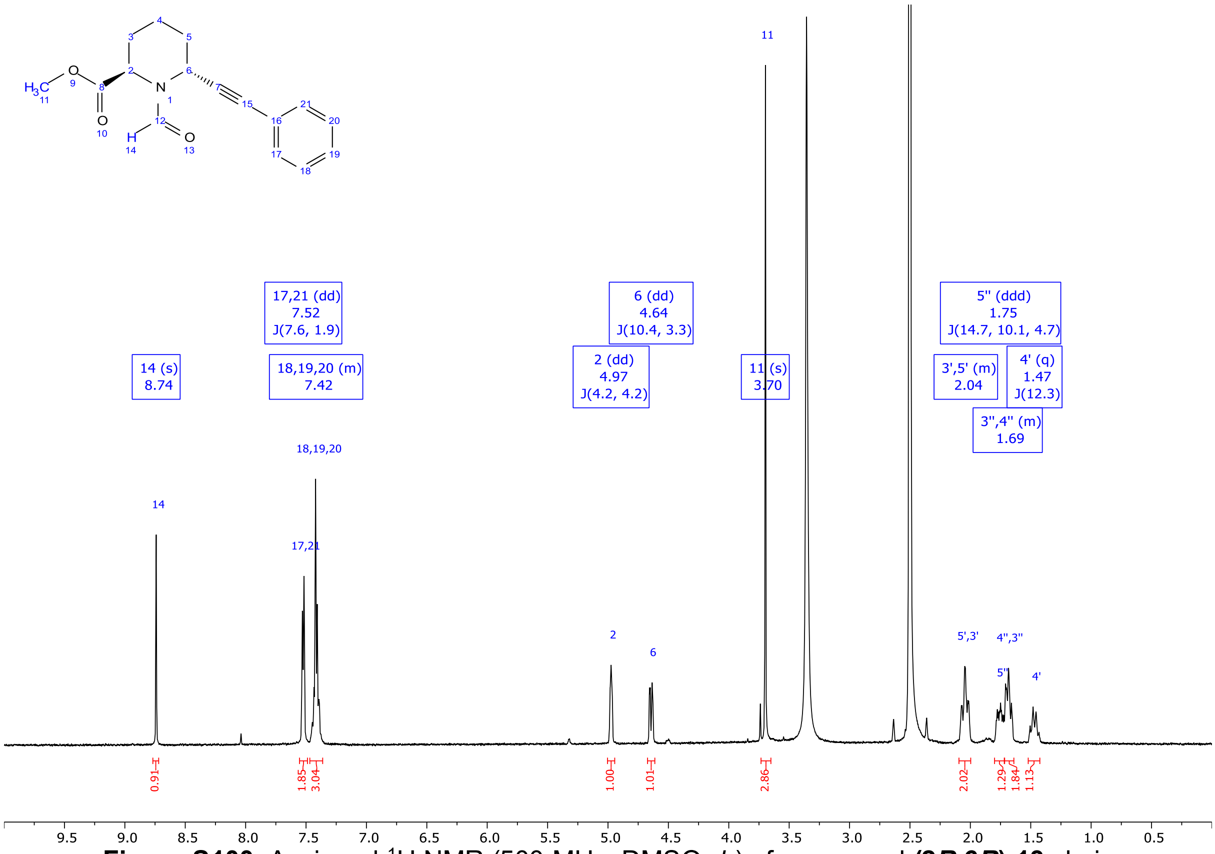

Figure S100: Assigned ^{1}H NMR (500 MHz, DMSO-d_6) of compound **(2*R*,6*R*)-13** chair.

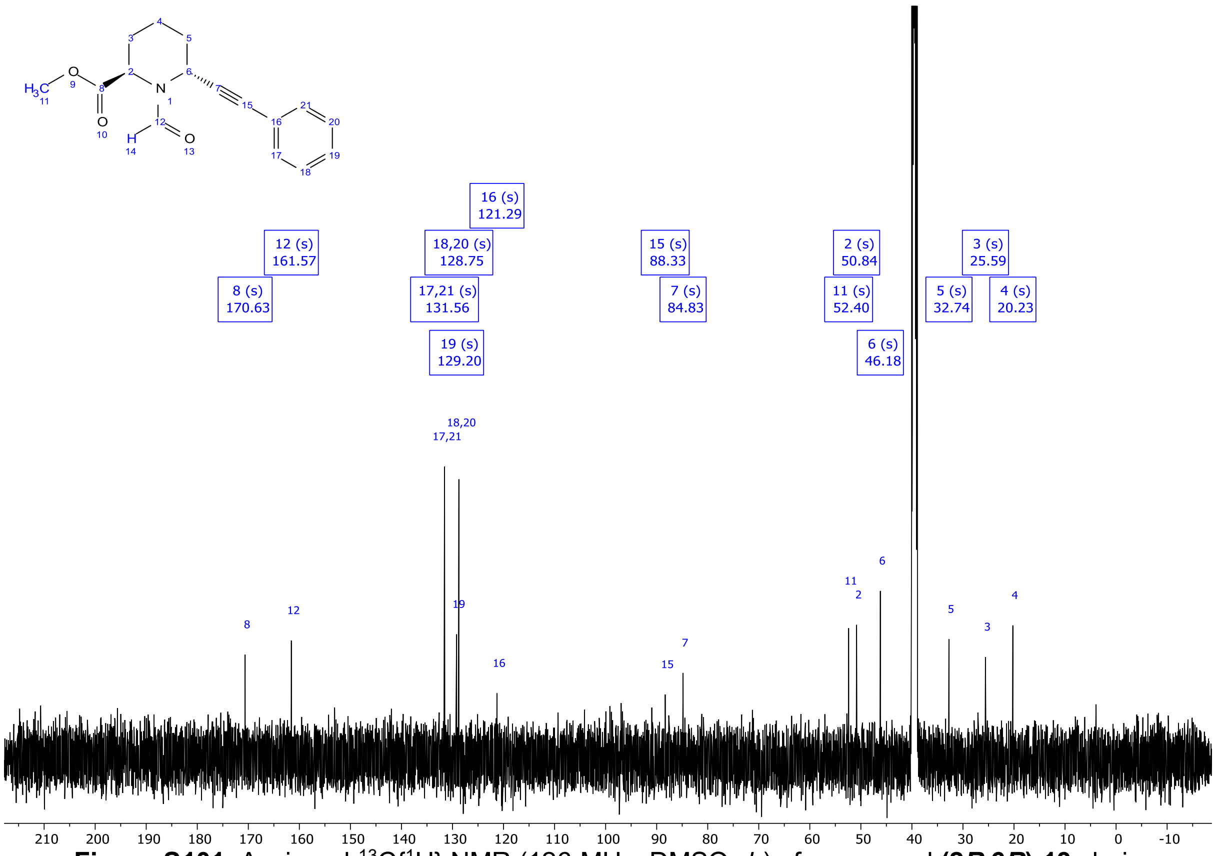

Figure S101: Assigned ^{13}C{^{1}H} NMR (126 MHz, DMSO-d_6) of compound **(2*R*,6*R*)-13** chair.

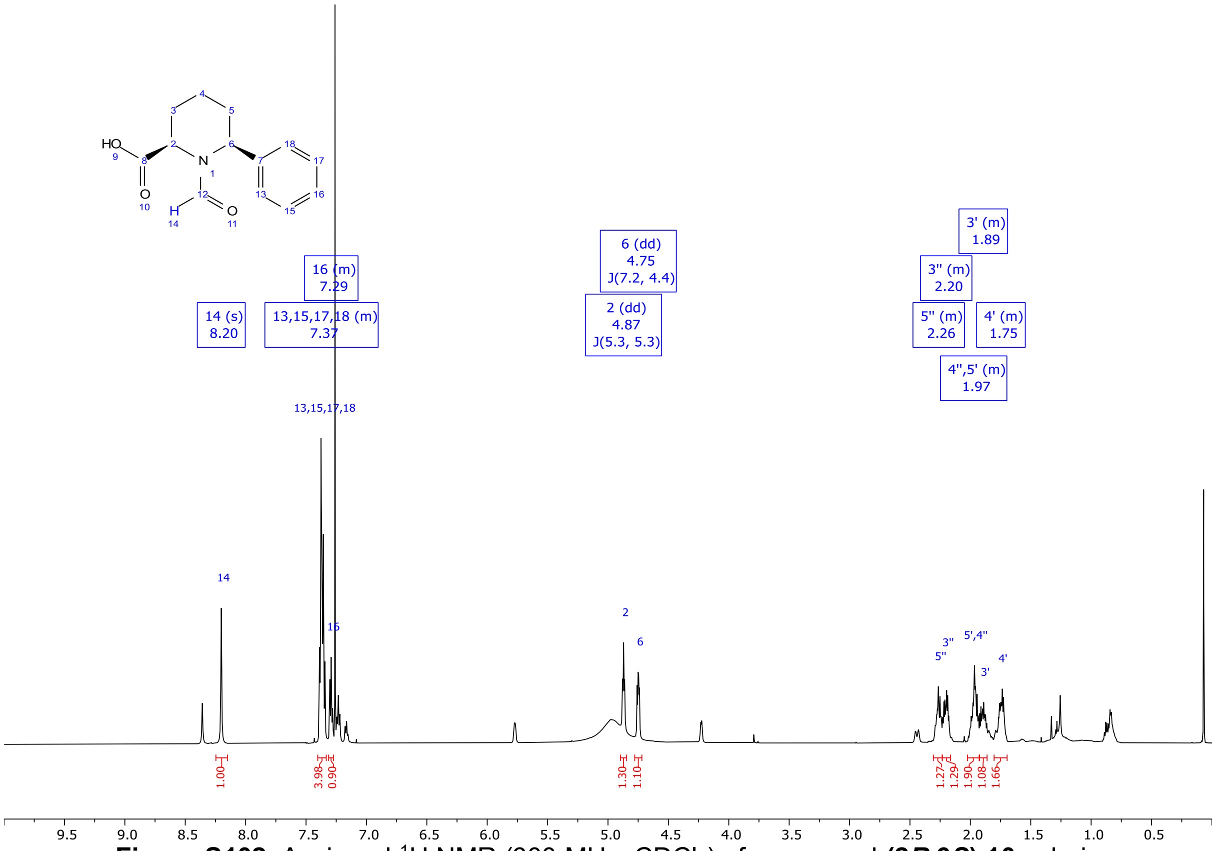

Figure S102: Assigned ^{1}H NMR (600 MHz, $CDCl_3$) of compound **(2*R*,6*S*)-10a** chair.

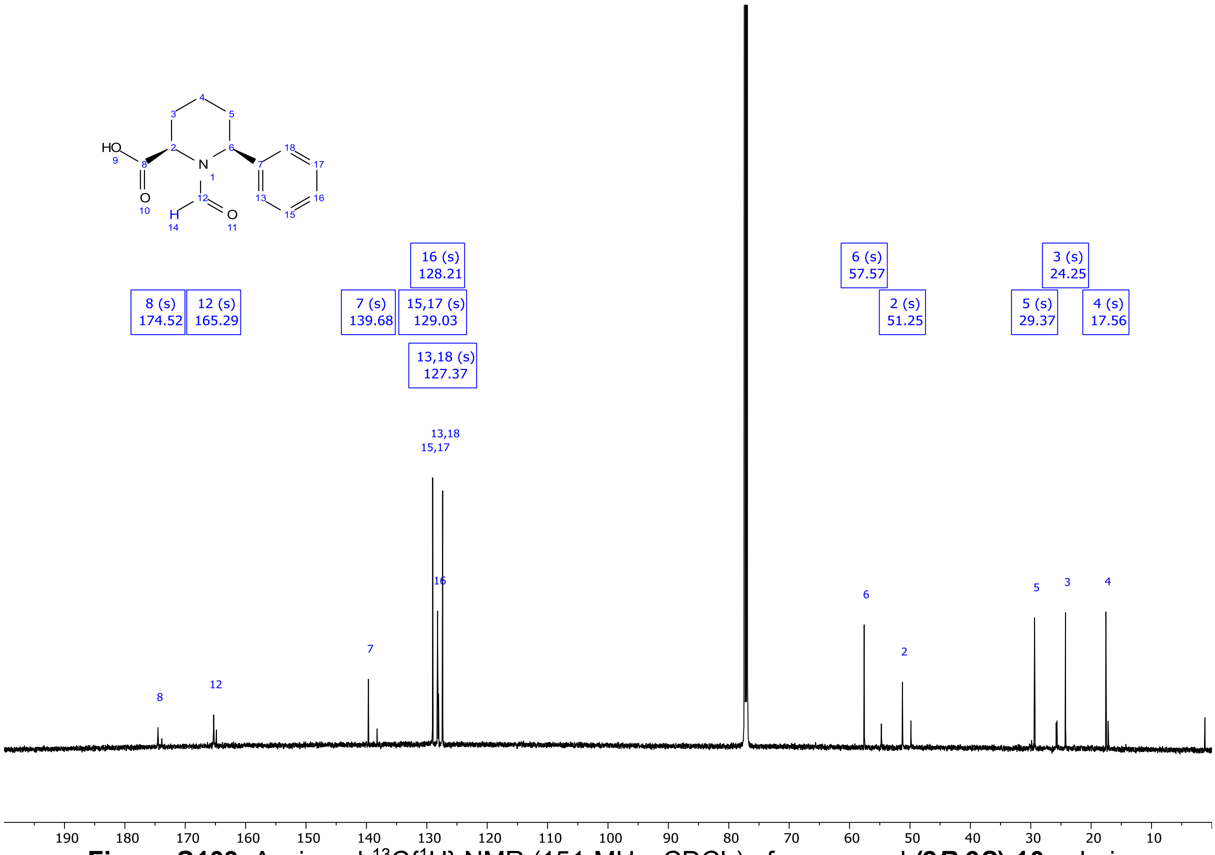

Figure S103: Assigned $^{13}C\{^{1}H\}$ NMR (151 MHz, $CDCl_3$) of compound **(2*R*,6*S*)-10a** chair.

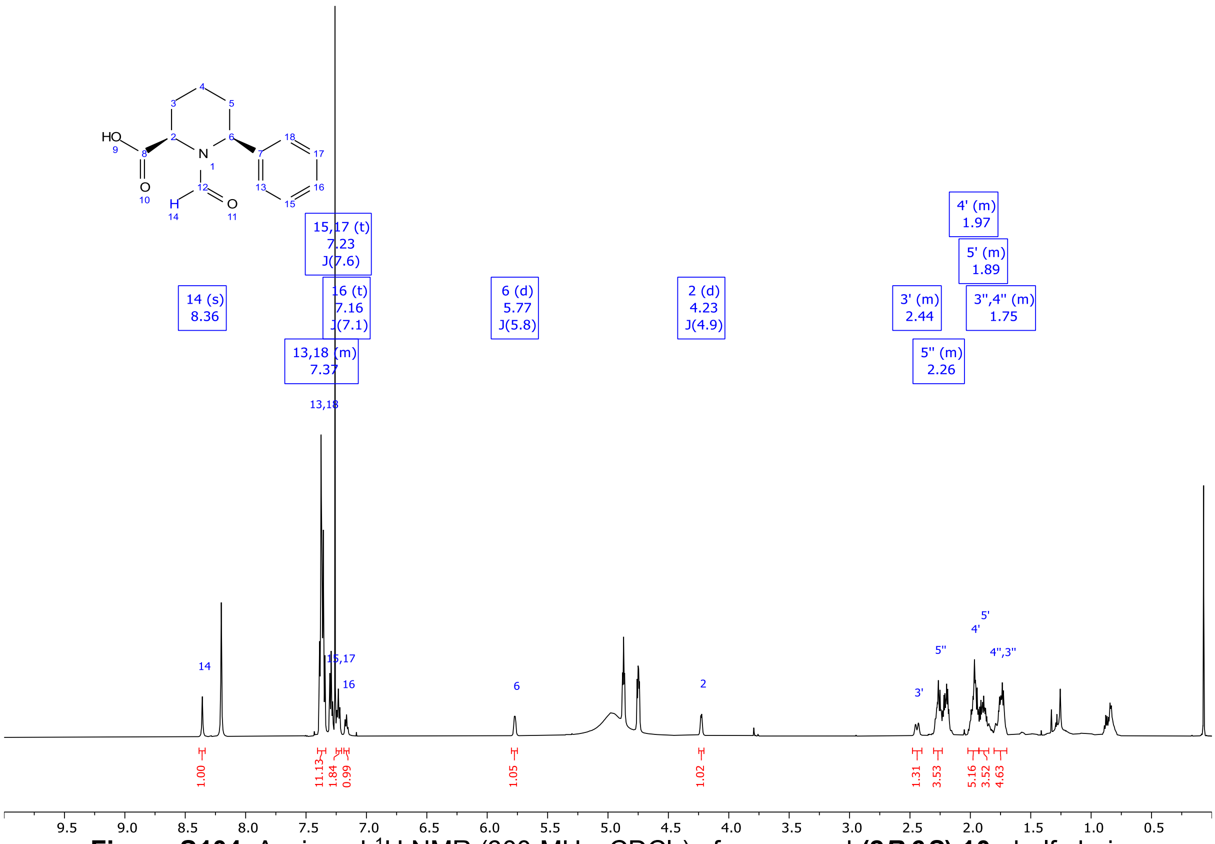

Figure S104: Assigned ^{1}H NMR (600 MHz, $CDCl_3$) of compound **(2*R*,6*S*)-10a** half-chair.

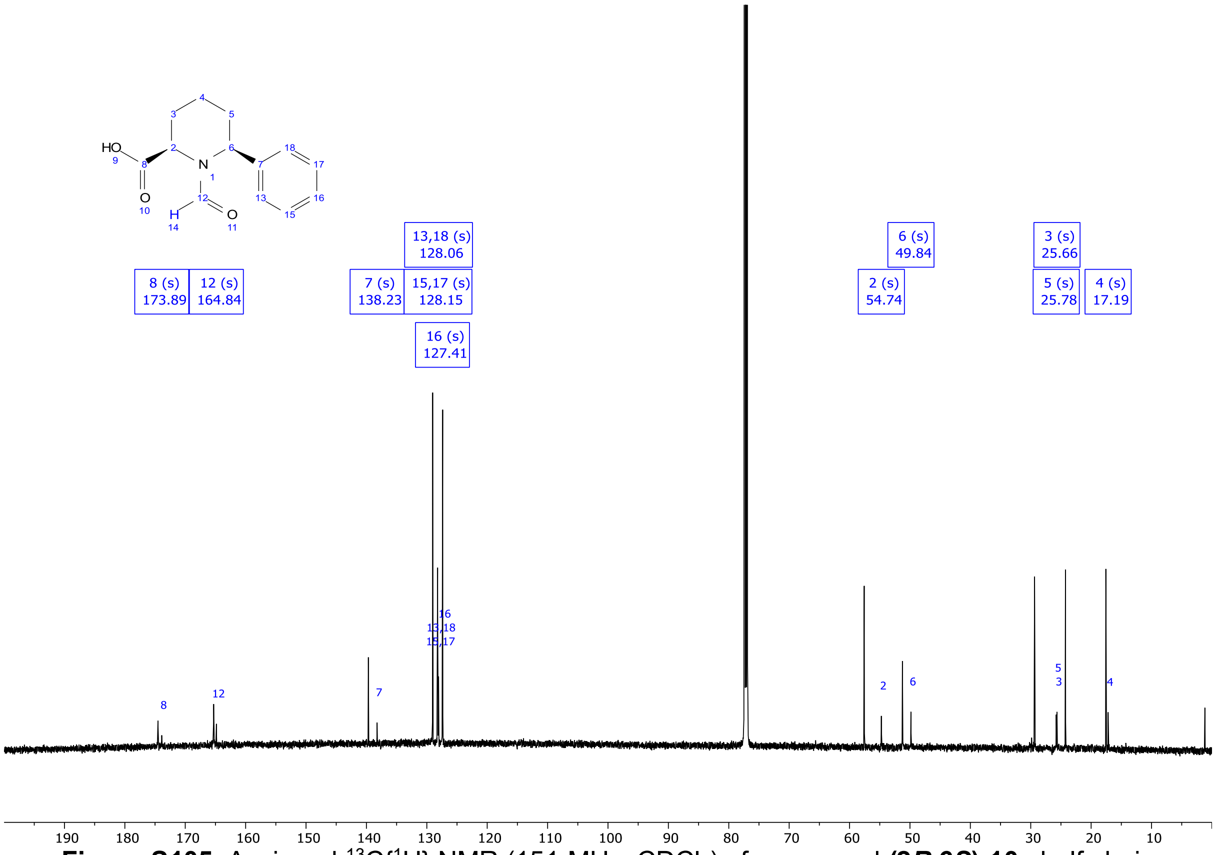

Figure S105: Assigned $^{13}C\{^{1}H\}$ NMR (151 MHz, $CDCl_3$) of compound **(2*R*,6*S*)-10a** half-chair.

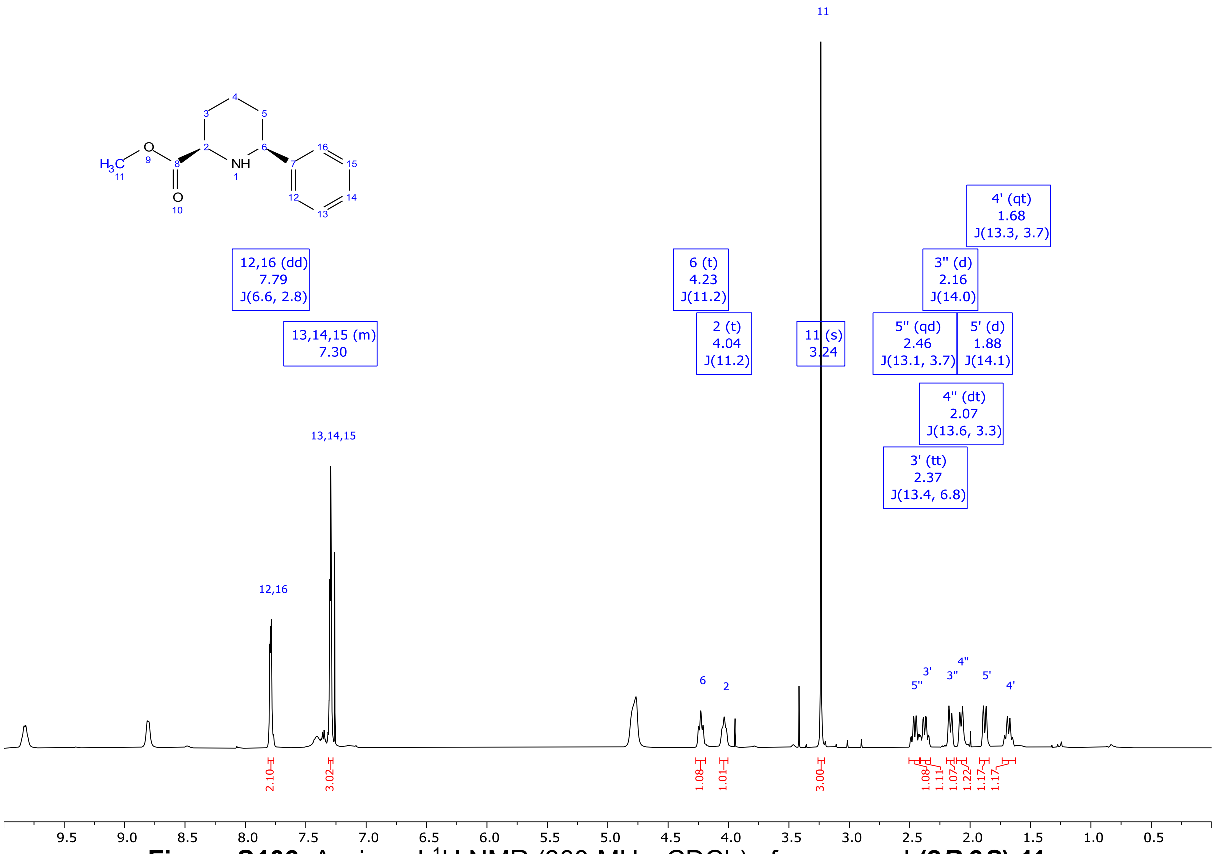

Figure S106: Assigned ^{1}H NMR (600 MHz, $CDCl_3$) of compound **(2*R*,6*S*)-11a**.

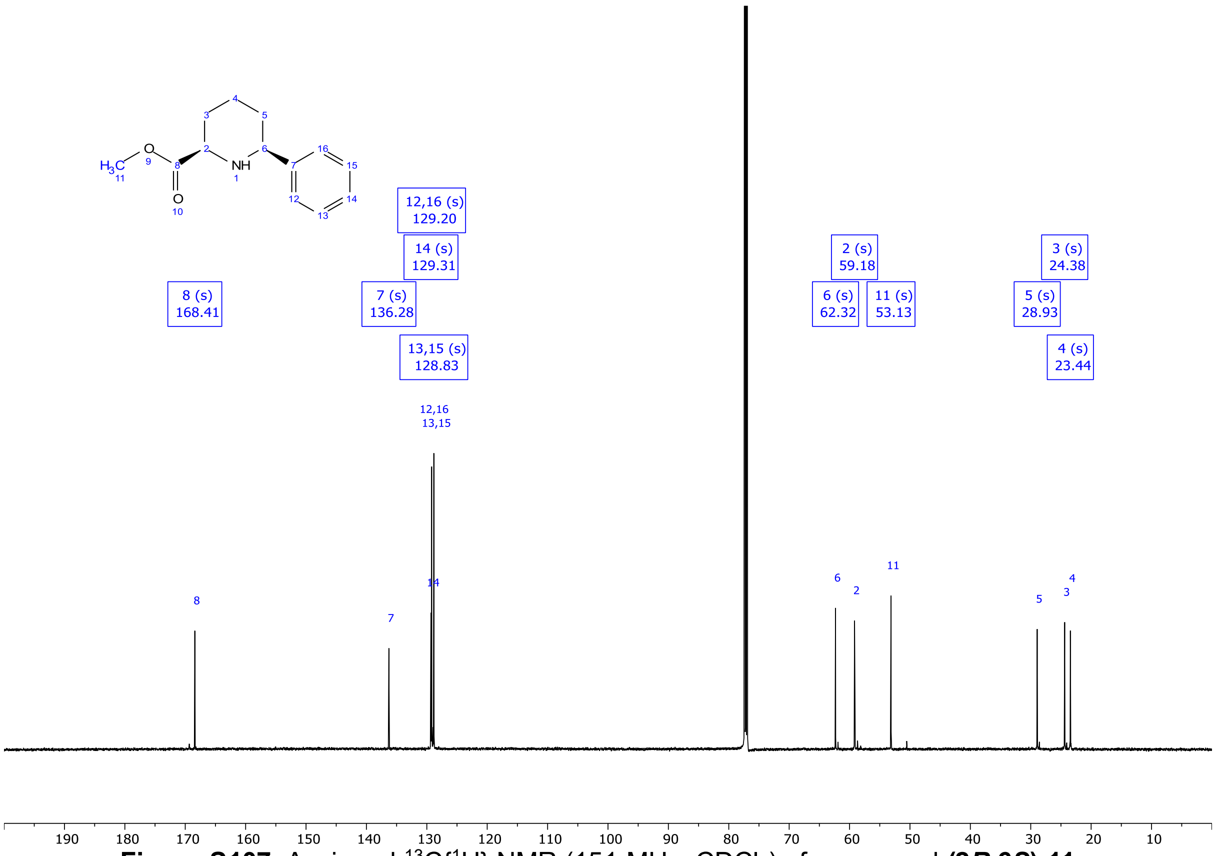

Figure S107: Assigned $^{13}C\{^{1}H\}$ NMR (151 MHz, $CDCl_3$) of compound **(2*R*,6*S*)-11a**.

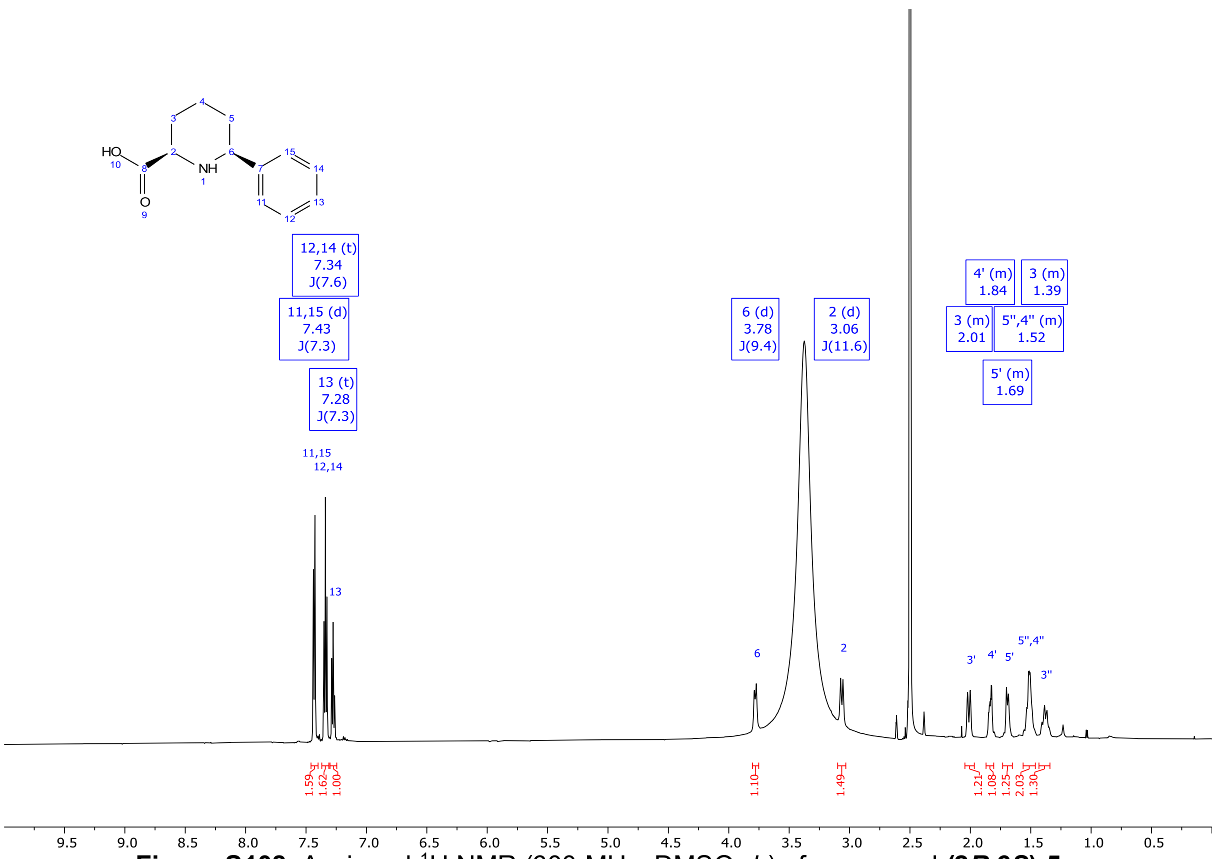

Figure S108: Assigned ^{1}H NMR (600 MHz, DMSO-d_6) of compound **(2*R*,6*S*)-5a**.

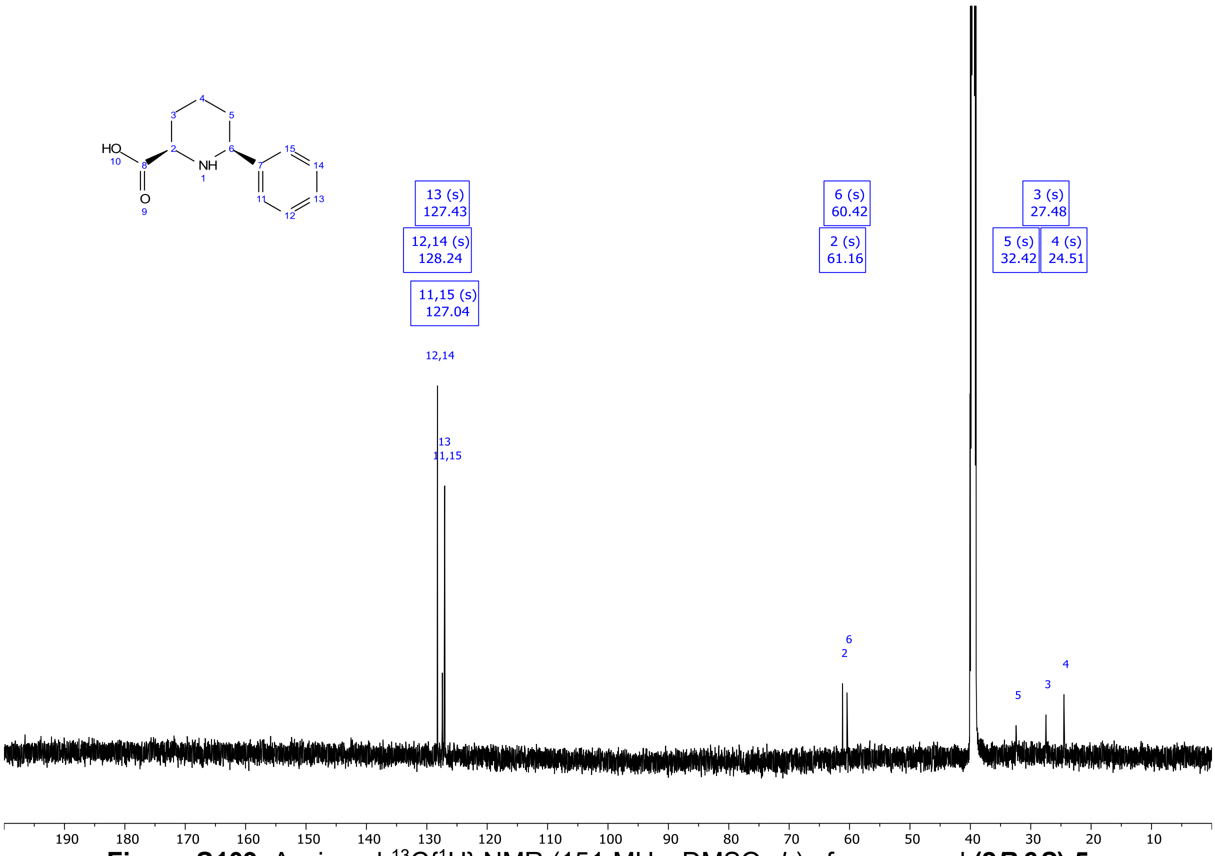

Figure S109: Assigned $^{13}C\{^{1}H\}$ NMR (151 MHz, DMSO-d_6) of compound **(2*R*,6*S*)-5a**.

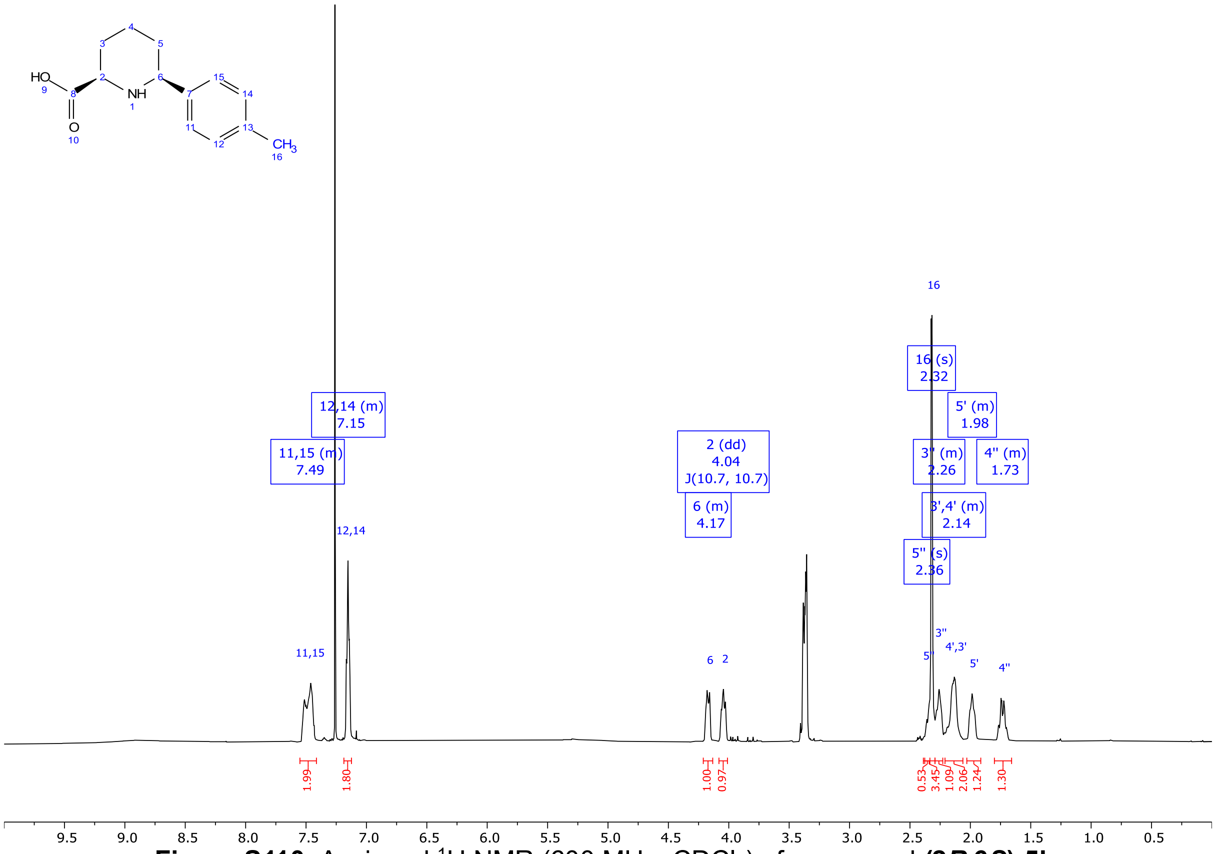

Figure S110: Assigned ^{1}H NMR (600 MHz, $CDCl_3$) of compound **(2*R*,6*S*)-5b**.

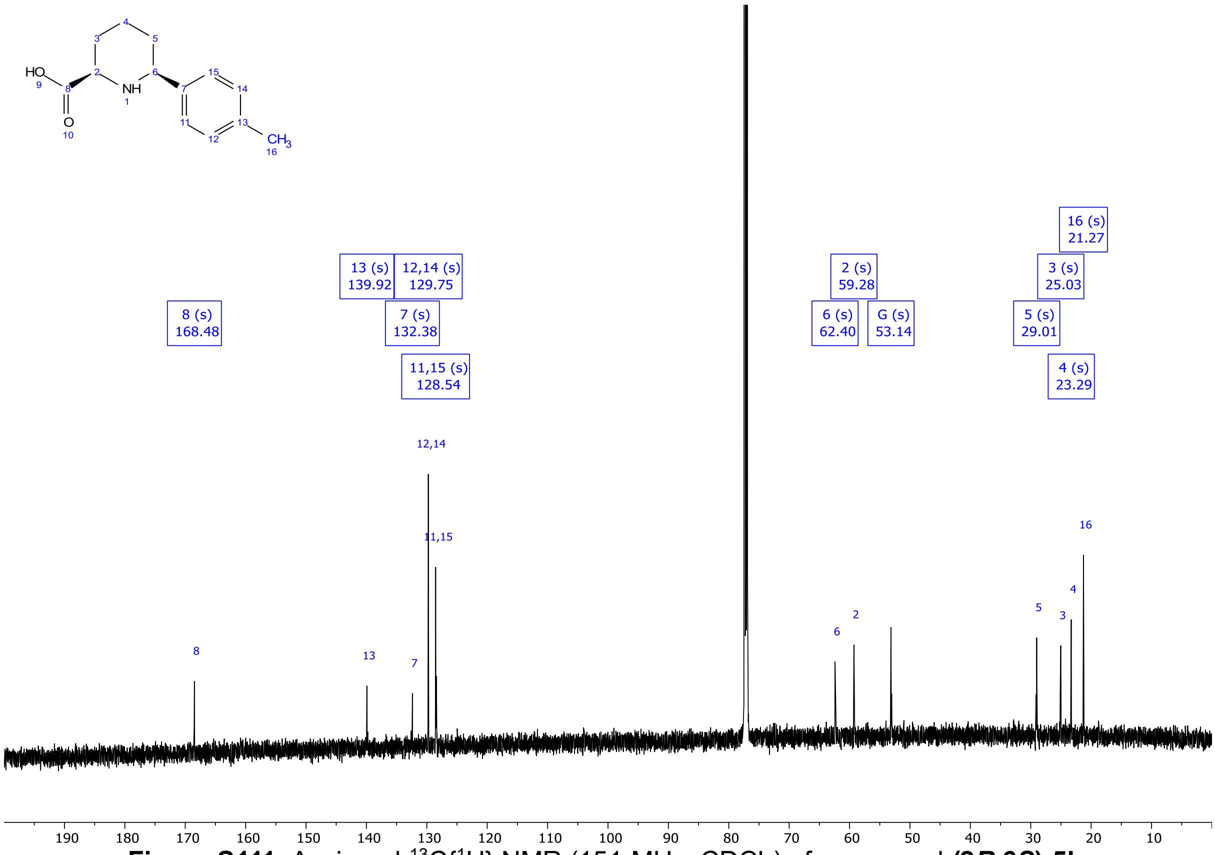

Figure S111: Assigned ^{13}C{^{1}H} NMR (151 MHz, $CDCl_3$) of compound **(2*R*,6*S*)-5b**.

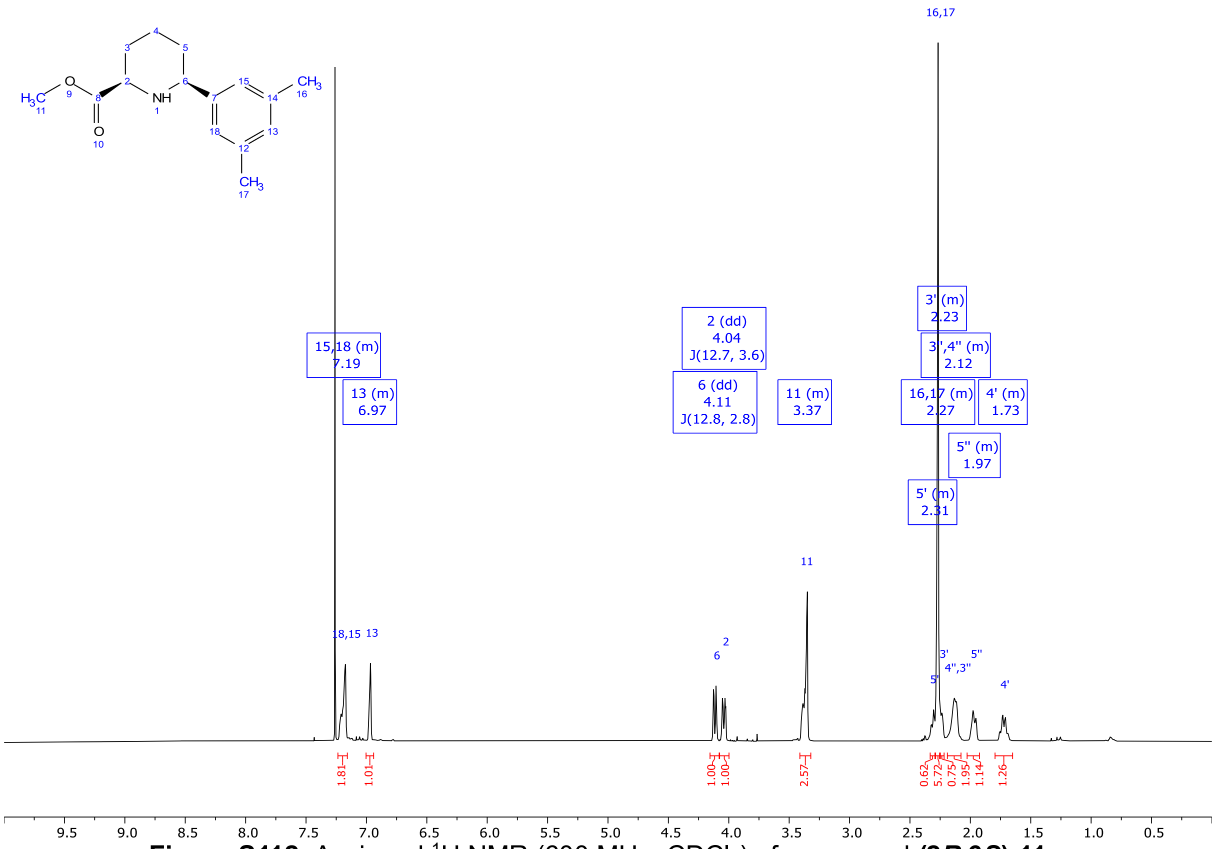

Figure S112: Assigned ^{1}H NMR (600 MHz, $CDCl_3$) of compound **(2*R*,6*S*)-11c**.

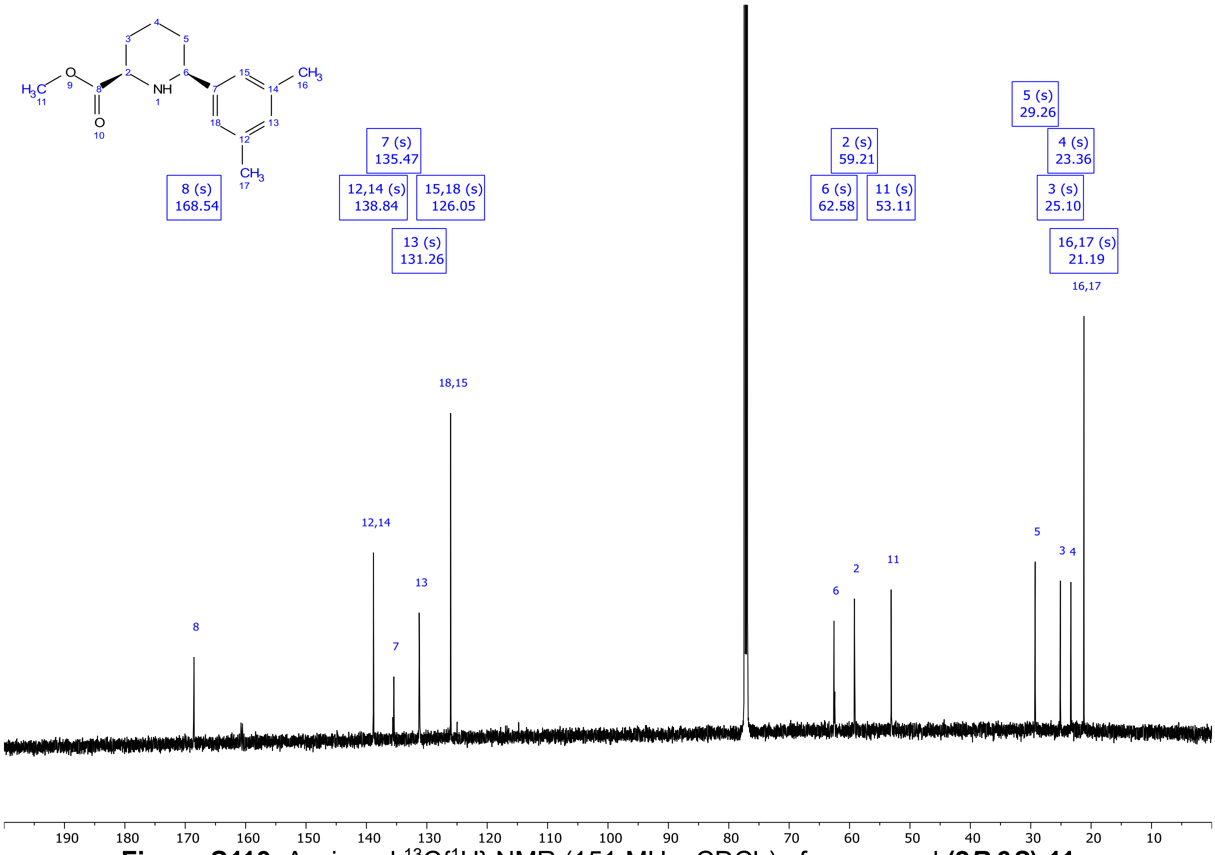

Figure S113: Assigned $^{13}C\{^{1}H\}$ NMR (151 MHz, $CDCl_3$) of compound **(2*R*,6*S*)-11c**.

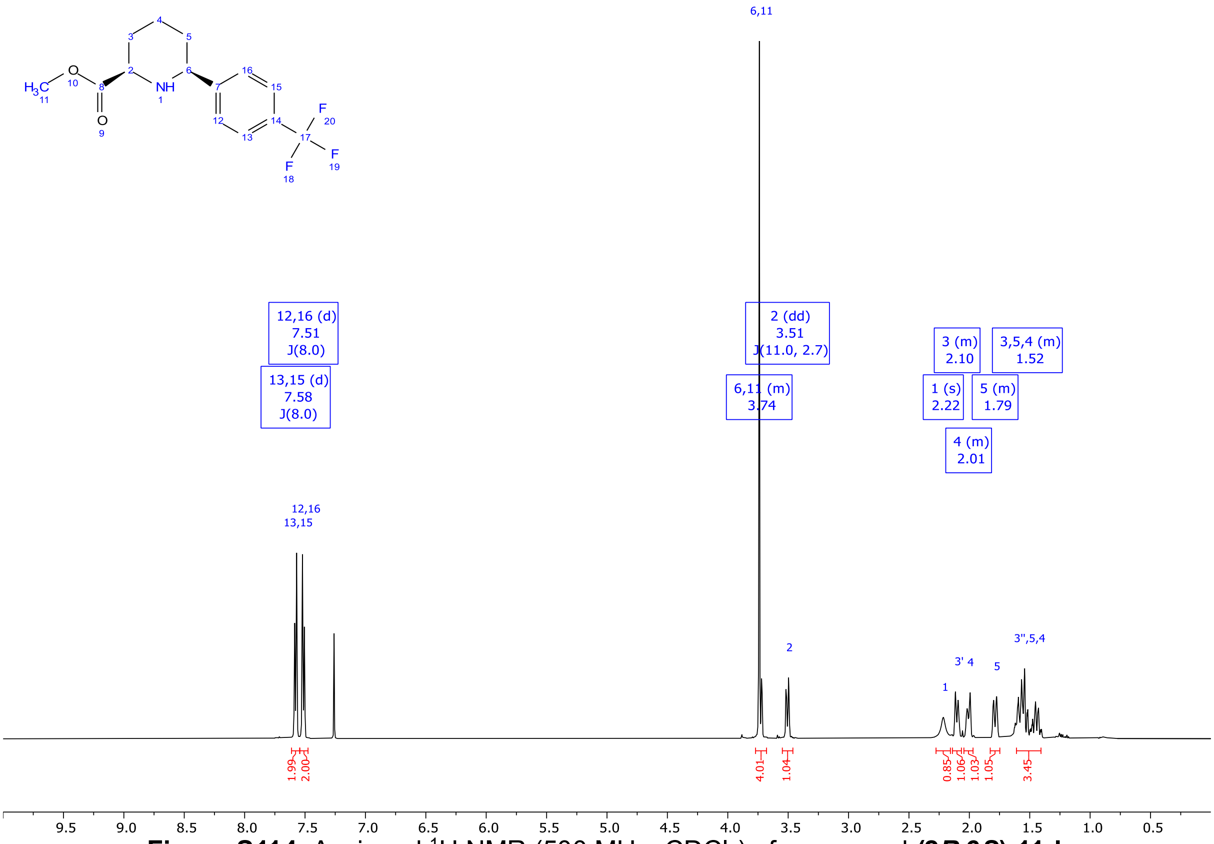

Figure S114: Assigned ^{1}H NMR (500 MHz, $CDCl_3$) of compound **(2*R*,6*S*)-11d**.

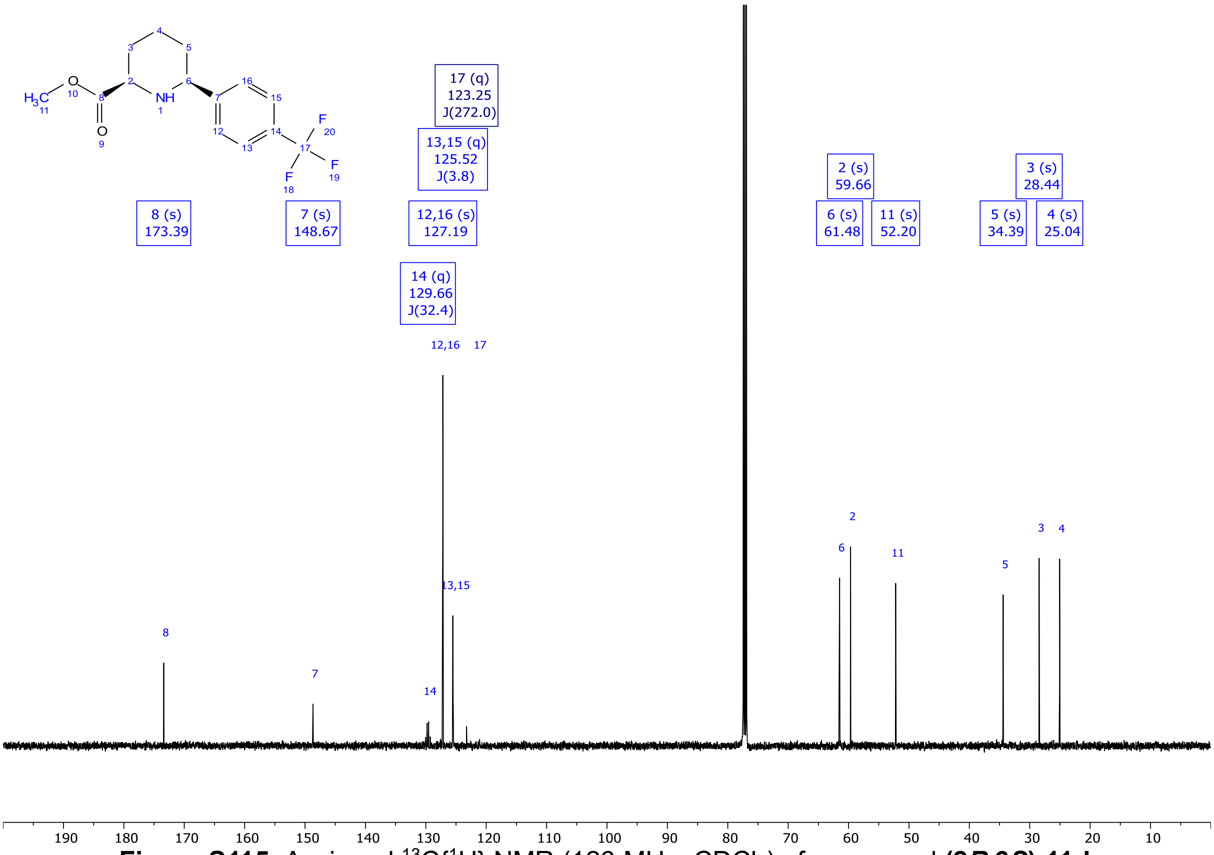

Figure S115: Assigned $^{13}C\{^{1}H\}$ NMR (126 MHz, $CDCl_3$) of compound **(2*R*,6*S*)-11d**.

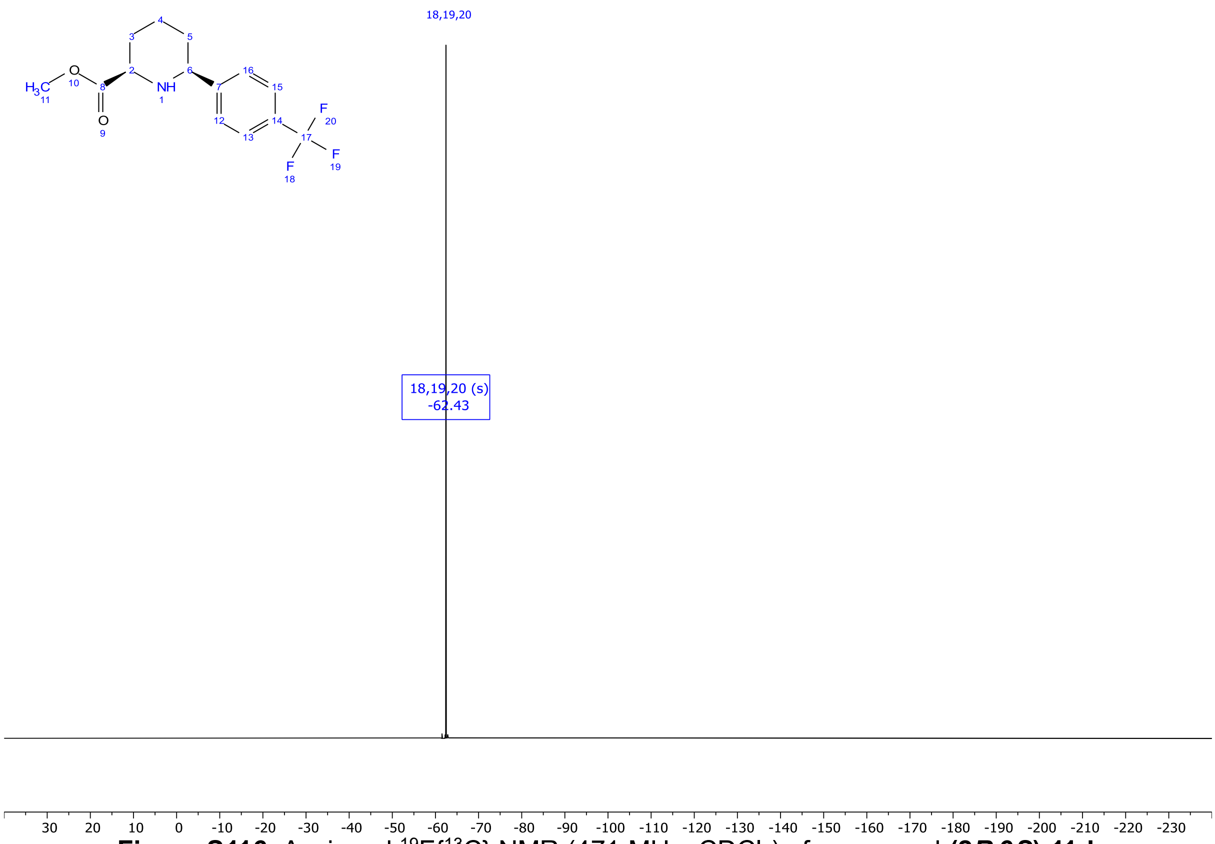

Figure S116: Assigned $^{19}F\{^{13}C\}$ NMR (471 MHz, $CDCl_3$) of compound **(2*R*,6*S*)-11d**.

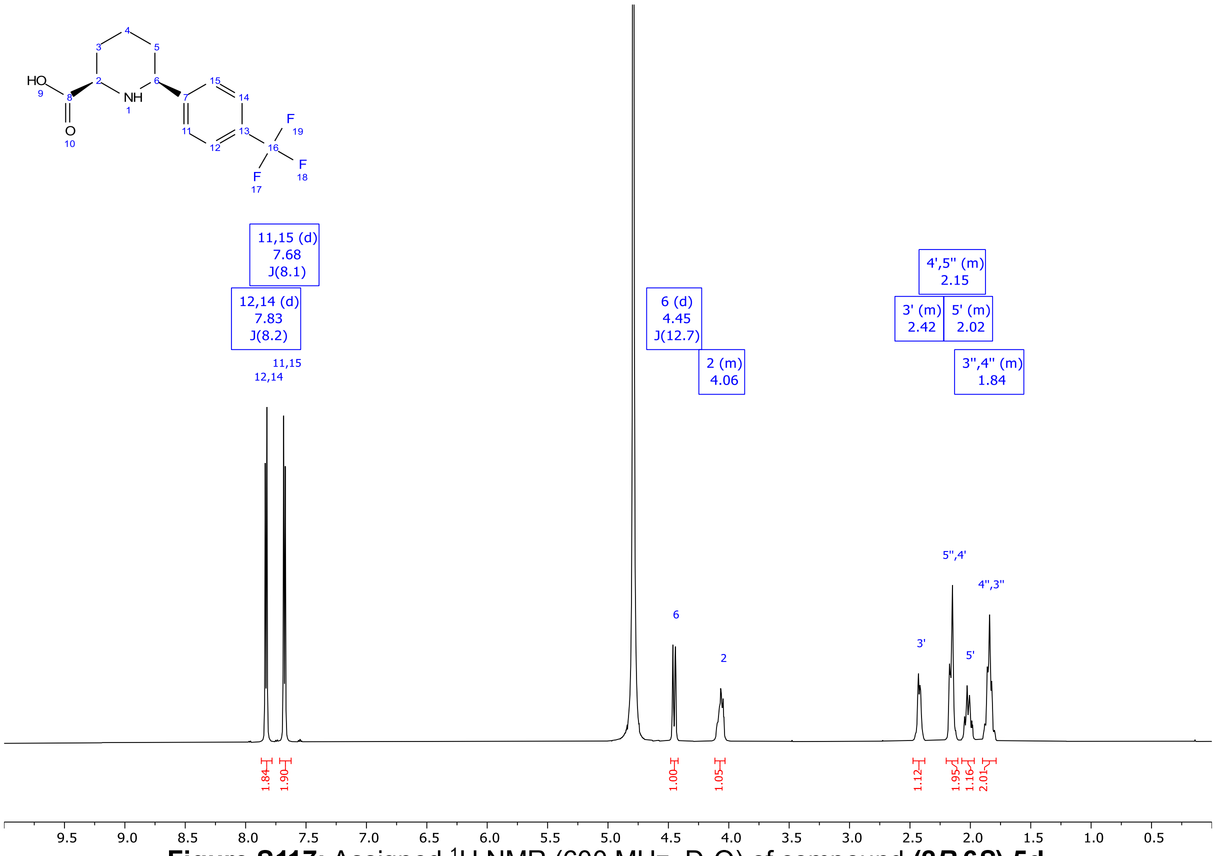

Figure S117: Assigned ^{1}H NMR (600 MHz, D_2O) of compound **(2*R*,6*S*)-5d**.

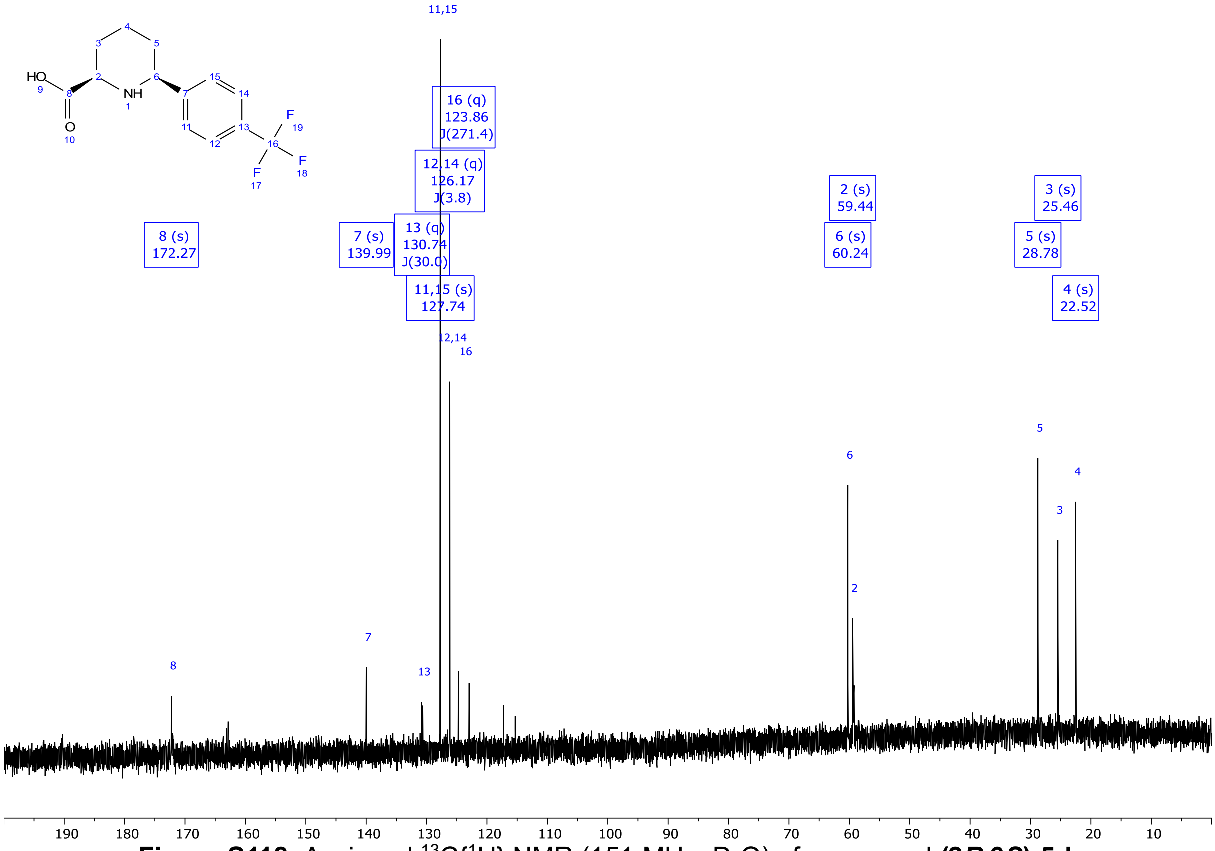

Figure S118: Assigned ^{13}C{^{1}H} NMR (151 MHz, D_2O) of compound **(2*R*,6*S*)-5d**.

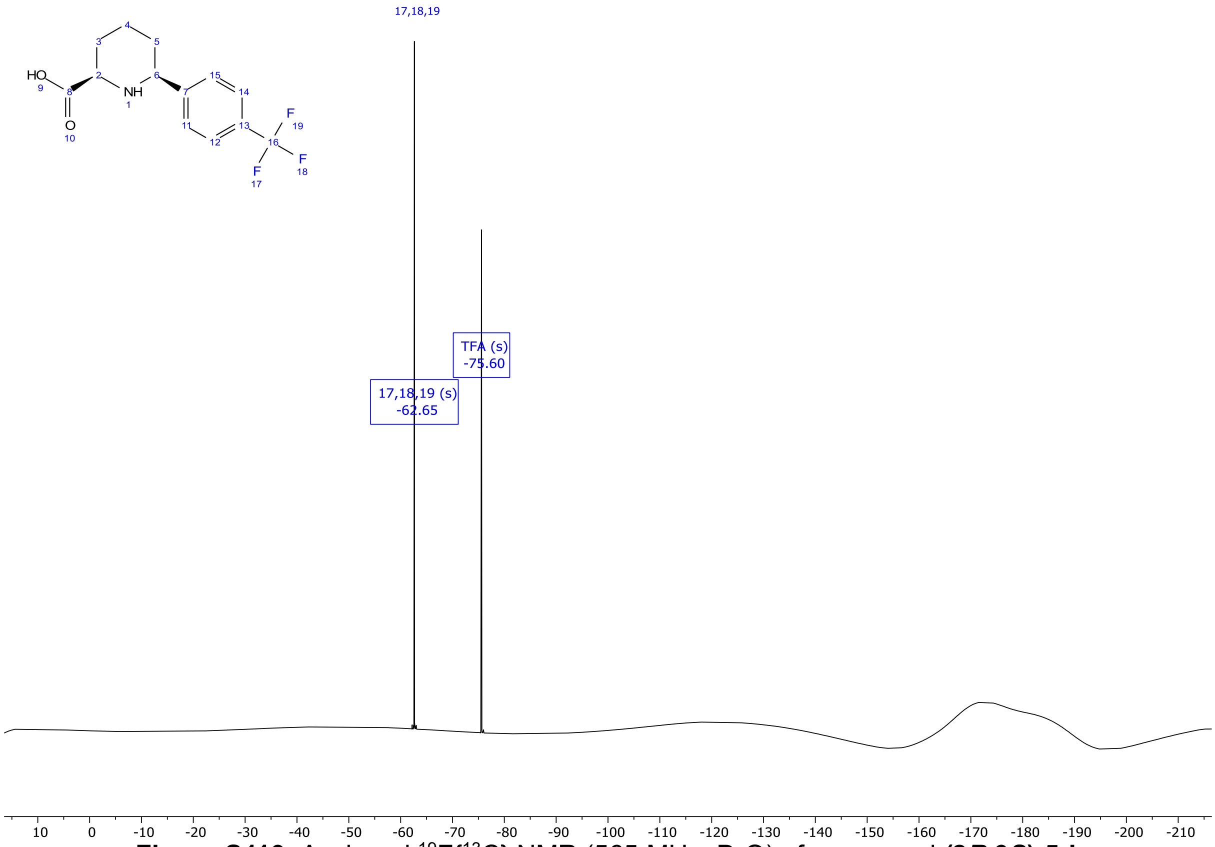

Figure S119: Assigned $^{19}F\{^{13}C\}$ NMR (565 MHz, D_2O) of compound **(2*R*,6*S*)-5d**.

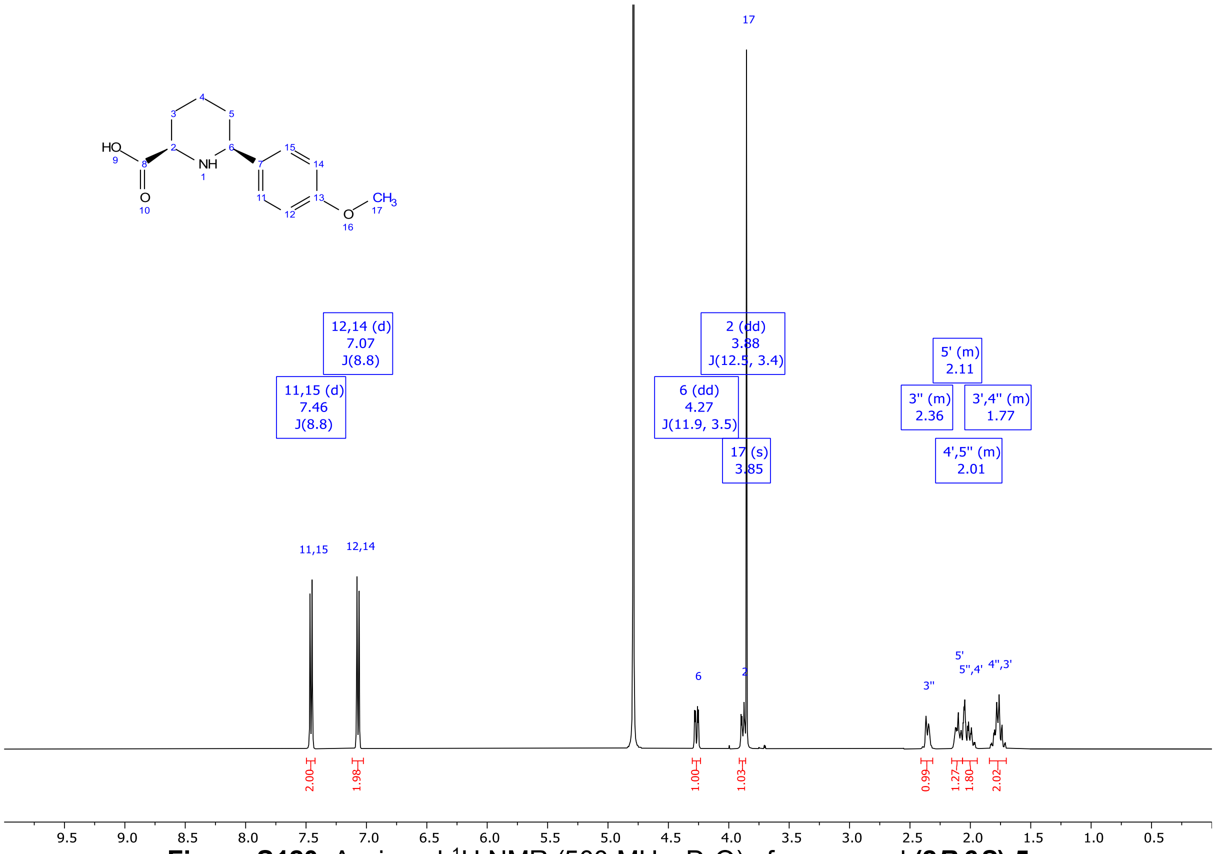

Figure S120: Assigned ^{1}H NMR (500 MHz, D_2O) of compound **(2*R*,6*S*)-5e**.

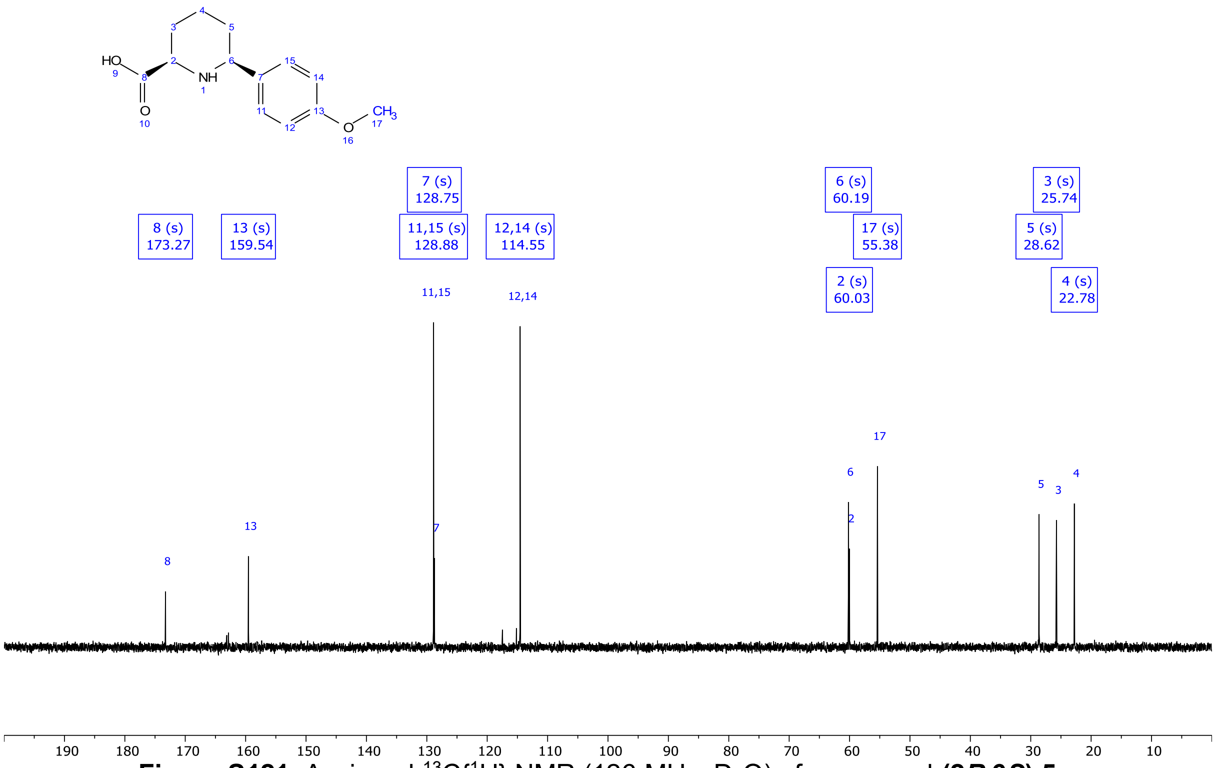

Figure S121: Assigned ^{13}C{^{1}H} NMR (126 MHz, D_2O) of compound **(2*R*,6*S*)-5e**.

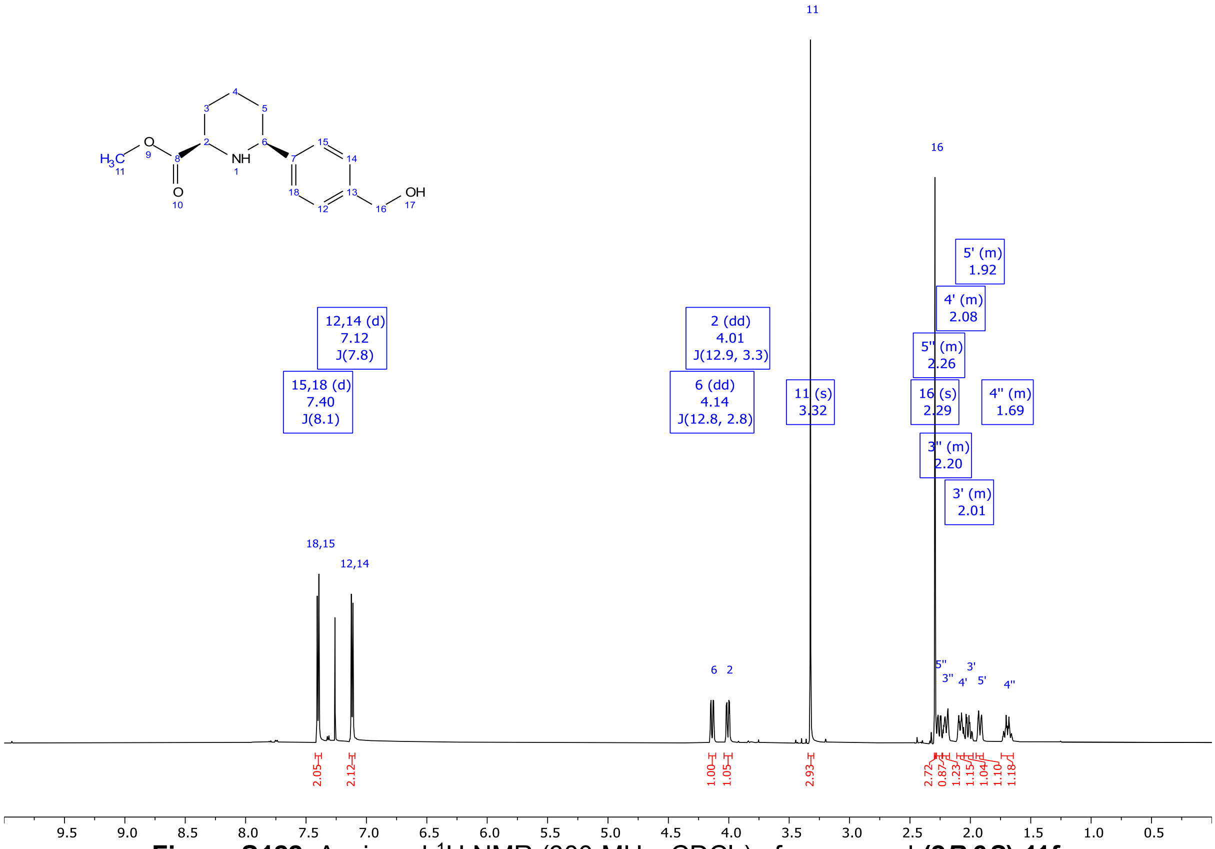

Figure S122: Assigned ^{1}H NMR (600 MHz, $CDCl_3$) of compound **(2*R*,6*S*)-11f**.

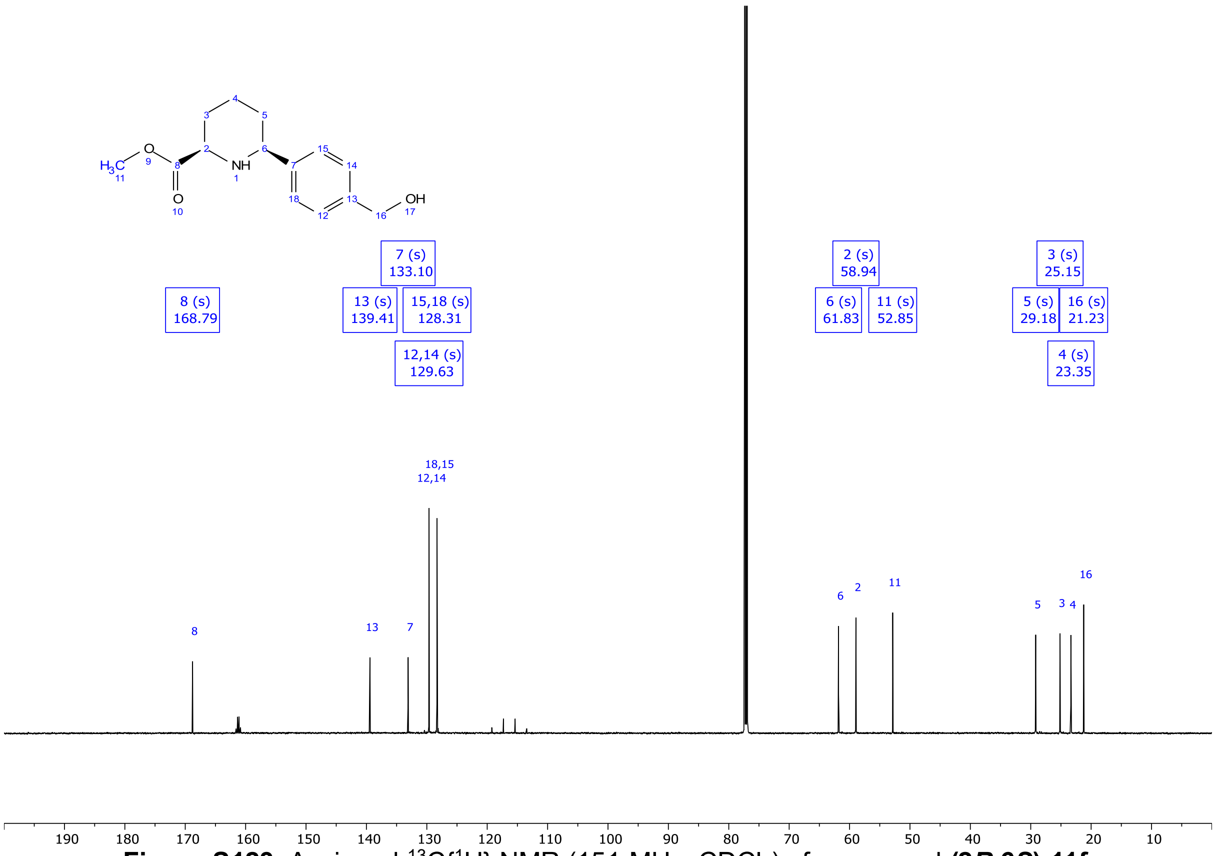

Figure S123: Assigned ^{13}C{^{1}H} NMR (151 MHz, $CDCl_3$) of compound **(2*R*,6*S*)-11f**.

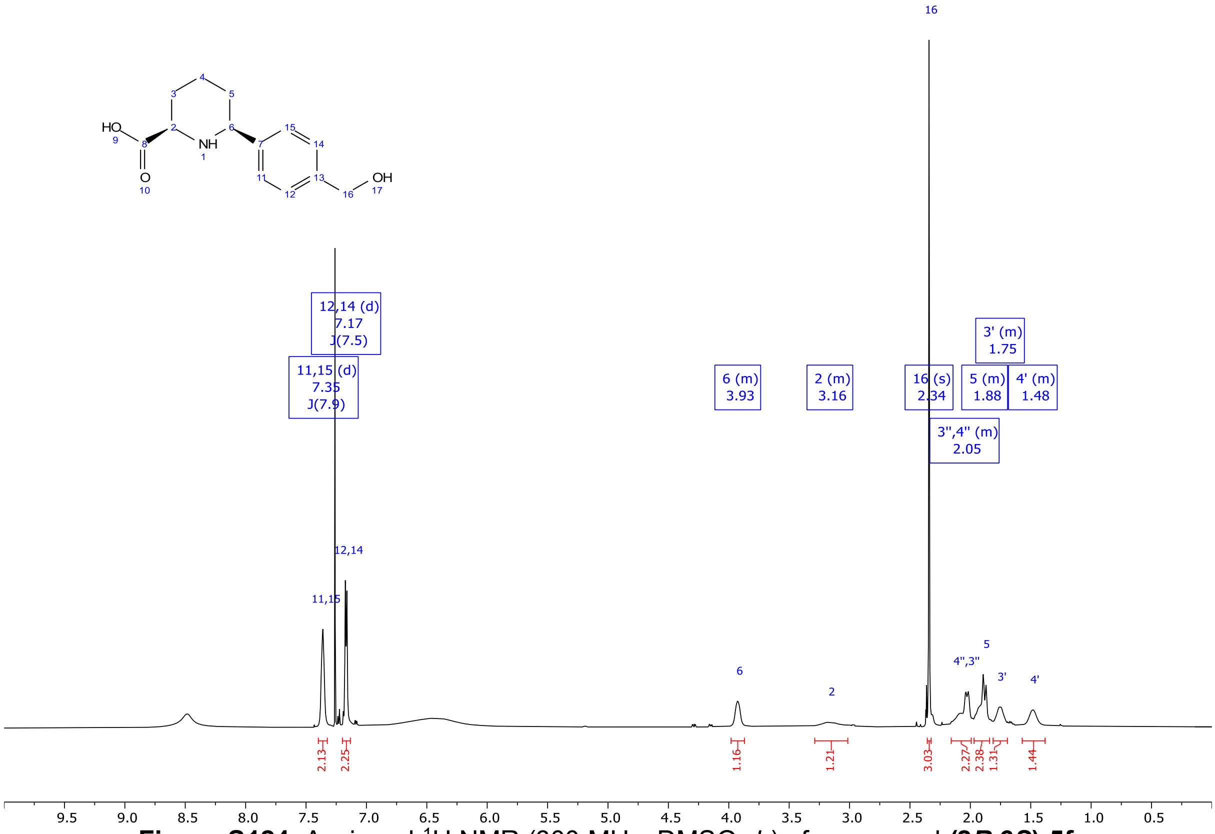

Figure S124: Assigned ^{1}H NMR (600 MHz, DMSO-d_6) of compound **(2*R*,6*S*)-5f**.

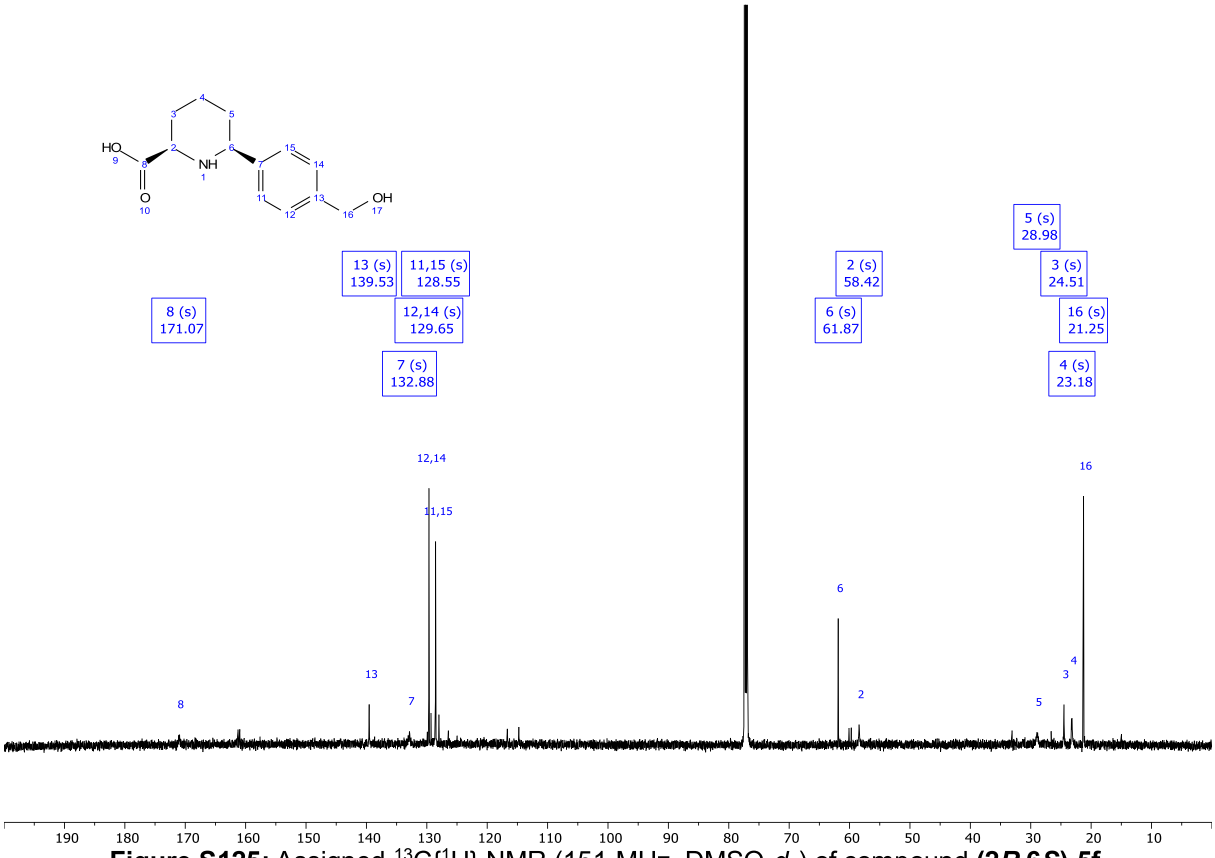

Figure S125: Assigned ^{13}C{^{1}H} NMR (151 MHz, DMSO-d_6) of compound **(2*R*,6*S*)-5f**.

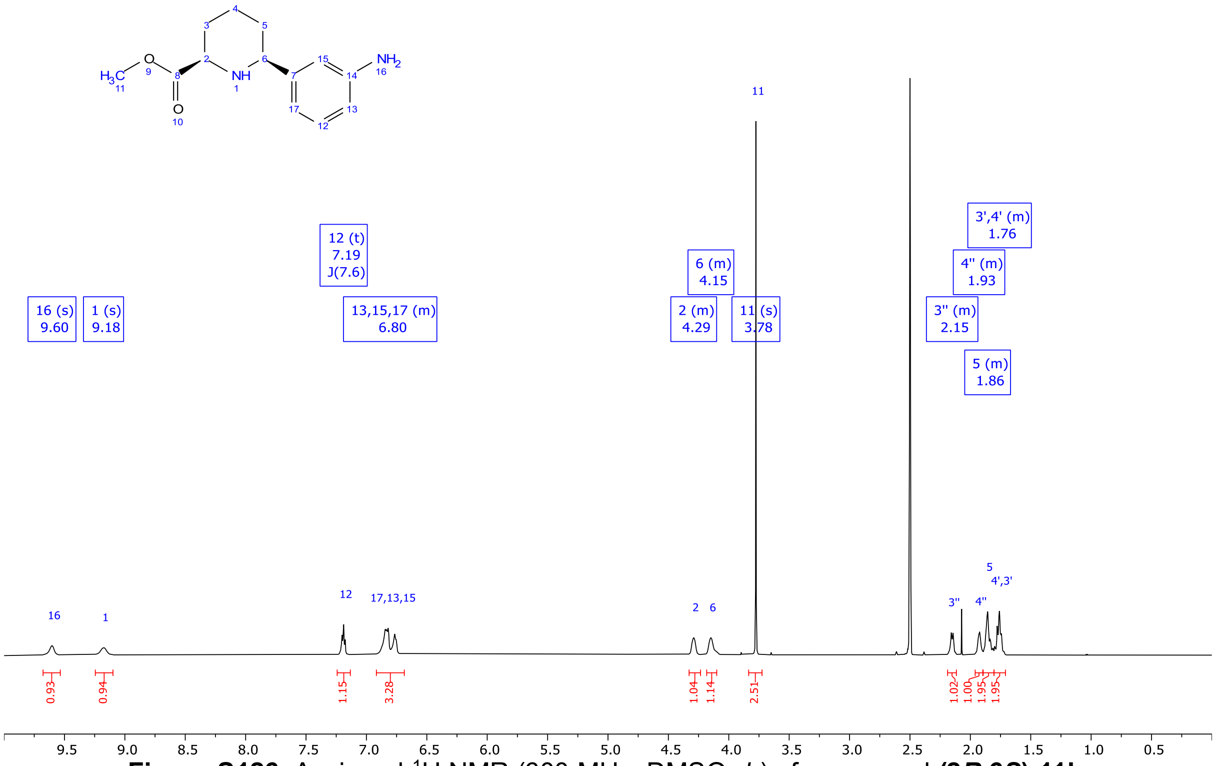

Figure S126: Assigned ^{1}H NMR (600 MHz, DMSO-*d*6) of compound **(2*R*,6*S*)-11h**.

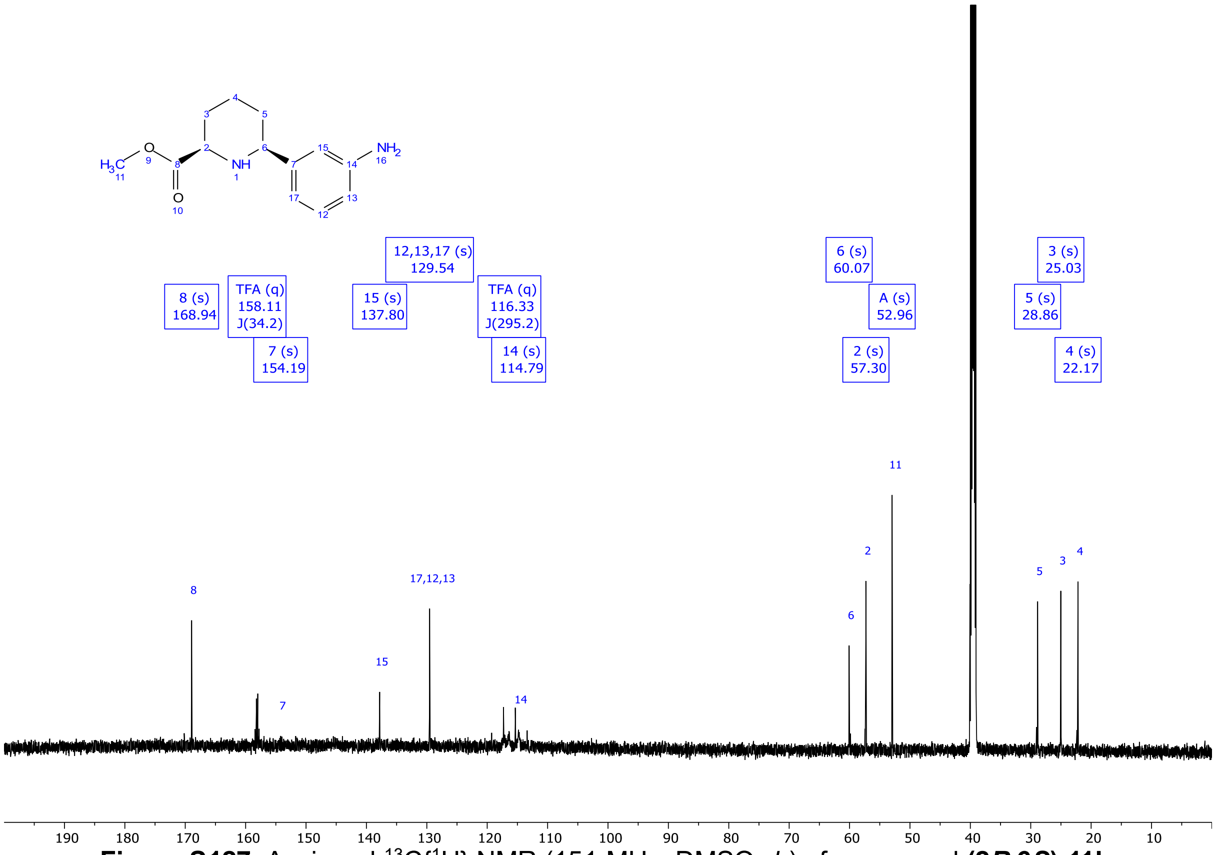

Figure S127: Assigned $^{13}C\{^{1}H\}$ NMR (151 MHz, DMSO-*d*6) of compound **(2*R*,6*S*)-11h**.

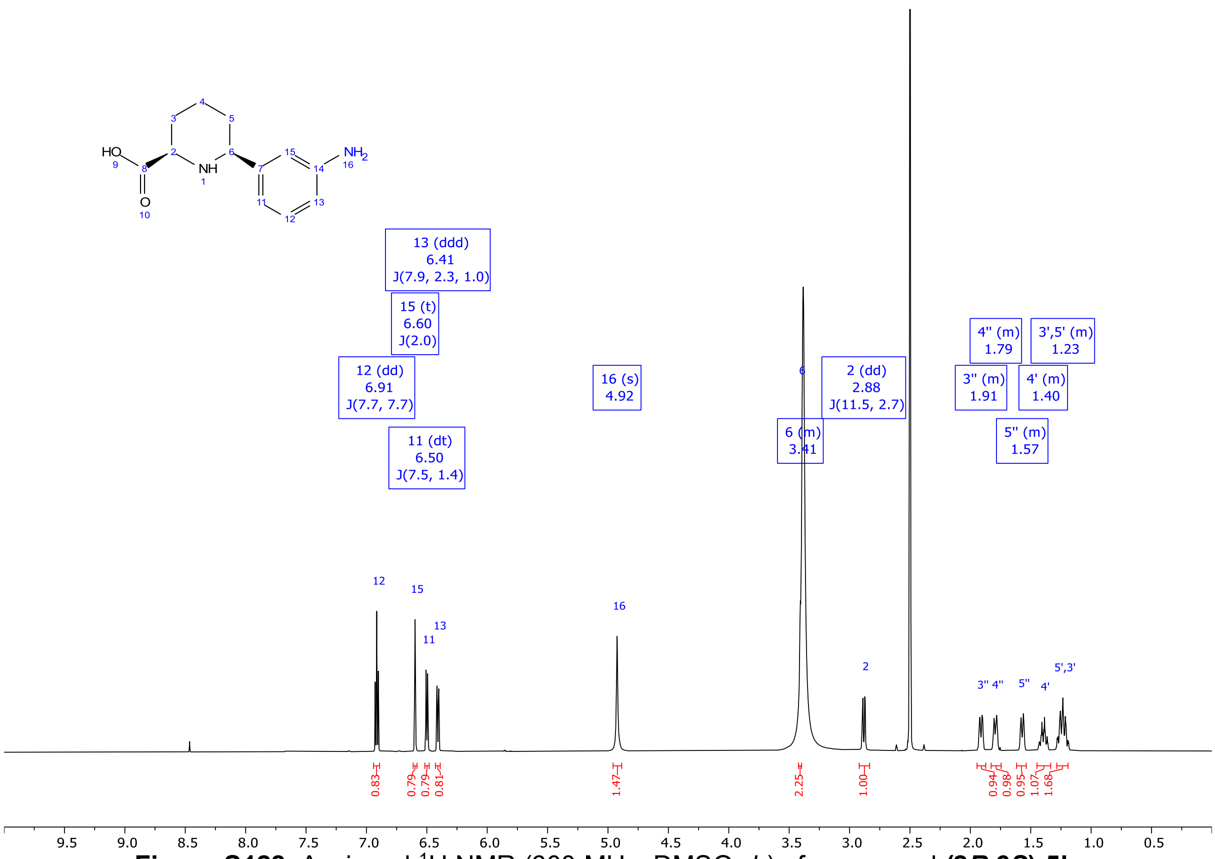

Figure S128: Assigned ^{1}H NMR (600 MHz, DMSO-d_6) of compound **(2*R*,6*S*)-5h**.

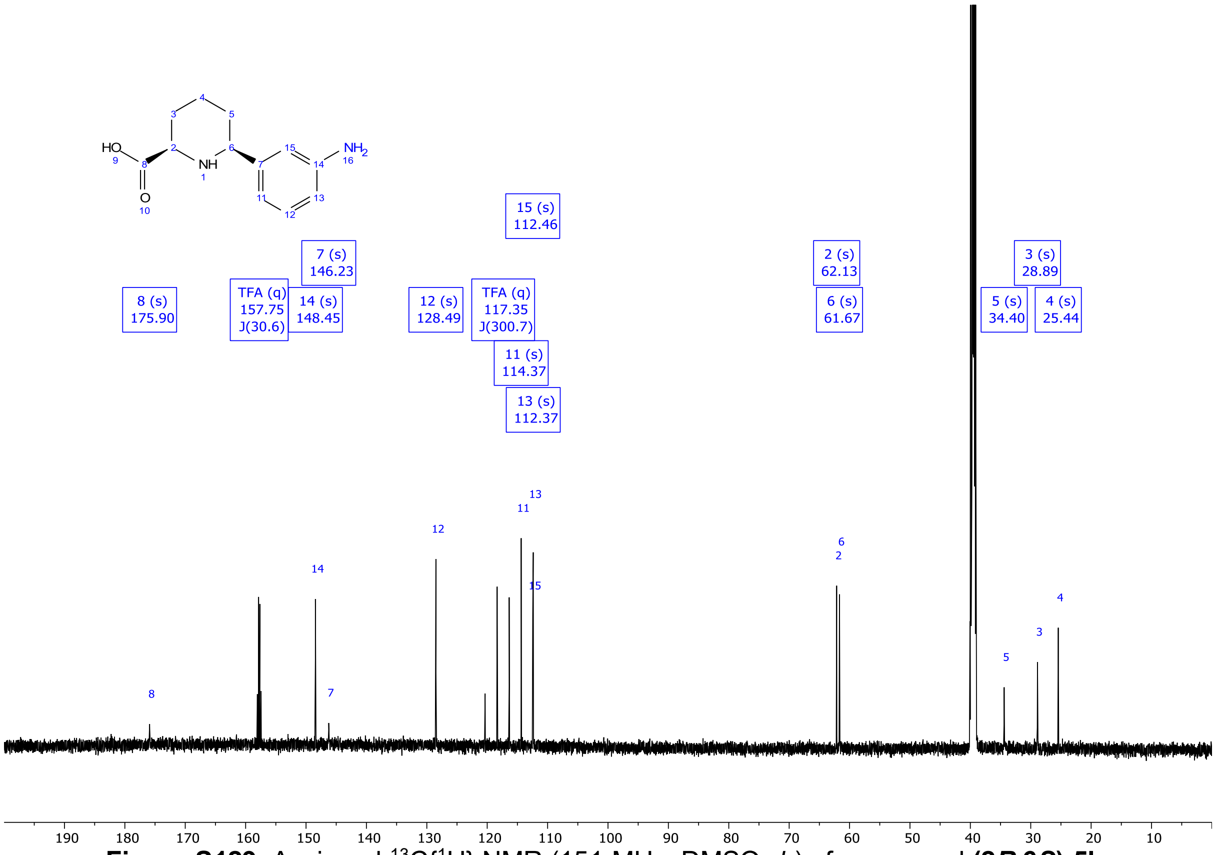

Figure S129: Assigned ^{13}C{^{1}H} NMR (151 MHz, DMSO-d_6) of compound **(2*R*,6*S*)-5h**.

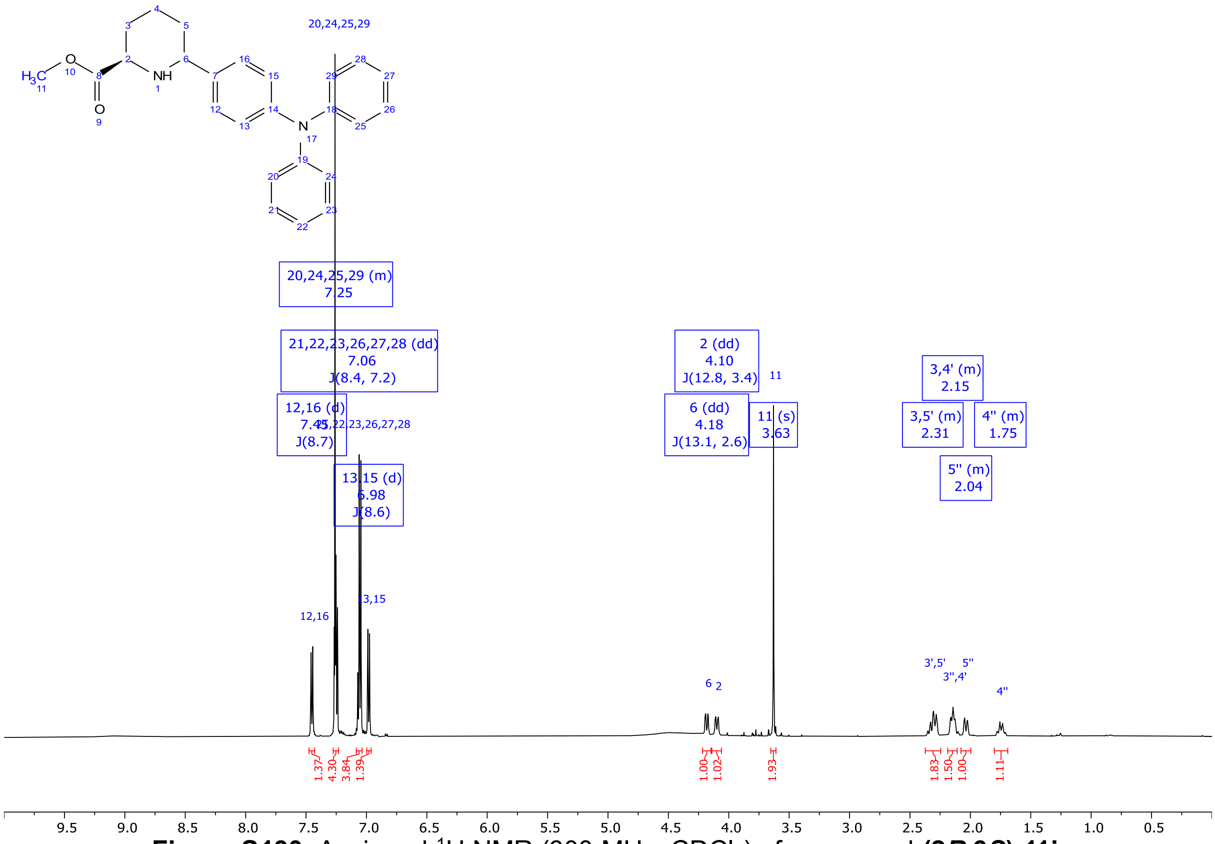

Figure S130: Assigned ^{1}H NMR (600 MHz, $CDCl_3$) of compound **(2*R*,6*S*)-11j**.

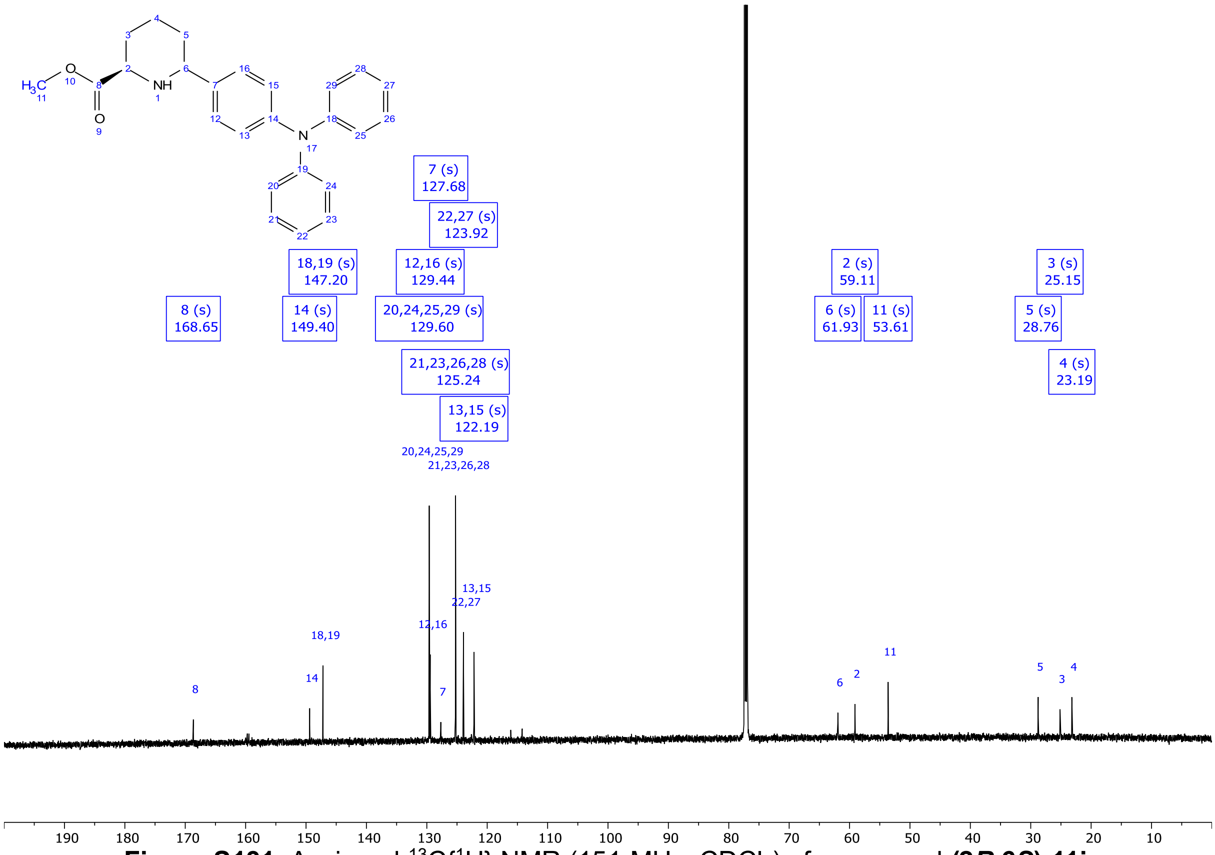

Figure S131: Assigned $^{13}C\{^{1}H\}$ NMR (151 MHz, $CDCl_3$) of compound **(2*R*,6*S*)-11j**.

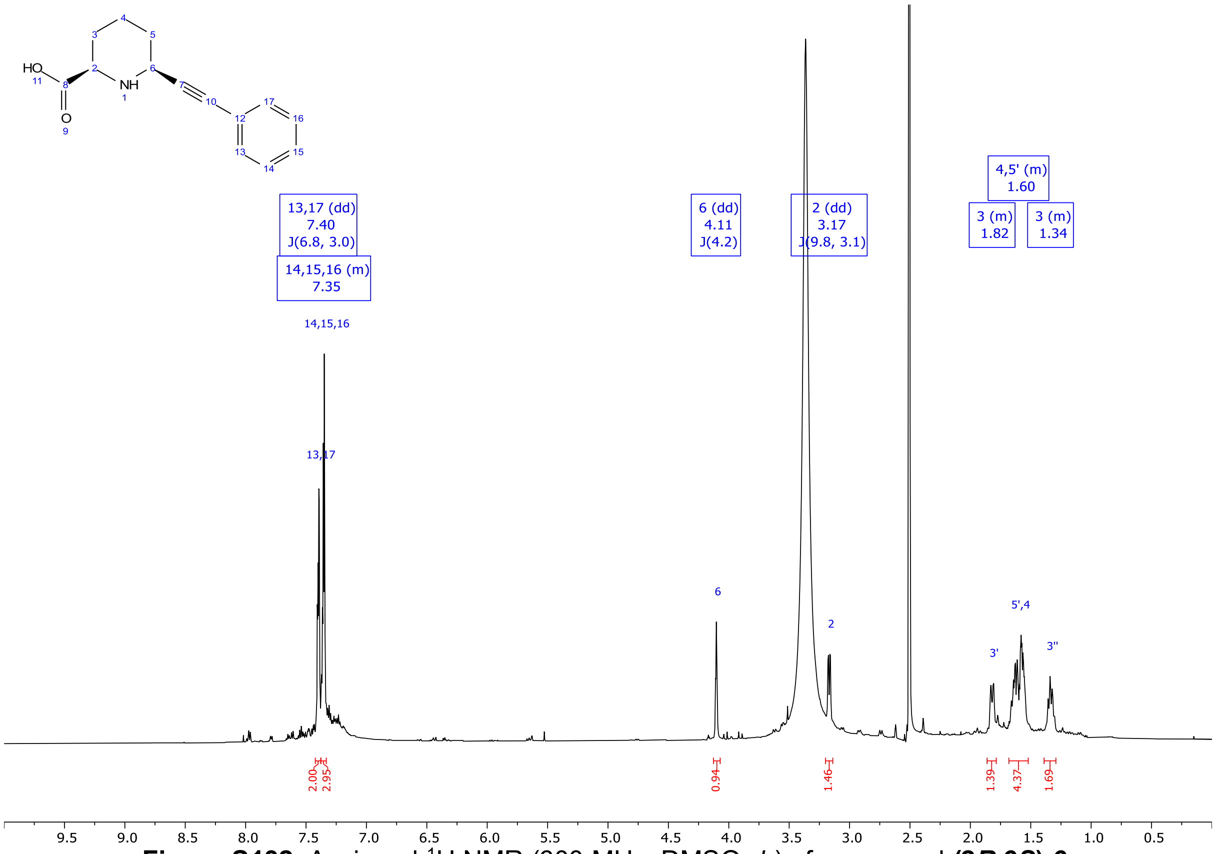

Figure S132: Assigned ^{1}H NMR (600 MHz, DMSO-d_6) of compound **(2*R*,6*S*)-6**.

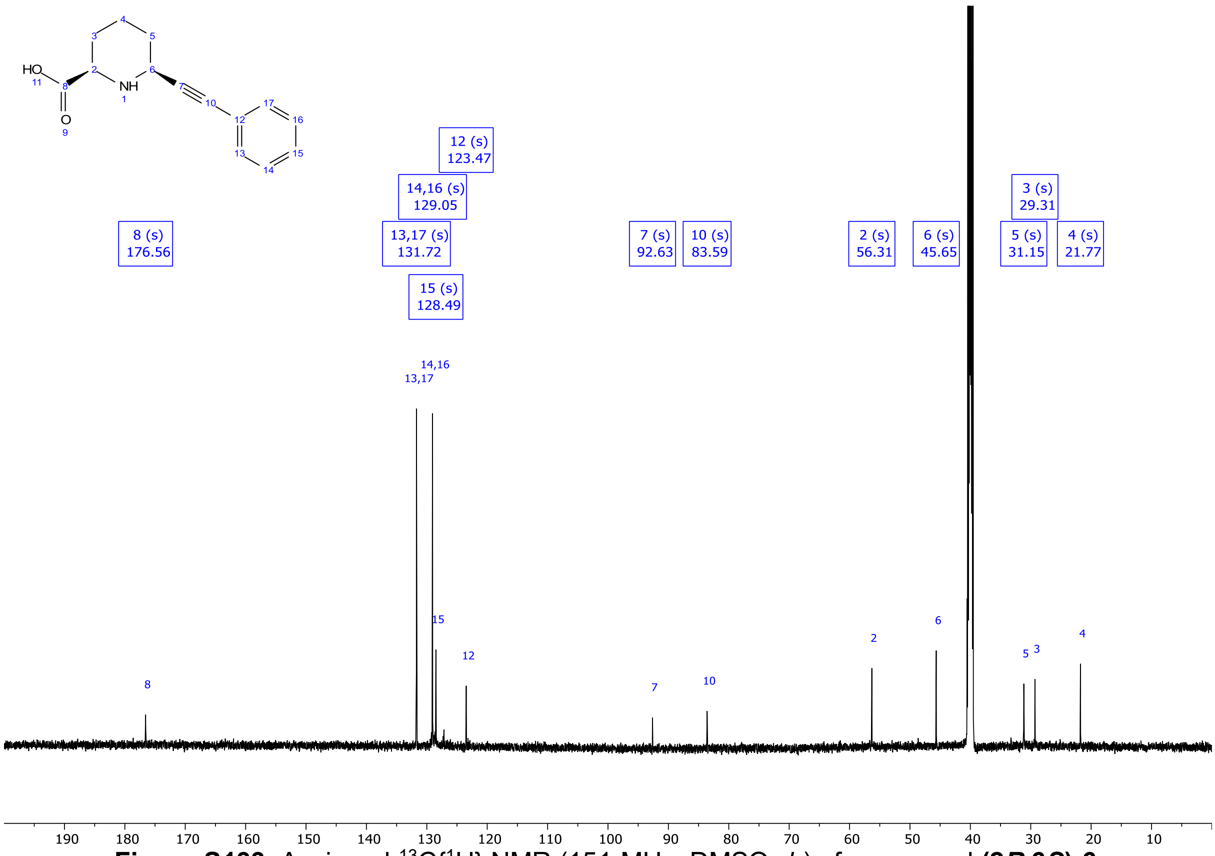

Figure S133: Assigned ^{13}C{^{1}H} NMR (151 MHz, DMSO-d_6) of compound **(2*R*,6*S*)-6**.

References

[1] Gable, K. P., Cycloalkanes, July 28, 2020, Oregon, USA. https://sites.science.oregonstate.edu/~gablek/CH334/Chapter4/bare_cyclohexane.htm (accessed October 06, 2024)

[2] Sunnam, S. K.; Schepmann, D.; Wibbeling, B.; Wünsch B., *Org. Biomol. Chem.*, **2010**, *8*, 3715-3722. doi.org/10.1039/C003878D

[3] Huang, S.-B.; Nelson, J. S.; Weller, D. D., *Synth. Commun.* **1989**, *19*, 3485-3496. doi.org/10.1080/00397918908052758

[4] Sadiq, A; Sewald, N., *Org. Lett.* **2013**, *15* (11), 2720-2722. doi.org/10.1021/ol4010728
